# DNA methylation status of the *SPHK1* and *LTB* genes underlies the clinicopathological diversity of non-alcoholic steatohepatitis-related hepatocellular carcinomas

**DOI:** 10.1007/s00432-022-04445-9

**Published:** 2022-11-08

**Authors:** Noboru Tsuda, Ying Tian, Mao Fujimoto, Junko Kuramoto, Satomi Makiuchi, Hidenori Ojima, Masahiro Gotoh, Nobuyoshi Hiraoka, Teruhiko Yoshida, Yae Kanai, Eri Arai

**Affiliations:** 1 Department of Pathology, Keio University School of Medicine, 35 Shinanomachi, Shinjuku-Ku, Tokyo, 160-8582 Japan; 2 Fundamental Innovative Oncology Core Center, National Cancer Center Research Institute, Tokyo, 104-0045 Japan; 3 Department of Diagnostic Pathology, National Cancer Center Hospital, Tokyo, 104-0045 Japan

**Keywords:** Genome-wide DNA methylation analysis, Non-alcoholic steatohepatitis, Hepatocellular carcinoma, *SPHK1*, *LTB*

## Abstract

**Purpose:**

This study was performed to identify the DNA methylation profiles underlying the clinicopathological diversity of non-alcoholic steatohepatitis (NASH)-related hepatocellular carcinomas (HCCs).

**Methods:**

Genome-wide DNA methylation analysis of 88 liver tissue samples was performed using the Infinium assay.

**Results:**

Principal component analysis revealed that distinct DNA methylation profiles differing from such profiles in normal control liver tissue had already been established in non-cancerous liver tissue showing NASH, which is considered to be a precancerous condition. Hierarchical clustering separated 26 NASH-related HCCs into Cluster I (*n* = 8) and Cluster II (*n* = 18). Such epigenetic clustering was significantly correlated with histopathological diversity, i.e. poorer tumor differentiation, tumor steatosis and development of a scirrhous HCC component. Significant differences in DNA methylation levels between the two clusters were accumulated in molecular pathways participating in cell adhesion and cytoskeletal remodeling, as well as cell proliferation and apoptosis. Among tumor-related genes characterizing Clusters I and II, differences in the levels of DNA methylation and mRNA expression for the *SPHK1*, *INHBA*, *LTB* and *PDE3B* genes were correlated with poorer tumor differentiation. 5-Aza-2′-deoxycytidine treatment of HCC cells revealed epigenetic regulation of the *SPHK1* and *LTB* genes. Knockdown experiments showed that SPHK1 promotes cell proliferation, represses apoptosis and enhances migration, whereas LTB enhances migration of HCC cells. DNA hypomethylation resulting in increased expression of *SPHK1* and *LTB* in poorly differentiated HCCs may underlie the aggressive phenotype of such HCCs.

**Conclusion:**

These data indicate that DNA methylation profiles may determine the clinicopathological heterogeneity of NASH-related HCCs via alterations of tumor-related gene expression.

**Supplementary Information:**

The online version contains supplementary material available at 10.1007/s00432-022-04445-9.

## Introduction

It is well known that not only genomic but also epigenomic alterations participate in the development and progression of cancers (Jones et al. 2016; Baylin and Jones 2016). Among epigenomic alterations, DNA methylation abnormalities play an important role in carcinogenesis through induction of chromosomal instability and alterations of tumor-related gene expression in multiple organs exposed to various carcinogens (Klustein et al. 2016). For example, we and other groups have reported the DNA methylation abnormalities occurring during multistage hepatocarcinogenesis related to hepatitis B virus (HBV) or hepatitis C virus (HCV) infection (Nagashio et al. 2011; Arai et al. 2009; Kaneto et al. 2001; Kanai et al. 1996). On the other hand, in recent years, non-alcoholic steatohepatitis (NASH), a hepatic manifestation of metabolic syndrome resulting in the development of liver cirrhosis, has shown an alarming increase (Sheka et al. 2020). Although viral hepatitis followed by liver cirrhosis used to be the main cause of hepatocellular carcinoma (HCC), there is now evidence that NASH is becoming another precancerous condition for HCC (Huang et al. 2021).

Our previous genome-wide DNA methylation analysis using the Infinium assay with specimens of pathological tissue has revealed NASH-specific DNA methylation profiles that differ from such profiles in liver tissue specimens showing viral hepatitis and/or cirrhosis (Kuramoto et al. 2017). DNA methylation alterations induced under the precancerous NASH conditions were inherited by or strengthened in NASH-related HCCs themselves in the same affected individuals (Kuramoto et al. 2017). Moreover, based on comparison between samples of non-cancerous liver tissue and NASH-related HCCs, we have identified tumor-related genes such as *WHSC1* (Kuramoto et al. 2017), *TRIM4*, *PRC1* and *TUBA1B* (Tian et al. 2020), whose expression levels are regulated by DNA methylation status as potential therapeutic targets in NASH-related HCCs.

On the other hand, even among NASH-related HCCs, clinicopathological heterogeneity is frequently observed (Anstee et al. 2019). Even though it has been shown that DNA methylation alterations determine histological diversity, tumor aggressiveness and patient outcome in various organ cancers (Endo et al. 2021; Yang et al. 2020; Makabe et al. 2019), correlations between clinicopathological heterogeneity and DNA methylation profiles have not yet been fully clarified in NASH-related HCCs. In the present study aimed at identifying the DNA methylation profiles underlying the clinicopathological diversity of NASH-related HCCs, we performed genome-wide DNA methylation screening of 88 liver tissue specimens using the Infinium assay (Bibikova et al. 2009), in combination with meticulous histopathological examinations.

## Materials and methods

### Patients and tissue samples

For the present analysis, we used 26 paired samples of non-cancerous liver tissue (N) and the corresponding tumorous tissue (T) obtained by partial hepatectomy from 26 HCC patients whose N samples showed histological features compatible with NASH. All 26 patients were negative for HBV surface antigen and anti-HCV antibody. NASH stage was evaluated microscopically on the basis of the histological scoring system for NASH and the Brunt classification criteria (Brunt et al. 2011). The HCCs were diagnosed histologically in accordance with the World Health Organization classification (Torbenson et al. 2019) and the Tumor-Node-Metastasis classification (Brierley et al. 2017). Moreover, in each tumor, the percentage of tumor cells with lipid droplets among all observed tumor cells was evaluated in 10 fields of view at 100x. The presence or absence of fibrous tissue spreading along the sinusoidal space within each tumor was evaluated in 10 fields of view at 20x, and the areal ratio of the scirrhous HCC component was calculated as the ratio of such spreading-positive fields among the 10 evaluated fields. For comparison, 36 samples of normal control liver tissue (C), obtained by partial hepatectomy from 36 patients with liver metastases of primary colorectal cancers without HBV or HCV infection, chronic hepatitis, liver cirrhosis or HCC, were examined.

None of the patients had received preoperative treatment, and all underwent surgery at the National Cancer Center Hospital, Tokyo, Japan. The age, sex and clinicopathological parameters of the 26 patients from whom N and T samples were obtained and the 36 patients from whom C samples were obtained are summarized in Supplementary Table 1. Immediately after surgical removal, tissue specimens were frozen and stored in liquid nitrogen at the National Cancer Center Biobank, Tokyo, Japan, until use in research, in accordance with the Japanese Society of Pathology Guidelines for the handling of pathological tissue samples for genomic research (Kanai et al. 2018). This study was approved by the Ethics Committees of the National Cancer Center, Tokyo, Japan, and Keio University, and was performed in accordance with the Declaration of Helsinki. All of the patients provided written informed consent prior to inclusion of their specimens in the study.

### Infinium assay

High-molecular-weight DNA was extracted from fresh-frozen tissue samples using phenol–chloroform, followed by dialysis. Five hundred nanograms of genomic DNA was subjected to bisulfite treatment using an EZ DNA Methylation-GoldTM Kit (Zymo Research, Irvine, CA) in accordance with the manufacturer’s protocol. DNA methylation status at 485,577 CpG loci was examined at single-CpG resolution using the Infinium HumanMethylation450 BeadChip (Illumina, San Diego, CA) (Bibikova et al. 2009). After hybridization, the specifically hybridized DNA was fluorescence-labeled by a single-base extension reaction and detected using an iScan reader (Illumina) in accordance with the manufacturer’s protocol.

The data were then assembled using GenomeStudio methylation software (Illumina). At each CpG site, the ratio of the fluorescence signal was measured using a methylated probe relative to the sum of the methylated and unmethylated probes, i.e. the so-called β-value, which ranges from 0.00 to 1.00, reflecting the methylation level of an individual CpG site. Some of the results of the Infinium assay had been used in our previous study focusing on comparison with viral hepatitis-related HCCs and deposited in the Gene Expression Omnibus (GEO) database (https://www.ncbi.nlm.nih.gov/geo/, Accession number GSE183468).

### Pathway analysis

MetaCoreTM software (version 19.3) (Thomson Reuters, NY) is a pathway analysis tool based on a proprietary manually curated database of human protein–protein, protein-DNA and protein compound interactions. Using genes showing significant differences in DNA methylation levels between epigenomic clusters, MetaCore pathway analysis by GeneGo was performed. Such genes were considered significantly enriched in pathways for which the false discovery rate (FDR) was less than 0.05.

### Real-time quantitative reverse transcription (RT)-PCR analysis

Total RNA was isolated from all 26 paired N and T samples, and 31 C samples from which tissue samples were available even after DNA extraction, using TRIzol reagent (Life Technologies, Carlsbad, CA). cDNA was generated from total RNA using random primers and SuperScript IV Reverse Transcriptase (Invitrogen, Carlsbad. CA). Levels of expression of mRNA for the *SPHK1* (*sphingosine kinase 1*), *INHBA* (*inhibin*, *beta A*), *LTB* (*lymphotoxin beta*) and *PDE3B* (*phosphodiesterase 3B*) genes were determined using the PowerUp SYBR Green Master Mix (Applied Biosystems, Foster City, CA) on the 7500 Fast Real-Time PCR system (Applied Biosystems) employing the relative standard curve method. PCR primers were designed using the Primer Designer software (Thermo Fisher Scientific, UK, https://www.thermofisher.com/order/genome-database/) and Primer-BLAST software (https://www.ncbi.nlm.nih.gov/tools/primer-blast/index.cgi). The sequences of the PCR primer sets employed are shown in Supplementary Table 2. Experiments were performed in triplicate, and the mean value for the three determinations was used as the threshold cycle (Ct) value. All Ct values were normalized to that of the *GAPDH* gene in the same sample.

### Cell lines

The human HCC cell line Hep3B (Knowles et al. 1980) was purchased from the American Type Culture Collection (Manassas, VA) in February 2020. The human HCC cell lines PLC/PRF/5 (MacNab et al. 1976), JHH-7 (Fujise et al. 1990) and HLF (Dor et al. 1975) were purchased from the Japanese Collection of Research Bioresources (JCRB) (Osaka, Japan) in January 2020. Hep3B, PLC/PRF/5, JHH-7 and HLF were authenticated based on short tandem repeat analysis by JCRB Cell Bank in August 2022 (certification numbers: KBN0850-01, KBN0850-02, KBN0850-03 and KBN0850-04, respectively). It was confirmed that all cell lines used were mycoplasma-free. JHH-7, HLF and PLC/PRF/5 were maintained in D6046 medium (Sigma-Aldrich, St. Louis, MO) supplemented with 10% fetal bovine serum, under 95% air and 5% CO2 at 37 °C. Hep3B was maintained in M4655 medium (Sigma-Aldrich), supplemented with S8636 sodium pyruvate solution (Sigma-Aldrich), M7145 non-essential amino acid solution (Sigma-Aldrich) and 10% fetal bovine serum, under 95% air and 5% CO_2_ at 37 °C.

### 5-Aza-2′-deoxycytidine (5-aza-dC) treatment of cell lines

JHH-7 and HLF cells were seeded at a density of 9 × 10^5^ cells per 15-cm dish on day 0 and then allowed to attach for 24 h. Then, 5-aza-dC (Sigma-Aldrich, St. Louis, MO) was added to a final concentration of 1 μM. Cells were passaged on day 3. At 24 h after replacing, 5-aza-dC was added again to the same final concentration. Control cells were treated with dimethyl sulfoxide. Genomic DNA and total RNA were extracted from both cell lines on day 6.

### Transfection with small interfering RNA (siRNA)

Hep3B cells were seeded in 96-well plates at a concentration of 5 × 10^6^ cells/well and PLC/PRF/5 cells at 1 × 10^6^ cells/well. When the cells had reached about 60% confluence, the medium was replaced with Opti-MEM^®^ I Reduced Serum Medium (Thermo Fisher Scientific). The cells were then transfected with either the negative control siRNA (siNC), *SPHK1*-specific siRNA (s16958 and s16959) or *LTB*-specific siRNA (s194597, s8311 and s8312) (Thermo Fisher Scientific) using LipofectamineTM RNAiMAX reagent (Thermo Fisher Scientific). At 48 h after transfection, the levels of expression of mRNAs for *SPHK1* or *LTB* were determined by quantitative real-time RT-PCR analysis, using *GAPDH* as the reference gene. Transfected cells were then subjected to the 3-(4,5-dimethylthiazol-2-yl)-5-(3-carboxymethoxyphenyl)-2-(4-sulfophenyl)-2H-tetrazolium (MTS) cell viability assay, cell apoptosis assay and cell migration assay.

### MTS cell viability assay

The MTS cell viability assay was performed as described previously (Hamada et al. 2021; Arai et al. 2015). Briefly, 48 h after transfection with negative control siRNA and *SPHK1*- or *LTB*-specific siRNAs, cells were treated with CellTiter 96 Aqueous One Solution Reagent (Promega, Madison, WI). After 1 h of treatment, the optical density was measured at 490 nm on a GloMax^®^-Multi + Detection System Glomax (Promega). Results were presented as the mean ± standard deviation for three separate determinations.

### Apoptosis assay

The apoptosis assay was performed as described previously (Hamada et al. 2021; Arai et al. 2015). Briefly, 48 h after transfection with negative control siRNA and *SPHK1*- or *LTB*-specific siRNAs, cells were treated with a Caspase-Glo 3/7 assay kit (Promega). After 1 h of incubation, the luminescent signal was measured on a GloMax-Multi + Detection System Glomax (Promega). Results were presented as the mean ± standard deviation for three separate determinations.

### Cell migration assay

The cell migration assay was performed as described previously (Hamada et al. 2021; Arai et al. 2015). Cell migration was determined using 24-well transwell chambers with an 8-μm pore polycarbonate filter (Corning Inc., Corning, NY). Forty-eight hours after transfection with the negative control siRNA and *SPHK1*- or *LTB*-specific siRNAs, 5 × 10^4^ PLC/PRF/5 and Hep3B cells were seeded onto the upper-side transwells in 100 μl of serum-free medium, and 500 μl of the complete medium was added to the lower chamber. The cells were incubated to allow migration for 48 h at 37 °C and 5% CO_2_. At the end of the assay, the non-motile cells on the top surface of the inserts were removed with cotton swabs. Cells that had passed through the polycarbonate membrane were fixed with 10% formalin and stained with 0.5% crystal violet to visualize the attached cells. The crystal violet was eluted with 10% acetic acid and the optical density was measured at 600 nm on a GloMax Multi Detection System (Promega). Results were presented as the mean ± standard deviation for three separate determinations.

### Statistical analysis

In the Infinium assay, the call proportions (*P* < 0.01 for detection of signals above the background) for 859 probes in all of the examined tissue samples were less than 90%. Since such a low proportion may due to polymorphism at the probe for CpG sites, these 859 probes were excluded from subsequent analysis, as described previously (Fujimoto et al. 2020; Tsumura et al. 2019). In addition, 45 probes with missing β values in more than 10% of the samples were excluded. Finally, probes on chromosomes X and Y were removed to avoid any gender-specific methylation bias, leaving a final total of 473,332 autosomal CpG sites.

Differences in levels of DNA methylation and mRNA expression between sample groups were examined by Welch’s *t* test. To correct for multiple testing, we used Bonferroni correction. The DNA methylation profiles were analyzed using principal component analysis (PCA) and hierarchical clustering (Euclidean distance, Ward’s method). Correlations between epigenetic clustering and clinicopathological parameters were tested by Welch’s *t* test and Fisher’s exact test. All statistical analyses were performed using the programing language R. Differences at *P* values of less than 0.05 were considered statistically significant.

## Results

### DNA methylation profiles during NASH-related multistage hepatocarcinogenesis

**Fig. 1:**
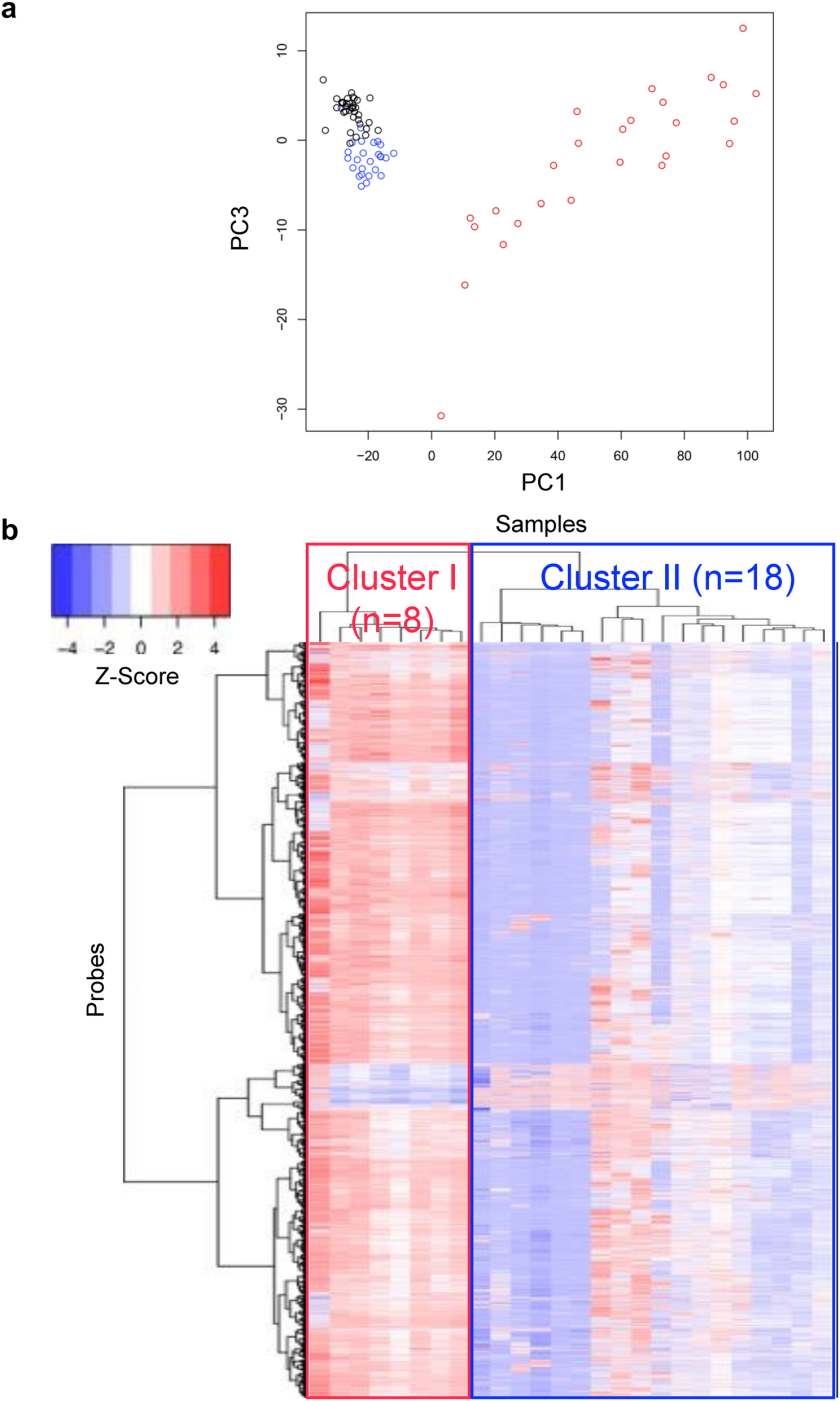
Principal component analysis (**a**) and hierarchical clustering (**b**) using the 64,027 probe CpG sites showing significant differences in DNA methylation levels between normal control liver tissue (C, black circles) and tumorous tissue (T, red circles) samples (Welch’s *t* test, *P* < 0.05 after Bonferroni correction, Δβ_T−C_ value of more than 0.2 or less than − 0.2) in liver tissue specimens. **a** Distinct DNA methylation profiles, differing from such profiles in C samples (*n* = 36), were established even in non-cancerous liver tissue (N, blue circles) samples showing histological features compatible with non-alcoholic steatohepatitis (NASH) (*n* = 26). Moreover, T samples themselves were scattered over a wider area on the scattergram, reflecting heterogeneity of the DNA methylation profiles of T. **b** Based on hierarchical clustering (Euclidean distance, Ward’s method), 26 T samples were separated into Cluster I (*n* = 8) and Cluster II (*n* = 18)

A total of 64, 027 probes, for which DNA methylation levels differed significantly between C and T samples, were identified (Welch’s t test, *P* < 0.05 after Bonferroni correction, Δβ_T−C_ value of more than 0.2 or less than − 0.2), indicating that DNA methylation alterations are associated with NASH-related hepatocarcinogenesis. To examine the DNA methylation profiles during multistage NASH-related hepatocarcinogenesis, principal component analysis of C, N and T samples was performed using the 64,027 probes (Fig. 1a). Since N samples were obtained from non-cancerous liver tissue that had already become the origin of NASH-related HCCs, such samples were considered to be at the precancerous stages. Such precancerous N samples showed distinct DNA methylation profiles that clearly differed from those of normal control C samples (Fig. 1a). Moreover, T samples themselves were scattered over a wider area, indicating heterogeneity of the DNA methylation profiles of such samples (Fig. 1a).

### Epigenetic clustering of NASH-related HCCs

**Fig. 2:**
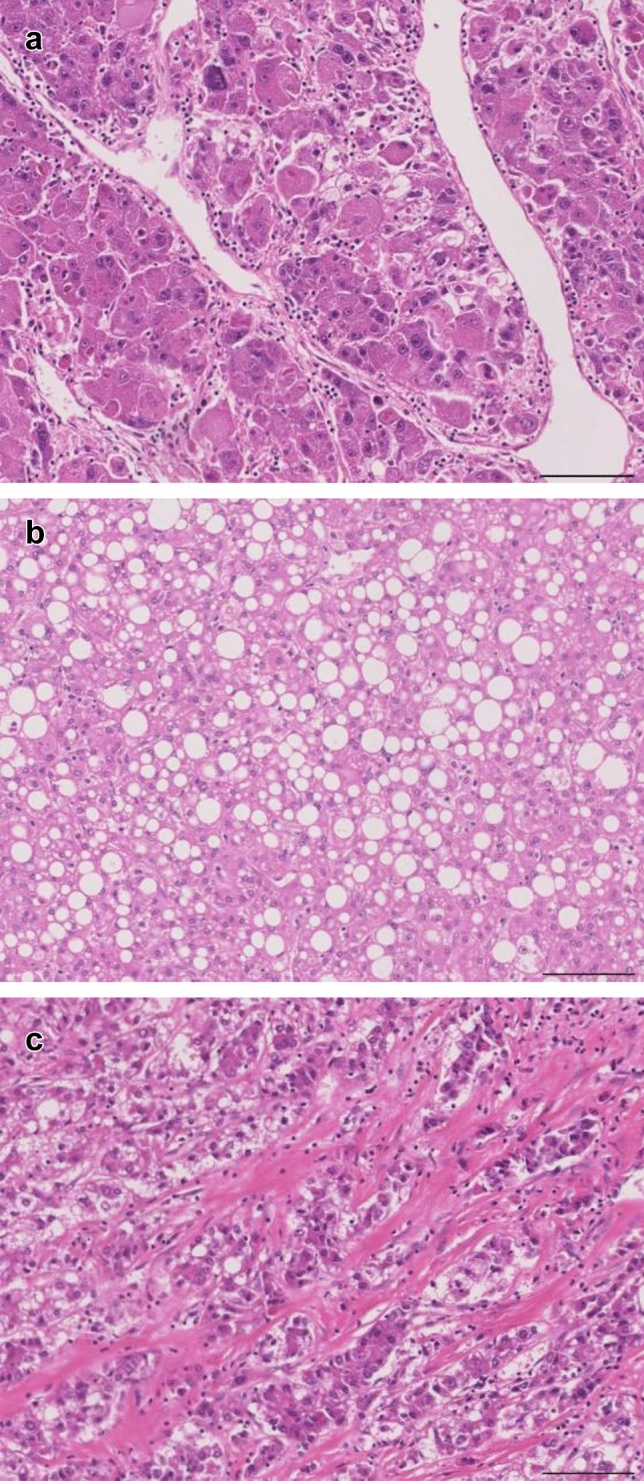
Representative photos of poorly differentiated hepatocellular carcinoma (HCC) (**a**), HCC with tumor cell steatosis (steatotic HCC) (**b**), and scirrhous HCC (**c**). Hematoxylin and eosin staining. Original magnification × 20

To further investigate the heterogeneity of T samples, we performed hierarchical clustering using the DNA methylation levels of the 64,027 probes (Euclidean distance, Ward’s method) (Fig. 1b): T samples were separated into Cluster I (*n* = 8) and Cluster II (*n* = 18). Correlations between such epigenetic clustering of NASH-related HCCs and clinicopathological parameters, such as age, sex and histopathological findings of N and T samples, were examined (Table 1). Poorly differentiated HCCs were significantly accumulated in Cluster I, whereas most of the well to moderately differentiated HCCs belonged to Cluster II (*P* = 0.020). The average percentage of tumor cells with lipid droplets (tumor steatosis) in Cluster II (15.2 ± 18.0%) was significantly higher than that in Cluster I (3.88 ± 5.25%) (*P* = 0.027). On the other hand, the average areal ratio of the scirrhous HCC component in Cluster I (41.1 ± 42.6%) was significantly higher than that in Cluster II (0.66 ± 1.57%) (*P* = 0.040). Representative photos of poorly differentiated HCC, HCC with tumor cell steatosis (steatotic HCC) and scirrhous HCC are shown in Fig. 2.

**Table 1:** Correlations between epigenetic clustering and clinicopathological parameters of patients with non-alcoholic steatohepatitis (NASH)-related hepatocellular carcinomas (HCCs)

Clinicopathological parameters	Number of samples or mean ± standard deviation		*P*^f^
	Cluster I (*n* = 8)	Cluster II (*n* = 18)	
Patients			
Age	70.6 ± 11.4	71.3 ± 4.25	0.800^g^
Sex			
Male	6	18	0.086^h^
Female	2	0	
Pathological findings of non-cancerous liver tissue			
Steatosis (%)^a^			
Less than 33	4	13	0.382^h^
33 or more	4	5	
Lobular inflammation (foci/200 × field)^a^			
Less than 2	6	15	0.628^h^
2 or more	2	3	
Ballooning^a^			
Non or few	7	16	1.000^h^
Many	1	2	
Fibrosis^b^			
1 or 2	5	11	1.000^h^
3 or 4	3	7	
NASH stage^b^			
1 or 2	8	16	1.000^h^
3	0	2	
Pathological findings of HCCs			
Tumor size (largest diameter) (mm)			
Less than 50	4	12	0.352^h^
50 or more	4	6	
Differentiation^c^			
Well to moderate	4	17	0.020^h^
Poor	4	1	
Tumor cell steatosis (%)^d^	3.88 ± 5.25	15.2 ± 18.0	0.027^g^
Scirrhous component (%)^d^	41.1 ± 42.6	0.66 ± 1.57	0.040^g^
Portal vein involvement			
Negative	5	11	0.401^h^
Positive	3	7	
Bile duct involvement			
Negative	7	18	0.308^h^
Positive	1	0	
Intrahepatic metastasis			
Negative	0	4	0.277^h^
Positive	8	14	
Pathological stage^e^			
T1a or T1b	4	7	0.683^h^
T2 to T4	4	11	

^a^Microscopic features (steatosis, lobular inflammation, ballooning and fibrosis) were evaluated according to the non-alcoholic fatty liver disease (NAFLD) activity score (NAS) (Brunt et al. 2011)

^b^NASH stage was defined according to the Brunt classification (Brunt et al. 2011)

^c^Histological differentiation of HCCs was in accordance with the World Health Organization classification (Torbenson et al. 2019)

^d^Scirrhous component and tumor cell steatosis were evaluated as described in the materials and methods section

^e^Pathological stage was defined according to the Union for International Cancer Control Tumor-Node-Metastasis classification (Brierley et al. 2017)

^f^*P* values of less than 0.05 are underlined

^g^Welch’s *t* test

^h^Fisher’s exact test

### Identification of genes for which DNA methylation status was associated with epigenetic clustering of NASH-related HCCs

Since epigenetic clustering was significantly correlated with the histological diversity of NASH-related HCCs, i.e. poorer tumor differentiation, tumor steatosis and development of the scirrhous HCC component, we identified the 140,241 probes showing significant differences in DNA methylation levels between Clusters I (*n* = 8) and Cluster II (*n* = 16) (Welch’s *t* test, *P* < 0.05). Among the 140,241 probes, 63,523 were located within CpG islands, island shores (2000-bp regions adjacent to a CpG island) or island shelves (2000-bp regions adjacent to an island shore) based on the University of California, Santa Cruz (UCSC) genome browser (https://genome.ucsc.edu/). Among the 63,523 probes, 22,512 are annotated with TSS1500 (from 200 bp upstream of the transcription start site [TSS] to 1500 bp upstream of it), TSS200 (from TSS to 200 bp upstream of it), the 5′ untranslated region (UTR) or the 1st exon based on the RefSeq database (http://www.ncbi.nlm.nih.gov/refseq/).

To focus on DNA methylation alterations possibly resulting in changes of expression, using datasets for 412 samples of non-cancerous and cancerous liver tissue deposited in the Cancer Genome Atlas (TCGA) database (https://www.cancer.gov/about-nci/organization/ccg/research/structural-genomics/tcga), correlations between DNA methylation and mRNA expression levels for the 22,512 probe CpG sites were examined. Among the 22,512 probes, 3888 designed for 1890 genes showed a significant inverse correlation between the levels of DNA methylation and mRNA expression (*r* < − 0.2, *P* < 0.05) (Supplementary Table 3). Inverse correlations between DNA methylation and mRNA expression levels on representative genes are shown in Supplementary Fig. 1.

The 3888 probes were then subjected to MetaCore pathway analysis. Genes showing significant differences in DNA methylation levels between the two clusters were significantly accumulated in 353 signaling pathways (FDR < 0.05). After elimination of pathways solely participating in diseases other than cancer or organs other than the liver, Table 2 summarizes the top 25 signaling pathways. Among them, representative pathways clearly participate in cell adhesion and cytoskeletal remodeling, such as “Cytoskeleton remodeling_Regulation of actin cytoskeleton organization by the kinase effectors of Rho GTPases (FDR = 5.49 × 10^−6^)” and “Cell adhesion_Histamine H1 receptor signaling in the interruption of cell barrier integrity (FDR = 1.62 × 10^−4^)”, cell proliferation and death, such as “Development_Positive regulation of STK3/4 (Hippo) pathway and negative regulation of YAP/TAZ function (FDR = 8.64 × 10^−5^)” and “Signal transduction_FGFR3 signaling (FDR = 3.97 × 10^−4^)” and epigenetic regulation, such as “CHDI_Correlations from Discovery data_Causal network (FDR = 2.09 × 10^−4^)” and “Development_H3K27 demethylases in differentiation of stem cells (FDR = 3.97 × 10^−4^)”. Representative pathway maps are shown schematically in Supplementary Fig. 2. The top 25 pathways in Table 2 consist of 149 genes in total. Among these 149 genes, taking into consideration their literature-based implications in the process of carcinogenesis, we further focused on the *SPHK1*, *INHBA*, *LTB* and *PDE3B* genes.

**Table 2:** Top 25 statistically significant GO pathway maps revealed by MetaCore software analysis using the 3888 probes designed for the 1890 genes showing significant differences in DNA methylation levels between Cluster I and Cluster II

Pathway maps	False discovery rate	Involved genes showing significant differences between Clusters I and II
Cytoskeleton remodeling_Regulation of actin cytoskeleton organization by the kinase effectors of Rho GTPases	5.49 × 10^−6^	*ARHGEF7, TLN1, ROCK2, RHOU, CDC42BPA, RHOG, RHOU, RDX, MYL9, ARPC1B, RHOC, CTTN, PPP1R14A, LIMK1, RHOB, MYH14, LIMK1, CFL2, CDC42BPA*
Development_positive regulation of STK3/4 (Hippo) pathway and negative regulation of YAP/TAZ function	8.64 × 10^−5^	*CDH1, CRB3, RASSF1, PRKACA, ADCY5, BTRC, ITCH, CCDC85C, FAS, SFN, RASSF5, CSNK1E, BTRC, PRKAR1B, RAF1, ADRB2, AXIN2, PRKACA, AMOTL2, TJP2*
Cell adhesion_Histamine H1 receptor signaling in the interruption of cell barrier integrity	1.62 × 10^−4^	*TLN1, BCAR1, CDH1, ROCK2, CALM2, PLCB1, PRKCD, MYL9, OCLN, GNB4, PPP1R14A, LIMK1, SRC, CFL2, ITPR3*
CHDI_correlations from discovery data_causal network	2.09 × 10^−4^	*TSC2, SHC1, CALM2, PLCB1, PLCG1, GNAI1, HRAS, GNB4, RAF1, SMAD3, AKT1, CAPN1, PIK3R5, SRC, DVL2, AXIN2, FZD8, ITPR3, TGFBR1*
NRF2 regulation of oxidative stress response	2.09 × 10^−4^	*GSTP1, UGT1A1, MAFG, PRKCH, SOD1, KEAP1, RARA, MAFK, RAF1, ENC1, AKT1, GCLC, PRDX1, TXNRD1, NFE2L2, NQO1*
Signal transduction_Adenosine A1 receptor signaling pathway	2.75 × 10^−4^	*NFKBIB, CALM2, PRKCH, PLCB1, GNAI1, HRAS, ADCY5, ADA, GNB4, PRKAR1B, RAF1, AKT1, SRC, PRKACA, STAT1, ITPR3*
Signal transduction_S1P2 receptor inhibitory signaling	2.75 × 10^−4^	*ROCK2, PLCB1, GNAI1, HRAS, ADCY5, MYL9, ARHGEF1, GNB4, PRKAR1B, RAF1, AKT1, RHOC, S1PR2, GNAI2, PRKACA, ITPR3*
Chemotaxis_CXCR3-A signaling	2.75 × 10^−4^	*SHC1, LCK, CALM2, GRK6, PLCG1, HRAS, OCLN, GNB4, STAT5A, RAF1, AKT1, CAPN1, PIK3R5, SRC, GNAI2, CTSB, STAT1, ITPR3*
Transport_induction of macropinocytosis	2.75 × 10^−4^	*RHOG, ELMO2, PDGFB, PLEKHG6, PRKCH, CORO1A, PLCG1, GNAI1, HRAS, YWHAZ, PLCG1, GNB4, IFNGR1, ALS2, DGKZ, AKT1, SRC, SH3YL1, EZR*
Signal transduction_S1P3 receptor signaling	3.06 × 10^−4^	*SPHK1, BCAR1, SHC1, ROCK2, CALM2, PLCB1, PRKCD, GNAI1, HRAS, ADCY5, ARHGEF1, ACTA2, GNB4, GATA4, VEGFA, RAF1, SMAD3, AKT1, LIMK1, SRC, GNAI2, CFL2, ITPR3, TGFBR1*
Development_H3K27 demethylases in differentiation of stem cells	3.97 × 10^−4^	*HOXA13, H3C7, HOXC4, NODAL, BMP4, BMI1, RARA, HOXA3, SMAD3, SMAD2, HOXA11, BMP2, PAX6*
Signal transduction_FGFR3 signaling	3.97 × 10^−4^	*CCND2, BCAR1, SHC1, PRKCH, TDGF1, PLCG1, STAT5A, HRAS, FGF2, RAF1, AKT1, IRF1, GCLC, NFE2L2, SRC, FGF21, STAT1, ITPR3*
Development_Role of growth factors in the maintenance of embryonic stem cell pluripotency	4.40 × 10^−4^	*ROCK2, ERBB2, FGFR2, INHBA, IRS1, NODAL, IGF2, HRAS, ERBB3, FGF2, RAF1, SMAD3, AKT1, SMAD2, TGFBR1*
Signal transduction_beta-adrenergic receptors signaling via MAPK cascade	4.80 × 10^−4^	*SHC1, CALM2, PRKCH, PLCB1, GNAI1, HRAS, GNB4, GATA4, RAF1, ADRB2, AKT1, SRC, PIK3R5, SRC, PRKACA, ITPR3*
CHDI_DEGs from replication data_causal network	5.05 × 10^−4^	*NFKBIB, SHC1, CALM2, PRKCH, PRKCD, PPP1CC, HRAS, LRP5, LTB, CSNK1E, CTBP2, RAF1, AKT1, NEFM, DVL2, AXIN2, FZD8, PLCB1, ITPR3*
Signal transduction_FGFR4 signaling	5.83 × 10^−4^	*BCAR1, NFKBIB, SHC1, PRKCH, CYP8B1, FGFR4, LDLR, PLCG1, SCAP, HRAS, FGF2, RAF1, AKT1, MVK, SRC, DHCR7, DHCR24, FGF21, STAT1*
Nociception_nociceptin receptor signaling	6.91 × 10^−4^	*ROCK2, PRKCH, PLCB1, GNAI1, HRAS, ADCY5, OPRL1, GNB4, PRKAR1B, GNAZ, RAF1, LIMK1, SRC, ACTA2, GNA14, PRKACA, CFL2, ITPR3*
Apoptosis and survival_HTR1A signaling	8.15 × 10^−4^	*NFKBIB, SHC1, CALM2, GNAI1, HRAS, ADCY5, GNB4, PPP2R3A, PRKAR1B, RAF1, AKT1, PIK3R5, SRC, PRKACA*
Immune response_CRTH2 signaling in Th2 cells	8.37 × 10^−4^	*CALM2, PRKCH, PLCB1, ITGB2, GNAI1, ADCY5, IL13, SFN, GNB4, CD40, AKT1, PIK3R5, ITPR3*
Cell adhesion_gap junctions	8.82 × 10^−4^	*CDH1, TUBA8, CALM2, PRKACA, OCLN, GJC1, ACTA2, TJP2, EZR*
Signal transduction_PKA signaling	8.96 × 10^−4^	*PDE4A, AKAP12, PDE3B, PALM2AKAP2, PRKACA, GNAI1, ADCY5, KDELR3, NFKBIB, PPP2R3A, PRKAR1B, SMAD3, PKIG*
Development_prolactin receptor signaling	9.43 × 10^−4^	*STAT5A, SHC1, NR3C1, PRKCD, IRS1, CEBPB, HRAS, PLCG1, STAT5A, RAF1, AKT1, IRF1, SRC, NEK3, STAT1*
Development_G-protein-mediated regulation of MAPK-ERK signaling	1.15 × 10^−3^	*SHC1, CALM2, PLCB1, GNAI1, HRAS, GNB4, MRAS, PRKAR1B, RAF1, SRC, SYNGAP1, PRKACA, ITPR3*
G-protein signaling_G-protein beta/gamma signaling cascades	1.16 × 10^−3^	*SHC1, ADCY5, HRAS, PLCG1, GNB4, PRKAR1B, RAF1, AKT1, PIK3R5, SRC, PRKACA*
G-protein signaling_Rac1 activation	1.26 × 10^−3^	*ARHGEF7, BCAR1, ELMO2, LCK, ERBB2, RHOG, CORO1A, FARP1, HRAS, GNB4, RHOU, SEMA6A, ALS2, PIK3R5, SRC, FARP2, PRKACA*

After elimination of pathways solely participating in diseases other than cancer or organs other than the liver, the top 25 pathways are summarized in this table.

### DNA methylation and mRNA expression levels of tumor-related genes in the present cohort

**Fig. 3:**
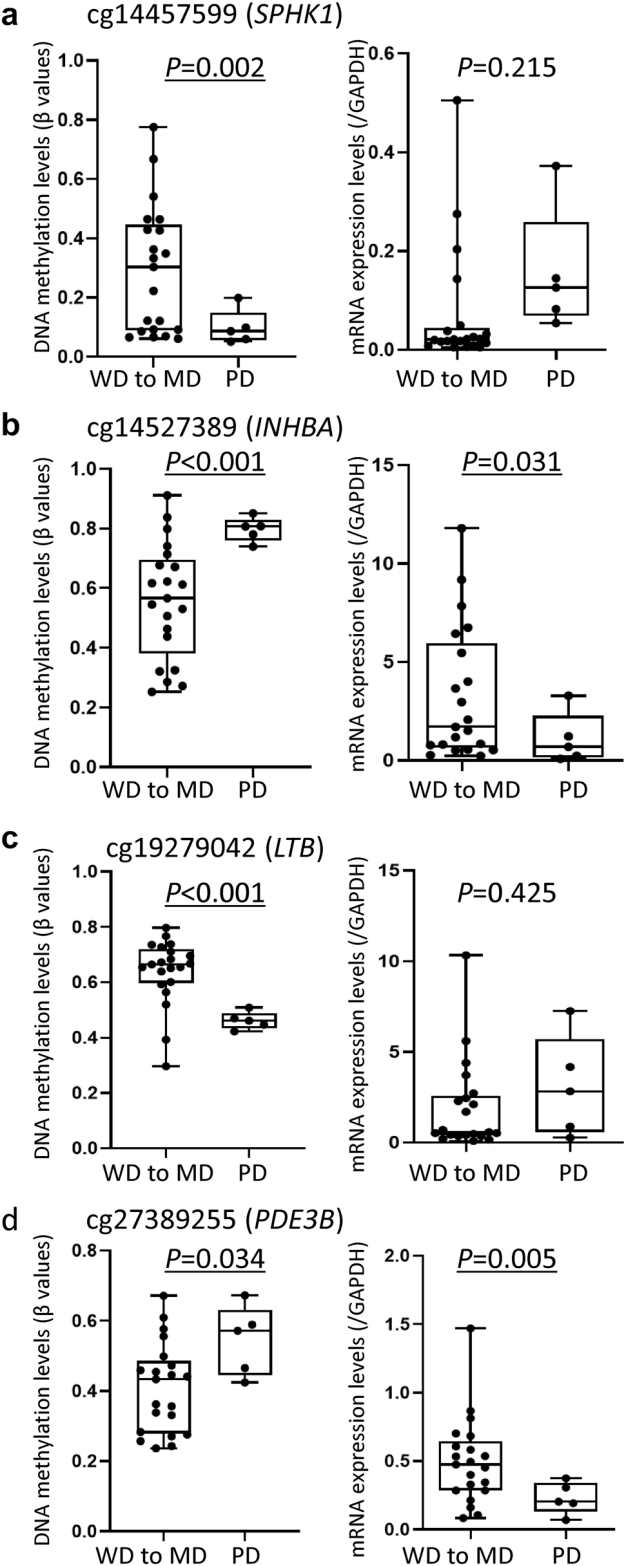
DNA methylation levels based on the Infinium assay and mRNA expression levels based on quantitative reverse transcription-PCR analysis of the *SPHK1* (**a**), *INHBA* (**b**), *LTB* (**c**) and *PDE3B* (**d**) genes in 26 samples of non-alcoholic steatohepatitis (NASH)-related hepatocellular carcinoma (HCC). *P*-values of less than 0.05 are underlined. Infinium probe ID is shown at the top of each panel. **a** DNA hypomethylation and overexpression of *SPHK1* are evident in poorly differentiated HCCs (PD) (*n* = 5) in comparison with well to moderately differentiated HCCs (WD to MD) (*n* = 21)*.* **b** DNA hypermethylation and reduced expression of *INHBA* are observed in PD. **c** DNA hypomethylation and overexpression of *LTB* are observed in PD. **d** DNA hypermethylation and reduced expression of *PDE3B* are observed in PD

Levels of mRNA expression for the *SPHK1*, *INHBA*, *LTB* and *PDE3B* genes were examined in the present liver tissue samples using real-time quantitative RT-PCR analysis. To confirm that DNA methylation profiles participate in histological diversity characterizing the epigenetic clustering, correlations between DNA methylation levels based on the Infinium assay and mRNA expression levels based on real-time quantitative RT-PCR analysis of the focused genes on one hand and poorer tumor differentiation were examined (Fig. 3). DNA hypermethylation of *INHBA* and *PDE3B* resulting in their reduced expression was observed in poorly differentiated HCCs (*n* = 5) relative to well to moderately differentiated HCCs (*n* = 21)*.* On the other hand, DNA hypomethylation of *SPHK1* and *LTB* resulting in their overexpression was observed in poorly differentiated HCCs, although such overexpression did not reach a statistically significant level due to a few outliers. These data indicated that DNA methylation profiles participate in determining the histological diversity of HCCs, such as poorer differentiation, via alterations of gene expression.

### DNA methylation status-related transcriptional regulation of tumor-related genes

**Fig. 4:**
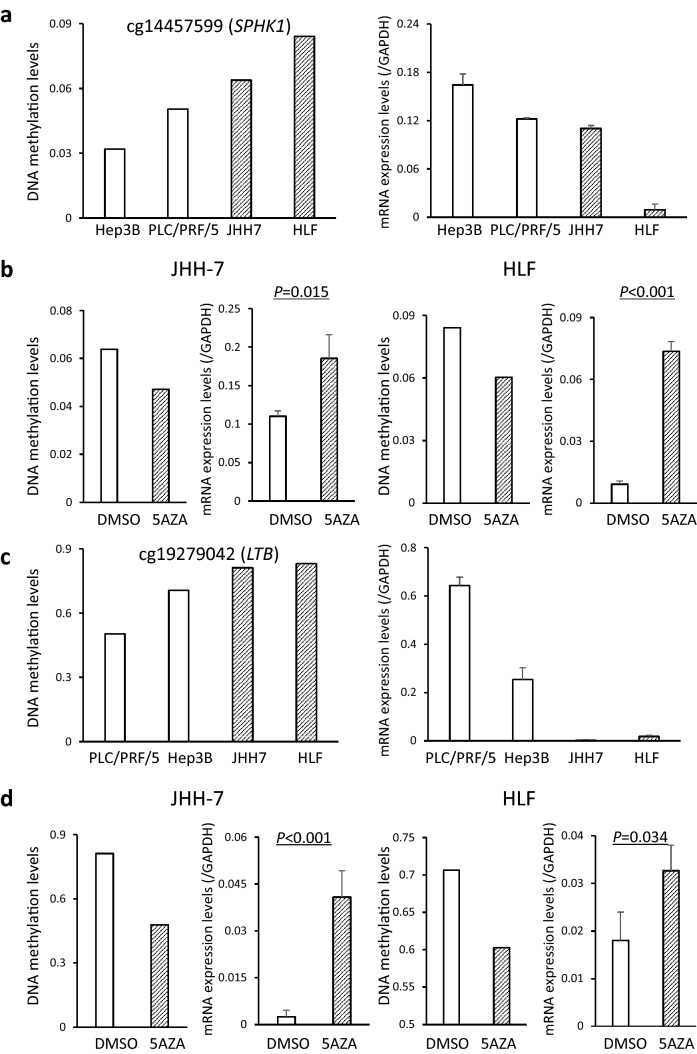
5-Aza-2′-deoxycytidine (5AZA) treatment of human hepatocellular carcinoma (HCC) cell lines. **a** DNA methylation levels based on the Infinium assay and mRNA expression levels based on quantitative reverse transcription-PCR analysis of the *SPHK1* gene in the human HCC cell lines, Hep3B, PLC/PRF/5, JHH-7 and HLF. Infinium probe ID is shown at the top of the panel. In the top two cell lines showing the highest levels of *SPHK1* DNA methylation, JHH-7 and HLF, the levels of mRNA expression were low. **b** In the JHH-7 and HLF cell lines, after 5AZA treatment, reduced DNA methylation levels and restored expression of the *SPHK1* gene were observed in comparison with dimethyl sulfoxide (DMSO)-treated controls. **c** DNA methylation levels and mRNA expression of the *LTB* gene in Hep3B, PLC/PRF/5, JHH-7 and HLF. In the top two cell lines showing the highest levels of *LTB* DNA methylation, JHH-7 and HLF, the levels of mRNA expression were low. **d** In the JHH-7 and HLF cell lines, after 5AZA treatment, reduced DNA methylation levels and restored expression of the *LTB* gene were observed

To further reveal details of DNA methylation status-related transcriptional regulation, we focused on the *SPHK1* and *LTB* genes. Based on the Infinium assay, the DNA methylation levels of the *SPHK1* gene in the human HCC cell lines, Hep3B, PLC/PRF/5, JHH-7 and HLF, are shown in Fig. 4a. In the top two cell lines showing the highest levels of DNA methylation for *SPHK1*, JHH-7 and HLF, the levels of mRNA expression were low (Fig. 4a). Similarly, in the top two cell lines showing the highest DNA methylation levels for *LTB*, JHH-7 and HLF, the levels of mRNA expression were also low (Fig. 4c). These cell lines were then subjected to 5-aza-dC treatment. This led to a marked reduction in the levels of DNA methylation and restoration of the expression levels of *SPHK1* and *LTB* mRNA (Fig. 4b, d, respectively), indicating that the mRNA expression levels of these genes are regulated by DNA methylation in HCC cells.

### Significance of the SPHK1 gene in proliferation, apoptosis and migration of HCC cells

**Fig. 5:**
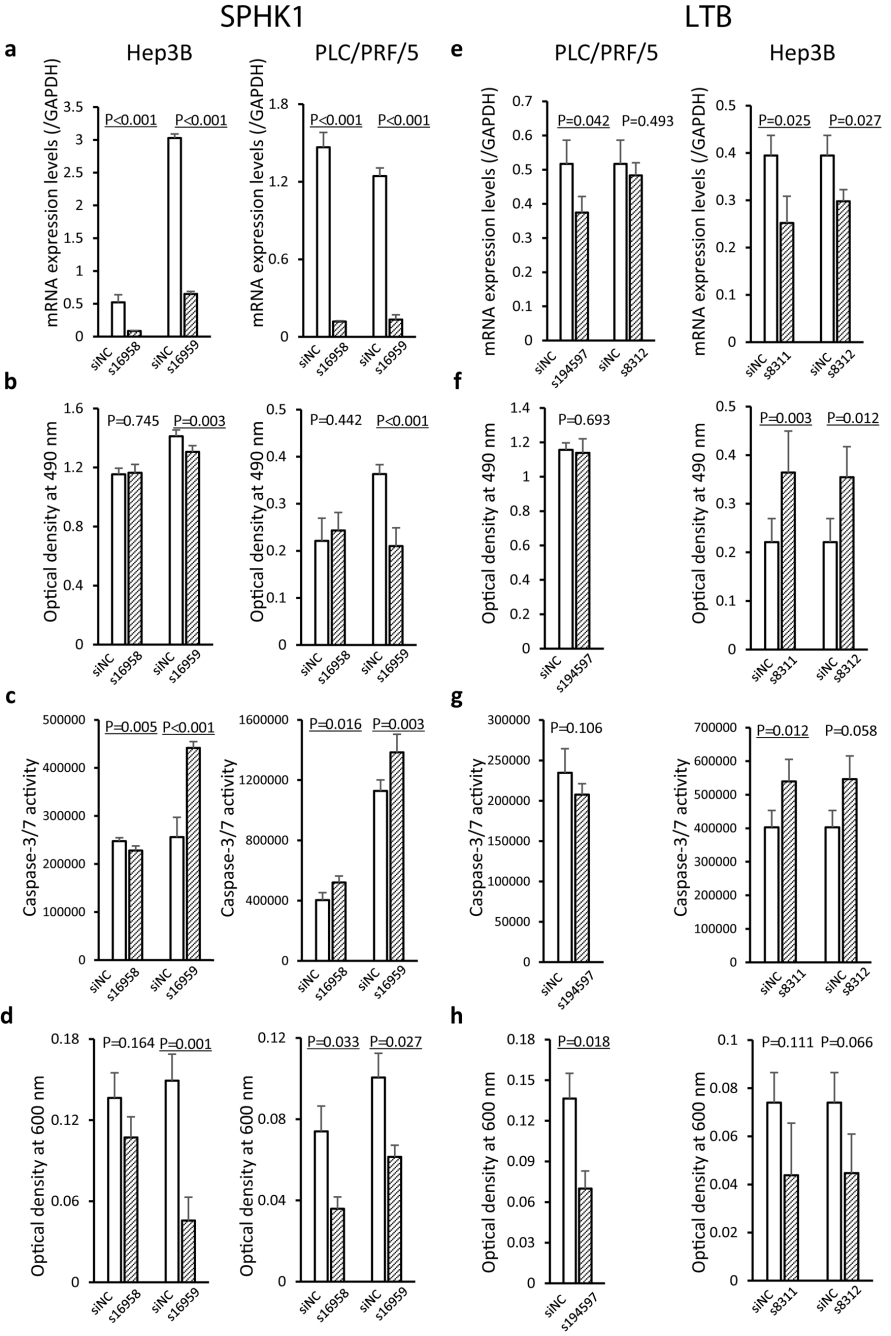
Knockdown of the *SPHK1* and *LTB* genes using small interfering RNA (siRNA) in human hepatocellular carcinoma (HCC) cell lines. **a** In the top two cell lines showing the highest levels of mRNA expression of the *SPHK1* gene in Fig. 4, Hep3B and PLC/PRF/5, after transfection with *SPHK1*-specific siRNA, s16958 and s16959, reduction of *SPHK1* expression was confirmed in both cell lines by quantitative real-time RT-PCR. **b** The results of the 3-(4,5-dimethylthiazol-2-yl)-5-(3-carboxymethoxyphenyl)-2-(4-sulfophenyl)-2H-tetrazolium (MTS) cell viability assay in s16958- or s16959-transfected Hep3B and PLC/PRF/5 cells. *P*-values of less than 0.05 are underlined. **c** The results of apoptosis assay in s16958- or s16959-transfected cells. **d** The results of the cell migration assay for s16958- or s16959-transfected cells. **e** In the top two cell lines showing the highest levels of mRNA expression for the *LTB* gene in Fig. 4, PLC/PRF/5 and Hep3B, after transfection with *LTB*-specific siRNA, s194597, s8311 and s8312, reduction of *LTB* expression was confirmed in both cell lines. **f** The results of the MTS cell viability assay for s194597-, s8311- and s8312-transfected cells. **g** The results of the apoptosis assay for s194597-, s8311- and s8312-transfected cells. **h** The results of the cell migration assay for s194597-, s8311- and s8312-transfected cells

Knockdown of *SPHK1* using siRNA transfection was performed in the top two cell lines showing the highest mRNA expression levels, Hep3B and PLC/PRF/5. After transfection with the *SPHK1*-specific siRNAs, s16958 and s16959, marked reduction of *SPHK1* expression was confirmed in both cell lines by quantitative real-time RT-PCR (Fig. 5a). Decreased cell growth was observed in s16959-treated Hep3B and PLC/PRF/5 cells (Fig. 5b). Moreover, caspase-3/7 activities were increased (Fig. 5c) and cell migration ability was repressed (Fig. 5d) using all *SPHK1*-specific siRNAs in both Hep3B and PLC/PRF/5 cells.

### Significance of the LTB gene in proliferation, apoptosis and migration of HCC cells

Knockdown of *LTB* using siRNA transfection was performed in the top two cell lines showing the highest mRNA expression levels, PLC/PRF/5 and Hep3B. After transfection, reduction of *LTB* expression was confirmed in PLC/PRF/5 (s194597) and Hep3B (s8311 and s8312) by quantitative real-time RT-PCR (Fig. 5e). Although an increase of both cell growth and caspase-3/7 activities was observed in only Hep3B cells (Fig. 5f, g), cell migration ability was clearly repressed by knockdown of *LTB* in both PLC/PRF/5 and Hep3B cells (Fig. 5h).

## Discussion

The present PCA based on genome-wide DNA methylation analysis clearly revealed that distinct DNA methylation profiles had already been established in N samples, differing from such profiles in C samples (Fig. 1a), indicating that DNA methylation alterations may participate in multistage hepatocarcinogenesis even from the precancerous NASH stage. Participation of DNA methylation alterations even from the precancerous stage is consistent with our previous findings in not only NASH-related and viral hepatitis-related multistage hepatocarcinogenesis but also tissue specimens at the precancerous stages in kidney (Arai et al. 2012), urothelium (Nishiyama et al. 2010), stomach (Yamanoi et al. 2015) and lung (Sato et al. 2014) exposed to various carcinogens.

Moreover, the PCA further indicated heterogeneity of the DNA methylation profiles of T samples (Fig. 1a). This led us to suspect that DNA methylation alterations may underlie the clinicopathological diversity of NASH-related HCCs. We then performed hierarchical clustering of T samples using their DNA methylation profiles. Epigenetic clustering, i.e. Cluster I vs Cluster II, was significantly correlated with histological features, indicating that DNA methylation profiles may participate in determining the histological diversity of NASH-related HCCs, characterized by features such as poorer differentiation, tumor steatosis and development of a scirrhous HCC component.

Then we focused on differences in DNA methylation profiles between the two epigenetic clusters and identified 3888 CpG sites located in chromosomal regions that are important for transcriptional regulation, e.g., CpG islands, island shores and shelves around the TSSs (Bird 2002) of the 1890 genes, where inverse correlations between DNA methylation and mRNA expression were confirmed using data deposited in the TCGA database. Genes for which differences in DNA methylation levels between the two clusters would potentially result in expression differences were accumulated in molecular pathways participating in cell adhesion and cytoskeletal remodeling, cell proliferation and death, and epigenetic regulation. It is quite feasible that functional disturbance of such molecular pathways would determine the clinicopathological diversity of cancers.

Among the 149 genes involved in molecular pathways possibly determining the clinicopathological diversity of NASH-related HCCs listed in Table 2, taking into consideration their literature-based implications in the process of carcinogenesis, we further focused on the *SPHK1*, *INHBA*, *LTB* and *PDE3B* genes. SPHK1 is a lipid kinase catalyzing the formation of sphingosine-1-phosphate (S1P) from the precursor sphingolipid. S1P is a vital lipid second messenger involved in diverse cellular processes including cell proliferation (Khoei et al 2021). Moreover, overexpression of *SPHK1* has been reported in cancers of multiple organs, such as the lung (Ma et al. 2021) and pancreas (Yu et al. 2021a). With respect to hepatocarcinogenesis, it has been reported that *SPHK1* becomes the target of non-coding RNAs involved in angiogenesis in HCCs (Lu et al. 2015) and that S1P export via the ABCC1 transporter participates in HCC progression (Satyananda et al. 2021). Although tissue-specific DNA methylation of the *SPHK1* gene is known (Imamura et al. 2001), abnormalities in its DNA methylation during carcinogenesis have not yet been elucidated.

INHBA is a member of the transforming growth factor (TGF)-β superfamily. Overexpression of *INHBA* has been reported in several cancers, such as those of the colon (Guo and Liu 2021) and stomach (Zhang et al. 2019). In breast cancer, INHBA reportedly induces epithelial-mesenchymal transition by activating the TGF-β signaling pathway (Yu et al. 2021b). Although DNA hypomethylation resulting in overexpression of *INHBA* has been reported in urothelial (Kao et al. 2022) and gastric (Zhang et al. 2019) carcinomas, the significance of INHBA in HCCs has not yet been clarified.

Lymphotoxin was originally purified and characterized as a TNF-like soluble molecule produced by lymphocytes. Type II membrane protein LTB anchors LTA (lymphotoxin-alpha) to the cell surface through heterotrimer formation and participates in normal development of lymphoid tissue (Korneev et al. 2017). With respect to carcinogenesis, mice with knockout of LTA have been reported to show enhanced tumor growth, suggesting a possible tumor-suppressive role of LTA (Ito et al. 1999). On the other hand, although HCV infection reportedly induces *LTB* expression in human hepatocytes (Haybaeck et al. 2009), the significance of *LTB* in human cancer cells, and not in lymphocytes infiltrating the cancer stroma, has remained unclear in both the liver and other organs. Moreover, DNA methylation alterations of the *LTB* gene in human cancers have not been reported previously.

PDE3B is a cyclic nucleotide phosphodiesterase that regulates various physiological processes such as cell proliferation by controlling the degradation of cyclic AMP and cyclic GMP (Beavo 1995). PDE3B plays an important role in the energy homeostasis of adipocytes and hepatocytes, as well as in insulin signaling (Beavo 1995). With respect to cancers, overexpression of PDE3B and lower cyclic AMP levels have been observed in colorectal cancers (McEwan et al. 2007) and gastrointestinal stromal tumor (Pulkka et al. 2019). Moreover, targeting of *PDE3B* enhances cisplatin sensitivity in human cancer cells (Uzawa et al. 2013). However, the significance of *PDE3*B during hepatocarcinogenesis and regulation of *PDE3B* through DNA methylation have not yet been clarified.

In our present set of tissue samples, differences in DNA methylation levels resulting in differences in the mRNA expression levels of the *SPHK1*, *INHBA*, *LTB* and *PDE3B* genes were correlated with poorer tumor differentiation (Fig. 3). These findings again confirmed that the mRNA expression levels of these tumor-related genes are regulated by DNA methylation and that their DNA methylation levels determine the clinicopathological diversity of HCCs. On the other hand, in viral hepatitis-related HCCs from our other cohort with HBV or HCV infection, there was no evident correlation between the DNA methylation levels of the *SPHK1*, *INHBA*, *LTB* and *PDE3B* genes and poorer tumor differentiation (Supplementary Fig. 3) (Kuramoto et al. 2017), indicating that participation of these genes in tumor differentiation may occur in a NASH-related HCC-specific manner. Moreover, with respect to the *SPHK1* and *LTB* genes on which we focused further, 5-aza-dC treatment clearly revealed epigenetic regulation of their expression in HCC cells.

Knockdown experiments have revealed that SPHK1 promotes cell proliferation, represses apoptosis and enhances the migration of HCC cells. DNA hypomethylation resulting in overexpression of the *SPHK1* gene in poorly differentiated HCCs, in comparison with well to moderately differentiated HCCs, may underlie the aggressive phenotype of poorly differentiated HCCs characterized by enhanced cell proliferation, repressed apoptosis and enhanced migration ability. Although the effects of cell growth and caspase-3/7 activities were not constant, indicating that the functions of LTB differ among cell lines, knockdown experiments have shown that LTB enhances the migration of HCC cells. DNA hypomethylation resulting in overexpression of the *LTB* gene in poorly differentiated HCCs may underlie the aggressive phenotype of poorly differentiated HCCs characterized by enhanced migration ability.

In summary, the DNA methylation profiles of NASH-related HCCs may determine the clinicopathological heterogeneity of tumors through alterations in the expression of tumor-related genes, such as *SPHK1*, *INHBA*, *LTB* and *PDE3B*. Participation of the DNA methylation status of these genes in determining the tumor phenotype may be specific to NASH-related HCCs. Genome-wide DNA methylation analysis is a powerful tool for identifying the tumor-related genes that underlie tumor heterogeneity.

## Supplementary Information

Below is the link to the electronic supplementary material.

Supplementary file1 (PDF 4790 KB)

## Abbreviations

5-aza-dC: 5-Aza-2′-deoxycytidine
C: Control liver tissue
Ct: Threshold cycle
FDR: False discovery rate
GEO: Gene expression omnibus
HBV: Hepatitis B virus
HCC: Hepatocellular carcinoma
HCV: Hepatitis C virus
JCRB: Japanese collection of research bioresources
MTS: 3-(4,5-Dimethylthiazol-2-yl)-5-(3-carboxymethoxyphenyl)-2-(4-sulfophenyl)-2H-tetrazolium
N: Non-cancerous liver tissue
NASH: Non-alcoholic steatohepatitis
PCA: Principal component analysis
RT: Reverse transcription
siRNA: Small interfering RNA
T: Tumorous tissue
TCGA: The cancer genome atlas
TGF: Transforming growth factor
TSS: Transcription start site
UCSC: The University of California, Santa Cruz
UTR: Untranslated region
