# Supplementary material for: DNA methylation status of the SPHK1 and LTB genes underlies the clinicopathological diversity of non-alcoholic steatohepatitis-related hepatocellular carcinomas
Source: J Cancer Res Clin Oncol. 2022 Nov 8;149(8):5109–25. doi: 10.1007/s00432-022-04445-9 (PMC10349775; doi:10.1007/s00432-022-04445-9)
Supplement: Supplementary file 1 — Supplementary file1 (PDF 4790 KB) [file 432_2022_4445_MOESM1_ESM.pdf]

Supplementary Table 1 Clinicopathological characteristics of the patients from whom tissue specimens were obtained.

Clinicopathological parameters		Number of samples or mean±standard deviation: Control patients (C, n=36)	Number of samples or mean±standard deviation: Hepatocellular carcinoma (HCC) patients (N and T, n=26)
Patients			
Age		61.5±7.94	71.4±7.24
Sex	Male	26	24
	Female	10	2
Pathological findings of N samples			
Steatosis (%)[a]	Less than 5		0
	5 or more but less than 33		17
	33 or more but less than 66		8
	66 or more		1
Lobular inflammation (foci/200 x field)[a]	Lesss than 2		21
	2 or more but less than 4		5
	4 or more		0
Ballooning[a]	None		0
	Few		23
	Many		3
Fibrosis[b]	0		0
	1		6
	2		10
	3		6
	4		4
Non-alcoholic steatohepatitis (NASH) stage[b]	1		20
	2		4
	3		2
Patholgical findings of HCCs			
Tumor size (largest diameter) (mm)	Less than 50		16
	50 or more		10
Differentiation[c]	Well		4
	Moderate		17
	Poor		5
Tumor cell steatosis (%)[d]			11.7±16.1
Scirrhous component (%)[d]			13.1±30.2
Portal vein involvement	Positive		14
	Negative		12
Bile duct involvement	Positive		1
	Negative		25
Intrahepatic metastasis	Positive		0
	Negative		26
Pathological stage[e]	T1a		2
	T1b		9
	T2		13
	T3		1
	T4		1

C, normal control liver tissue; N, non-cancerous liver tissue obtained by partial hepatectomy from HCC patients; T, corresponding cancerous tissue. [a]Microscopic features (steatosis, lobular inflammation, ballooning and fibrosis) were evaluated according to the non-alcoholic fatty liver disease (NAFLD) activity score (NAS) (Brunt et al .2011). [b]NASH stage was defined according to the Brunt classification (Brunt et al. 2011). [c]Histological differentiation of HCCs was in accordance with the World Health Organization classification (Torbenson et al. 2019). [d]Tumor cell steatosis and the scirrhous component were evaluated as described in the Methods section. [e]Pathological stage was defined according to the Union for International Cancer Control Tumor-Node-Metastasis classification (Brierley et al. 2017).

Supplementary Table 2 List of primers used for the quantitative reverse transcription-PCR reaction.

Gene	Primer	Sequence
GAPDH	Forward	5`tgtgggcaaggtcatccct3`
	Reverse	5`gccctccgacgcctgcttca3`
SPHK1	Forward	5`aggctgaaatctccttcacgc3`
	Reverse	5`gtctccagacatgaccaccag3`
INHBA	Forward	5`gaacagccaggaagacgctgc3`
	Reverse	5`accactgacaggtcactgccttc3`
LTB	Forward	5`cacaggcccagcaaggactg3`
	Reverse	5`gctgagatctgtttctggctcctc3`
PDE3B	Forward	5`atgtaggtctcagaagagctggtg3`
	Reverse	5`gggtgatcgattagttagagagccag3`

Supplementary Table 3 The 3,888 probes designed for the 1,890 genes showing significant differences in DNA methylation levels between Cluster I and Cluster II (*P* value <0.05 by Welch's t test and $\Delta\beta_{I-II}$>0.1 or <-0.1).

a The 1,795 probes showing DNA hypermethylation in Cluster I in comparison with Cluster II ($\Delta\beta_{I-II}$>0.1).

ProbeID[a]	Chromosome	Position[b]	Gene symbol	Annotation[c]	CpG type[d]	DNA methylatio levels (β value)					
						Cluster I (mean±SD)	Cluster II (mean±SD)	$\Delta\beta_{I-II}$	Welch's T-test	Inverse correlation with mRNA expression levels[e]	
									P	*P*	r
cg17415355	11	1320010	*TOLLIP*	5'UTR/1st intron	S_Shelf	0.815±0.057	0.372±0.212	0.443	6.24E-08	4.98E-10	-0.300
cg24222440	11	118397413	*TTC36*	TSS1500	N_Shelf	0.814±0.054	0.381±0.179	0.433	3.38E-09	1.27E-33	-0.548
cg02376018	3	187453520	*BCL6*	5'UTR/1st intron	N_Shelf	0.809±0.096	0.381±0.175	0.429	4.35E-08	6.80E-14	-0.358
cg26245202	1	205602864	*ELK4*	TSS1500	S_Shore	0.802±0.053	0.39±0.266	0.413	3.84E-06	1.84E-12	-0.338
cg19028997	1	205603040	*ELK4*	TSS1500	S_Shore	0.808±0.047	0.403±0.291	0.405	1.62E-05	3.38E-11	-0.319
cg00807909	5	169659618	*C5orf58*	TSS1500	N_Shore	0.721±0.103	0.324±0.149	0.397	1.91E-07	1.69E-06	-0.233
cg16694837	14	99945655	*SETD3*	5'UTR/1st intron	N_Shore	0.61±0.12	0.213±0.085	0.397	6.10E-06	1.90E-08	-0.273
cg19931529	2	64072167	*UGP2*	5'UTR/1st intron	S_Shelf	0.719±0.125	0.329±0.15	0.390	3.41E-06	2.72E-41	-0.598
cg04657700	2	223535084	*MOGAT1*	TSS1500	N_Shore	0.696±0.089	0.308±0.142	0.388	3.45E-08	9.88E-24	-0.467
cg18520851	5	115149189	*CDO1*	5'UTR/1st intron	N_Shelf	0.69±0.13	0.311±0.168	0.379	8.38E-06	3.78E-25	-0.480
cg25611443	1	205603068	*ELK4*	TSS1500	S_Shore	0.791±0.059	0.412±0.268	0.379	1.35E-05	1.82E-11	-0.323
cg01808050	2	3521258	*ADI1*	5'UTR/1st exon	N_Shore	0.708±0.227	0.33±0.152	0.378	1.61E-03	1.51E-48	-0.638
cg05152432	1	1689887	*NADK*	1st exon	N_Shore	0.671±0.238	0.297±0.104	0.373	2.63E-03	3.34E-12	-0.334
cg01585794	8	27347372	*EPHX2*	TSS1500	N_Shore	0.608±0.116	0.242±0.124	0.366	3.31E-06	7.57E-21	-0.439
cg18760876	5	169659627	*C5orf58*	TSS1500	N_Shore	0.748±0.109	0.381±0.19	0.366	2.99E-06	9.42E-07	-0.239
cg16589271	11	82993272	*CCDC90B*	5'UTR	N_Shelf	0.691±0.107	0.327±0.094	0.364	2.54E-06	3.14E-05	-0.204
cg19045070	11	118397595	*TTC36*	TSS1500	N_Shelf	0.662±0.175	0.301±0.092	0.362	4.09E-04	1.41E-09	-0.293
cg18422423	3	51576922	*RAD54L2*	5'UTR	S_Shelf	0.67±0.184	0.312±0.12	0.359	5.42E-04	2.30E-08	-0.271
cg18811130	10	131268886	*MGMT*	5'UTR/1st intron	S_Shelf	0.47±0.137	0.115±0.042	0.355	1.24E-04	2.50E-11	-0.321
cg15781838	2	128422717	*LIMS2*	5'UTR/1st intron	S_Shore	0.592±0.159	0.238±0.109	0.354	1.91E-04	1.08E-11	-0.327
cg07111988	1	24971764	*SRRM1*	5'UTR/1st intron	S_Shore	0.612±0.18	0.264±0.11	0.347	6.05E-04	6.68E-07	-0.242
cg15095727	3	138311508	*CEP70*	5'UTR/1st intron	N_Shore	0.604±0.197	0.257±0.096	0.347	1.25E-03	3.22E-08	-0.269
cg21200986	7	75546790	*POR*	5'UTR/1st intron	S_Shelf	0.598±0.145	0.255±0.095	0.343	1.25E-04	3.26E-33	-0.545
cg03233464	5	139938009	*SRA1*	TSS1500	S_Shore	0.687±0.097	0.345±0.101	0.342	1.02E-06	3.06E-07	-0.249
cg07766743	8	27348001	*EPHX2*	TSS1500	N_Shore	0.634±0.13	0.292±0.111	0.342	3.58E-05	8.53E-19	-0.417
cg07521912	5	169660734	*C5orf58*	5'UTR/1st intron	S_Shore	0.667±0.118	0.325±0.117	0.342	1.03E-05	6.35E-08	-0.263
cg00768179	1	8083892	*ERRFI1*	5'UTR/1st intron	N_Shore	0.616±0.176	0.276±0.092	0.341	6.40E-04	6.02E-07	-0.243
cg27527503	4	108909664	*HADH*	TSS1500	N_Shore	0.644±0.203	0.305±0.114	0.339	1.62E-03	5.88E-17	-0.396
cg02059055	6	18390303	*RNF144B*	5'UTR/1st intron	S_Shelf	0.571±0.109	0.233±0.137	0.338	3.53E-06	1.77E-11	-0.323
cg11903188	2	239195602	*PER2*	5'UTR/1st intron	N_Shore	0.592±0.138	0.254±0.106	0.338	7.62E-05	1.18E-06	-0.237
cg04676799	5	115152938	*CDO1*	TSS1500	S_Shore	0.63±0.132	0.293±0.098	0.337	5.33E-05	1.42E-22	-0.456
cg00938429	12	43946880	*ADAMTS20*	TSS1500	S_Shore	0.677±0.081	0.34±0.15	0.337	1.67E-07	1.26E-05	-0.213
cg18736775	8	18248649	*NAT2*	TSS200	S_Shelf	0.738±0.151	0.402±0.155	0.337	1.37E-04	1.64E-21	-0.446
cg26749361	16	30455027	*SEPHS2*	1st exon/3'UTR	N_Shore	0.626±0.189	0.289±0.085	0.336	1.17E-03	4.83E-24	-0.470
cg18554976	19	35629356	*FXYD1*	TSS1500	N_Shelf	0.567±0.17	0.232±0.109	0.335	4.95E-04	1.02E-21	-0.448
cg07094847	7	27160586	*HOXA3*	5'UTR	N_Shelf	0.558±0.101	0.227±0.079	0.332	5.00E-06	1.33E-07	-0.256
cg10550308	3	184969158	*EHHADH*	5'UTR/1st intron	N_Shelf	0.534±0.172	0.203±0.074	0.332	7.27E-04	3.81E-21	-0.442
cg13243168	17	61915833	*SMARCD2*	5'UTR/1st intron	N_Shelf	0.578±0.088	0.247±0.097	0.331	3.71E-07	9.36E-12	-0.327
cg27547703	10	115939024	*TDRD1*	TSS200	Island	0.775±0.119	0.445±0.31	0.330	6.66E-04	7.76E-11	-0.313
cg05385923	7	150495881	*TMEM176B*	5'UTR/1st intron	N_Shore	0.581±0.17	0.251±0.092	0.330	6.24E-04	4.05E-25	-0.480
cg10665490	2	86946078	*RMND5A*	TSS1500	N_Shore	0.728±0.089	0.399±0.129	0.329	3.87E-07	4.17E-12	-0.333
cg19831369	22	26881026	*HPS4*	TSS1500	S_Shore	0.678±0.189	0.35±0.146	0.329	1.13E-03	1.23E-07	-0.257
cg09824255	5	140014002	*CD14*	TSS1500	S_Shore	0.663±0.147	0.335±0.136	0.328	1.39E-04	1.01E-17	-0.405
cg26724545	5	169659950	*C5orf58*	1st exon	Island	0.65±0.106	0.322±0.162	0.328	5.33E-06	1.66E-09	-0.291
cg22624827	1	64058180	*PGM1*	TSS1500	N_Shore	0.626±0.158	0.298±0.072	0.327	4.47E-04	1.43E-15	-0.380
cg03498175	4	185724569	*ACSL1*	5'UTR	Island	0.642±0.164	0.315±0.093	0.327	5.05E-04	8.84E-11	-0.312
cg10021288	2	128175891	*PROC*	TSS200	S_Shelf	0.578±0.167	0.252±0.092	0.327	5.68E-04	2.36E-10	-0.306
cg26204042	11	110163765	*RDX*	5'UTR/1st intron	N_Shelf	0.71±0.139	0.384±0.169	0.326	8.80E-05	4.04E-09	-0.285
cg27475365	6	125476259	*TPD52L1*	5'UTR/1st intron	S_Shore	0.534±0.227	0.208±0.084	0.326	4.45E-03	1.10E-18	-0.416
cg14019146	3	50243930	*SLC38A3*	5'UTR/1st intron	S_Shore	0.511±0.178	0.186±0.066	0.325	1.06E-03	1.05E-49	-0.644
cg16415646	7	41743001	*INHBA*	TSS1500	N_Shelf	0.723±0.102	0.399±0.172	0.324	5.62E-06	5.56E-06	-0.222
cg00574819	4	128981252	*LARP1B*	TSS1500	N_Shore	0.633±0.178	0.309±0.087	0.324	9.89E-04	3.47E-07	-0.248
cg03231596	8	12987546	*DLC1*	5'UTR	N_Shelf	0.666±0.101	0.342±0.153	0.324	3.25E-06	3.08E-12	-0.335
cg09798023	7	27161624	*HOXA3*	5'UTR	N_Shelf	0.655±0.054	0.331±0.162	0.323	1.04E-07	1.52E-19	-0.425
cg10089626	1	45969600	*MMACHC*	5'UTR/1st intron	S_Shelf	0.482±0.113	0.161±0.081	0.322	2.14E-05	4.14E-14	-0.361
cg06163411	7	129691519	*ZC3HC1*	TSS1500	S_Shore	0.715±0.174	0.393±0.215	0.322	9.00E-04	2.23E-08	-0.271
cg08823975	4	185724646	*ACSL1*	5'UTR	Island	0.582±0.145	0.26±0.085	0.322	2.30E-04	1.09E-14	-0.368
cg09866303	6	7142638	*RREB1*	5'UTR/1st intron	S_Shore	0.64±0.129	0.32±0.087	0.320	8.12E-05	2.83E-05	-0.205
cg09514524	8	145729799	*GPT*	1st exon	N_Shelf	0.76±0.213	0.441±0.125	0.319	3.20E-03	1.40E-16	-0.392
cg01287342	8	63999202	*TTPA*	TSS1500	S_Shore	0.573±0.195	0.255±0.126	0.318	1.88E-03	4.82E-14	-0.360
cg00177290	1	110161285	*AMPD2*	TSS1500	N_Shore	0.593±0.149	0.275±0.083	0.318	3.13E-04	3.97E-07	-0.247
cg00842076	14	89881835	*FOXN3*	5'UTR/1st intron	N_Shore	0.631±0.118	0.313±0.133	0.318	1.93E-05	2.23E-11	-0.322
cg23340345	2	233501672	*EFHD1*	5'UTR/1st intron	S_Shore	0.552±0.097	0.236±0.113	0.316	1.99E-06	3.53E-07	-0.248
cg11498870	8	88886243	*DCAF4L2*	5'UTR/1st exon	Island	0.567±0.209	0.251±0.113	0.316	3.08E-03	1.21E-17	-0.404
cg24598094	1	211753582	*SLC30A1*	TSS1500	S_Shore	0.467±0.161	0.151±0.079	0.316	5.99E-04	3.55E-05	-0.202
cg00787429	5	169660328	*C5orf58*	5'UTR/1st intron	S_Shore	0.713±0.079	0.398±0.102	0.315	1.24E-07	4.62E-10	-0.301
cg11755245	3	184969918	*EHHADH*	5'UTR/1st intron	N_Shore	0.576±0.143	0.261±0.087	0.315	2.26E-04	7.35E-20	-0.429
cg06573254	5	174909270	*SFXN1*	5'UTR/1st intron	S_Shelf	0.758±0.049	0.444±0.173	0.315	4.02E-07	2.66E-42	-0.604
cg00013196	8	88885254	*DCAF4L2*	1st exon	N_Shore	0.601±0.174	0.287±0.119	0.315	9.14E-04	1.20E-17	-0.404
cg02426414	17	18265933	*SHMT1*	5'UTR/1st intron	N_Shore	0.559±0.155	0.245±0.069	0.314	5.11E-04	3.56E-10	-0.303
cg19595092	8	22227320	*SLC39A14*	5'UTR/1st intron	S_Shelf	0.611±0.151	0.298±0.1	0.314	3.26E-04	8.17E-07	-0.240
cg07776993	8	145596943	*ADCK5*	TSS1500	N_Shore	0.483±0.209	0.17±0.085	0.313	3.42E-03	6.10E-06	-0.221
cg00887112	2	202644572	*ALS2*	5'UTR/1st intron	N_Shore	0.593±0.165	0.28±0.076	0.313	7.78E-04	7.31E-06	-0.219
cg20712820	7	27160276	*HOXA3*	5'UTR	N_Shelf	0.646±0.123	0.334±0.138	0.313	3.64E-05	6.83E-21	-0.440
cg15747733	10	88850398	*GLUD1*	5'UTR	N_Shelf	0.614±0.124	0.302±0.085	0.312	7.20E-05	2.13E-07	-0.252
cg15223899	22	24891158	*UPB1*	TSS200	Island	0.571±0.15	0.259±0.085	0.312	3.72E-04	7.07E-14	-0.358
cg02219197	2	128175300	*PROC*	TSS1500	S_Shore	0.545±0.124	0.234±0.144	0.312	4.05E-05	1.40E-18	-0.415

cg24469729	7	27160520	*HOXA3*	5'UTR	N_Shelf	0.525±0.058	0.214±0.08	0.311	1.27E-09	4.63E-10	-0.301
cg20901787	17	79936477	*ASPSCR1*	5'UTR/1st intron	S_Shore	0.63±0.161	0.319±0.086	0.311	6.36E-04	2.10E-09	-0.290
cg07074042	22	30783944	*RNF215*	TSS1500	S_Shore	0.539±0.134	0.228±0.102	0.311	1.21E-04	7.32E-10	-0.297
cg04930693	1	8083186	*ERRFI1*	5'UTR/1st intron	N_Shelf	0.714±0.171	0.403±0.12	0.311	8.33E-04	1.02E-10	-0.311
cg02923856	3	23989124	*NR1D2*	5'UTR/1st intron	S_Shore	0.49±0.175	0.179±0.149	0.311	9.63E-04	3.91E-09	-0.285
cg02310286	8	88886432	*DCAF4L2*	TSS200	Island	0.567±0.223	0.258±0.122	0.309	5.05E-03	4.63E-20	-0.431
cg07726085	19	59022705	*SLC27A5*	1st exon	N_Shelf	0.604±0.097	0.294±0.09	0.309	4.27E-06	1.15E-30	-0.526
cg00431187	7	27161749	*HOXA3*	5'UTR	N_Shelf	0.59±0.08	0.281±0.096	0.309	2.46E-07	3.32E-09	-0.286
cg09451188	13	45989144	*SLC25A30*	5'UTR/1st intron	N_Shelf	0.451±0.179	0.144±0.075	0.308	1.52E-03	4.20E-08	-0.266
cg20303572	2	38602546	*ATL2*	5'UTR/1st intron	N_Shore	0.687±0.149	0.38±0.129	0.307	2.83E-04	4.01E-13	-0.348
cg00142933	2	128420784	*LIMS2*	5'UTR/1st intron	N_Shore	0.755±0.084	0.448±0.196	0.307	9.42E-06	2.29E-15	-0.377
cg16295676	10	124766619	*IKZF5*	5'UTR	N_Shore	0.707±0.062	0.4±0.171	0.306	6.89E-07	2.74E-05	-0.205
cg09437283	3	13008096	*IQSEC1*	5'UTR/1st intron	N_Shore	0.484±0.136	0.178±0.077	0.306	2.10E-04	6.49E-10	-0.298
cg24483493	15	63333476	*TPM1*	TSS1500	N_Shore	0.588±0.17	0.282±0.158	0.305	8.80E-04	6.85E-06	-0.220
cg15410675	4	154144800	*TRIM2*	5'UTR/1st intron	S_Shore	0.448±0.151	0.142±0.078	0.305	4.93E-04	3.13E-08	-0.268
cg21852589	7	142981689	*TMEM139*	TSS1500	N_Shelf	0.625±0.171	0.32±0.144	0.305	9.41E-04	9.20E-18	-0.406
cg08755736	8	95957928	*TP53INP1*	5'UTR/1st intron	N_Shelf	0.482±0.171	0.178±0.073	0.305	1.22E-03	2.09E-05	-0.208
cg07649206	3	170742094	*SLC2A2*	5'UTR/1st intron	N_Shelf	0.58±0.149	0.277±0.08	0.303	4.52E-04	1.76E-14	-0.366
cg05578673	1	90307339	*LRRC8D*	5'UTR/1st intron	N_Shore	0.663±0.167	0.36±0.163	0.303	8.19E-04	3.11E-05	-0.204
cg03585070	1	205602206	*ELK4*	TSS1500	S_Shore	0.791±0.033	0.488±0.187	0.303	2.27E-06	5.06E-07	-0.244
cg23361969	3	184971183	*EHHADH*	5'UTR/1st intron	N_Shore	0.487±0.15	0.184±0.076	0.303	4.90E-04	2.69E-06	-0.229
cg01390728	17	45919287	*SCRN2*	TSS1500	S_Shore	0.634±0.175	0.331±0.13	0.302	1.20E-03	9.91E-10	-0.295
cg17848496	2	227661213	*IRS1*	1st exon	N_Shore	0.567±0.18	0.265±0.104	0.302	1.60E-03	7.26E-19	-0.418
cg00492055	3	138312356	*CEP70*	5'UTR/1st intron	N_Shore	0.554±0.163	0.252±0.091	0.302	8.43E-04	4.34E-10	-0.301
cg00653085	1	53385434	*ECHDC2*	5'UTR/1st intron	N_Shore	0.532±0.067	0.23±0.075	0.302	3.30E-08	4.37E-23	-0.461
cg11654662	17	18264018	*SHMT1*	5'UTR/1st intron	N_Shelf	0.604±0.146	0.302±0.128	0.302	2.77E-04	2.24E-35	-0.560
cg26034799	9	5185804	*INSL6*	TSS200	S_Shore	0.57±0.084	0.268±0.112	0.302	5.85E-07	5.43E-06	-0.222
cg17777531	8	88885043	*DCAF4L2*	1st exon	N_Shore	0.555±0.155	0.254±0.128	0.301	4.85E-04	1.86E-13	-0.352
cg23731272	15	67356838	*SMAD3*	TSS1500	N_Shore	0.545±0.203	0.244±0.092	0.301	3.62E-03	5.20E-08	-0.264
cg14554415	15	69222988	*SPESP1*	5'UTR/1st exon	Island	0.567±0.134	0.266±0.121	0.301	1.35E-04	1.04E-05	-0.215
cg17213304	5	78364769	*BHMT2*	TSS1500	N_Shore	0.696±0.118	0.395±0.184	0.300	6.63E-05	1.65E-69	-0.729
cg00383081	1	182362088	*GLUL*	TSS1500	S_Shore	0.727±0.096	0.427±0.288	0.300	6.12E-04	8.34E-65	-0.711
cg09593860	11	113929287	*ZBTB16*	TSS1500	N_Shore	0.624±0.198	0.324±0.181	0.300	3.10E-03	1.37E-07	-0.256
cg23449764	11	915337	*CHID1*	TSS1500	S_Shelf	0.558±0.126	0.259±0.159	0.299	8.31E-05	4.13E-10	-0.302
cg16674116	11	1319689	*TOLLIP*	5'UTR/1st intron	S_Shelf	0.741±0.084	0.443±0.109	0.298	6.03E-07	3.18E-21	-0.443
cg01573501	4	108910070	*HADH*	TSS1500	N_Shore	0.582±0.155	0.284±0.087	0.298	6.37E-04	2.03E-12	-0.337
cg21048763	17	77997997	*TBC1D16*	5'UTR/1st intron	S_Shore	0.623±0.195	0.325±0.138	0.298	2.80E-03	2.96E-05	-0.204
cg01371233	16	30456325	*SEPHS2*	1st exon	N_Shore	0.524±0.159	0.226±0.053	0.298	9.38E-04	3.99E-17	-0.398
cg03032025	5	173316748	*CPEB4*	1st exon	S_Shore	0.533±0.126	0.236±0.077	0.298	1.30E-04	2.00E-08	-0.272
cg06285337	16	27281406	*NSMCE1*	TSS1500	S_Shore	0.579±0.158	0.281±0.067	0.298	8.67E-04	2.85E-06	-0.228
cg16973107	12	57828180	*INHBC*	TSS1500	S_Shelf	0.57±0.167	0.273±0.08	0.297	1.14E-03	6.99E-08	-0.262
cg19841423	20	62366755	*LIME1*	TSS1500	N_Shelf	0.642±0.116	0.345±0.123	0.297	3.46E-05	6.28E-17	-0.396
cg01662334	7	27704065	*HIBADH*	TSS1500	S_Shore	0.715±0.095	0.418±0.2	0.297	2.96E-05	1.75E-32	-0.539
cg10881745	2	216945596	*TMEM169*	TSS1500	N_Shore	0.548±0.155	0.251±0.082	0.297	6.86E-04	1.57E-11	-0.324
cg25302704	8	27470629	*CLU*	5'UTR/1st intron	N_Shore	0.609±0.155	0.313±0.121	0.296	5.77E-04	4.11E-12	-0.333
cg19506201	17	79936561	*ASPSCR1*	5'UTR/1st intron	S_Shore	0.586±0.142	0.29±0.079	0.296	3.65E-04	7.05E-11	-0.314
cg19442470	8	27470225	*CLU*	5'UTR/1st intron	N_Shore	0.58±0.125	0.285±0.089	0.296	1.09E-04	2.24E-07	-0.252
cg24214471	20	36154292	*BLCAP*	5'UTR/1st intron	S_Shelf	0.493±0.122	0.198±0.081	0.295	1.02E-04	5.21E-13	-0.346
cg00994717	10	102731539	*SEMA4G*	TSS1500	S_Shore	0.606±0.112	0.311±0.081	0.295	4.40E-05	8.95E-15	-0.370
cg14222630	9	127961717	*RABEPK*	TSS1500	N_Shore	0.717±0.201	0.422±0.204	0.295	4.19E-03	9.72E-12	-0.327
cg12559925	2	235863365	*SH3BP4*	5'UTR/1st intron	S_Shore	0.676±0.138	0.382±0.181	0.295	2.63E-04	1.02E-26	-0.494
cg11830061	9	5185415	*INSL6*	1st exon	Island	0.707±0.09	0.413±0.173	0.294	8.39E-06	5.13E-10	-0.300
cg09484541	5	74161187	*FAM169A*	5'UTR/1st intron	N_Shore	0.538±0.161	0.244±0.119	0.294	8.05E-04	1.64E-09	-0.291
cg24277788	4	185724837	*ACSL1*	5'UTR/1st intron	S_Shore	0.552±0.135	0.258±0.075	0.294	2.68E-04	1.41E-13	-0.354
cg21871746	7	99616820	*ZKSCAN1*	5'UTR/1st intron	S_Shelf	0.606±0.109	0.312±0.139	0.294	1.96E-05	1.34E-08	-0.275
cg26721627	11	1319713	*TOLLIP*	5'UTR/1st intron	S_Shelf	0.64±0.099	0.346±0.082	0.294	1.22E-05	1.27E-20	-0.437
cg06837799	7	27160960	*HOXA3*	5'UTR	N_Shelf	0.484±0.11	0.19±0.089	0.294	3.26E-05	1.07E-14	-0.368
cg23974688	17	49333393	*MBTD1*	5'UTR	N_Shelf	0.513±0.139	0.22±0.071	0.293	3.57E-04	1.18E-11	-0.326
cg12277225	13	52734032	*NEK3*	TSS1500	S_Shore	0.58±0.147	0.287±0.131	0.293	3.89E-04	5.33E-09	-0.283
cg19853565	19	2540907	*GNG7*	5'UTR	Island	0.547±0.175	0.254±0.099	0.293	1.61E-03	8.75E-06	-0.217
cg11436087	12	57827593	*INHBC*	TSS1500	S_Shelf	0.696±0.122	0.403±0.149	0.293	7.33E-05	1.35E-10	-0.309
cg07704990	5	101834577	*SLCO6A1*	5'UTR/1st exon	Island	0.746±0.113	0.453±0.133	0.293	3.06E-05	5.54E-07	-0.244
cg01897036	2	120282327	*SCTR*	TSS1500	S_Shore	0.725±0.084	0.432±0.241	0.292	1.35E-04	5.32E-07	-0.244
cg02571470	2	73294845	*SFXN5*	5'UTR/1st intron	N_Shelf	0.496±0.048	0.204±0.059	0.292	2.96E-10	1.17E-24	-0.476
cg22856114	2	128175905	*PROC*	TSS200	S_Shelf	0.553±0.164	0.261±0.085	0.292	1.13E-03	2.55E-10	-0.305
cg22957206	3	184096500	*THPO*	5'UTR/1st intron	N_Shelf	0.735±0.121	0.443±0.102	0.292	7.37E-05	2.01E-32	-0.539
cg22957206	3	184096500	*CHRD*	TSS1500	N_Shelf	0.735±0.121	0.443±0.102	0.292	7.37E-05	6.65E-09	-0.281
cg07331053	8	27347861	*EPHX2*	TSS1500	N_Shore	0.556±0.107	0.265±0.094	0.292	2.22E-05	1.61E-16	-0.392
cg20976694	18	55714052	*NEDD4L*	TSS1500	S_Shore	0.499±0.162	0.208±0.076	0.291	1.09E-03	1.11E-09	-0.294
cg08621893	4	123841798	*NUDT6*	5'UTR/1st intron	N_Shelf	0.598±0.18	0.307±0.133	0.291	1.91E-03	3.47E-06	-0.226
cg04977856	14	89886941	*FOXN3*	5'UTR/1st intron	S_Shelf	0.541±0.115	0.25±0.068	0.291	8.28E-05	3.36E-06	-0.227
cg11233163	3	23955549	*NKIRAS1*	5'UTR/1st intron	N_Shelf	0.522±0.143	0.231±0.074	0.290	4.56E-04	1.62E-05	-0.211
cg06682875	5	115150172	*CDO1*	5'UTR/1st intron	N_Shore	0.528±0.165	0.238±0.068	0.290	1.31E-03	1.21E-28	-0.510
cg14864022	6	7142610	*RREB1*	5'UTR/1st intron	S_Shore	0.648±0.144	0.36±0.089	0.289	4.62E-04	4.15E-05	-0.200
cg20227860	4	39368720	*RFC1*	TSS1500	S_Shore	0.557±0.155	0.268±0.079	0.289	8.36E-04	2.63E-11	-0.321
cg10117831	14	89881817	*FOXN3*	5'UTR/1st intron	N_Shore	0.546±0.172	0.258±0.159	0.288	1.48E-03	7.62E-12	-0.329
cg23072629	2	239195537	*PER2*	5'UTR/1st intron	N_Shore	0.504±0.127	0.215±0.08	0.288	1.72E-04	7.41E-07	-0.241
cg06687091	18	43912991	*RNF165*	TSS1500	N_Shore	0.687±0.137	0.398±0.184	0.288	3.13E-04	8.94E-31	-0.527
cg19458020	17	38473119	*RARA*	5'UTR/1st intron	Island	0.474±0.13	0.186±0.104	0.288	1.66E-04	1.11E-07	-0.258
cg04350520	7	95027158	*PON3*	TSS1500	S_Shore	0.494±0.125	0.206±0.081	0.288	1.54E-04	9.47E-09	-0.278
cg08377000	4	90033921	*TIGD2*	TSS200	S_Shore	0.672±0.145	0.384±0.205	0.288	6.43E-04	2.40E-34	-0.553
cg17145652	8	42750650	*RNF170*	5'UTR/1st intron	N_Shore	0.549±0.181	0.261±0.118	0.288	2.17E-03	9.58E-12	-0.327
cg17782713	10	22613360	*BMI1*	5'UTR/1st intron	N_Shore	0.714±0.084	0.426±0.174	0.288	8.02E-06	2.30E-09	-0.289
cg18051316	6	31925660	*SKIV2L*	TSS1500	N_Shore	0.597±0.122	0.31±0.13	0.288	8.35E-05	2.49E-07	-0.251
cg16922340	20	33873526	*EIF6*	TSS1500	S_Shore	0.587±0.149	0.301±0.076	0.287	6.89E-04	8.59E-11	-0.313
cg19326543	2	128422715	*LIMS2*	5'UTR/1st intron	S_Shore	0.57±0.138	0.284±0.095	0.287	3.29E-04	1.20E-10	-0.310
cg00590251	2	119980583	*STEAP3*	TSS1500	N_Shore	0.799±0.105	0.513±0.197	0.287	7.27E-05	3.71E-45	-0.620

cg05169454	2	38602911	*ATL2*	5'UTR/1st intron	N_Shore	0.541±0.145	0.255±0.094	0.286	4.95E-04	9.22E-13	-0.342
cg05380921	16	46921663	*GPT2*	5'UTR	S_Shelf	0.503±0.154	0.217±0.065	0.286	9.44E-04	2.69E-21	-0.444
cg11365440	3	46608179	*LRRC2*	TSS200	S_Shore	0.486±0.152	0.2±0.081	0.286	7.67E-04	6.32E-13	-0.345
cg23011886	5	179230002	*MGAT4B*	TSS200	N_Shelf	0.509±0.153	0.223±0.067	0.286	8.87E-04	1.40E-09	-0.293
cg14166189	11	915440	*CHID1*	TSS1500	S_Shelf	0.716±0.103	0.431±0.264	0.285	5.93E-04	9.04E-12	-0.328
cg03065308	6	137538584	*IFNGR1*	5'UTR/1st intron	N_Shore	0.571±0.176	0.286±0.193	0.285	2.16E-03	4.11E-18	-0.410
cg15507475	7	75624460	*TMEM120A*	TSS1500	S_Shore	0.555±0.153	0.27±0.095	0.285	7.50E-04	2.82E-10	-0.304
cg09246479	22	24891129	*UPB1*	TSS200	Island	0.554±0.148	0.269±0.088	0.285	6.35E-04	3.78E-13	-0.348
cg22707705	2	219646114	*CYP27A1*	TSS1500	N_Shore	0.453±0.142	0.168±0.083	0.285	4.80E-04	1.02E-07	-0.259
cg01345727	13	52734018	*NEK3*	TSS1500	S_Shore	0.522±0.153	0.238±0.131	0.284	6.71E-04	2.04E-05	-0.208
cg21761922	3	16309088	*OXNAD1*	5'UTR/1st intron	S_Shelf	0.601±0.167	0.318±0.13	0.284	1.33E-03	8.09E-26	-0.486
cg07104895	17	79936499	*ASPSCR1*	5'UTR/1st intron	S_Shore	0.571±0.14	0.288±0.077	0.283	4.52E-04	1.18E-09	-0.294
cg11168687	4	185726782	*ACSL1*	5'UTR/1st exon	S_Shelf	0.507±0.116	0.223±0.067	0.283	1.12E-04	6.80E-06	-0.220
cg01879083	5	10306031	*CMBL*	5'UTR/1st intron	N_Shore	0.599±0.183	0.316±0.157	0.283	2.64E-03	1.98E-52	-0.658
cg25610294	14	24777910	*CIDEB*	5'UTR	N_Shore	0.73±0.067	0.448±0.151	0.283	8.16E-07	7.36E-22	-0.449
cg25610294	14	24777910	*LTB4R2*	TSS1500	N_Shore	0.73±0.067	0.448±0.151	0.283	8.16E-07	3.40E-12	-0.334
cg19212391	17	72432790	*GPRC5C*	5'UTR/1st intron	S_Shelf	0.546±0.13	0.263±0.078	0.282	2.54E-04	6.20E-12	-0.330
cg20100668	8	88885469	*DCAF4L2*	1st exon	N_Shore	0.504±0.133	0.222±0.075	0.282	3.28E-04	1.27E-15	-0.380
cg04118119	13	47371987	*ESD*	TSS1500	S_Shore	0.564±0.163	0.282±0.085	0.282	1.33E-03	7.30E-10	-0.297
cg02486855	15	67356942	*SMAD3*	TSS1500	N_Shore	0.477±0.188	0.195±0.071	0.282	3.42E-03	8.06E-07	-0.240
cg09993814	20	36154222	*BLCAP*	5'UTR/1st intron	S_Shelf	0.485±0.111	0.203±0.097	0.282	4.75E-05	3.50E-17	-0.399
cg07843390	19	2541015	*GNG7*	5'UTR	Island	0.531±0.149	0.25±0.099	0.281	6.80E-04	2.55E-05	-0.206
cg22455392	5	169659831	*C5orf58*	TSS200	Island	0.751±0.096	0.47±0.219	0.281	1.32E-04	5.74E-16	-0.385
cg26783146	16	4423632	*VASN*	5'UTR/1st intron	S_Shore	0.537±0.139	0.256±0.077	0.281	4.54E-04	1.98E-05	-0.209
cg05489957	6	30177295	*TRIM26*	5'UTR/1st intron	N_Shelf	0.478±0.139	0.198±0.076	0.281	4.57E-04	2.67E-12	-0.336
cg22780475	19	45281526	*CBLC*	1st exon	S_Shore	0.611±0.141	0.33±0.104	0.281	4.21E-04	9.20E-13	-0.342
cg18241308	7	95026502	*PON3*	TSS1500	S_Shore	0.474±0.111	0.193±0.079	0.281	6.51E-05	3.53E-08	-0.267
cg14153624	13	98626476	*IPO5*	5'UTR	N_Shore	0.7±0.13	0.42±0.167	0.280	2.32E-04	9.59E-08	-0.259
cg08124399	6	74104868	*DDX43*	1st exon	Island	0.679±0.177	0.399±0.154	0.280	2.20E-03	6.66E-12	-0.330
cg15891218	14	61748354	*TMEM30B*	5'UTR/1st exon	S_Shore	0.549±0.134	0.27±0.134	0.280	2.46E-04	2.24E-11	-0.322
cg20123637	3	133467435	*TF*	5'UTR/1st intron	S_Shelf	0.538±0.13	0.258±0.07	0.279	3.01E-04	1.12E-11	-0.326
cg03481658	7	6051355	*AIMP2*	5'UTR	S_Shelf	0.623±0.086	0.344±0.122	0.279	2.28E-06	6.17E-10	-0.299
cg03821443	11	1320329	*TOLLIP*	5'UTR/1st intron	S_Shelf	0.499±0.162	0.22±0.076	0.279	1.47E-03	2.45E-12	-0.336
cg04819351	15	36872220	*C15orf41*	5'UTR/1st intron	S_Shore	0.476±0.118	0.197±0.097	0.279	9.53E-05	2.35E-08	-0.271
cg08083088	11	118899758	*SLC37A4*	5'UTR	N_Shore	0.53±0.175	0.251±0.082	0.279	2.32E-03	9.27E-06	-0.216
cg12508078	17	27505845	*MYO18A*	5'UTR/1st intron	S_Shore	0.493±0.144	0.214±0.079	0.279	6.30E-04	1.59E-06	-0.234
cg07294814	8	144348648	*GLI4*	TSS1500	N_Shore	0.513±0.172	0.234±0.082	0.279	2.06E-03	6.97E-07	-0.242
cg11625933	3	81811322	*GBE1*	TSS1500	S_Shore	0.44±0.155	0.161±0.085	0.279	1.05E-03	1.13E-10	-0.311
cg18069356	17	66594875	*FAM20A*	5'UTR/1st intron	N_Shore	0.512±0.162	0.234±0.076	0.278	1.45E-03	1.49E-07	-0.255
cg15835805	5	132162441	*SHROOM1*	5'UTR	S_Shore	0.44±0.116	0.162±0.069	0.278	1.22E-04	2.61E-12	-0.336
cg17152436	11	1318418	*TOLLIP*	5'UTR/1st intron	S_Shore	0.861±0.117	0.582±0.283	0.278	1.62E-03	2.08E-14	-0.365
cg01241390	5	80596277	*ZCCHC9*	TSS1500	N_Shore	0.495±0.115	0.217±0.086	0.278	8.53E-05	1.61E-10	-0.308
cg21126626	5	68787747	*OCLN*	TSS1500	N_Shore	0.461±0.134	0.184±0.072	0.278	4.02E-04	5.74E-07	-0.243
cg20029140	10	12235293	*NUDT5*	5'UTR/1st intron	N_Shelf	0.596±0.143	0.318±0.087	0.278	5.68E-04	5.42E-18	-0.408
cg04919234	6	99392478	*FBXL4*	5'UTR/1st intron	N_Shelf	0.555±0.154	0.278±0.07	0.277	1.12E-03	2.07E-09	-0.290
cg14494812	17	48941039	*TOB1*	5'UTR	N_Shore	0.544±0.141	0.266±0.077	0.277	5.60E-04	2.60E-18	-0.412
cg06090298	10	89263946	*MINPP1*	TSS1500	N_Shore	0.708±0.122	0.431±0.154	0.276	1.41E-04	1.45E-06	-0.235
cg25879118	7	95026672	*PON3*	TSS1500	S_Shore	0.522±0.119	0.245±0.082	0.276	1.37E-04	1.15E-07	-0.258
cg08651937	3	156530506	*PA2G4P4*	TSS1500	N_Shelf	0.486±0.125	0.209±0.056	0.276	2.96E-04	3.00E-05	-0.204
cg19074198	21	46954058	*SLC19A1*	5'UTR	N_Shelf	0.761±0.057	0.485±0.181	0.276	5.95E-06	1.36E-15	-0.380
cg22558871	7	72723150	*NSUN5*	TSS1500	S_Shore	0.465±0.202	0.19±0.099	0.275	5.75E-03	4.18E-06	-0.225
cg18560264	3	52324089	*GLYCTK*	5'UTR/1st intron	S_Shelf	0.497±0.159	0.221±0.068	0.275	1.44E-03	1.21E-10	-0.310
cg00202496	16	11037903	*CLEC16A*	TSS1500	S_Shore	0.489±0.129	0.214±0.08	0.275	2.96E-04	6.72E-06	-0.220
cg00008971	1	45966925	*MMACHC*	5'UTR/1st intron	S_Shore	0.54±0.132	0.265±0.071	0.275	3.87E-04	6.74E-13	-0.344
cg16129213	21	33030780	*SOD1*	TSS1500	N_Shore	0.613±0.168	0.339±0.093	0.274	1.90E-03	4.88E-10	-0.300
cg24083364	1	2325254	*RER1*	5'UTR/1st intron	S_Shore	0.645±0.158	0.371±0.109	0.274	1.19E-03	6.99E-08	-0.262
cg00944166	5	101834675	*SLCO6A1*	5'UTR/1st exon	Island	0.733±0.143	0.459±0.201	0.274	8.59E-04	8.11E-09	-0.279
cg00054210	8	65711644	*CYP7B1*	TSS1500	Island	0.406±0.152	0.133±0.105	0.274	2.15E-03	1.21E-13	-0.354
cg01206378	1	95698827	*RWDD3*	TSS1500	N_Shore	0.423±0.138	0.149±0.066	0.274	5.85E-04	6.25E-06	-0.221
cg06264922	11	1319648	*TOLLIP*	5'UTR/1st intron	S_Shelf	0.611±0.058	0.337±0.082	0.274	9.77E-09	1.97E-22	-0.455
cg17875935	15	45666384	*GATM*	5'UTR	N_Shelf	0.487±0.115	0.213±0.066	0.273	1.37E-04	7.58E-06	-0.219
cg04071104	3	53878642	*CHDH*	5'UTR/1st intron	N_Shore	0.617±0.208	0.344±0.15	0.273	7.12E-03	2.41E-13	-0.350
cg06073041	5	141346577	*RNF14*	5'UTR/1st intron	N_Shore	0.65±0.171	0.377±0.195	0.273	2.59E-03	7.24E-06	-0.219
cg05875176	17	66594586	*FAM20A*	5'UTR/1st intron	N_Shore	0.543±0.145	0.27±0.099	0.273	6.78E-04	1.14E-19	-0.427
cg03044471	11	1321009	*TOLLIP*	5'UTR/1st intron	S_Shelf	0.712±0.141	0.439±0.202	0.273	8.53E-04	7.36E-06	-0.219
cg15589042	17	4691376	*GLTPD2*	TSS1500	S_Shore	0.644±0.135	0.371±0.109	0.272	3.51E-04	1.86E-09	-0.291
cg13859860	10	51569878	*NCOA4*	5'UTR/1st intron	N_Shelf	0.674±0.04	0.402±0.119	0.272	9.96E-09	1.34E-09	-0.293
cg04290171	1	207924482	*CD46*	TSS1500	N_Shore	0.479±0.139	0.207±0.077	0.272	5.63E-04	4.10E-05	-0.201
cg16265717	1	65993149	*LEPR*	5'UTR	S_Shore	0.441±0.117	0.168±0.07	0.272	1.57E-04	3.46E-06	-0.226
cg18198550	6	35889893	*SRPK1*	TSS1500	S_Shore	0.614±0.18	0.341±0.142	0.272	2.95E-03	1.43E-05	-0.212
cg00421645	5	43312201	*HMGCS1*	5'UTR/1st intron	N_Shore	0.501±0.136	0.229±0.08	0.272	4.71E-04	6.69E-12	-0.330
cg17379666	17	71304784	*CDC42EP4*	5'UTR/1st intron	N_Shelf	0.458±0.118	0.186±0.086	0.272	1.28E-04	2.25E-11	-0.322
cg02179764	22	37419795	*MPST*	5'UTR	S_Shelf	0.542±0.116	0.27±0.08	0.272	1.23E-04	2.56E-14	-0.364
cg24194775	9	35791475	*NPR2*	TSS1500	S_Shore	0.45±0.151	0.179±0.104	0.271	9.13E-04	1.82E-13	-0.352
cg14489933	2	128422619	*LIMS2*	5'UTR/1st intron	S_Shore	0.673±0.096	0.402±0.162	0.271	2.64E-05	2.29E-26	-0.491
cg11518349	1	247171838	*ZNF695*	TSS1500	S_Shore	0.648±0.151	0.377±0.177	0.271	1.06E-03	5.56E-11	-0.316
cg24994355	10	99445520	*AVPI1*	5'UTR/1st intron	N_Shore	0.519±0.143	0.248±0.072	0.271	7.48E-04	7.42E-08	-0.261
cg07906527	1	204116483	*ETNK2*	5'UTR/1st intron	N_Shelf	0.585±0.159	0.314±0.096	0.271	1.45E-03	2.41E-39	-0.586
cg07062764	3	170744540	*SLC2A2*	5'UTR/1st exon	N_Shore	0.541±0.144	0.27±0.076	0.270	7.65E-04	2.78E-07	-0.250
cg16806210	11	118398318	*TTC36*	1st exon	N_Shelf	0.717±0.047	0.446±0.172	0.270	3.56E-06	6.37E-42	-0.602
cg16297435	6	43028296	*KLC4*	5'UTR	S_Shore	0.523±0.143	0.252±0.075	0.270	7.57E-04	4.69E-13	-0.346
cg16297435	6	43028296	*MRPL2*	TSS1500	S_Shore	0.523±0.143	0.252±0.075	0.270	7.57E-04	1.04E-12	-0.341
cg03053290	1	95698378	*RWDD3*	TSS1500	N_Shore	0.548±0.151	0.278±0.101	0.270	1.00E-03	9.61E-07	-0.239
cg18549946	14	58862146	*TOMM20L*	TSS1500	N_Shore	0.465±0.106	0.195±0.076	0.270	5.93E-05	7.64E-06	-0.218
cg02439077	10	72202215	*NODAL*	5'UTR/1st intron	S_Shore	0.551±0.089	0.281±0.064	0.270	1.36E-05	3.43E-07	-0.248
cg02858826	14	75389222	*RPS6KL1*	TSS200	N_Shore	0.724±0.109	0.455±0.159	0.270	7.40E-05	2.68E-12	-0.336
cg23763877	20	5988223	*CRLS1*	5'UTR/1st intron	S_Shore	0.525±0.142	0.255±0.073	0.270	7.18E-04	2.49E-10	-0.305

cg24804106	8	18872143	*PSD3*	TSS1500	S_Shore	0.642±0.119	0.372±0.116	0.270	1.17E-04	1.01E-09	-0.295
cg01257194	9	116161247	*ALAD*	5'UTR/1st intron	N_Shore	0.525±0.096	0.255±0.062	0.269	3.13E-05	6.06E-19	-0.419
cg08597832	8	144416327	*TOP1MT*	5'UTR	N_Shore	0.577±0.172	0.308±0.081	0.269	2.64E-03	2.74E-06	-0.229
cg07922062	19	33867079	*CEBPG*	5'UTR/1st intron	S_Shelf	0.493±0.128	0.224±0.062	0.269	3.84E-04	2.81E-13	-0.349
cg03533858	1	2324396	*RER1*	5'UTR/1st intron	S_Shore	0.495±0.156	0.226±0.066	0.269	1.49E-03	1.25E-05	-0.213
cg16681268	7	117067616	*ASZ1*	TSS200	Island	0.848±0.16	0.579±0.283	0.269	5.51E-03	3.57E-05	-0.202
cg13913666	7	1198122	*ZFAND2A*	5'UTR/1st intron	N_Shore	0.464±0.131	0.195±0.079	0.269	4.02E-04	2.87E-07	-0.250
cg13892257	15	86313941	*KLHL25*	5'UTR/1st intron	S_Shore	0.627±0.133	0.358±0.18	0.268	4.83E-04	6.63E-11	-0.314
cg17065262	17	27506097	*MYO18A*	5'UTR/1st intron	S_Shelf	0.478±0.164	0.21±0.085	0.268	1.96E-03	1.27E-06	-0.236
cg00908631	1	51433616	*CDKN2C*	TSS1500	N_Shore	0.587±0.167	0.319±0.157	0.268	2.09E-03	9.07E-12	-0.328
cg04049033	17	1553661	*RILP*	TSS1500	S_Shore	0.547±0.117	0.279±0.081	0.268	1.45E-04	1.45E-08	-0.275
cg21181391	8	88886411	*DCAF4L2*	TSS200	Island	0.529±0.121	0.261±0.114	0.267	1.49E-04	2.84E-15	-0.376
cg19817912	7	27162768	*HOXA3*	5'UTR/1st intron	N_Shore	0.548±0.069	0.28±0.119	0.267	3.47E-07	1.67E-07	-0.254
cg23146998	13	113776915	*F10*	TSS200	S_Shelf	0.506±0.14	0.239±0.083	0.267	6.73E-04	6.25E-08	-0.263
cg15122621	1	156341523	*RHBG*	5'UTR/1st intron	S_Shelf	0.467±0.114	0.201±0.087	0.266	1.09E-04	3.50E-15	-0.375
cg07147573	3	42918806	*CYP8B1*	TSS1500	N_Shelf	0.643±0.135	0.377±0.206	0.266	8.79E-04	5.21E-24	-0.470
cg07304015	11	47665221	*MTCH2*	TSS1500	S_Shore	0.513±0.154	0.247±0.096	0.266	1.28E-03	2.16E-10	-0.306
cg00744433	21	18884067	*CXADR*	TSS1500	N_Shore	0.51±0.148	0.244±0.075	0.266	1.09E-03	7.18E-06	-0.219
cg10961323	1	24127976	*GALE*	TSS1500	S_Shore	0.507±0.138	0.241±0.102	0.266	5.39E-04	5.60E-08	-0.264
cg04308185	17	38084377	*ORMDL3*	TSS1500	S_Shore	0.44±0.155	0.175±0.09	0.266	1.41E-03	1.24E-09	-0.294
cg06794441	1	67520733	*SLC35D1*	TSS1500	S_Shore	0.693±0.18	0.427±0.15	0.266	3.52E-03	2.62E-14	-0.363
cg06787519	5	78364863	*BHMT2*	TSS1500	N_Shore	0.54±0.115	0.275±0.114	0.265	9.93E-05	2.86E-40	-0.592
cg06639440	11	915170	*CHID1*	TSS200	S_Shelf	0.598±0.11	0.333±0.189	0.265	1.86E-04	1.75E-10	-0.308
cg08261841	7	142982463	*TMEM139*	1st exon	N_Shelf	0.675±0.179	0.411±0.213	0.264	4.88E-03	9.21E-40	-0.589
cg14382976	7	117067624	*ASZ1*	TSS200	Island	0.761±0.161	0.497±0.276	0.264	5.91E-03	3.80E-05	-0.201
cg02441982	17	76183641	*TK1*	TSS1500	S_Shore	0.518±0.141	0.254±0.068	0.264	8.33E-04	3.21E-06	-0.227
cg16571786	17	74237602	*RNF157*	TSS1500	S_Shore	0.532±0.109	0.269±0.131	0.264	6.14E-05	3.08E-10	-0.304
cg20557688	2	230133040	*PID1*	5'UTR/1st intron	N_Shelf	0.693±0.094	0.43±0.279	0.264	1.56E-03	1.22E-37	-0.575
cg03532673	7	102787746	*NAPEPLD*	5'UTR/1st intron	N_Shore	0.445±0.108	0.182±0.073	0.264	9.08E-05	4.12E-10	-0.302
cg01355753	16	30457524	*SEPHS2*	TSS1500	S_Shore	0.379±0.141	0.116±0.04	0.263	9.99E-04	1.64E-11	-0.324
cg01946364	5	175296158	*CPLX2*	5'UTR	N_Shelf	0.558±0.129	0.295±0.102	0.263	3.36E-04	2.13E-08	-0.272
cg12910830	5	162929657	*MAT2B*	TSS1500	N_Shelf	0.73±0.082	0.467±0.215	0.263	1.46E-04	1.34E-07	-0.256
cg27143326	3	58227354	*ABHD6*	5'UTR/1st intron	S_Shelf	0.572±0.172	0.309±0.079	0.263	2.98E-03	1.04E-17	-0.405
cg04918002	16	87423124	*FBXO31*	5'UTR	N_Shelf	0.552±0.154	0.29±0.054	0.263	1.68E-03	4.84E-07	-0.245
cg02709052	11	86748071	*TMEM135*	TSS1500	N_Shore	0.549±0.151	0.286±0.068	0.263	1.40E-03	1.95E-05	-0.209
cg06097580	16	56456934	*AMFR*	5'UTR/1st intron	N_Shore	0.777±0.045	0.514±0.176	0.263	7.04E-06	4.66E-19	-0.420
cg17772163	5	68787760	*OCLN*	TSS1500	N_Shore	0.458±0.13	0.196±0.078	0.262	4.31E-04	3.92E-08	-0.267
cg26718585	2	128176454	*PROC*	5'UTR/1st intron	N_Shelf	0.567±0.148	0.304±0.089	0.262	1.11E-03	2.57E-18	-0.412
cg12279019	6	43741054	*VEGFA*	5'UTR/1st intron	S_Shore	0.557±0.144	0.295±0.077	0.262	9.74E-04	4.52E-06	-0.224
cg24411597	4	123841683	*NUDT6*	5'UTR/1st intron	N_Shelf	0.687±0.183	0.425±0.184	0.262	4.88E-03	1.66E-08	-0.274
cg20874324	2	62930083	*EHBP1*	5'UTR/1st intron	N_Shelf	0.809±0.05	0.548±0.216	0.262	8.74E-05	4.65E-32	-0.536
cg13508949	4	166131313	*KLHL2*	1st exon	S_Shelf	0.787±0.076	0.526±0.196	0.262	8.26E-05	6.66E-14	-0.358
cg20701461	1	45801852	*MUTYH*	5'UTR	N_Shelf	0.466±0.136	0.205±0.078	0.262	6.55E-04	1.02E-09	-0.295
cg11617144	8	145729772	*GPT*	1st exon	N_Shore	0.648±0.194	0.387±0.062	0.261	6.32E-03	2.41E-09	-0.289
cg00571819	9	133455886	*OC10027221*	TSS1500	S_Shore	0.465±0.147	0.203±0.071	0.261	1.16E-03	1.80E-05	-0.210
cg04255044	16	87415168	*FBXO31*	5'UTR	N_Shore	0.458±0.14	0.197±0.065	0.261	8.63E-04	2.15E-15	-0.377
cg08662766	19	4536321	*PLIN5*	TSS1500	S_Shore	0.596±0.16	0.335±0.104	0.261	1.83E-03	3.43E-25	-0.481
cg25312054	13	110434467	*IRS2*	1st exon	Island	0.673±0.116	0.412±0.132	0.261	1.27E-04	5.17E-10	-0.300
cg16582986	6	160514231	*LOC729603*	1st exon	S_Shore	0.755±0.1	0.494±0.175	0.261	8.73E-05	6.35E-09	-0.281
cg03010018	11	116708299	*APOA1*	5'UTR/1st exon	S_Shore	0.559±0.172	0.299±0.074	0.261	3.20E-03	1.84E-06	-0.232
cg01486987	19	39443861	*FBXO17*	5'UTR/1st intron	S_Shelf	0.694±0.046	0.433±0.103	0.261	4.78E-09	3.50E-06	-0.226
cg13702181	14	77791657	*GSTZ1*	5'UTR	S_Shelf	0.581±0.072	0.32±0.115	0.260	7.20E-07	2.51E-07	-0.251
cg12235622	1	43856473	*MED8*	TSS1500	S_Shore	0.591±0.112	0.331±0.118	0.260	9.78E-05	1.48E-08	-0.274
cg00204262	19	39323158	*ECH1*	TSS1500	S_Shore	0.461±0.134	0.201±0.06	0.260	7.01E-04	4.07E-09	-0.285
cg20985067	7	95026538	*PON3*	TSS1500	S_Shore	0.446±0.112	0.186±0.077	0.260	1.31E-04	1.45E-07	-0.256
cg03606098	1	1690840	*NADK*	TSS1500	N_Shore	0.627±0.146	0.367±0.124	0.259	9.66E-04	4.01E-22	-0.452
cg14016166	7	75543425	*POR*	TSS1500	N_Shore	0.757±0.122	0.498±0.145	0.259	2.39E-04	1.90E-10	-0.307
cg14719865	5	101834365	*SLCO6A1*	1st exon	Island	0.637±0.106	0.378±0.113	0.259	5.52E-05	2.76E-05	-0.205
cg25603883	1	154944357	*SHC1*	5'UTR/1st intron	N_Shelf	0.401±0.241	0.142±0.081	0.259	1.88E-02	9.95E-10	-0.295
cg03085549	16	75150819	*LDHD*	TSS200	S_Shore	0.429±0.123	0.171±0.062	0.259	3.64E-04	2.84E-10	-0.304
cg21733794	20	36154388	*BLCAP*	5'UTR/1st intron	S_Shelf	0.623±0.039	0.365±0.182	0.258	1.28E-05	4.28E-27	-0.497
cg24439334	1	2345374	*PEX10*	TSS1500	S_Shore	0.491±0.124	0.233±0.075	0.258	3.50E-04	4.30E-05	-0.200
cg08370787	5	148208041	*ADRB2*	1st exon/3'UTR	S_Shore	0.44±0.145	0.182±0.077	0.258	1.14E-03	2.07E-11	-0.322
cg13719901	3	46608139	*LRRC2*	TSS200	S_Shore	0.408±0.127	0.15±0.07	0.258	4.50E-04	1.14E-09	-0.294
cg16452651	21	35016873	*ITSN1*	5'UTR/1st intron	S_Shelf	0.404±0.15	0.146±0.073	0.258	1.43E-03	4.36E-06	-0.224
cg03558769	2	242497595	*BOK*	TSS1500	N_Shore	0.554±0.14	0.296±0.085	0.258	8.30E-04	8.33E-07	-0.240
cg24051786	3	47514673	*SCAP*	5'UTR/1st intron	N_Shelf	0.652±0.135	0.394±0.131	0.258	5.46E-04	1.47E-11	-0.325
cg18671057	6	13489248	*GFOD1*	TSS1500	S_Shore	0.895±0.066	0.637±0.266	0.257	9.35E-04	3.63E-06	-0.226
cg13561372	7	121781778	*AASS*	5'UTR/1st intron	N_Shelf	0.635±0.084	0.378±0.166	0.257	2.53E-05	8.90E-23	-0.458
cg12369869	3	184035508	*EIF4G1*	5'UTR	S_Shelf	0.554±0.163	0.297±0.107	0.257	2.34E-03	2.16E-06	-0.231
cg02930951	20	48806066	*CEBPB*	TSS1500	N_Shore	0.571±0.107	0.314±0.063	0.257	1.28E-04	1.39E-08	-0.275
cg14093103	1	61546214	*NFIA*	TSS1500	N_Shelf	0.585±0.148	0.329±0.162	0.257	1.26E-03	8.06E-10	-0.297
cg16267236	3	133464463	*TF*	5'UTR	N_Shore	0.609±0.155	0.352±0.092	0.257	1.73E-03	8.18E-19	-0.418
cg01032398	19	45408121	*APOE*	TSS1500	N_Shelf	0.483±0.129	0.227±0.094	0.257	4.21E-04	8.65E-08	-0.260
cg04414860	6	116575894	*TSPYL4*	TSS1500	S_Shore	0.507±0.075	0.25±0.161	0.256	1.13E-05	6.58E-10	-0.298
cg13102658	1	2324224	*RER1*	5'UTR/1st intron	S_Shore	0.464±0.136	0.207±0.076	0.256	7.52E-04	8.10E-07	-0.240
cg14527389	7	41742993	*INHBA*	TSS1500	N_Shelf	0.789±0.09	0.533±0.19	0.256	9.71E-05	2.78E-05	-0.205
cg27140914	3	39095348	*WDR48*	5'UTR/1st intron	S_Shore	0.634±0.163	0.378±0.115	0.256	2.32E-03	1.02E-06	-0.238
cg19092837	1	10271724	*KIF1B*	5'UTR/1st exon	S_Shore	0.441±0.164	0.185±0.076	0.256	2.63E-03	2.46E-10	-0.305
cg07531356	9	5185951	*INSL6*	TSS1500	S_Shore	0.689±0.08	0.434±0.176	0.256	3.42E-05	1.82E-10	-0.307
cg03009240	19	3507867	*FZR1*	5'UTR/1st intron	S_Shore	0.645±0.227	0.389±0.209	0.255	1.84E-02	2.68E-17	-0.400
cg16409259	11	68117110	*LRP5*	5'UTR	S_Shore	0.59±0.194	0.334±0.131	0.255	6.98E-03	2.88E-08	-0.269
cg09238666	16	66584358	*TK2*	TSS1500	N_Shore	0.436±0.144	0.181±0.064	0.255	1.25E-03	5.96E-11	-0.315
cg12250062	2	128175068	*PROC*	TSS1500	S_Shore	0.773±0.103	0.518±0.176	0.255	1.32E-04	1.89E-09	-0.290
cg18735146	13	21140606	*IFT88*	TSS1500	N_Shore	0.435±0.127	0.18±0.07	0.255	4.97E-04	7.88E-08	-0.261
cg01089602	13	113760101	*F7*	1st exon	N_Shore	0.453±0.128	0.198±0.073	0.254	5.15E-04	2.67E-15	-0.376
cg05338976	7	114561850	*MDFIC*	TSS1500	N_Shore	0.463±0.102	0.209±0.112	0.254	4.82E-05	6.66E-06	-0.220

cg10120856	4	157996959	*GLRB*	TSS1500	N_Shore	0.58±0.065	0.326±0.171	0.254	1.34E-05	2.76E-08	-0.269
cg16677647	16	67750395	*GFOD2*	5'UTR/1st intron	N_Shelf	0.56±0.148	0.306±0.082	0.254	1.41E-03	9.75E-22	-0.448
cg00470183	6	116575913	*TSPYL4*	TSS1500	S_Shore	0.448±0.087	0.195±0.154	0.253	2.36E-05	4.27E-08	-0.266
cg16787352	14	102975405	*ANKRD9*	5'UTR/1st intron	Island	0.472±0.105	0.219±0.083	0.253	8.20E-05	2.43E-05	-0.206
cg07543138	21	16434067	*NRIP1*	5'UTR/1st intron	N_Shelf	0.495±0.199	0.242±0.132	0.253	8.28E-03	1.43E-05	-0.212
cg02593507	19	4535582	*PLIN5*	TSS1500	S_Shore	0.541±0.134	0.288±0.086	0.253	6.91E-04	4.99E-13	-0.346
cg17347634	8	65711916	*CYP7B1*	TSS1500	S_Shore	0.438±0.223	0.186±0.1	0.253	1.47E-02	9.12E-18	-0.406
cg03054529	7	30029938	*SCRN1*	TSS1500	S_Shore	0.703±0.092	0.45±0.2	0.253	1.90E-04	1.12E-05	-0.215
cg04354393	17	33775963	*SLFN13*	TSS200	N_Shore	0.438±0.136	0.186±0.095	0.253	7.26E-04	2.01E-05	-0.208
cg26141247	14	94644017	*PPP4R4*	5'UTR	S_Shelf	0.738±0.045	0.485±0.205	0.252	7.09E-05	1.19E-26	-0.493
cg08030373	3	51574322	*RAD54L2*	5'UTR	S_Shore	0.745±0.084	0.493±0.197	0.252	1.22E-04	8.21E-06	-0.218
cg08099331	16	11037187	*DEXI*	TSS1500	S_Shore	0.689±0.123	0.437±0.217	0.252	1.08E-03	7.92E-17	-0.395
cg08099331	16	11037187	*CLEC16A*	TSS1500	S_Shore	0.689±0.123	0.437±0.217	0.252	1.08E-03	6.60E-09	-0.281
cg12058064	15	86315201	*KLHL25*	5'UTR/1st intron	S_Shelf	0.508±0.122	0.256±0.135	0.252	2.98E-04	1.34E-09	-0.293
cg04839706	16	46920075	*GPT2*	5'UTR	S_Shore	0.579±0.192	0.327±0.16	0.252	7.40E-03	1.59E-30	-0.525
cg24064639	17	66592243	*FAM20A*	5'UTR/1st intron	N_Shelf	0.505±0.164	0.253±0.113	0.251	2.70E-03	1.76E-10	-0.308
cg00969323	2	242627866	*DTYMK*	TSS1500	S_Shore	0.543±0.109	0.292±0.106	0.251	1.06E-04	3.71E-06	-0.226
cg03582736	1	95586852	*TMEM56*	5'UTR/1st intron	S_Shelf	0.669±0.113	0.418±0.149	0.251	1.77E-04	1.50E-26	-0.492
cg13788387	2	97652806	*FAM178B*	TSS1500	S_Shore	0.815±0.107	0.564±0.287	0.251	3.59E-03	1.96E-14	-0.365
cg23284609	5	10306947	*CMBL*	5'UTR/1st intron	N_Shore	0.556±0.126	0.305±0.106	0.250	3.96E-04	3.39E-39	-0.585
cg22082456	1	53392538	*SCP2*	TSS1500	N_Shore	0.465±0.141	0.215±0.085	0.250	1.06E-03	4.95E-13	-0.346
cg17547295	5	157282503	*CLINT1*	5'UTR/1st intron	N_Shelf	0.416±0.154	0.166±0.068	0.250	2.09E-03	8.44E-06	-0.217
cg02898499	16	1831468	*NUBP2*	TSS1500	N_Shore	0.618±0.058	0.368±0.129	0.250	4.77E-07	6.20E-09	-0.281
cg11777420	2	120769933	*EPB41L5*	TSS1500	N_Shore	0.459±0.125	0.21±0.079	0.250	4.56E-04	1.34E-09	-0.293
cg00884805	1	68296607	*GNG12*	5'UTR/1st intron	N_Shore	0.536±0.159	0.287±0.121	0.250	2.28E-03	6.33E-13	-0.345
cg14510299	1	27928494	*AHDC1*	5'UTR/1st intron	N_Shore	0.394±0.186	0.144±0.164	0.250	6.58E-03	2.01E-05	-0.208
cg18550003	19	49259284	*FGF21*	5'UTR	S_Shelf	0.552±0.117	0.302±0.116	0.250	2.13E-04	9.86E-10	-0.295
cg19554037	11	1318877	*TOLLIP*	5'UTR/1st intron	S_Shore	0.65±0.165	0.4±0.075	0.250	3.24E-03	8.99E-14	-0.356
cg06520369	5	139938342	*SRA1*	TSS1500	S_Shore	0.823±0.042	0.573±0.206	0.250	8.45E-05	2.35E-08	-0.271
cg15824543	1	1695391	*NADK*	5'UTR	S_Shelf	0.716±0.139	0.467±0.154	0.249	1.02E-03	2.47E-14	-0.364
cg22472290	19	52391480	*ZNF577*	TSS1500	S_Shore	0.567±0.175	0.318±0.132	0.249	4.28E-03	3.47E-27	-0.498
cg14961476	1	9130738	*SLC2A5*	5'UTR/1st intron	N_Shore	0.526±0.141	0.277±0.135	0.249	1.03E-03	1.47E-06	-0.235
cg04305808	7	99614209	*ZKSCAN1*	5'UTR/1st intron	S_Shore	0.403±0.111	0.154±0.077	0.249	1.78E-04	2.13E-07	-0.252
cg18880500	1	53392492	*SCP2*	TSS1500	N_Shore	0.45±0.136	0.201±0.079	0.249	8.94E-04	7.26E-13	-0.344
cg24269657	13	113759771	*F7*	TSS1500	N_Shore	0.466±0.129	0.217±0.07	0.248	6.58E-04	5.77E-16	-0.385
cg18038894	17	48506385	*ACSF2*	5'UTR/1st intron	S_Shelf	0.544±0.12	0.296±0.071	0.248	3.70E-04	7.33E-10	-0.297
cg03464896	3	170744964	*SLC2A2*	TSS200	N_Shore	0.521±0.078	0.273±0.068	0.248	5.13E-06	5.20E-31	-0.528
cg12421282	1	41158986	*NFYC*	5'UTR/1st intron	S_Shore	0.547±0.133	0.299±0.095	0.248	6.80E-04	1.02E-10	-0.311
cg26398586	5	101834472	*SLCO6A1*	1st exon	Island	0.794±0.095	0.546±0.186	0.248	1.63E-04	4.88E-12	-0.332
cg06098215	10	51575702	*NCOA4*	5'UTR/1st intron	S_Shelf	0.371±0.114	0.123±0.068	0.248	2.63E-04	1.69E-06	-0.233
cg16324018	2	85580627	*ELMOD3*	TSS1500	N_Shore	0.478±0.14	0.23±0.081	0.247	1.14E-03	6.51E-08	-0.262
cg08606497	8	28244769	*ZNF395*	TSS1500	S_Shore	0.431±0.097	0.184±0.062	0.247	7.03E-05	8.09E-13	-0.343
cg12433575	6	30881464	*VARS2*	TSS1500	N_Shore	0.443±0.134	0.195±0.067	0.247	8.92E-04	3.71E-10	-0.302
cg26025557	3	42842344	*HIGD1A*	5'UTR/1st intron	N_Shelf	0.792±0.059	0.545±0.187	0.247	4.33E-05	2.58E-30	-0.523
cg02102684	16	4421445	*VASN*	TSS1500	N_Shore	0.455±0.124	0.208±0.055	0.247	5.92E-04	3.14E-05	-0.204
cg11143063	2	128177475	*PROC*	5'UTR/1st intron	N_Shelf	0.528±0.139	0.281±0.068	0.247	1.15E-03	1.72E-20	-0.435
cg25103337	1	9293583	*H6PD*	TSS1500	N_Shore	0.366±0.169	0.12±0.058	0.247	4.07E-03	2.33E-13	-0.351
cg01026796	5	54602543	*DHX29*	5'UTR/1st intron	N_Shore	0.412±0.124	0.165±0.063	0.247	5.30E-04	1.13E-06	-0.237
cg14533644	9	133453231	*OC10027221*	1st exon	N_Shore	0.792±0.065	0.546±0.233	0.246	4.38E-04	3.35E-06	-0.227
cg14172428	9	71787160	*TJP2*	5'UTR	N_Shore	0.587±0.134	0.341±0.148	0.246	8.33E-04	2.00E-07	-0.253
cg20932822	3	32279236	*CMTM8*	TSS1500	N_Shore	0.451±0.127	0.206±0.067	0.246	6.31E-04	9.78E-24	-0.467
cg07958192	4	90033974	*TIGD2*	5'UTR/1st exon	S_Shore	0.584±0.143	0.338±0.223	0.246	2.93E-03	4.26E-51	-0.651
cg02747210	13	113776917	*F10*	TSS200	S_Shelf	0.532±0.156	0.286±0.089	0.246	2.37E-03	4.15E-08	-0.266
cg00912573	2	227661291	*IRS1*	1st exon	N_Shore	0.501±0.13	0.255±0.084	0.246	6.74E-04	6.25E-17	-0.396
cg12377368	11	18128126	*SAAL1*	TSS1500	S_Shore	0.548±0.059	0.302±0.169	0.245	1.46E-05	4.18E-07	-0.246
cg01455981	1	20511095	*UBXN10*	TSS1500	N_Shore	0.588±0.112	0.343±0.132	0.245	1.83E-04	4.60E-25	-0.479
cg04980849	15	45671347	*GATM*	5'UTR	S_Shore	0.503±0.114	0.258±0.163	0.245	3.12E-04	1.91E-06	-0.232
cg08884029	12	110012500	*MVK*	5'UTR/1st intron	S_Shore	0.604±0.189	0.359±0.143	0.245	7.56E-03	7.07E-18	-0.407
cg08884029	12	110012500	*MMAB*	TSS1500	S_Shore	0.604±0.189	0.359±0.143	0.245	7.56E-03	8.54E-10	-0.296
cg01066936	22	41776843	*TEF*	TSS1500	N_Shore	0.531±0.152	0.286±0.101	0.245	2.02E-03	2.98E-14	-0.363
cg13615367	3	58479231	*KCTD6*	5'UTR/1st intron	S_Shore	0.508±0.147	0.263±0.091	0.245	1.60E-03	6.92E-27	-0.495
cg12016746	11	68084453	*LRP5*	5'UTR/1st intron	S_Shelf	0.391±0.16	0.146±0.064	0.245	3.00E-03	4.24E-05	-0.200
cg08012294	8	11723816	*CTSB*	5'UTR/1st intron	N_Shore	0.476±0.122	0.231±0.078	0.245	4.29E-04	5.08E-06	-0.223
cg13443575	17	33775961	*SLFN13*	TSS200	N_Shore	0.513±0.125	0.268±0.154	0.245	5.29E-04	5.75E-07	-0.243
cg18495047	22	24891166	*UPB1*	TSS200	Island	0.521±0.119	0.277±0.072	0.245	3.94E-04	2.86E-14	-0.363
cg12616941	10	22612797	*BMI1*	5'UTR/1st intron	N_Shore	0.711±0.163	0.466±0.208	0.245	4.89E-03	8.77E-13	-0.343
cg17564844	22	23413784	*GNAZ*	5'UTR/1st intron	S_Shore	0.481±0.149	0.237±0.065	0.245	2.01E-03	1.39E-06	-0.235
cg18092474	15	75019302	*CYP1A1*	TSS1500	Island	0.409±0.171	0.164±0.074	0.244	4.40E-03	2.60E-06	-0.229
cg06527213	15	86315211	*KLHL25*	5'UTR/1st intron	S_Shelf	0.532±0.132	0.288±0.151	0.244	8.05E-04	8.49E-10	-0.296
cg08696640	6	41531327	*FOXP4*	5'UTR/1st intron	S_Shelf	0.478±0.12	0.233±0.067	0.244	4.35E-04	2.65E-12	-0.336
cg03537537	8	88886262	*DCAF4L2*	5'UTR/1st exon	Island	0.677±0.207	0.433±0.262	0.244	2.08E-02	1.51E-31	-0.532
cg03668539	19	7553324	*PEX11G*	5'UTR/1st intron	N_Shore	0.515±0.142	0.271±0.092	0.244	1.30E-03	4.83E-11	-0.317
cg06374748	17	52978856	*TOM1L1*	5'UTR/1st intron	S_Shore	0.47±0.133	0.226±0.07	0.244	9.12E-04	3.86E-17	-0.399
cg10580282	3	50340564	*HYAL1*	1st exon	S_Shelf	0.499±0.113	0.255±0.08	0.244	2.38E-04	2.84E-07	-0.250
cg27126059	22	24891338	*UPB1*	5'UTR/1st exon	Island	0.552±0.138	0.308±0.073	0.244	1.20E-03	1.87E-27	-0.500
cg01915822	8	145596918	*ADCK5*	TSS1500	N_Shore	0.502±0.164	0.258±0.07	0.243	3.59E-03	8.22E-14	-0.357
cg03837313	11	57195350	*SLC43A3*	TSS1500	S_Shore	0.568±0.158	0.326±0.217	0.243	4.83E-03	3.25E-13	-0.349
cg26511386	7	142982262	*TMEM139*	1st exon	N_Shelf	0.538±0.174	0.295±0.146	0.243	5.09E-03	1.28E-30	-0.525
cg01800148	1	9130886	*SLC2A5*	5'UTR/1st intron	N_Shore	0.602±0.111	0.36±0.211	0.242	8.78E-04	2.17E-09	-0.289
cg10835823	5	169659763	*C5orf58*	TSS1500	N_Shore	0.771±0.106	0.529±0.21	0.242	7.06E-04	1.49E-18	-0.415
cg02872476	16	90086806	*DBNDD1*	TSS1500	S_Shore	0.623±0.13	0.381±0.115	0.242	6.59E-04	6.18E-30	-0.520
cg10806586	14	45366116	*C14orf28*	TSS1500	N_Shore	0.441±0.087	0.199±0.078	0.242	1.75E-05	1.13E-05	-0.214
cg04428805	17	65239522	*HELZ*	5'UTR	N_Shore	0.558±0.123	0.316±0.134	0.242	4.53E-04	1.38E-09	-0.293
cg03533472	16	46919112	*GPT2*	5'UTR/1st exon	S_Shore	0.345±0.152	0.103±0.048	0.242	2.53E-03	1.55E-12	-0.339
cg25742458	19	18081620	*KCNN1*	5'UTR	N_Shelf	0.845±0.069	0.603±0.226	0.241	4.42E-04	2.34E-05	-0.207
cg22531018	7	142981779	*TMEM139*	TSS1500	N_Shelf	0.547±0.143	0.306±0.092	0.241	1.49E-03	1.30E-18	-0.415
cg06711560	9	139746365	*MAMDC4*	TSS1500	S_Shore	0.575±0.121	0.334±0.098	0.241	3.90E-04	5.90E-07	-0.243

cg06801028	10	51575592	*NCOA4*	5'UTR/1st intron	S_Shelf	0.379±0.1	0.138±0.055	0.241	1.32E-04	3.97E-06	-0.225
cg05677630	22	22020012	*PPIL2*	TSS1500	N_Shore	0.579±0.096	0.338±0.089	0.241	4.99E-05	3.02E-10	-0.304
cg18252102	13	98796790	*FARP1*	5'UTR/1st intron	S_Shore	0.495±0.129	0.254±0.067	0.241	8.30E-04	6.35E-12	-0.330
cg05920345	6	44282149	*AARS2*	TSS1500	S_Shore	0.762±0.09	0.522±0.145	0.241	4.03E-05	1.17E-05	-0.214
cg21897315	3	81811448	*GBE1*	TSS1500	S_Shore	0.637±0.168	0.396±0.162	0.241	4.72E-03	1.20E-10	-0.310
cg03412763	11	125771358	*PUS3*	5'UTR/1st intron	N_Shelf	0.698±0.069	0.457±0.211	0.240	2.47E-04	7.75E-11	-0.313
cg04749433	14	58862246	*TOMM20L*	TSS1500	N_Shore	0.515±0.108	0.275±0.078	0.240	1.86E-04	1.19E-07	-0.257
cg11208165	3	16308357	*OXNAD1*	5'UTR/1st intron	S_Shore	0.506±0.158	0.266±0.092	0.240	2.93E-03	3.42E-34	-0.552
cg22747501	7	29233476	*CHN2*	TSS1500	N_Shore	0.373±0.12	0.133±0.047	0.240	6.11E-04	5.50E-23	-0.460
cg21771682	3	11036537	*SLC6A1*	5'UTR/1st intron	S_Shore	0.501±0.181	0.262±0.102	0.240	6.58E-03	3.37E-25	-0.483
cg17334359	5	162929830	*MAT2B*	TSS1500	N_Shelf	0.574±0.106	0.335±0.204	0.239	6.75E-04	1.44E-05	-0.212
cg06613263	5	142779552	*NR3C1*	5'UTR	N_Shelf	0.37±0.049	0.131±0.058	0.239	8.29E-09	2.03E-10	-0.307
cg20029153	17	27505257	*MYO18A*	5'UTR/1st intron	S_Shore	0.421±0.136	0.182±0.069	0.239	1.26E-03	1.61E-05	-0.211
cg22332066	1	210501621	*HHAT*	5'UTR/1st exon	N_Shore	0.55±0.178	0.311±0.139	0.239	6.23E-03	2.55E-09	-0.288
cg05311626	11	1320114	*TOLLIP*	5'UTR/1st intron	S_Shelf	0.668±0.067	0.429±0.109	0.239	9.06E-07	5.25E-18	-0.409
cg12210543	19	12175107	*ZNF844*	TSS1500	N_Shore	0.491±0.14	0.252±0.188	0.239	2.07E-03	1.38E-14	-0.367
cg11700490	7	95026937	*PON3*	TSS1500	S_Shore	0.421±0.131	0.182±0.069	0.238	9.79E-04	2.53E-08	-0.270
cg21932672	1	95584445	*TMEM56*	5'UTR/1st intron	S_Shore	0.417±0.144	0.179±0.053	0.238	2.02E-03	1.64E-25	-0.483
cg24334259	3	138311416	*CEP70*	5'UTR/1st intron	N_Shore	0.684±0.141	0.446±0.13	0.238	1.45E-03	4.88E-07	-0.245
cg00653041	7	122524465	*CADPS2*	5'UTR	N_Shore	0.649±0.167	0.412±0.172	0.238	5.11E-03	7.28E-09	-0.280
cg00354381	11	57195187	*SLC43A3*	TSS1500	S_Shore	0.513±0.179	0.276±0.158	0.238	7.04E-03	2.28E-12	-0.337
cg23165541	15	64338164	*DAPK2*	5'UTR/1st intron	Island	0.469±0.086	0.231±0.086	0.238	1.77E-05	3.05E-11	-0.320
cg06047020	13	93877835	*GPC6*	TSS1500	N_Shore	0.434±0.137	0.197±0.085	0.237	1.28E-03	4.84E-06	-0.223
cg05423018	7	36193854	*EEPD1*	5'UTR	S_Shore	0.437±0.108	0.2±0.061	0.237	2.62E-04	8.57E-13	-0.343
cg26705561	10	102279791	*SEC31B*	TSS200	S_Shore	0.553±0.111	0.317±0.158	0.236	3.27E-04	4.15E-06	-0.225
cg13615983	9	139746152	*MAMDC4*	TSS1500	S_Shore	0.668±0.075	0.432±0.176	0.236	7.03E-05	1.06E-15	-0.381
cg06412323	20	43108009	*TTPAL*	5'UTR	S_Shelf	0.531±0.101	0.296±0.17	0.236	2.43E-04	2.73E-25	-0.481
cg08616234	15	86313973	*KLHL25*	5'UTR/1st intron	S_Shore	0.668±0.111	0.433±0.153	0.236	3.14E-04	5.55E-12	-0.331
cg13046608	11	57195382	*SLC43A3*	TSS1500	S_Shore	0.58±0.156	0.344±0.231	0.236	6.47E-03	2.16E-14	-0.365
cg26124860	3	42918661	*CYP8B1*	TSS1500	N_Shelf	0.632±0.182	0.397±0.197	0.235	9.88E-03	1.35E-24	-0.475
cg08537660	11	12699661	*TEAD1*	5'UTR	S_Shelf	0.528±0.12	0.292±0.156	0.235	5.92E-04	2.40E-06	-0.230
cg18205787	11	915239	*CHID1*	TSS200	S_Shelf	0.617±0.095	0.382±0.217	0.235	8.03E-04	3.75E-10	-0.302
cg01052512	16	2098739	*TSC2*	5'UTR	S_Shore	0.415±0.127	0.179±0.052	0.235	9.39E-04	1.10E-13	-0.355
cg01052512	16	2098739	*NTHL1*	TSS1500	S_Shore	0.415±0.127	0.179±0.052	0.235	9.39E-04	5.78E-09	-0.282
cg20701556	1	95698924	*RWDD3*	TSS1500	N_Shore	0.412±0.129	0.177±0.079	0.235	9.15E-04	1.08E-06	-0.237
cg06791446	10	123355268	*FGFR2*	5'UTR/1st intron	N_Shore	0.484±0.11	0.249±0.096	0.235	2.09E-04	3.34E-07	-0.248
cg07730946	4	185743727	*ACSL1*	5'UTR/1st intron	N_Shelf	0.666±0.07	0.431±0.155	0.235	1.92E-05	8.44E-25	-0.477
cg17703554	8	88886339	*DCAF4L2*	TSS200	Island	0.52±0.106	0.285±0.105	0.235	1.55E-04	1.55E-16	-0.391
cg21339084	2	128422433	*LIMS2*	5'UTR/1st intron	S_Shore	0.487±0.108	0.252±0.08	0.235	2.06E-04	5.09E-07	-0.244
cg26697065	16	30456379	*SEPHS2*	1st exon	N_Shore	0.5±0.148	0.265±0.064	0.234	2.44E-03	3.11E-13	-0.349
cg20081724	7	102787173	*NAPEPLD*	5'UTR/1st intron	N_Shelf	0.577±0.149	0.342±0.21	0.234	4.25E-03	1.11E-06	-0.237
cg02614016	10	89263900	*MINPP1*	TSS1500	N_Shore	0.614±0.136	0.379±0.146	0.234	1.36E-03	4.56E-10	-0.301
cg06135495	18	47338985	*SCARNA17*	TSS1500	N_Shore	0.518±0.131	0.283±0.062	0.234	1.16E-03	1.98E-07	-0.253
cg19987060	6	52530180	*LOC730101*	1st exon	S_Shore	0.47±0.121	0.236±0.076	0.234	5.74E-04	4.70E-18	-0.409
cg05654329	5	177581506	*NHP2*	TSS1500	S_Shore	0.521±0.141	0.287±0.105	0.234	1.58E-03	6.61E-08	-0.262
cg14283647	2	227662014	*IRS1*	1st exon	N_Shore	0.518±0.129	0.284±0.078	0.234	9.51E-04	6.94E-09	-0.280
cg01205165	1	204119766	*ETNK2*	5'UTR/1st intron	N_Shore	0.454±0.072	0.221±0.085	0.234	2.27E-06	7.08E-37	-0.570
cg27506210	22	32150725	*DEPDC5*	5'UTR/1st intron	S_Shore	0.398±0.119	0.165±0.063	0.233	5.89E-04	2.26E-12	-0.337
cg07251788	22	19280165	*CLTCL1*	TSS1500	S_Shore	0.528±0.158	0.294±0.084	0.233	3.60E-03	1.94E-08	-0.272
cg03893872	1	210501634	*HHAT*	5'UTR/1st exon	N_Shore	0.559±0.194	0.326±0.137	0.233	1.13E-02	8.49E-09	-0.279
cg16432031	1	12674914	*DHRS3*	5'UTR/1st intron	N_Shelf	0.468±0.135	0.234±0.061	0.233	1.43E-03	6.84E-12	-0.330
cg21238609	17	38472961	*RARA*	5'UTR/1st intron	Island	0.417±0.11	0.184±0.085	0.233	2.43E-04	1.33E-08	-0.275
cg16580728	8	109800338	*TMEM74*	TSS1500	S_Shore	0.817±0.062	0.584±0.201	0.233	1.83E-04	1.45E-08	-0.275
cg06126593	16	20910405	*LYRM1*	TSS1500	N_Shore	0.513±0.105	0.281±0.133	0.233	1.66E-04	4.93E-06	-0.223
cg06126593	16	20910405	*DCUN1D3*	5'UTR/1st intron	N_Shore	0.513±0.105	0.281±0.133	0.233	1.66E-04	6.25E-06	-0.220
cg20329620	13	113759894	*F7*	TSS1500	N_Shore	0.5±0.118	0.267±0.067	0.233	5.54E-04	1.16E-18	-0.416
cg00287477	4	185721584	*ACSL1*	5'UTR	N_Shelf	0.515±0.07	0.282±0.153	0.233	1.88E-05	2.68E-20	-0.433
cg00287477	4	185721584	*SLED1*	TSS1500	N_Shelf	0.515±0.07	0.282±0.153	0.233	1.88E-05	2.77E-08	-0.269
cg16814786	10	51575763	*NCOA4*	5'UTR/1st intron	S_Shelf	0.411±0.14	0.178±0.098	0.233	1.60E-03	4.47E-07	-0.246
cg01987333	2	242750561	*NEU4*	5'UTR/1st intron	N_Shelf	0.515±0.133	0.282±0.075	0.232	1.22E-03	8.31E-11	-0.313
cg11915001	19	12175268	*ZNF844*	TSS1500	N_Shore	0.516±0.133	0.283±0.113	0.232	1.07E-03	5.04E-24	-0.471
cg21472642	7	29233468	*CHN2*	TSS1500	N_Shore	0.396±0.132	0.164±0.056	0.232	1.34E-03	1.51E-26	-0.492
cg10201616	19	11198556	*LDLR*	TSS1500	N_Shelf	0.796±0.071	0.563±0.234	0.232	8.76E-04	5.25E-06	-0.222
cg25051805	7	23637943	*CCDC126*	5'UTR	S_Shore	0.414±0.115	0.182±0.064	0.232	4.58E-04	1.39E-08	-0.275
cg17326451	7	86975534	*CROT*	5'UTR/1st intron	S_Shore	0.396±0.12	0.164±0.065	0.232	6.18E-04	5.10E-06	-0.223
cg17901584	1	55353706	*DHCR24*	TSS1500	S_Shore	0.4±0.102	0.168±0.066	0.232	1.69E-04	3.01E-07	-0.249
cg18712919	2	28112170	*RBKS*	5'UTR/1st intron	N_Shore	0.752±0.075	0.521±0.165	0.231	5.24E-05	1.08E-10	-0.311
cg07785936	2	133174635	*GPR39*	1st exon	N_Shore	0.487±0.124	0.255±0.066	0.231	8.26E-04	3.18E-11	-0.319
cg27567495	3	53877436	*CHDH*	5'UTR/1st intron	N_Shelf	0.546±0.149	0.315±0.089	0.231	2.56E-03	8.26E-12	-0.328
cg06212760	1	46664937	*POMGNT1*	5'UTR/1st intron	N_Shelf	0.64±0.162	0.408±0.11	0.231	4.32E-03	5.49E-10	-0.300
cg06493930	11	22689814	*GAS2*	5'UTR/1st exon	S_Shore	0.51±0.147	0.278±0.111	0.231	2.33E-03	1.50E-21	-0.446
cg25052156	10	123355454	*FGFR2*	5'UTR/1st intron	N_Shore	0.605±0.169	0.374±0.179	0.231	6.89E-03	1.36E-17	-0.404
cg02019444	21	35016787	*ITSN1*	5'UTR/1st intron	S_Shelf	0.337±0.144	0.106±0.06	0.231	2.26E-03	2.46E-06	-0.230
cg03284308	16	81039325	*CENPN*	TSS1500	N_Shore	0.441±0.153	0.21±0.149	0.231	3.31E-03	1.34E-06	-0.235
cg11677744	2	235863589	*SH3BP4*	5'UTR/1st intron	S_Shore	0.522±0.087	0.291±0.133	0.231	3.88E-05	7.76E-23	-0.459
cg05424060	7	79768675	*GNAI1*	5'UTR/1st intron	S_Shelf	0.377±0.124	0.146±0.071	0.231	7.87E-04	3.96E-10	-0.302
cg15597984	22	42527061	*CYP2D6*	TSS1500	S_Shore	0.452±0.084	0.221±0.066	0.231	2.48E-05	1.17E-05	-0.214
cg00423871	19	4827986	*TICAM1*	5'UTR/1st intron	N_Shelf	0.519±0.133	0.288±0.097	0.231	1.16E-03	2.16E-07	-0.252
cg00982641	5	169659845	*C5orf58*	TSS200	Island	0.636±0.152	0.406±0.215	0.231	5.73E-03	3.31E-12	-0.334
cg09378425	7	150067896	*REPIN1*	5'UTR	S_Shore	0.54±0.182	0.31±0.181	0.231	1.02E-02	4.83E-08	-0.265
cg16689434	16	90089547	*GAS8*	5'UTR/1st intron	S_Shelf	0.469±0.141	0.239±0.105	0.230	1.80E-03	3.48E-08	-0.268
cg25671484	6	42927993	*GNMT*	TSS1500	N_Shore	0.444±0.106	0.214±0.077	0.230	2.18E-04	1.62E-05	-0.211
cg23202388	11	914849	*CHID1*	5'UTR/1st exon	S_Shelf	0.511±0.097	0.281±0.179	0.230	3.22E-04	2.65E-08	-0.270
cg27247252	8	67975895	*COPS5*	TSS1500	N_Shore	0.49±0.116	0.26±0.128	0.230	4.28E-04	3.60E-09	-0.286
cg20497704	18	43913147	*RNF165*	TSS1500	N_Shore	0.489±0.082	0.259±0.104	0.230	1.29E-05	8.40E-21	-0.439
cg06293365	7	75624427	*TMEM120A*	TSS1500	S_Shore	0.467±0.138	0.238±0.08	0.230	1.71E-03	6.95E-12	-0.329
cg06980173	1	154376344	*IL6R*	TSS1500	N_Shore	0.629±0.059	0.399±0.204	0.230	2.29E-04	1.29E-07	-0.257

cg06417125	5	169659847	*C5orf58*	TSS200	Island	0.681±0.108	0.451±0.19	0.230	7.63E-04	2.99E-11	-0.320
cg26975524	8	1713012	*CLN8*	5'UTR/1st intron	S_Shore	0.356±0.093	0.127±0.046	0.230	1.18E-04	2.60E-08	-0.270
cg06219660	1	1695509	*NADK*	5'UTR	S_Shelf	0.629±0.064	0.4±0.097	0.229	6.31E-07	5.94E-07	-0.243
cg01262667	19	19385393	*TM6SF2*	TSS1500	S_Shore	0.69±0.096	0.46±0.194	0.229	5.02E-04	3.20E-15	-0.375
cg11211951	8	145729740	*GPT*	1st exon	N_Shore	0.657±0.139	0.429±0.084	0.229	1.76E-03	1.09E-11	-0.326
cg13981319	19	10446556	*ICAM3*	5'UTR	S_Shore	0.697±0.169	0.468±0.153	0.229	6.46E-03	7.41E-08	-0.261
cg20353653	15	85143219	*ZSCAN2*	TSS1500	N_Shore	0.486±0.142	0.258±0.078	0.229	2.15E-03	1.20E-11	-0.326
cg00330789	8	99306953	*NIPAL2*	TSS1500	S_Shore	0.496±0.173	0.267±0.12	0.229	6.67E-03	6.07E-07	-0.243
cg23824183	8	124285055	*ZHX1*	5'UTR/1st intron	N_Shore	0.397±0.132	0.168±0.069	0.228	1.38E-03	8.68E-10	-0.296
cg00154455	8	146023573	*ZNF517*	TSS1500	N_Shore	0.512±0.155	0.284±0.089	0.228	3.54E-03	1.00E-17	-0.405
cg08266168	13	113760006	*F7*	TSS200	N_Shore	0.447±0.115	0.219±0.062	0.228	5.39E-04	6.61E-15	-0.371
cg23921523	21	45876245	*LRRC3*	5'UTR/1st intron	Island	0.534±0.13	0.306±0.065	0.228	1.25E-03	9.31E-12	-0.328
cg17142134	3	170744641	*SLC2A2*	5'UTR/1st exon	N_Shore	0.473±0.134	0.245±0.062	0.228	1.55E-03	7.22E-07	-0.241
cg14204433	1	7844775	*PER3*	5'UTR	Island	0.462±0.103	0.234±0.093	0.228	1.56E-04	7.75E-13	-0.343
cg14851700	1	182362230	*GLUL*	TSS1500	S_Shore	0.678±0.059	0.45±0.293	0.228	4.99E-03	1.11E-40	-0.594
cg16736989	7	150495791	*TMEM176B*	5'UTR/1st intron	N_Shore	0.532±0.16	0.304±0.092	0.228	4.43E-03	9.47E-34	-0.549
cg20708135	11	76493489	*TSKU*	5'UTR/1st intron	N_Shore	0.567±0.169	0.34±0.126	0.228	6.08E-03	4.64E-40	-0.590
cg15237661	3	11037574	*SLC6A1*	5'UTR/1st intron	S_Shelf	0.714±0.069	0.487±0.124	0.227	4.97E-06	7.64E-44	-0.613
cg25514503	1	7845070	*PER3*	5'UTR	Island	0.665±0.174	0.438±0.174	0.227	8.67E-03	4.11E-29	-0.514
cg06642677	1	203829775	*SNRPE*	TSS1500	N_Shore	0.423±0.13	0.196±0.089	0.227	1.14E-03	7.59E-06	-0.219
cg19390582	19	16740428	*MED26*	TSS1500	S_Shore	0.406±0.113	0.179±0.077	0.227	4.08E-04	1.58E-05	-0.211
cg01579001	12	56107347	*ITGA7*	TSS1500	N_Shelf	0.795±0.097	0.568±0.197	0.227	6.53E-04	2.61E-20	-0.434
cg02206980	6	13574034	*SIRT5*	TSS1500	N_Shore	0.332±0.147	0.105±0.046	0.227	3.03E-03	9.86E-10	-0.295
cg09380135	19	40789866	*AKT2*	5'UTR/1st intron	N_Shore	0.497±0.118	0.271±0.074	0.227	6.20E-04	2.22E-08	-0.271
cg02957185	1	161068781	*KLHDC9*	1st exon	Island	0.568±0.192	0.342±0.159	0.227	1.33E-02	3.25E-19	-0.422
cg09855983	7	29233211	*CHN2*	TSS1500	N_Shore	0.706±0.202	0.48±0.181	0.226	1.85E-02	7.21E-28	-0.504
cg13986417	12	110012484	*MVK*	5'UTR/1st intron	S_Shore	0.653±0.177	0.427±0.182	0.226	1.01E-02	4.67E-24	-0.470
cg13986417	12	110012484	*MMAB*	TSS1500	S_Shore	0.653±0.177	0.427±0.182	0.226	1.01E-02	1.84E-11	-0.323
cg05688348	4	53146	*ZNF595*	TSS200	N_Shore	0.507±0.056	0.281±0.091	0.226	1.25E-07	3.02E-14	-0.363
cg17300723	11	62647206	*SLC3A2*	TSS1500	N_Shore	0.475±0.06	0.25±0.118	0.225	1.37E-06	1.24E-06	-0.236
cg16370701	6	43029051	*KLC4*	5'UTR	S_Shore	0.577±0.143	0.351±0.135	0.225	2.41E-03	7.99E-25	-0.477
cg02621528	3	133464047	*TF*	5'UTR	N_Shore	0.582±0.159	0.357±0.134	0.225	4.63E-03	1.49E-30	-0.525
cg01510388	8	65711658	*CYP7B1*	TSS1500	Island	0.39±0.194	0.165±0.107	0.225	1.31E-02	4.55E-14	-0.360
cg13924974	18	12656824	*SPIRE1*	TSS200	N_Shore	0.468±0.148	0.243±0.14	0.225	3.13E-03	1.99E-17	-0.402
cg07808761	19	37329760	*ZNF790*	5'UTR/1st intron	S_Shore	0.427±0.106	0.202±0.083	0.225	2.33E-04	1.94E-05	-0.209
cg03017946	4	72897281	*NPFFR2*	TSS1500	N_Shore	0.654±0.091	0.429±0.162	0.225	1.69E-04	5.92E-09	-0.282
cg00582941	20	32888855	*AHCY*	5'UTR/1st intron	N_Shore	0.502±0.106	0.278±0.062	0.225	3.16E-04	5.39E-20	-0.430
cg11316386	1	247275808	*C1orf229*	TSS200	N_Shelf	0.496±0.145	0.271±0.143	0.224	2.72E-03	2.77E-12	-0.335
cg18204685	3	15641821	*BTD*	TSS1500	N_Shore	0.633±0.2	0.409±0.191	0.224	1.91E-02	1.26E-15	-0.380
cg07312641	7	150778955	*FASTK*	TSS1500	Island	0.527±0.124	0.303±0.077	0.224	9.40E-04	6.18E-06	-0.221
cg23974739	5	6637525	*SRD5A1*	5'UTR/1st intron	S_Shelf	0.621±0.065	0.398±0.146	0.224	1.44E-05	9.41E-10	-0.296
cg15652212	7	142981776	*TMEM139*	TSS1500	N_Shelf	0.571±0.136	0.348±0.102	0.224	1.70E-03	5.75E-18	-0.408
cg20758593	5	169659863	*C5orf58*	TSS200	Island	0.631±0.098	0.407±0.161	0.224	2.79E-04	6.65E-11	-0.314
cg10519437	14	35184556	*CFL2*	TSS1500	S_Shore	0.537±0.157	0.313±0.158	0.224	5.02E-03	1.58E-11	-0.324
cg09024606	1	2324221	*RER1*	5'UTR/1st intron	S_Shore	0.419±0.14	0.196±0.072	0.223	2.25E-03	7.94E-08	-0.261
cg10210624	19	58662595	*ZNF329*	TSS1500	N_Shelf	0.511±0.14	0.287±0.168	0.223	2.80E-03	2.13E-12	-0.337
cg12404831	2	192114017	*MYO1B*	5'UTR/1st intron	S_Shelf	0.433±0.134	0.21±0.124	0.223	1.58E-03	1.23E-27	-0.502
cg01812328	2	211091510	*ACADL*	TSS1500	S_Shore	0.7±0.11	0.477±0.199	0.223	1.32E-03	1.82E-25	-0.483
cg10772086	3	133464651	*TF*	5'UTR	N_Shore	0.422±0.113	0.199±0.05	0.223	6.29E-04	3.20E-16	-0.388
cg08032525	3	184096413	*THPO*	5'UTR/1st intron	N_Shelf	0.752±0.102	0.529±0.189	0.223	7.71E-04	5.09E-31	-0.529
cg08032525	3	184096413	*CHRD*	TSS1500	N_Shelf	0.752±0.102	0.529±0.189	0.223	7.71E-04	3.04E-12	-0.335
cg18251984	17	20799162	*CCDC144NL*	1st exon	Island	0.708±0.089	0.485±0.13	0.223	6.20E-05	5.75E-07	-0.243
cg08751913	6	18387077	*RNF144B*	TSS1500	N_Shore	0.378±0.104	0.155±0.078	0.222	2.38E-04	2.50E-06	-0.230
cg17197774	8	59467209	*SDCBP*	5'UTR/1st intron	S_Shore	0.357±0.173	0.135±0.088	0.222	7.91E-03	5.93E-21	-0.440
cg06277588	6	13489246	*GFOD1*	TSS1500	S_Shore	0.868±0.043	0.646±0.215	0.222	4.60E-04	2.38E-05	-0.207
cg19146119	2	234668042	*UGT1A1*	TSS1500	S_Shelf	0.687±0.116	0.465±0.202	0.222	1.91E-03	5.72E-14	-0.359
cg21659949	22	22020025	*PPIL2*	TSS1500	N_Shore	0.449±0.048	0.228±0.066	0.222	1.13E-08	3.13E-09	-0.287
cg01217071	2	201450323	*AOX1*	TSS1500	N_Shore	0.364±0.138	0.142±0.054	0.222	2.27E-03	8.51E-10	-0.296
cg23888304	17	1554059	*RILP*	TSS1500	S_Shore	0.809±0.058	0.587±0.167	0.221	4.53E-05	6.84E-09	-0.281
cg11405475	1	203830200	*SNRPE*	TSS1500	N_Shore	0.393±0.117	0.172±0.074	0.221	6.67E-04	5.24E-10	-0.300
cg09736922	3	184095976	*THPO*	5'UTR	N_Shelf	0.467±0.12	0.246±0.046	0.221	1.02E-03	2.30E-29	-0.516
cg05267955	6	30881562	*VARS2*	TSS1500	Island	0.39±0.11	0.169±0.068	0.221	4.48E-04	4.45E-08	-0.266
cg00042254	1	46019963	*AKR1A1*	5'UTR	S_Shelf	0.606±0.144	0.385±0.106	0.221	2.75E-03	1.25E-22	-0.457
cg26291655	15	86315115	*KLHL25*	5'UTR/1st intron	S_Shelf	0.414±0.117	0.194±0.088	0.221	6.33E-04	3.91E-09	-0.285
cg15729154	11	2925026	*SLC22A18AS*	5'UTR/1st exon	S_Shore	0.494±0.139	0.273±0.071	0.221	2.38E-03	9.14E-16	-0.382
cg12746427	4	53362	*ZNF595*	5'UTR/1st exon	Island	0.525±0.085	0.305±0.089	0.221	3.19E-05	5.22E-18	-0.409
cg01550445	11	72929983	*P2RY2*	5'UTR/1st intron	S_Shore	0.497±0.173	0.276±0.166	0.220	9.69E-03	1.13E-26	-0.493
cg08594681	8	27468684	*CLU*	1st exon	N_Shelf	0.358±0.1	0.138±0.062	0.220	2.31E-04	8.01E-08	-0.261
cg27379442	17	4801965	*C17orf107*	TSS1500	N_Shore	0.615±0.117	0.395±0.122	0.220	6.55E-04	3.72E-10	-0.302
cg15832329	2	242296992	*FARP2*	5'UTR/1st intron	S_Shore	0.499±0.081	0.279±0.07	0.220	2.43E-05	2.74E-07	-0.250
cg02078520	1	84944298	*RPF1*	TSS1500	N_Shore	0.591±0.087	0.371±0.143	0.220	8.80E-05	4.25E-06	-0.224
cg21967668	19	49259265	*FGF21*	5'UTR	S_Shelf	0.481±0.112	0.261±0.091	0.220	4.54E-04	4.44E-07	-0.246
cg02346062	6	24491168	*GPLD1*	TSS1500	N_Shelf	0.573±0.079	0.354±0.127	0.220	2.52E-05	1.68E-16	-0.391
cg07572927	14	21155679	*ANG*	5'UTR/1st intron	S_Shelf	0.471±0.119	0.251±0.061	0.220	8.91E-04	5.84E-06	-0.221
cg03546163	6	35654363	*FKBP5*	5'UTR	N_Shore	0.52±0.158	0.301±0.124	0.219	5.11E-03	3.94E-11	-0.318
cg00359590	8	27469673	*CLU*	5'UTR/1st intron	N_Shelf	0.482±0.117	0.262±0.106	0.219	6.12E-04	1.15E-09	-0.294
cg16419724	5	68711519	*MARVELD2*	5'UTR/1st intron	Island	0.4±0.104	0.181±0.064	0.219	3.14E-04	9.80E-13	-0.342
cg01189606	3	48471901	*PLXNB1*	TSS1500	S_Shore	0.4±0.114	0.181±0.053	0.219	7.20E-04	2.07E-06	-0.231
cg02789126	2	234667777	*UGT1A1*	TSS1500	S_Shelf	0.773±0.096	0.554±0.182	0.219	5.60E-04	5.15E-12	-0.331
cg21377071	15	84323963	*ADAMTSL3*	5'UTR/1st intron	S_Shore	0.557±0.161	0.339±0.103	0.219	5.66E-03	1.43E-08	-0.275
cg05064489	10	102279954	*SEC31B*	TSS1500	S_Shore	0.616±0.086	0.397±0.176	0.218	2.89E-04	2.72E-06	-0.229
cg14792350	1	149983257	*OTUD7B*	TSS1500	S_Shore	0.426±0.126	0.208±0.078	0.218	1.28E-03	2.23E-12	-0.337
cg01550799	7	114561804	*MDFIC*	TSS1500	N_Shore	0.554±0.112	0.336±0.139	0.218	5.66E-04	8.83E-07	-0.239
cg00933603	6	30883001	*VARS2*	5'UTR	S_Shore	0.531±0.079	0.313±0.122	0.218	2.29E-05	4.66E-07	-0.245
cg01250879	22	46660593	*PKDREJ*	TSS1500	S_Shore	0.656±0.133	0.438±0.141	0.218	1.94E-03	4.23E-06	-0.224
cg16107172	19	59023222	*SLC27A5*	1st exon	N_Shelf	0.567±0.079	0.349±0.067	0.218	2.27E-05	2.52E-14	-0.364
cg24370698	1	64058614	*PGM1*	TSS1500	N_Shore	0.351±0.143	0.133±0.057	0.218	3.08E-03	4.50E-09	-0.284

cg04129548	2	227662083	*IRS1*	1st exon	Island	0.524±0.133	0.306±0.086	0.218	1.75E-03	5.56E-07	-0.244
cg05343105	16	29801371	*KIF22*	TSS1500	N_Shore	0.416±0.111	0.199±0.055	0.218	5.88E-04	6.08E-08	-0.263
cg05175337	5	80596475	*ZCCHC9*	TSS1500	N_Shore	0.371±0.108	0.153±0.056	0.218	5.03E-04	2.15E-11	-0.322
cg26234644	17	10634427	*TMEM220*	TSS1500	S_Shore	0.573±0.162	0.356±0.092	0.217	6.13E-03	5.85E-12	-0.331
cg08845721	5	142780693	*NR3C1*	5'UTR/1st intron	N_Shore	0.376±0.097	0.158±0.071	0.217	1.74E-04	6.51E-06	-0.220
cg11142617	1	2324205	*RER1*	5'UTR/1st intron	S_Shore	0.371±0.135	0.154±0.058	0.217	2.25E-03	1.29E-07	-0.257
cg21523719	1	24128335	*GALE*	TSS1500	S_Shore	0.666±0.088	0.449±0.124	0.217	6.99E-05	3.58E-12	-0.334
cg06983052	1	90288099	*LRRC8D*	5'UTR/1st intron	S_Shore	0.411±0.108	0.194±0.047	0.217	5.33E-04	1.34E-10	-0.310
cg12876594	9	35791798	*NPR2*	TSS1500	S_Shore	0.393±0.121	0.176±0.073	0.217	9.98E-04	2.97E-18	-0.411
cg20893838	6	7145478	*RREB1*	5'UTR/1st intron	S_Shelf	0.521±0.048	0.304±0.094	0.217	6.98E-08	2.01E-05	-0.208
cg03269757	2	38602134	*ATL2*	5'UTR/1st intron	N_Shore	0.607±0.148	0.39±0.159	0.217	4.44E-03	1.53E-10	-0.309
cg01587454	8	88886051	*DCAF4L2*	1st exon	N_Shore	0.589±0.145	0.372±0.204	0.217	6.23E-03	1.05E-18	-0.416
cg16563370	17	33775952	*SLFN13*	TSS200	N_Shore	0.532±0.144	0.315±0.133	0.217	3.23E-03	5.22E-10	-0.300
cg15157024	3	58481149	*KCTD6*	5'UTR/1st intron	S_Shelf	0.708±0.07	0.492±0.217	0.217	9.09E-04	3.47E-30	-0.522
cg06972969	10	60935663	*PHYHIPL*	TSS1500	N_Shore	0.339±0.107	0.122±0.044	0.217	5.32E-04	6.75E-09	-0.281
cg14621763	9	8856695	*PTPRD*	5'UTR	N_Shore	0.732±0.078	0.516±0.259	0.216	3.80E-03	2.63E-18	-0.412
cg16855929	1	231561414	*EGLN1*	TSS1500	S_Shelf	0.709±0.095	0.492±0.213	0.216	1.47E-03	2.01E-05	-0.208
cg04064050	6	127663586	*ECHDC1*	5'UTR/1st intron	N_Shore	0.45±0.114	0.233±0.066	0.216	6.80E-04	7.56E-11	-0.314
cg08113027	3	142683329	*PAQR9*	TSS1500	S_Shore	0.549±0.146	0.333±0.141	0.216	3.68E-03	3.56E-08	-0.267
cg02415661	19	16308183	*AP1M1*	TSS1500	N_Shore	0.453±0.13	0.237±0.085	0.216	1.64E-03	3.24E-08	-0.268
cg00063909	2	64877006	*SERTAD2*	5'UTR/1st intron	N_Shelf	0.53±0.07	0.314±0.056	0.216	7.75E-06	7.48E-07	-0.241
cg03083695	9	130659142	*ST6GALNAC6*	5'UTR/1st intron	N_Shore	0.789±0.074	0.573±0.156	0.216	7.22E-05	2.17E-24	-0.473
cg10436546	16	66969837	*CES2*	5'UTR/1st exon	Island	0.464±0.121	0.248±0.066	0.216	1.08E-03	1.82E-23	-0.465
cg08481491	3	125900108	*ALDH1L1*	TSS1500	S_Shore	0.409±0.117	0.194±0.051	0.216	9.61E-04	6.43E-19	-0.419
cg21784940	7	150067807	*REPIN1*	5'UTR	S_Shore	0.474±0.157	0.258±0.15	0.216	6.04E-03	9.49E-10	-0.296
cg04027302	1	236685524	*LGALS8*	5'UTR	N_Shore	0.756±0.079	0.54±0.174	0.216	2.16E-04	2.76E-07	-0.250
cg10508783	2	165478953	*GRB14*	TSS1500	S_Shore	0.544±0.136	0.328±0.117	0.216	2.19E-03	4.87E-06	-0.223
cg16474952	16	16315534	*ABCC6*	5'UTR	N_Shore	0.554±0.11	0.339±0.123	0.216	4.62E-04	2.60E-20	-0.434
cg16198692	5	115152835	*CDO1*	TSS1500	S_Shore	0.393±0.146	0.178±0.058	0.215	3.70E-03	2.86E-21	-0.443
cg01042334	8	145114994	*OPLAH*	5'UTR/1st intron	S_Shore	0.516±0.159	0.3±0.065	0.215	5.92E-03	2.94E-13	-0.349
cg18061319	10	71891928	*AIFM2*	5'UTR/1st intron	N_Shore	0.54±0.071	0.325±0.063	0.215	7.74E-06	2.81E-06	-0.228
cg00992048	1	1695585	*NADK*	5'UTR	S_Shelf	0.537±0.056	0.321±0.089	0.215	2.60E-07	2.78E-09	-0.288
cg25135457	17	40715244	*COASY*	1st exon	N_Shelf	0.542±0.139	0.326±0.082	0.215	2.65E-03	1.45E-06	-0.235
cg05544413	6	127663627	*ECHDC1*	5'UTR/1st intron	N_Shore	0.439±0.119	0.224±0.069	0.215	9.82E-04	8.80E-07	-0.239
cg10903281	3	11036746	*SLC6A1*	5'UTR/1st intron	S_Shore	0.592±0.151	0.377±0.142	0.215	4.79E-03	1.37E-39	-0.587
cg20576322	4	85886384	*WDFY3*	5'UTR/1st intron	N_Shore	0.607±0.199	0.392±0.193	0.215	2.32E-02	2.34E-06	-0.230
cg05913474	8	144815204	*FAM83H*	5'UTR/1st intron	Island	0.512±0.159	0.297±0.063	0.215	5.97E-03	1.01E-25	-0.485
cg25182621	3	184097440	*CHRD*	TSS1500	N_Shore	0.554±0.115	0.339±0.103	0.215	6.60E-04	2.77E-06	-0.229
cg27029623	4	39368603	*RFC1*	TSS1500	S_Shore	0.579±0.104	0.365±0.07	0.215	3.45E-04	1.16E-11	-0.326
cg15255455	19	4534986	*PLIN5*	5'UTR/1st intron	N_Shore	0.382±0.104	0.168±0.072	0.215	3.44E-04	1.44E-12	-0.339
cg24812167	9	139293358	*SNAPC4*	TSS1500	N_Shore	0.476±0.125	0.262±0.087	0.214	1.31E-03	1.69E-05	-0.210
cg14123992	19	45407868	*APOE*	TSS1500	N_Shelf	0.471±0.12	0.257±0.097	0.214	9.68E-04	4.57E-09	-0.284
cg21090344	17	6948023	*SLC16A11*	TSS1500	S_Shore	0.489±0.126	0.275±0.099	0.214	1.32E-03	1.85E-10	-0.307
cg02304481	2	97652129	*FAM178B*	5'UTR/1st exon	Island	0.723±0.104	0.509±0.158	0.214	5.83E-04	3.50E-11	-0.319
cg26493224	13	52980671	*THSD1*	TSS200	S_Shore	0.365±0.132	0.151±0.083	0.214	1.90E-03	1.86E-08	-0.273
cg12524553	20	32952045	*ITCH*	5'UTR/1st intron	S_Shore	0.394±0.126	0.18±0.078	0.214	1.44E-03	1.41E-05	-0.212
cg07230486	7	11012873	*PHF14*	TSS1500	N_Shore	0.482±0.159	0.268±0.09	0.214	6.06E-03	3.63E-10	-0.303
cg08036764	22	24891220	*UPB1*	TSS200	Island	0.625±0.125	0.412±0.074	0.214	1.36E-03	2.85E-26	-0.490
cg25995289	19	30207352	*C19orf12*	TSS1500	S_Shore	0.606±0.076	0.393±0.145	0.214	5.62E-05	1.61E-09	-0.292
cg13393830	19	52391605	*ZNF577*	TSS1500	S_Shore	0.673±0.129	0.46±0.196	0.214	3.60E-03	7.10E-33	-0.542
cg26033529	2	20425981	*SDC1*	TSS1500	S_Shore	0.524±0.11	0.311±0.075	0.213	5.47E-04	4.05E-11	-0.318
cg08180572	16	28985143	*SPNS1*	TSS1500	N_Shore	0.675±0.127	0.462±0.225	0.213	5.46E-03	1.18E-05	-0.214
cg05005077	1	247276966	*C1orf229*	TSS1500	N_Shelf	0.451±0.124	0.238±0.078	0.213	1.34E-03	3.15E-06	-0.227
cg23629150	8	144416404	*TOP1MT*	5'UTR	N_Shore	0.586±0.164	0.373±0.158	0.213	8.69E-03	6.23E-12	-0.330
cg15291090	13	36788776	*SOHLH2*	TSS200	Island	0.73±0.119	0.517±0.158	0.213	1.35E-03	8.22E-06	-0.218
cg17519037	17	36452075	*MRPL45*	TSS1500	N_Shore	0.436±0.113	0.223±0.071	0.213	7.04E-04	7.72E-06	-0.218
cg02006107	8	80943347	*MRPS28*	TSS1500	S_Shore	0.537±0.138	0.325±0.094	0.213	2.65E-03	1.36E-06	-0.235
cg22158648	18	20713759	*CABLES1*	TSS1500	N_Shore	0.434±0.096	0.222±0.078	0.213	1.71E-04	8.67E-11	-0.313
cg23884175	11	76495749	*TSKU*	5'UTR/1st intron	S_Shore	0.401±0.092	0.188±0.058	0.213	1.55E-04	1.33E-18	-0.415
cg12435725	3	58293450	*RPP14*	5'UTR/1st intron	S_Shore	0.404±0.115	0.191±0.048	0.213	9.71E-04	5.50E-07	-0.244
cg00501904	1	226001675	*EPHX1*	5'UTR/1st intron	S_Shelf	0.368±0.117	0.155±0.06	0.212	1.00E-03	5.40E-20	-0.430
cg19489188	19	4183268	*ANKRD24*	TSS200	S_Shore	0.495±0.125	0.282±0.08	0.212	1.44E-03	1.37E-06	-0.235
cg26916966	17	40274524	*HSPB9*	TSS1500	Island	0.587±0.17	0.375±0.132	0.212	9.72E-03	9.53E-19	-0.417
cg26916966	17	40274524	*KAT2A*	TSS1500	Island	0.587±0.17	0.375±0.132	0.212	9.72E-03	4.07E-05	-0.201
cg01899937	4	185723505	*ACSL1*	5'UTR	N_Shore	0.616±0.125	0.404±0.12	0.212	1.37E-03	7.72E-23	-0.459
cg07346603	5	169659751	*C5orf58*	TSS1500	N_Shore	0.789±0.123	0.578±0.163	0.211	1.87E-03	4.55E-22	-0.451
cg26761618	11	72928926	*P2RY2*	TSS1500	N_Shore	0.495±0.152	0.284±0.1	0.211	4.96E-03	4.48E-23	-0.461
cg22601917	1	9294274	*H6PD*	TSS1500	N_Shore	0.415±0.053	0.204±0.07	0.211	1.13E-07	3.08E-22	-0.453
cg00636737	20	62695569	*TCEA2*	5'UTR	S_Shore	0.455±0.097	0.243±0.085	0.211	1.81E-04	2.94E-10	-0.304
cg11556489	6	30178724	*TRIM26*	5'UTR/1st intron	N_Shelf	0.449±0.095	0.238±0.067	0.211	1.83E-04	5.03E-16	-0.385
cg12122057	11	12133015	*MICAL2*	5'UTR/1st intron	Island	0.593±0.108	0.382±0.116	0.211	4.84E-04	1.86E-11	-0.323
cg06849167	11	61449886	*DAGLA*	5'UTR/1st intron	S_Shore	0.588±0.062	0.378±0.147	0.211	4.45E-05	5.09E-06	-0.223
cg07692489	16	8960972	*CARHSP1*	5'UTR	N_Shore	0.391±0.111	0.18±0.084	0.211	5.92E-04	1.02E-07	-0.259
cg02349264	16	8960833	*CARHSP1*	5'UTR	N_Shore	0.372±0.107	0.161±0.071	0.211	5.06E-04	3.12E-07	-0.249
cg24808901	1	95698954	*RWDD3*	TSS1500	N_Shore	0.367±0.116	0.156±0.064	0.210	9.85E-04	7.68E-06	-0.218
cg21320567	8	67975880	*COPS5*	TSS1500	N_Shore	0.454±0.113	0.244±0.12	0.210	7.16E-04	5.08E-09	-0.283
cg19026231	17	73761710	*GALK1*	TSS1500	S_Shore	0.411±0.13	0.201±0.067	0.210	2.05E-03	8.80E-11	-0.312
cg14757492	19	19029194	*DDX49*	TSS1500	N_Shore	0.573±0.152	0.363±0.15	0.210	6.00E-03	2.45E-15	-0.377
cg07838483	19	30202712	*C19orf12*	5'UTR/1st intron	N_Shelf	0.381±0.121	0.171±0.042	0.210	1.51E-03	9.40E-07	-0.239
cg05694095	5	169659745	*C5orf58*	TSS1500	N_Shore	0.765±0.106	0.555±0.213	0.210	2.70E-03	9.22E-18	-0.406
cg07896832	8	67975874	*COPS5*	TSS1500	N_Shore	0.438±0.117	0.228±0.126	0.210	9.96E-04	4.51E-09	-0.284
cg01303236	5	73934842	*ENC1*	5'UTR/1st intron	N_Shore	0.661±0.113	0.451±0.225	0.210	4.32E-03	9.68E-16	-0.382
cg03785076	2	241936915	*SNED1*	TSS1500	N_Shore	0.477±0.119	0.268±0.145	0.210	1.34E-03	2.54E-10	-0.305
cg18160691	11	68081686	*LRP5*	5'UTR/1st intron	S_Shore	0.367±0.111	0.158±0.07	0.210	7.09E-04	3.68E-06	-0.226
cg20400248	8	9005257	*PPP1R3B*	5'UTR/1st intron	N_Shelf	0.617±0.113	0.407±0.173	0.210	1.49E-03	1.47E-17	-0.403
cg15922246	7	150778803	*FASTK*	TSS1500	Island	0.484±0.104	0.274±0.089	0.209	3.49E-04	1.17E-05	-0.214
cg10444806	17	38084428	*ORMDL3*	TSS1500	S_Shore	0.371±0.119	0.162±0.054	0.209	1.30E-03	4.30E-08	-0.266

cg16402814	11	915227	*CHID1*	TSS200	S_Shelf	0.463±0.1	0.253±0.124	0.209	2.98E-04	1.85E-09	-0.291
cg22989379	20	33462683	*ACSS2*	TSS200	N_Shore	0.58±0.156	0.371±0.095	0.209	6.22E-03	1.21E-11	-0.326
cg10081469	14	61748336	*TMEM30B*	5'UTR/1st exon	S_Shore	0.393±0.109	0.184±0.08	0.209	5.77E-04	1.11E-11	-0.326
cg11836372	10	123353822	*FGFR2*	5'UTR/1st intron	N_Shelf	0.616±0.149	0.407±0.205	0.209	9.03E-03	1.83E-08	-0.273
cg01617211	16	90040387	*CENPBD1*	TSS1500	S_Shore	0.49±0.082	0.282±0.057	0.209	6.27E-05	1.05E-11	-0.327
cg20844262	20	62367372	*LIME1*	5'UTR/1st exon	N_Shore	0.838±0.028	0.629±0.167	0.209	5.90E-05	1.90E-29	-0.516
cg08224159	6	151646312	*AKAP12*	TSS1500	N_Shore	0.514±0.164	0.305±0.107	0.209	8.19E-03	2.35E-05	-0.207
cg00319334	11	2397523	*CD81*	5'UTR/1st exon	N_Shore	0.561±0.16	0.353±0.167	0.209	8.97E-03	1.54E-10	-0.309
cg17575915	22	37414442	*MPST*	TSS1500	Island	0.548±0.079	0.34±0.07	0.208	3.25E-05	4.35E-07	-0.246
cg03962678	11	118398094	*TTC36*	TSS200	N_Shelf	0.381±0.077	0.173±0.049	0.208	4.45E-05	2.65E-09	-0.288
cg07360805	20	36148457	*BLCAP*	5'UTR	N_Shelf	0.52±0.144	0.312±0.146	0.208	4.52E-03	1.17E-13	-0.355
cg20808613	15	89168305	*AEN*	5'UTR/1st intron	S_Shelf	0.539±0.134	0.331±0.224	0.207	7.88E-03	9.69E-08	-0.259
cg14991769	7	150497157	*TMEM176A*	TSS1500	Island	0.321±0.093	0.113±0.067	0.207	1.82E-04	1.30E-08	-0.276
cg14991769	7	150497157	*TMEM176B*	5'UTR/1st intron	Island	0.321±0.093	0.113±0.067	0.207	1.82E-04	2.54E-08	-0.270
cg03880355	5	74162880	*FAM169A*	TSS1500	S_Shore	0.373±0.125	0.165±0.056	0.207	1.81E-03	1.51E-13	-0.353
cg07058998	18	52625365	*CCDC68*	5'UTR/1st intron	N_Shore	0.513±0.126	0.306±0.134	0.207	1.89E-03	4.87E-10	-0.300
cg22151881	11	68082621	*LRP5*	5'UTR/1st intron	S_Shore	0.359±0.117	0.152±0.053	0.207	1.25E-03	1.70E-06	-0.233
cg21714557	13	113776873	*F10*	TSS1500	S_Shelf	0.324±0.134	0.117±0.061	0.207	2.85E-03	1.67E-05	-0.210
cg10710439	19	38183976	*ZNF781*	TSS1500	S_Shore	0.508±0.055	0.301±0.126	0.207	5.13E-06	2.63E-05	-0.205
cg05934698	12	121416390	*HNF1A*	5'UTR/1st exon	N_Shore	0.493±0.119	0.286±0.07	0.207	1.22E-03	3.46E-24	-0.471
cg01234517	7	150021553	*LRRC61*	5'UTR/1st intron	S_Shore	0.532±0.125	0.325±0.072	0.207	1.75E-03	6.02E-10	-0.299
cg12912293	1	150849805	*ARNT*	TSS1500	S_Shore	0.548±0.14	0.341±0.193	0.207	6.40E-03	2.08E-10	-0.306
cg01596520	19	14225029	*PRKACA*	TSS200	N_Shelf	0.731±0.148	0.524±0.199	0.207	8.87E-03	1.61E-05	-0.211
cg27021181	10	5488346	*NET1*	TSS200	N_Shore	0.33±0.144	0.124±0.062	0.207	4.39E-03	1.44E-08	-0.275
cg08063051	16	1428706	*UNKL*	5'UTR/1st intron	N_Shore	0.494±0.135	0.287±0.154	0.207	6.07E-03	1.03E-20	-0.438
cg20305595	1	9293833	*H6PD*	TSS1500	N_Shore	0.509±0.066	0.302±0.057	0.207	6.46E-06	2.62E-24	-0.473
cg06906869	13	52734154	*NEK3*	TSS1500	S_Shore	0.626±0.091	0.419±0.157	0.207	3.62E-04	1.85E-29	-0.516
cg00716257	14	75897417	*JDP2*	5'UTR/1st intron	S_Shore	0.748±0.102	0.542±0.243	0.206	5.49E-03	7.09E-22	-0.449
cg16124934	14	75389480	*RPS6KL1*	TSS1500	N_Shore	0.521±0.115	0.315±0.141	0.206	1.11E-03	3.06E-40	-0.591
cg17818912	5	68787696	*OCLN*	TSS1500	N_Shore	0.379±0.128	0.173±0.059	0.206	2.19E-03	5.09E-06	-0.223
cg14032742	1	229409358	*RAB4A*	5'UTR/1st intron	S_Shelf	0.563±0.131	0.357±0.091	0.206	2.30E-03	1.61E-21	-0.446
cg00368356	19	39395717	*NFKBIB*	5'UTR	N_Shore	0.527±0.157	0.321±0.101	0.206	7.05E-03	1.07E-09	-0.295
cg02026246	11	22687589	*GAS2*	TSS1500	N_Shore	0.609±0.161	0.403±0.161	0.206	9.77E-03	6.44E-08	-0.262
cg25612480	8	48920460	*UBE2V2*	TSS1500	N_Shore	0.425±0.099	0.219±0.107	0.206	2.77E-04	4.24E-06	-0.224
cg04756491	14	23385384	*RBM23*	5'UTR	N_Shelf	0.562±0.116	0.357±0.156	0.205	1.52E-03	9.72E-06	-0.216
cg16363146	11	22689281	*GAS2*	5'UTR/1st intron	S_Shore	0.656±0.161	0.451±0.21	0.205	1.44E-02	5.93E-15	-0.372
cg01341572	12	121416512	*HNF1A*	5'UTR/1st exon	Island	0.537±0.125	0.332±0.083	0.205	1.75E-03	4.44E-21	-0.441
cg21874832	2	97652249	*FAM178B*	5'UTR/1st exon	Island	0.72±0.126	0.514±0.194	0.205	4.34E-03	1.71E-13	-0.352
cg10222925	8	88885186	*DCAF4L2*	1st exon	N_Shore	0.381±0.076	0.176±0.056	0.205	3.62E-05	9.24E-11	-0.312
cg01933228	1	100316637	*AGL*	5'UTR/1st exon	S_Shore	0.363±0.104	0.158±0.066	0.205	5.12E-04	1.75E-13	-0.352
cg07880854	2	112895559	*FBLN7*	TSS1500	N_Shore	0.452±0.168	0.248±0.15	0.205	1.15E-02	1.57E-05	-0.211
cg18711369	17	38081186	*ORMDL3*	5'UTR	N_Shelf	0.416±0.107	0.211±0.056	0.205	6.95E-04	1.73E-15	-0.379
cg11819637	3	184095505	*THPO*	5'UTR	N_Shelf	0.425±0.123	0.22±0.046	0.205	1.91E-03	8.26E-27	-0.495
cg01656133	11	17566772	*USH1C*	TSS1500	S_Shore	0.847±0.068	0.642±0.161	0.205	1.31E-04	1.47E-05	-0.212
cg07141002	22	38201690	*H1F0*	1st exon	Island	0.339±0.097	0.134±0.06	0.205	3.20E-04	1.20E-14	-0.368
cg09831081	21	47647568	*LSS*	5'UTR	N_Shore	0.654±0.135	0.449±0.171	0.205	4.41E-03	3.97E-20	-0.432
cg12911952	11	2924523	*SLC22A18*	5'UTR	S_Shore	0.525±0.123	0.32±0.078	0.204	1.63E-03	5.67E-06	-0.221
cg17852385	15	75019188	*CYP1A1*	TSS1500	Island	0.326±0.134	0.122±0.038	0.204	3.22E-03	9.85E-06	-0.216
cg19047340	20	33873068	*EIF6*	TSS1500	S_Shore	0.486±0.093	0.282±0.061	0.204	2.22E-04	6.37E-09	-0.281
cg03594078	8	22131675	*PIWIL2*	TSS1500	N_Shore	0.398±0.099	0.194±0.07	0.204	3.40E-04	7.06E-07	-0.241
cg08387551	5	72417180	*TMEM171*	5'UTR/1st intron	S_Shore	0.664±0.116	0.46±0.204	0.204	3.78E-03	4.38E-09	-0.284
cg03890680	1	227504721	*CDC42BPA*	1st exon	N_Shore	0.324±0.098	0.12±0.062	0.204	3.47E-04	3.62E-18	-0.410
cg15481791	1	203829890	*SNRPE*	TSS1500	N_Shore	0.417±0.106	0.212±0.091	0.204	5.15E-04	9.20E-06	-0.217
cg13008631	2	227663034	*IRS1*	1st exon	Island	0.498±0.103	0.294±0.079	0.204	4.41E-04	8.64E-12	-0.328
cg25017777	1	9298817	*H6PD*	5'UTR/1st intron	S_Shelf	0.451±0.135	0.247±0.067	0.204	3.11E-03	4.91E-07	-0.245
cg09289251	16	2207865	*TRAF7*	5'UTR/1st intron	S_Shore	0.477±0.12	0.273±0.056	0.204	1.59E-03	5.54E-07	-0.244
cg02585931	22	38219283	*GALR3*	TSS200	N_Shore	0.578±0.119	0.375±0.141	0.204	1.59E-03	2.56E-07	-0.251
cg08005460	17	38473382	*RARA*	5'UTR/1st intron	S_Shore	0.347±0.115	0.143±0.068	0.204	1.11E-03	5.29E-06	-0.222
cg08472797	11	2925570	*SLC22A18AS*	TSS1500	S_Shore	0.524±0.137	0.321±0.091	0.204	3.39E-03	6.43E-09	-0.281
cg02801114	6	125476162	*TPD52L1*	5'UTR/1st intron	S_Shore	0.468±0.184	0.265±0.06	0.203	1.65E-02	6.89E-18	-0.407
cg16540789	16	75150784	*LDHD*	TSS200	S_Shore	0.343±0.106	0.14±0.048	0.203	7.58E-04	1.53E-11	-0.324
cg09899215	16	75150799	*LDHD*	TSS200	S_Shore	0.341±0.101	0.138±0.055	0.203	4.83E-04	1.35E-09	-0.293
cg02733918	20	47801453	*STAU1*	5'UTR	N_Shelf	0.406±0.109	0.203±0.055	0.203	8.53E-04	2.19E-05	-0.207
cg08633074	16	11837719	*TXNDC11*	TSS1500	S_Shore	0.482±0.102	0.279±0.07	0.203	4.49E-04	5.51E-08	-0.264
cg01963374	11	704987	*EPS8L2*	TSS1500	N_Shore	0.404±0.098	0.201±0.073	0.202	3.06E-04	1.18E-05	-0.214
cg04781305	10	35624429	*CCNY*	5'UTR	N_Shore	0.409±0.133	0.207±0.054	0.202	3.23E-03	1.11E-07	-0.258
cg02898721	17	38473222	*RARA*	5'UTR/1st intron	S_Shore	0.436±0.108	0.234±0.082	0.202	6.75E-04	4.28E-07	-0.246
cg05053220	16	56457177	*AMFR*	5'UTR/1st intron	N_Shore	0.512±0.123	0.311±0.13	0.202	1.90E-03	5.51E-08	-0.264
cg08202720	2	239196219	*PER2*	5'UTR/1st intron	N_Shore	0.657±0.132	0.455±0.166	0.202	4.18E-03	7.43E-16	-0.383
cg05414254	16	53535931	*AKTIP*	5'UTR/1st intron	N_Shore	0.452±0.131	0.25±0.096	0.202	2.60E-03	3.44E-10	-0.303
cg09424348	6	30881549	*VARS2*	TSS1500	Island	0.33±0.105	0.129±0.063	0.201	6.14E-04	1.26E-09	-0.293
cg25383093	6	83902161	*RWDD2A*	TSS1500	N_Shore	0.427±0.129	0.226±0.092	0.201	2.48E-03	3.20E-07	-0.249
cg22163535	18	6411387	*L3MBTL4*	5'UTR/1st intron	N_Shelf	0.641±0.073	0.44±0.203	0.201	1.12E-03	2.18E-08	-0.271
cg27181716	3	133467304	*TF*	5'UTR	S_Shore	0.504±0.099	0.303±0.058	0.201	4.44E-04	2.26E-12	-0.337
cg23956760	19	19178708	*SLC25A42*	5'UTR/1st intron	S_Shelf	0.755±0.064	0.554±0.199	0.201	8.22E-04	1.64E-10	-0.308
cg09118420	22	30477270	*HORMAD2*	5'UTR	S_Shore	0.758±0.133	0.557±0.24	0.201	1.20E-02	3.74E-43	-0.609
cg10287137	11	72929054	*P2RY2*	TSS1500	Island	0.469±0.101	0.268±0.068	0.201	4.49E-04	6.28E-08	-0.263
cg04439218	5	37839407	*GDNF*	5'UTR/1st intron	Island	0.356±0.18	0.155±0.106	0.201	1.64E-02	6.84E-07	-0.242
cg04921771	1	40349687	*TRIT1*	TSS1500	S_Shore	0.486±0.075	0.286±0.121	0.200	4.29E-05	4.65E-06	-0.223
cg20852557	3	184035519	*EIF4G1*	5'UTR	S_Shelf	0.525±0.146	0.325±0.114	0.200	5.46E-03	5.08E-10	-0.300
cg07787240	1	9130576	*SLC2A5*	5'UTR/1st intron	N_Shore	0.528±0.107	0.327±0.075	0.200	6.86E-04	3.03E-06	-0.228
cg02719634	11	2924899	*SLC22A18AS*	5'UTR/1st exon	S_Shore	0.539±0.145	0.338±0.111	0.200	5.22E-03	8.45E-23	-0.458
cg11193767	4	83930905	*LIN54*	5'UTR/1st intron	N_Shelf	0.503±0.127	0.302±0.086	0.200	2.28E-03	1.54E-06	-0.234
cg10000250	17	80023016	*DUS1L*	5'UTR/1st intron	Island	0.497±0.084	0.297±0.096	0.200	7.30E-05	5.97E-08	-0.263
cg10668614	11	915163	*CHID1*	TSS200	S_Shelf	0.448±0.087	0.248±0.125	0.200	1.50E-04	1.04E-09	-0.295
cg13580783	1	24128567	*GALE*	TSS1500	S_Shore	0.422±0.105	0.222±0.07	0.200	6.22E-04	5.94E-13	-0.345
cg00516966	17	19651653	*ALDH3A1*	5'UTR/1st exon	S_Shelf	0.453±0.109	0.253±0.083	0.200	7.71E-04	6.38E-07	-0.242

cg07859880	19	39323060	*ECH1*	TSS1500	S_Shore	0.316±0.089	0.116±0.048	0.200	2.29E-04	6.20E-09	-0.281
cg07124366	3	10361044	*SEC13*	5'UTR/1st intron	N_Shore	0.434±0.145	0.234±0.088	0.200	5.33E-03	7.71E-06	-0.218
cg08190844	16	2526203	*TBC1D24*	5'UTR/1st intron	S_Shore	0.521±0.15	0.321±0.131	0.200	6.78E-03	1.33E-10	-0.310
cg07765167	17	36451845	*MRPL45*	TSS1500	N_Shore	0.426±0.107	0.226±0.069	0.200	7.32E-04	9.22E-06	-0.217
cg27494647	7	150038898	*RARRES2*	TSS200	Island	0.326±0.107	0.126±0.047	0.200	9.14E-04	4.12E-06	-0.225
cg11120551	1	146713996	*CHD1L*	5'UTR	N_Shore	0.335±0.125	0.135±0.049	0.200	2.38E-03	3.04E-05	-0.204
cg02560408	10	62757486	*RHOBTB1*	5'UTR/1st intron	N_Shelf	0.73±0.102	0.53±0.214	0.200	3.70E-03	1.51E-06	-0.234
cg26578274	15	78832312	*PSMA4*	TSS1500	N_Shore	0.6±0.091	0.401±0.224	0.200	3.56E-03	1.23E-05	-0.214
cg06568323	13	100745507	*PCCA*	5'UTR/1st intron	S_Shelf	0.752±0.097	0.553±0.192	0.199	1.83E-03	9.91E-15	-0.369
cg27203309	17	19550687	*ALDH3A2*	TSS1500	N_Shore	0.398±0.105	0.198±0.052	0.199	7.77E-04	9.70E-08	-0.259
cg20304988	7	29237766	*CHN2*	5'UTR/1st intron	S_Shelf	0.676±0.112	0.477±0.166	0.199	1.89E-03	6.64E-24	-0.469
cg19884658	1	6664268	*KLHL21*	TSS1500	S_Shore	0.33±0.107	0.131±0.052	0.199	8.64E-04	5.24E-10	-0.300
cg27367170	10	5488628	*NET1*	1st exon	Island	0.348±0.122	0.149±0.076	0.199	1.91E-03	1.32E-07	-0.256
cg16937168	2	241936844	*SNED1*	TSS1500	N_Shore	0.505±0.113	0.306±0.205	0.199	4.26E-03	1.23E-15	-0.380
cg01730857	8	144912074	*PUF60*	TSS1500	Island	0.441±0.133	0.242±0.069	0.199	3.35E-03	2.86E-14	-0.363
cg02306936	6	30881316	*VARS2*	TSS1500	N_Shore	0.418±0.111	0.219±0.073	0.199	9.50E-04	1.78E-09	-0.291
cg26306893	20	37074908	*SNHG11*	TSS1500	N_Shore	0.345±0.09	0.146±0.046	0.198	2.63E-04	1.80E-08	-0.273
cg16592832	22	24891141	*UPB1*	TSS200	Island	0.344±0.105	0.146±0.052	0.198	7.60E-04	1.03E-11	-0.327
cg14996810	12	121416501	*HNF1A*	5'UTR/1st exon	Island	0.555±0.134	0.357±0.074	0.198	3.47E-03	4.90E-24	-0.470
cg11669516	2	217501092	*IGFBP2*	5'UTR/1st intron	S_Shelf	0.651±0.142	0.454±0.273	0.198	2.37E-02	4.14E-30	-0.521
cg19324627	11	116708329	*APOA1*	5'UTR/1st exon	S_Shore	0.454±0.123	0.257±0.058	0.198	2.18E-03	7.59E-06	-0.219
cg17084863	1	162530364	*UAP1*	TSS1500	N_Shore	0.506±0.114	0.309±0.117	0.197	1.26E-03	3.21E-11	-0.319
cg06246941	15	45671708	*GATM*	5'UTR	S_Shore	0.706±0.1	0.508±0.183	0.197	1.76E-03	2.83E-22	-0.453
cg15049101	10	123353889	*FGFR2*	5'UTR/1st intron	N_Shelf	0.517±0.15	0.319±0.196	0.197	1.20E-02	3.31E-06	-0.227
cg09573761	22	38219336	*GALR3*	TSS200	N_Shore	0.668±0.133	0.471±0.157	0.197	4.64E-03	4.97E-07	-0.245
cg19618279	17	40715228	*COASY*	1st exon	N_Shelf	0.535±0.108	0.337±0.071	0.197	8.77E-04	1.31E-05	-0.213
cg03910196	16	2256384	*MLST8*	5'UTR	S_Shore	0.526±0.084	0.328±0.113	0.197	1.12E-04	2.49E-05	-0.206
cg02711665	8	11664728	*FDFT1*	5'UTR	N_Shore	0.684±0.131	0.487±0.174	0.197	5.20E-03	1.52E-22	-0.456
cg26841483	12	81332540	*LIN7A*	TSS1500	S_Shore	0.492±0.149	0.296±0.154	0.196	8.35E-03	8.37E-07	-0.240
cg15350036	7	86973677	*CROT*	TSS1500	N_Shore	0.575±0.148	0.379±0.218	0.196	1.47E-02	2.16E-08	-0.271
cg24324903	19	35629275	*FXYD1*	TSS1500	N_Shelf	0.649±0.089	0.453±0.125	0.196	2.19E-04	1.04E-14	-0.369
cg03305454	3	42843817	*HIGD1A*	5'UTR/1st intron	N_Shore	0.816±0.107	0.62±0.214	0.196	5.01E-03	1.72E-23	-0.465
cg17498424	22	42528123	*CYP2D6*	TSS1500	S_Shelf	0.761±0.057	0.565±0.138	0.196	3.06E-05	2.51E-12	-0.336
cg06483820	17	55167149	*AKAP1*	5'UTR	S_Shelf	0.771±0.057	0.575±0.16	0.196	1.27E-04	2.23E-14	-0.364
cg13494428	1	19639494	*PQLC2*	5'UTR	S_Shore	0.452±0.099	0.257±0.063	0.195	5.15E-04	2.23E-08	-0.271
cg13494428	1	19639494	*AKR7A2*	TSS1500	S_Shore	0.452±0.099	0.257±0.063	0.195	5.15E-04	3.99E-07	-0.247
cg02909320	3	45879990	*LZTFL1*	5'UTR	N_Shelf	0.762±0.072	0.567±0.223	0.195	2.82E-03	6.92E-07	-0.242
cg06653796	20	62367805	*LIME1*	5'UTR/1st intron	N_Shore	0.482±0.115	0.286±0.182	0.195	3.45E-03	1.45E-21	-0.446
cg10581876	1	205645246	*SLC45A3*	5'UTR/1st intron	N_Shelf	0.544±0.115	0.349±0.101	0.195	1.40E-03	1.25E-05	-0.213
cg25152404	1	225999183	*EPHX1*	5'UTR/1st intron	S_Shore	0.364±0.102	0.169±0.06	0.195	6.47E-04	1.06E-37	-0.575
cg05739419	12	53571394	*CSAD*	5'UTR	N_Shelf	0.533±0.128	0.339±0.051	0.195	3.13E-03	1.70E-05	-0.210
cg08880849	17	40274728	*HSPB9*	TSS200	Island	0.409±0.15	0.214±0.123	0.195	7.75E-03	7.81E-14	-0.357
cg13178361	1	162532502	*UAP1*	5'UTR	S_Shore	0.378±0.127	0.183±0.076	0.195	2.80E-03	1.72E-08	-0.273
cg11787780	5	110844727	*STARD4*	5'UTR/1st intron	N_Shelf	0.609±0.148	0.415±0.162	0.195	8.98E-03	3.65E-07	-0.247
cg00345314	8	67624394	*SGK3*	TSS1500	N_Shore	0.305±0.132	0.11±0.045	0.195	3.91E-03	4.01E-12	-0.333
cg08898569	11	125760891	*HYLS1*	5'UTR/1st intron	S_Shelf	0.599±0.1	0.405±0.152	0.194	9.94E-04	1.05E-05	-0.215
cg05385434	1	225998870	*EPHX1*	5'UTR/1st intron	S_Shore	0.328±0.108	0.134±0.073	0.194	9.47E-04	2.28E-11	-0.322
cg17616192	17	80009015	*GPS1*	TSS1500	N_Shelf	0.543±0.167	0.35±0.116	0.193	1.39E-02	1.65E-05	-0.210
cg26688911	20	39969536	*LPIN3*	1st exon	S_Shore	0.318±0.087	0.124±0.067	0.193	1.65E-04	9.46E-11	-0.312
cg04654716	5	74162924	*FAM169A*	TSS1500	S_Shore	0.488±0.114	0.294±0.085	0.193	1.35E-03	2.59E-18	-0.412
cg08596055	20	60722646	*SS18L1*	5'UTR/1st intron	S_Shelf	0.58±0.066	0.387±0.149	0.193	1.24E-04	1.83E-35	-0.561
cg14559422	1	26148138	*LOC646471*	1st exon	S_Shore	0.579±0.147	0.386±0.104	0.193	6.98E-03	2.88E-05	-0.205
cg06928993	19	49313421	*BCAT2*	5'UTR/1st intron	N_Shore	0.578±0.106	0.385±0.197	0.193	3.60E-03	7.44E-11	-0.314
cg10613381	22	24890330	*UPB1*	TSS1500	N_Shore	0.445±0.111	0.252±0.051	0.193	1.38E-03	1.04E-11	-0.327
cg07816556	6	26017280	*HIST1H1A*	1st exon/3'UTR	N_Shelf	0.444±0.14	0.251±0.2	0.193	1.09E-02	3.45E-11	-0.319
cg18924816	14	103388762	*AMN*	TSS1500	N_Shore	0.512±0.11	0.319±0.112	0.193	1.14E-03	1.42E-11	-0.325
cg17800870	1	182362757	*GLUL*	TSS1500	S_Shore	0.847±0.032	0.654±0.255	0.193	5.40E-03	7.65E-73	-0.741
cg13160251	15	56286575	*NEDD4*	TSS1500	S_Shore	0.333±0.123	0.14±0.071	0.193	2.46E-03	7.71E-08	-0.261
cg08764927	1	7844895	*PER3*	5'UTR	Island	0.556±0.109	0.364±0.148	0.193	1.59E-03	2.38E-16	-0.389
cg09037712	21	38362754	*HLCS*	TSS200	Island	0.392±0.121	0.2±0.086	0.193	2.14E-03	1.85E-07	-0.253
cg19226017	6	35697185	*FKBP5*	TSS1500	N_Shore	0.695±0.079	0.502±0.163	0.193	4.75E-04	7.02E-08	-0.262
cg07359991	16	30995944	*HSD3B7*	TSS1500	S_Shelf	0.618±0.099	0.425±0.096	0.193	4.69E-04	1.03E-08	-0.277
cg24129600	8	42395557	*SLC20A2*	5'UTR/1st intron	N_Shore	0.421±0.166	0.228±0.084	0.193	1.34E-02	5.72E-14	-0.359
cg02157083	11	116662800	*APOA5*	5'UTR/1st intron	S_Shelf	0.696±0.044	0.503±0.181	0.192	3.73E-04	6.80E-38	-0.577
cg01434349	11	118900337	*SLC37A4*	5'UTR	N_Shore	0.402±0.118	0.21±0.052	0.192	2.10E-03	1.79E-05	-0.210
cg04849878	5	176516112	*FGFR4*	5'UTR/1st intron	S_Shore	0.412±0.127	0.22±0.06	0.192	3.23E-03	1.27E-08	-0.276
cg17339327	6	31939025	*STK19*	1st exon	N_Shore	0.516±0.136	0.325±0.107	0.192	4.69E-03	2.46E-06	-0.230
cg22332037	1	153951279	*JTB*	TSS1500	S_Shore	0.569±0.186	0.377±0.167	0.192	2.72E-02	1.20E-07	-0.257
cg11157127	6	143998869	*PHACTR2*	TSS1500	N_Shore	0.33±0.157	0.138±0.108	0.192	1.05E-02	2.60E-06	-0.229
cg16208084	17	40274703	*HSPB9*	TSS200	Island	0.413±0.163	0.221±0.13	0.192	1.34E-02	3.03E-15	-0.376
cg06346081	1	42921584	*ZMYND12*	1st exon	N_Shore	0.407±0.155	0.216±0.151	0.192	1.16E-02	5.33E-27	-0.496
cg15080939	6	30881560	*VARS2*	TSS1500	Island	0.4±0.101	0.208±0.072	0.192	6.02E-04	4.11E-09	-0.285
cg25987102	11	116709735	*APOA1*	TSS1500	S_Shelf	0.547±0.159	0.355±0.109	0.192	1.13E-02	2.64E-05	-0.205
cg01620611	17	71306145	*CDC42EP4*	5'UTR/1st intron	N_Shore	0.332±0.095	0.141±0.048	0.191	4.85E-04	2.74E-10	-0.305
cg10749808	14	61746626	*TMEM30B*	1st exon/3'UTR	N_Shore	0.423±0.084	0.231±0.051	0.191	1.85E-04	5.60E-09	-0.282
cg22650942	22	42527158	*CYP2D6*	TSS1500	S_Shore	0.441±0.081	0.25±0.051	0.191	1.22E-04	6.72E-11	-0.314
cg07815521	19	40789857	*AKT2*	5'UTR/1st intron	N_Shore	0.389±0.089	0.197±0.066	0.191	2.32E-04	1.64E-06	-0.234
cg08393516	4	987108	*SLC26A1*	5'UTR/1st exon	S_Shelf	0.513±0.116	0.321±0.051	0.191	1.91E-03	6.41E-06	-0.220
cg20886011	2	165479150	*GRB14*	TSS1500	S_Shore	0.373±0.11	0.182±0.099	0.191	1.13E-03	7.47E-06	-0.219
cg03233793	19	19626605	*TSSK6*	TSS200	Island	0.433±0.12	0.242±0.088	0.191	2.07E-03	1.83E-07	-0.254
cg21940220	17	76184081	*TK1*	TSS1500	S_Shore	0.412±0.087	0.221±0.059	0.191	2.18E-04	2.51E-07	-0.251
cg12433386	11	2722340	*KCNQ1OT1*	TSS1500	S_Shore	0.61±0.101	0.419±0.215	0.191	5.15E-03	1.18E-05	-0.214
cg24640065	1	234615370	*TARBP1*	TSS1500	S_Shore	0.614±0.054	0.424±0.174	0.191	3.29E-04	1.37E-08	-0.275
cg06038358	2	128176007	*PROC*	5'UTR/1st exon	S_Shelf	0.538±0.104	0.347±0.066	0.191	8.15E-04	6.79E-10	-0.298
cg16260700	11	62414786	*GANAB*	TSS1500	S_Shore	0.523±0.126	0.332±0.112	0.191	3.09E-03	5.71E-06	-0.221
cg12639234	8	18248964	*NAT2*	5'UTR/1st intron	S_Shelf	0.559±0.124	0.369±0.071	0.190	2.78E-03	5.98E-12	-0.330
cg07576541	1	153630883	*SNAPIN*	TSS1500	N_Shore	0.732±0.126	0.542±0.243	0.190	1.50E-02	1.82E-11	-0.323

cg15089314	5	78365255	*BHMT2*	TSS1500	N_Shore	0.318±0.096	0.128±0.076	0.190	4.35E-04	2.58E-19	-0.423
cg08331981	19	19626599	*TSSK6*	TSS200	Island	0.504±0.115	0.314±0.11	0.190	1.65E-03	4.03E-08	-0.266
cg05454635	15	74657911	*CYP11A1*	5'UTR/1st intron	N_Shore	0.598±0.186	0.408±0.175	0.190	2.99E-02	1.40E-40	-0.594
cg23160522	15	75015787	*CYP1A1*	5'UTR/1st intron	N_Shelf	0.521±0.069	0.331±0.112	0.190	3.10E-05	2.87E-09	-0.287
cg09861583	2	238769419	*RAMP1*	5'UTR/1st intron	S_Shore	0.595±0.152	0.405±0.228	0.190	2.14E-02	4.79E-19	-0.420
cg20379919	3	184095940	*THPO*	5'UTR	N_Shelf	0.429±0.107	0.239±0.045	0.190	1.21E-03	4.69E-22	-0.451
cg21189492	8	42395162	*SLC20A2*	5'UTR/1st intron	N_Shore	0.56±0.21	0.37±0.144	0.190	4.23E-02	3.94E-09	-0.285
cg13175778	17	202759	*RPH3AL*	TSS200	N_Shelf	0.43±0.123	0.241±0.076	0.190	2.61E-03	7.70E-06	-0.218
cg08736072	16	1471543	*C16orf91*	TSS1500	S_Shore	0.457±0.105	0.267±0.083	0.190	8.33E-04	5.67E-06	-0.221
cg18261909	6	30881571	*VARS2*	TSS1500	Island	0.331±0.087	0.141±0.054	0.190	2.38E-04	1.52E-10	-0.309
cg14080585	20	60639721	*TAF4*	1st exon	Island	0.443±0.082	0.254±0.088	0.189	1.01E-04	1.76E-05	-0.210
cg24656529	4	17515119	*QDPR*	TSS1500	S_Shore	0.817±0.033	0.627±0.145	0.189	3.47E-05	2.95E-16	-0.388
cg11924019	15	75019283	*CYP1A1*	TSS1500	Island	0.422±0.127	0.233±0.078	0.189	3.26E-03	2.22E-06	-0.231
cg14830003	1	38022657	*DNALI1*	1st exon	Island	0.647±0.139	0.458±0.24	0.189	1.95E-02	1.04E-28	-0.510
cg15664854	9	114362707	*PTGR1*	TSS1500	S_Shore	0.781±0.13	0.592±0.191	0.189	8.35E-03	2.64E-14	-0.363
cg03490567	7	43944817	*URGCP*	5'UTR	N_Shore	0.304±0.078	0.115±0.041	0.189	1.26E-04	8.43E-06	-0.217
cg04185382	16	4424441	*VASN*	5'UTR/1st intron	S_Shelf	0.672±0.143	0.483±0.118	0.189	6.96E-03	3.06E-16	-0.388
cg00726046	20	43161260	*PKIG*	5'UTR/1st intron	S_Shore	0.388±0.098	0.2±0.081	0.189	5.29E-04	5.78E-10	-0.299
cg04091702	5	68711640	*MARVELD2*	5'UTR/1st intron	S_Shore	0.367±0.075	0.179±0.057	0.189	6.02E-05	6.41E-08	-0.262
cg03173570	21	33031392	*SOD1*	TSS1500	N_Shore	0.306±0.104	0.117±0.055	0.189	9.63E-04	8.65E-14	-0.356
cg15715507	5	140053663	*DND1*	TSS1500	S_Shore	0.594±0.101	0.405±0.119	0.188	8.00E-04	6.23E-08	-0.263
cg00466249	17	79269884	*SLC38A10*	TSS1500	S_Shore	0.432±0.126	0.244±0.074	0.188	3.38E-03	1.65E-08	-0.274
cg02210151	10	123356041	*FGFR2*	5'UTR/1st exon	N_Shore	0.553±0.156	0.365±0.154	0.188	1.35E-02	7.31E-21	-0.439
cg07551677	8	28570561	*EXTL3*	5'UTR/1st intron	N_Shelf	0.859±0.035	0.671±0.215	0.188	1.88E-03	1.01E-06	-0.238
cg00540067	3	126074344	*KLF15*	5'UTR/1st intron	N_Shore	0.428±0.095	0.24±0.056	0.188	5.18E-04	4.17E-27	-0.497
cg14010829	9	133322940	*ASS1*	5'UTR/1st intron	S_Shelf	0.743±0.125	0.555±0.155	0.188	4.57E-03	8.02E-22	-0.449
cg16550453	10	115939018	*TDRD1*	TSS200	Island	0.881±0.098	0.693±0.232	0.188	7.82E-03	5.97E-15	-0.372
cg13771161	9	99146400	*SLC35D2*	TSS1500	S_Shore	0.379±0.131	0.192±0.057	0.187	4.33E-03	1.37E-09	-0.293
cg26646411	22	19841374	*GNB1L*	5'UTR	N_Shore	0.349±0.107	0.162±0.071	0.187	1.16E-03	7.46E-06	-0.219
cg03886898	1	54359132	*DIO1*	TSS1500	S_Shelf	0.697±0.107	0.51±0.138	0.187	1.54E-03	3.64E-30	-0.522
cg02369725	2	178936951	*PDE11A*	1st exon	N_Shore	0.43±0.112	0.243±0.097	0.187	1.56E-03	2.32E-08	-0.271
cg18500085	11	45827628	*SLC35C1*	1st exon	S_Shore	0.475±0.104	0.288±0.065	0.187	9.82E-04	2.50E-06	-0.230
cg06374185	3	45637480	*LIMD1*	1st exon	S_Shore	0.329±0.1	0.142±0.059	0.187	7.81E-04	4.57E-11	-0.317
cg08932654	17	38084459	*ORMDL3*	TSS1500	S_Shore	0.379±0.115	0.192±0.056	0.187	2.04E-03	8.82E-09	-0.279
cg03566191	14	68067294	*PIGH*	TSS1500	S_Shore	0.601±0.13	0.414±0.156	0.187	5.87E-03	8.55E-06	-0.217
cg03766851	2	47141740	*MCFD2*	5'UTR/1st intron	N_Shore	0.537±0.155	0.35±0.112	0.187	1.12E-02	5.97E-09	-0.282
cg03606774	2	27432830	*SLC5A6*	5'UTR	N_Shore	0.574±0.09	0.387±0.183	0.186	1.94E-03	2.03E-08	-0.272
cg14162906	1	27647606	*TMEM222*	TSS1500	N_Shore	0.562±0.087	0.376±0.136	0.186	4.39E-04	5.03E-06	-0.223
cg09341580	22	38218159	*GALR3*	TSS1500	N_Shelf	0.301±0.09	0.115±0.049	0.186	3.84E-04	2.40E-06	-0.230
cg03419014	21	38362742	*HLCS*	TSS200	Island	0.353±0.12	0.167±0.056	0.186	2.78E-03	6.90E-06	-0.220
cg26178184	16	67232921	*ELMO3*	TSS200	N_Shore	0.613±0.11	0.427±0.175	0.186	3.60E-03	1.02E-16	-0.394
cg07217063	22	30901886	*SEC14L4*	TSS200	S_Shore	0.466±0.103	0.28±0.057	0.186	1.01E-03	6.57E-07	-0.242
cg14525897	10	99053150	*ARHGAP19*	TSS1500	S_Shore	0.591±0.163	0.405±0.162	0.186	1.79E-02	1.39E-05	-0.212
cg13306335	1	2345376	*PEX10*	TSS1500	S_Shore	0.482±0.101	0.296±0.053	0.186	8.69E-04	1.81E-07	-0.254
cg26266876	3	23957486	*NKIRAS1*	5'UTR/1st intron	N_Shore	0.3±0.115	0.114±0.057	0.186	2.09E-03	3.04E-17	-0.400
cg25484139	3	13008273	*IQSEC1*	5'UTR/1st intron	N_Shore	0.298±0.099	0.112±0.04	0.186	8.71E-04	7.55E-06	-0.219
cg18328933	3	52008538	*ABHD14B*	5'UTR/1st exon	S_Shore	0.48±0.083	0.295±0.093	0.186	1.29E-04	1.73E-10	-0.308
cg18328933	3	52008538	*ABHD14A*	TSS1500	S_Shore	0.48±0.083	0.295±0.093	0.186	1.29E-04	8.38E-06	-0.218
cg24810305	14	103389022	*AMN*	5'UTR/1st exon	Island	0.397±0.084	0.211±0.062	0.186	1.88E-04	8.56E-07	-0.240
cg00273124	5	177581512	*NHP2*	TSS1500	S_Shore	0.493±0.119	0.307±0.096	0.186	2.41E-03	2.31E-08	-0.271
cg18981338	14	24422419	*DHRS4*	TSS1500	N_Shore	0.338±0.101	0.152±0.04	0.185	9.99E-04	2.49E-09	-0.288
cg11813387	5	43310340	*HMGCS1*	5'UTR/1st intron	N_Shelf	0.766±0.089	0.581±0.216	0.185	4.87E-03	4.15E-38	-0.578
cg10033675	2	172015098	*TLK1*	5'UTR/1st intron	N_Shore	0.363±0.105	0.178±0.072	0.185	1.05E-03	7.89E-08	-0.261
cg03861785	16	1873952	*HAGH*	5'UTR	N_Shelf	0.413±0.121	0.228±0.079	0.185	2.76E-03	1.49E-20	-0.436
cg00446123	20	62367888	*LIME1*	5'UTR/1st exon	N_Shore	0.625±0.143	0.439±0.306	0.185	4.59E-02	1.27E-23	-0.466
cg08727202	22	37414747	*MPST*	TSS1500	Island	0.441±0.057	0.256±0.076	0.185	2.05E-06	7.02E-06	-0.219
cg17854654	1	155232925	*SCAMP3*	TSS1500	S_Shore	0.566±0.105	0.381±0.151	0.185	1.89E-03	6.03E-06	-0.221
cg19636672	12	56881121	*GLS2*	5'UTR/1st intron	N_Shore	0.404±0.129	0.219±0.082	0.185	4.15E-03	1.10E-09	-0.294
cg16836675	17	52981853	*TOM1L1*	5'UTR	S_Shelf	0.365±0.188	0.18±0.121	0.185	2.88E-02	5.10E-14	-0.360
cg02153339	12	121415499	*HNF1A*	TSS1500	N_Shore	0.464±0.14	0.279±0.125	0.185	7.27E-03	4.31E-19	-0.421
cg18702012	10	126106555	*OAT*	5'UTR/1st intron	N_Shore	0.44±0.125	0.255±0.109	0.185	3.47E-03	3.11E-05	-0.204
cg04361266	16	20910376	*DCUN1D3*	5'UTR/1st intron	N_Shore	0.745±0.089	0.56±0.153	0.185	8.42E-04	1.21E-10	-0.310
cg04361266	16	20910376	*LYRM1*	TSS1500	N_Shore	0.745±0.089	0.56±0.153	0.185	8.42E-04	7.81E-10	-0.297
cg12861503	13	52734071	*NEK3*	TSS1500	S_Shore	0.319±0.099	0.135±0.06	0.185	7.65E-04	1.39E-11	-0.325
cg21296602	11	93476060	*C11orf54*	5'UTR	S_Shore	0.618±0.194	0.433±0.186	0.185	4.07E-02	1.27E-07	-0.257
cg26039042	8	144815275	*FAM83H*	5'UTR/1st intron	Island	0.456±0.091	0.272±0.069	0.185	3.76E-04	3.50E-13	-0.348
cg23772843	13	113759909	*F7*	TSS200	N_Shore	0.418±0.091	0.233±0.06	0.185	3.94E-04	1.76E-15	-0.378
cg08511651	6	26017619	*HIST1H1A*	1st exon	N_Shelf	0.412±0.19	0.228±0.198	0.185	4.03E-02	6.56E-12	-0.330
cg12974394	1	150522256	*ADAMTSL4*	5'UTR/1st intron	Island	0.362±0.1	0.178±0.068	0.184	7.63E-04	6.24E-07	-0.243
cg07042338	17	26990202	*SDF2*	TSS1500	S_Shore	0.41±0.138	0.226±0.115	0.184	6.63E-03	1.46E-07	-0.255
cg07042338	17	26990202	*SUPT6H*	5'UTR/1st intron	S_Shore	0.41±0.138	0.226±0.115	0.184	6.63E-03	1.80E-05	-0.210
cg08794763	19	2977843	*TLE6*	5'UTR/1st intron	S_Shore	0.48±0.153	0.296±0.131	0.184	1.23E-02	1.24E-11	-0.326
cg06979924	1	155277503	*FDPS*	TSS1500	N_Shore	0.441±0.158	0.257±0.089	0.184	1.29E-02	1.13E-07	-0.258
cg27389255	11	14664598	*PDE3B*	TSS1500	N_Shore	0.553±0.132	0.369±0.086	0.184	4.80E-03	1.02E-05	-0.215
cg18149919	8	144912049	*PUF60*	TSS1500	Island	0.585±0.136	0.402±0.092	0.184	5.85E-03	8.03E-17	-0.395
cg05318361	4	1166839	*SPON2*	5'UTR	S_Shore	0.447±0.111	0.263±0.066	0.184	1.69E-03	2.25E-06	-0.231
cg04162118	8	71519030	*TRAM1*	5'UTR/1st intron	N_Shore	0.571±0.186	0.388±0.171	0.184	3.37E-02	1.99E-14	-0.365
cg11001769	14	61748440	*TMEM30B*	5'UTR/1st exon	S_Shore	0.642±0.163	0.458±0.196	0.184	2.40E-02	3.72E-20	-0.432
cg01849085	7	63505811	*ZNF727*	5'UTR/1st exon	N_Shore	0.281±0.17	0.097±0.046	0.184	1.81E-02	8.34E-06	-0.218
cg01854491	8	144815021	*FAM83H*	5'UTR/1st intron	N_Shore	0.378±0.17	0.195±0.122	0.184	1.97E-02	2.76E-15	-0.376
cg07626033	3	11036898	*SLC6A1*	5'UTR/1st intron	S_Shore	0.484±0.14	0.301±0.156	0.183	9.48E-03	6.63E-25	-0.478
cg07593177	11	6501850	*ARFIP2*	5'UTR/1st intron	N_Shore	0.599±0.176	0.416±0.17	0.183	2.77E-02	4.32E-10	-0.301
cg15707869	8	88884985	*DCAF4L2*	1st exon/3'UTR	N_Shore	0.433±0.073	0.25±0.06	0.183	5.91E-05	1.49E-06	-0.234
cg07097417	1	212002770	*LPGAT1*	5'UTR/1st intron	N_Shore	0.33±0.1	0.147±0.06	0.183	8.48E-04	5.68E-07	-0.243
cg04964333	20	39969604	*LPIN3*	1st exon	S_Shore	0.485±0.091	0.302±0.072	0.183	3.73E-04	2.14E-18	-0.413
cg05211342	19	49890915	*CCDC155*	TSS1500	N_Shore	0.659±0.118	0.476±0.187	0.183	6.65E-03	8.64E-09	-0.279
cg04648402	4	987561	*SLC26A1*	TSS1500	S_Shelf	0.521±0.108	0.338±0.051	0.183	1.62E-03	1.39E-05	-0.212

cg27344791	19	12145947	ZNF433	5'UTR/1st intron	N_Shore	0.302±0.132	0.12±0.056	0.183	5.26E-03	7.49E-17	-0.395
cg11084470	6	35108061	TCP11	5'UTR	N_Shore	0.417±0.108	0.234±0.105	0.182	1.44E-03	2.18E-05	-0.208
cg22399646	2	242750705	NEU4	5'UTR/1st intron	N_Shelf	0.524±0.18	0.342±0.171	0.182	3.06E-02	4.67E-30	-0.521
cg10377144	10	89419177	PAPSS2	TSS1500	N_Shore	0.505±0.135	0.323±0.157	0.182	8.40E-03	4.00E-17	-0.398
cg21854895	17	71305638	CDC42EP4	5'UTR/1st intron	N_Shore	0.565±0.164	0.383±0.179	0.182	2.30E-02	2.52E-10	-0.305
cg04859918	10	71893017	AIFM2	TSS1500	S_Shore	0.486±0.103	0.304±0.137	0.182	1.53E-03	1.40E-05	-0.212
cg07168142	17	40274740	HSPB9	TSS200	Island	0.511±0.147	0.329±0.122	0.182	1.03E-02	2.40E-15	-0.377
cg22785629	17	1554549	RILP	TSS1500	S_Shore	0.651±0.102	0.469±0.151	0.182	1.87E-03	1.67E-06	-0.233
cg18138826	6	44354646	CDC5L	TSS1500	N_Shore	0.417±0.124	0.235±0.15	0.182	5.17E-03	5.90E-12	-0.330
cg06182359	1	161088957	PFDN2	TSS1500	S_Shore	0.335±0.109	0.154±0.075	0.182	1.53E-03	2.58E-05	-0.206
cg04084026	16	57832309	KIFC3	5'UTR/1st intron	N_Shelf	0.62±0.121	0.438±0.118	0.182	3.42E-03	4.30E-05	-0.200
cg00727310	2	227662704	IRS1	1st exon	Island	0.501±0.126	0.32±0.071	0.182	4.11E-03	5.32E-09	-0.283
cg10085305	17	77704942	ENPP7	1st exon	N_Shelf	0.565±0.142	0.383±0.077	0.182	7.89E-03	1.83E-16	-0.391
cg25464235	13	93878289	GPC6	TSS1500	N_Shore	0.524±0.147	0.342±0.119	0.182	1.04E-02	2.16E-11	-0.322
cg07546654	11	1320546	TOLLIP	5'UTR/1st intron	S_Shelf	0.371±0.15	0.19±0.063	0.181	1.09E-02	4.89E-06	-0.223
cg06460691	5	74162851	FAM169A	TSS1500	S_Shore	0.445±0.102	0.264±0.113	0.181	1.06E-03	7.92E-20	-0.428
cg18288193	5	180233833	MGAT1	5'UTR	N_Shelf	0.417±0.103	0.236±0.087	0.181	1.05E-03	2.96E-07	-0.249
cg07800524	19	49259452	FGF21	5'UTR	S_Shelf	0.509±0.114	0.328±0.103	0.181	2.20E-03	1.69E-11	-0.324
cg13104938	11	843956	TSPAN4	5'UTR/1st intron	S_Shore	0.353±0.127	0.172±0.132	0.181	5.11E-03	1.43E-14	-0.367
cg16545821	1	204118840	ETNK2	5'UTR/1st intron	N_Shore	0.785±0.079	0.604±0.223	0.181	5.77E-03	4.76E-40	-0.590
cg24725692	4	140478735	SETD7	TSS1500	S_Shore	0.324±0.094	0.143±0.039	0.181	7.51E-04	3.50E-06	-0.226
cg20640261	6	31707019	MSH5	TSS1500	N_Shore	0.325±0.102	0.144±0.045	0.181	1.25E-03	3.34E-20	-0.433
cg13500113	2	216947149	TMEM169	5'UTR	S_Shore	0.496±0.119	0.315±0.109	0.181	2.99E-03	2.07E-17	-0.402
cg13500113	2	216947149	PECR	TSS1500	S_Shore	0.496±0.119	0.315±0.109	0.181	2.99E-03	1.01E-10	-0.312
cg01878807	14	24422368	DHRS4	TSS1500	N_Shore	0.393±0.135	0.212±0.083	0.181	6.14E-03	5.87E-16	-0.384
cg15546227	1	61546247	NFIA	TSS1500	N_Shelf	0.342±0.115	0.161±0.06	0.181	2.42E-03	1.41E-17	-0.404
cg14186992	1	6639570	ZBTB48	TSS1500	Island	0.486±0.137	0.306±0.072	0.181	6.86E-03	1.39E-09	-0.293
cg17886959	16	56642024	MT2A	TSS1500	N_Shore	0.377±0.086	0.197±0.086	0.181	2.36E-04	4.19E-09	-0.284
cg19258882	12	56472805	ERBB3	TSS1500	Island	0.337±0.1	0.156±0.064	0.181	9.27E-04	3.97E-10	-0.302
cg01054354	15	100272751	LYSMD4	5'UTR	N_Shore	0.38±0.125	0.199±0.082	0.181	3.99E-03	9.96E-08	-0.259
cg11243029	1	85929755	DDAH1	5'UTR	N_Shore	0.335±0.085	0.154±0.044	0.180	3.54E-04	7.33E-06	-0.219
cg09710316	3	170744871	SLC2A2	TSS200	N_Shore	0.418±0.105	0.238±0.056	0.180	1.42E-03	1.23E-10	-0.310
cg02654291	9	86572014	C9orf64	TSS1500	Island	0.384±0.101	0.203±0.052	0.180	1.14E-03	1.37E-12	-0.340
cg09102447	8	57907138	IMPAD1	TSS1500	S_Shore	0.31±0.093	0.13±0.076	0.180	4.85E-04	3.06E-08	-0.269
cg08530484	2	220024606	NHEJ1	5'UTR/1st intron	N_Shore	0.345±0.1	0.164±0.062	0.180	9.54E-04	5.23E-08	-0.264
cg11865601	6	24491317	GPLD1	TSS1500	N_Shelf	0.859±0.059	0.679±0.211	0.180	2.97E-03	4.35E-25	-0.480
cg12127991	1	228870637	RHOU	TSS200	N_Shore	0.35±0.098	0.17±0.114	0.180	8.77E-04	6.45E-14	-0.358
cg05399544	14	74481427	ENTPD5	5'UTR	N_Shelf	0.647±0.12	0.467±0.173	0.180	6.36E-03	2.84E-06	-0.228
cg07994485	2	97652440	FAM178B	TSS200	Island	0.793±0.121	0.613±0.237	0.180	1.75E-02	4.21E-22	-0.452
cg18222865	11	14664689	PDE3B	TSS1500	N_Shore	0.435±0.102	0.255±0.061	0.180	1.15E-03	1.81E-07	-0.254
cg00912149	17	74449810	UBE2O	TSS1500	S_Shore	0.305±0.072	0.125±0.05	0.180	7.18E-05	7.16E-13	-0.344
cg02985694	4	123300456	ADAD1	5'UTR/1st intron	Island	0.787±0.08	0.607±0.204	0.179	3.65E-03	1.95E-05	-0.209
cg24724917	11	2925439	SLC22A18AS	TSS1500	S_Shore	0.534±0.156	0.354±0.126	0.179	1.50E-02	4.52E-19	-0.420
cg14115346	6	3118207	BPHL	TSS1500	N_Shore	0.343±0.088	0.164±0.067	0.179	3.48E-04	4.82E-08	-0.265
cg07555437	20	45279830	SLC13A3	5'UTR	Island	0.531±0.127	0.352±0.129	0.179	5.46E-03	4.79E-11	-0.317
cg04315771	19	50433949	ATF5	5'UTR	S_Shore	0.539±0.123	0.36±0.255	0.179	2.38E-02	1.74E-28	-0.509
cg25114611	6	35696870	FKBP5	TSS1500	N_Shelf	0.424±0.11	0.245±0.078	0.179	1.83E-03	2.79E-11	-0.320
cg14738273	3	126074601	KLF15	5'UTR/1st intron	N_Shore	0.369±0.102	0.19±0.055	0.179	1.24E-03	6.73E-17	-0.396
cg12188860	8	144416485	TOP1MT	5'UTR	N_Shore	0.51±0.124	0.33±0.069	0.179	4.00E-03	1.13E-05	-0.214
cg17781925	3	49726151	RNF123	TSS1500	S_Shore	0.463±0.096	0.284±0.059	0.179	7.56E-04	9.86E-08	-0.259
cg17781925	3	49726151	MST1	1st exon	S_Shore	0.463±0.096	0.284±0.059	0.179	7.56E-04	1.50E-06	-0.234
cg22691587	1	40349894	TRIT1	TSS1500	S_Shore	0.571±0.139	0.392±0.125	0.179	8.52E-03	4.63E-06	-0.224
cg26934993	17	40274722	HSPB9	TSS200	Island	0.484±0.155	0.305±0.116	0.179	1.41E-02	4.71E-16	-0.386
cg00533390	1	245028657	HNRNPU	TSS1500	S_Shore	0.396±0.118	0.217±0.129	0.179	3.52E-03	1.86E-05	-0.209
cg01621080	16	28608125	SULT1A2	5'UTR/1st intron	N_Shelf	0.507±0.126	0.328±0.094	0.179	4.41E-03	3.55E-14	-0.362
cg08181251	10	99443455	AVPI1	5'UTR/1st intron	N_Shelf	0.459±0.084	0.28±0.101	0.179	2.37E-04	8.84E-22	-0.448
cg20067780	17	78008520	TBC1D16	5'UTR/1st intron	N_Shore	0.459±0.165	0.281±0.174	0.179	2.49E-02	2.07E-20	-0.435
cg11935638	3	184972145	EHHADH	TSS1500	S_Shore	0.318±0.099	0.14±0.086	0.179	8.45E-04	6.80E-07	-0.242
cg12727940	3	145969718	PLSCR4	TSS1500	S_Shore	0.674±0.076	0.495±0.205	0.178	3.57E-03	2.04E-11	-0.322
cg24892871	19	10611043	KEAP1	5'UTR/1st intron	N_Shelf	0.603±0.083	0.424±0.143	0.178	6.20E-04	1.08E-06	-0.237
cg24670552	13	110436780	IRS2	1st exon	Island	0.563±0.129	0.385±0.183	0.178	1.05E-02	5.00E-09	-0.283
cg01617750	3	32279261	CMTM8	TSS1500	N_Shore	0.364±0.094	0.186±0.061	0.178	6.47E-04	2.16E-19	-0.424
cg15393702	18	21243529	ANKRD29	TSS1500	S_Shore	0.338±0.123	0.16±0.118	0.178	4.31E-03	8.24E-07	-0.240
cg06998765	14	75389618	RPS6KL1	TSS1500	N_Shore	0.485±0.097	0.306±0.127	0.178	1.04E-03	5.11E-29	-0.513
cg25886479	11	50257625	LOC441601	1st exon	Island	0.655±0.13	0.477±0.202	0.178	1.38E-02	1.75E-13	-0.352
cg08234618	3	133464842	TF	5'UTR	N_Shore	0.356±0.096	0.179±0.047	0.178	8.61E-04	5.61E-10	-0.299
cg20310100	11	68081207	LRP5	5'UTR/1st intron	S_Shore	0.395±0.139	0.217±0.094	0.178	8.27E-03	2.76E-07	-0.250
cg20113012	17	71308660	CDC42EP4	TSS1500	Island	0.253±0.101	0.076±0.045	0.178	1.30E-03	7.89E-09	-0.279
cg07702888	1	156338793	RHBG	TSS200	Island	0.41±0.08	0.233±0.074	0.177	1.50E-04	3.51E-07	-0.248
cg25325446	5	139938201	SRA1	TSS1500	S_Shore	0.872±0.029	0.695±0.153	0.177	1.36E-04	9.27E-11	-0.312
cg14398963	11	2397538	CD81	5'UTR/1st exon	N_Shore	0.511±0.124	0.334±0.146	0.177	6.00E-03	5.67E-07	-0.243
cg04716394	14	74318162	PTGR2	TSS1500	N_Shore	0.339±0.085	0.163±0.057	0.177	3.37E-04	1.57E-11	-0.324
cg07959978	1	65994091	LEPR	5'UTR	S_Shelf	0.642±0.097	0.466±0.17	0.177	2.84E-03	9.84E-17	-0.394
cg09430445	3	42918546	CYP8B1	TSS1500	N_Shelf	0.536±0.138	0.36±0.184	0.176	1.48E-02	4.62E-23	-0.461
cg09557462	8	26721736	ADRA1A	1st exon	Island	0.38±0.13	0.203±0.121	0.176	6.50E-03	3.35E-22	-0.453
cg03512692	22	44350088	SAMM50	TSS1500	N_Shore	0.531±0.081	0.356±0.137	0.176	5.22E-04	9.26E-06	-0.217
cg24772525	2	172014861	TLK1	5'UTR/1st intron	N_Shore	0.636±0.135	0.461±0.181	0.176	1.33E-02	1.27E-09	-0.293
cg06872514	9	139746715	MAMDC4	TSS200	S_Shelf	0.517±0.112	0.341±0.123	0.176	2.83E-03	1.80E-08	-0.273
cg16529268	19	45281284	CBLC	1st exon	Island	0.257±0.098	0.082±0.049	0.176	1.13E-03	6.38E-10	-0.298
cg12230883	3	184035527	EIF4G1	5'UTR	S_Shelf	0.681±0.165	0.505±0.157	0.176	2.45E-02	6.12E-12	-0.330
cg07148318	8	145115260	OPLAH	5'UTR/1st intron	S_Shore	0.571±0.126	0.395±0.084	0.175	4.85E-03	3.35E-11	-0.319
cg09419900	10	124739466	PSTK	TSS200	N_Shore	0.312±0.07	0.137±0.043	0.175	8.56E-05	6.51E-09	-0.281
cg08736216	1	53307985	ZYG11A	TSS200	N_Shore	0.604±0.084	0.429±0.159	0.175	1.32E-03	2.49E-27	-0.499
cg05151185	11	45827271	SLC35C1	5'UTR/1st exon	S_Shore	0.319±0.085	0.144±0.052	0.175	3.62E-04	3.86E-05	-0.201
cg03486974	16	2199702	RAB26	5'UTR	S_Shore	0.555±0.126	0.38±0.102	0.175	5.07E-03	4.32E-05	-0.200
cg23448505	19	37826008	HKR1	5'UTR	S_Shore	0.572±0.151	0.397±0.18	0.175	2.08E-02	1.09E-16	-0.393
cg12024906	19	37825679	HKR1	1st exon	Island	0.457±0.179	0.282±0.208	0.175	4.43E-02	7.92E-13	-0.343

cg26271591	2	178125956	*NFE2L2*	5'UTR/1st intron	N_Shelf	0.282±0.1	0.106±0.043	0.175	1.32E-03	1.63E-06	-0.234
cg18485872	2	242750355	*NEU4*	5'UTR/1st exon	N_Shelf	0.565±0.085	0.39±0.08	0.175	2.87E-04	4.08E-18	-0.410
cg08534147	3	48471771	*PLXNB1*	TSS1500	S_Shore	0.358±0.099	0.183±0.053	0.175	1.21E-03	7.82E-07	-0.241
cg08601673	16	2083393	*SLC9A3R2*	1st exon	N_Shelf	0.475±0.135	0.301±0.147	0.175	9.88E-03	7.01E-26	-0.487
cg17647273	22	24891452	*UPB1*	1st exon	Island	0.52±0.108	0.346±0.052	0.175	2.07E-03	5.05E-33	-0.543
cg22061832	2	173293627	*ITGA6*	5'UTR/1st intron	S_Shore	0.351±0.115	0.176±0.085	0.175	2.82E-03	2.52E-08	-0.270
cg10999598	5	125936257	*PHAX*	TSS1500	N_Shore	0.343±0.057	0.169±0.066	0.174	5.01E-06	1.11E-05	-0.215
cg21402035	22	38219400	*GALR3*	5'UTR/1st exon	N_Shore	0.52±0.109	0.346±0.099	0.174	2.10E-03	1.47E-05	-0.212
cg11705746	11	2398061	*CD81*	5'UTR/1st intron	N_Shore	0.462±0.104	0.288±0.124	0.174	1.92E-03	4.11E-05	-0.201
cg25117600	4	10023201	*SLC2A9*	TSS200	S_Shelf	0.297±0.078	0.123±0.103	0.174	1.73E-04	5.63E-19	-0.419
cg01883777	22	38201848	*H1F0*	1st exon	S_Shore	0.265±0.079	0.091±0.042	0.174	2.60E-04	7.35E-18	-0.407
cg24474409	7	150021518	*LRRC61*	5'UTR/1st intron	S_Shore	0.512±0.125	0.339±0.073	0.174	5.05E-03	9.12E-12	-0.328
cg10409299	7	150498843	*TMEM176B*	TSS1500	S_Shore	0.491±0.091	0.317±0.072	0.174	5.83E-04	1.06E-19	-0.427
cg08976550	21	46876385	*COL18A1*	1st exon	Island	0.598±0.119	0.425±0.08	0.174	3.73E-03	1.87E-06	-0.232
cg23959311	1	229406360	*RAB4A*	TSS1500	N_Shore	0.402±0.105	0.228±0.073	0.174	1.68E-03	2.17E-16	-0.390
cg19576556	17	80024177	*DUS1L*	TSS1500	Island	0.527±0.118	0.354±0.161	0.173	6.37E-03	5.22E-09	-0.283
cg21577356	7	150021488	*LRRC61*	5'UTR/1st intron	S_Shore	0.434±0.105	0.261±0.057	0.173	1.78E-03	2.85E-09	-0.287
cg02451670	1	212207806	*DTL*	TSS1500	N_Shore	0.358±0.097	0.185±0.066	0.173	9.84E-04	3.74E-05	-0.202
cg23718736	18	6413908	*L3MBTL4*	5'UTR/1st intron	N_Shore	0.371±0.124	0.197±0.071	0.173	4.82E-03	1.81E-12	-0.338
cg15114013	1	41159283	*NFYC*	5'UTR/1st intron	S_Shore	0.495±0.086	0.322±0.119	0.173	5.39E-04	3.50E-08	-0.268
cg23497644	2	170654835	*SSB*	TSS1500	N_Shore	0.347±0.068	0.174±0.083	0.173	3.99E-05	3.86E-06	-0.225
cg07645844	8	26722635	*ADRA1A*	1st exon	Island	0.374±0.076	0.201±0.07	0.173	1.12E-04	7.09E-14	-0.358
cg03870862	8	144624225	*ZC3H3*	TSS1500	S_Shore	0.552±0.117	0.379±0.153	0.173	5.62E-03	1.90E-05	-0.209
cg07308161	2	211090487	*ACADL*	TSS1500	S_Shore	0.405±0.134	0.232±0.133	0.173	9.18E-03	1.77E-15	-0.378
cg01116810	22	24891153	*UPB1*	TSS200	Island	0.51±0.099	0.337±0.039	0.173	1.42E-03	5.76E-12	-0.331
cg05063690	21	46876305	*COL18A1*	1st exon	Island	0.632±0.121	0.459±0.083	0.173	4.15E-03	1.79E-09	-0.291
cg14076390	17	34947837	*DHRS11*	TSS1500	N_Shore	0.43±0.116	0.257±0.117	0.173	3.67E-03	4.73E-10	-0.301
cg26385222	7	150497077	*TMEM176B*	5'UTR/1st intron	Island	0.31±0.097	0.137±0.062	0.173	1.03E-03	2.65E-10	-0.305
cg26385222	7	150497077	*TMEM176A*	TSS1500	Island	0.31±0.097	0.137±0.062	0.173	1.03E-03	1.17E-09	-0.294
cg20277356	10	123353800	*FGFR2*	5'UTR/1st intron	N_Shelf	0.744±0.111	0.571±0.195	0.173	9.11E-03	4.40E-14	-0.360
cg26134090	4	48909057	*OCIAD2*	TSS1500	S_Shore	0.478±0.112	0.306±0.046	0.173	3.00E-03	7.68E-27	-0.495
cg09476306	22	22086335	*YPEL1*	5'UTR/1st intron	N_Shelf	0.776±0.043	0.603±0.205	0.173	2.78E-03	2.55E-29	-0.515
cg18642234	3	49394622	*GPX1*	1st exon/3'UTR	N_Shore	0.361±0.138	0.189±0.128	0.173	1.02E-02	1.44E-18	-0.415
cg25683744	12	681324	*NINJ2*	5'UTR/1st intron	S_Shelf	0.638±0.121	0.466±0.187	0.172	1.07E-02	1.13E-11	-0.326
cg20995835	17	18528589	*CCDC144B*	1st exon	Island	0.725±0.08	0.552±0.129	0.172	4.43E-04	2.13E-14	-0.365
cg00003014	3	197279819	*BDH1*	5'UTR	N_Shore	0.759±0.086	0.587±0.15	0.172	1.24E-03	2.29E-09	-0.289
cg08820801	19	39465821	*FBXO17*	5'UTR/1st intron	N_Shore	0.384±0.086	0.212±0.067	0.172	3.74E-04	3.11E-06	-0.227
cg13504907	9	5185586	*INSL6*	1st exon	Island	0.739±0.07	0.567±0.192	0.172	2.83E-03	4.43E-14	-0.360
cg13348905	19	10735578	*SLC44A2*	TSS1500	N_Shore	0.665±0.095	0.493±0.264	0.172	2.29E-02	2.21E-07	-0.252
cg14078053	2	227661656	*IRS1*	1st exon	N_Shore	0.556±0.077	0.384±0.077	0.172	1.30E-04	5.46E-12	-0.331
cg02641676	8	42397764	*SLC20A2*	TSS1500	S_Shore	0.363±0.129	0.191±0.083	0.172	6.45E-03	9.86E-13	-0.342
cg23684449	16	46919194	*GPT2*	5'UTR/1st exon	S_Shore	0.438±0.12	0.266±0.066	0.172	4.20E-03	9.45E-25	-0.477
cg17719951	2	69970639	*ANXA4*	5'UTR	S_Shore	0.549±0.171	0.377±0.118	0.171	2.77E-02	1.51E-06	-0.234
cg12432038	1	247275735	*C1orf229*	TSS200	N_Shelf	0.635±0.146	0.464±0.231	0.171	3.33E-02	2.88E-15	-0.376
cg04604142	17	48796515	*LUC7L3*	TSS1500	N_Shore	0.355±0.095	0.184±0.076	0.171	8.90E-04	5.03E-07	-0.245
cg09355820	1	10092237	*UBE4B*	TSS1500	N_Shore	0.6±0.154	0.428±0.138	0.171	1.92E-02	4.53E-06	-0.224
cg00714531	21	46955647	*SLC19A1*	5'UTR	N_Shore	0.879±0.015	0.708±0.221	0.171	4.37E-03	4.46E-10	-0.301
cg16385330	4	151503878	*MAB21L2*	5'UTR/1st exon	S_Shelf	0.658±0.122	0.486±0.248	0.171	2.71E-02	3.44E-06	-0.226
cg06599920	14	47121117	*RPL10L*	TSS200	Island	0.642±0.152	0.471±0.197	0.171	2.76E-02	1.10E-06	-0.237
cg21330960	22	23415915	*GNAZ*	5'UTR/1st intron	S_Shelf	0.582±0.078	0.411±0.11	0.171	2.40E-04	1.35E-06	-0.235
cg14324805	22	30902615	*SEC14L4*	TSS1500	S_Shore	0.543±0.141	0.372±0.137	0.171	1.29E-02	2.70E-05	-0.205
cg11961138	17	38599366	*IGFBP4*	TSS1500	N_Shore	0.287±0.1	0.116±0.085	0.171	1.30E-03	3.88E-05	-0.201
cg02976109	12	124119053	*EIF2B1*	TSS1500	S_Shore	0.409±0.105	0.238±0.059	0.171	2.00E-03	7.24E-13	-0.344
cg04406254	19	45407945	*APOE*	TSS1500	N_Shelf	0.429±0.08	0.258±0.058	0.171	2.53E-04	1.33E-07	-0.256
cg13883232	9	139746122	*MAMDC4*	TSS1500	S_Shore	0.721±0.059	0.55±0.195	0.171	2.62E-03	6.60E-31	-0.528
cg13987606	1	160002698	*PIGM*	TSS1500	S_Shore	0.365±0.108	0.194±0.103	0.171	2.27E-03	1.76E-15	-0.378
cg17329648	2	73297389	*SFXN5*	5'UTR/1st intron	N_Shore	0.268±0.125	0.097±0.052	0.171	5.78E-03	2.59E-14	-0.363
cg24607398	3	37033625	*EPM2AIP1*	1st exon	N_Shore	0.411±0.173	0.241±0.118	0.171	2.95E-02	2.60E-08	-0.270
cg17639959	2	73297338	*SFXN5*	5'UTR/1st intron	N_Shore	0.339±0.116	0.168±0.054	0.171	3.73E-03	2.69E-10	-0.305
cg26385085	4	53109	*ZNF595*	TSS200	N_Shore	0.429±0.041	0.259±0.073	0.171	1.26E-07	1.45E-16	-0.392
cg18919106	8	132918665	*EFR3A*	5'UTR/1st intron	S_Shore	0.289±0.129	0.119±0.097	0.170	7.03E-03	9.51E-12	-0.327
cg26301689	3	142681704	*PAQR9*	1st exon	Island	0.57±0.095	0.4±0.065	0.170	9.84E-04	1.35E-15	-0.380
cg05800289	11	77301496	*AQP11*	1st exon	S_Shore	0.279±0.085	0.109±0.052	0.170	4.84E-04	1.69E-07	-0.254
cg22778981	17	37856133	*ERBB2*	5'UTR	N_Shore	0.237±0.106	0.067±0.039	0.170	2.44E-03	1.36E-07	-0.256
cg01116188	13	36788859	*SOHLH2*	TSS200	Island	0.738±0.129	0.569±0.169	0.169	1.22E-02	1.26E-05	-0.213
cg13723011	3	52737693	*GLT8D1*	5'UTR	N_Shore	0.801±0.176	0.632±0.199	0.169	4.61E-02	1.19E-28	-0.510
cg03963919	6	33167187	*SLC39A7*	TSS1500	N_Shore	0.431±0.061	0.263±0.113	0.169	5.67E-05	6.56E-07	-0.242
cg01313363	3	48471695	*PLXNB1*	TSS1500	S_Shore	0.362±0.087	0.194±0.046	0.169	6.18E-04	6.30E-09	-0.281
cg06624368	16	30797627	*ZNF629*	5'UTR/1st intron	N_Shore	0.374±0.101	0.205±0.08	0.169	1.57E-03	2.08E-06	-0.231
cg00468320	8	109800379	*TMEM74*	TSS1500	S_Shore	0.588±0.101	0.419±0.2	0.168	8.96E-03	5.64E-14	-0.359
cg23112188	14	24563095	*PCK2*	TSS1500	N_Shore	0.279±0.086	0.111±0.047	0.168	5.65E-04	4.60E-11	-0.317
cg27210390	17	52978583	*TOM1L1*	5'UTR/1st intron	S_Shore	0.313±0.102	0.145±0.048	0.168	1.94E-03	2.16E-11	-0.322
cg04240583	1	151485306	*CGN*	5'UTR/1st intron	S_Shore	0.433±0.149	0.265±0.116	0.168	1.65E-02	3.61E-33	-0.544
cg03989567	1	236686012	*LGALS8*	5'UTR	N_Shore	0.484±0.087	0.316±0.136	0.168	1.10E-03	2.09E-05	-0.208
cg00715323	2	11479980	*ROCK2*	5'UTR/1st intron	N_Shelf	0.795±0.067	0.627±0.242	0.168	1.26E-02	6.01E-10	-0.299
cg20939084	3	147109784	*ZIC4*	1st exon	Island	0.418±0.091	0.25±0.134	0.168	1.42E-03	8.70E-07	-0.240
cg24022152	11	12133196	*MICAL2*	5'UTR/1st intron	S_Shore	0.628±0.16	0.46±0.18	0.168	3.11E-02	1.43E-16	-0.392
cg24184792	8	132919238	*EFR3A*	5'UTR/1st intron	S_Shelf	0.795±0.042	0.627±0.203	0.168	3.17E-03	1.00E-18	-0.417
cg14688272	17	80673958	*FN3KRP*	TSS1500	N_Shore	0.416±0.116	0.248±0.113	0.168	4.47E-03	1.63E-05	-0.211
cg15466702	20	62688050	*TCEA2*	TSS1500	N_Shore	0.6±0.13	0.432±0.186	0.168	1.61E-02	3.15E-07	-0.249
cg02216394	6	30178715	*TRIM26*	5'UTR/1st intron	N_Shelf	0.693±0.062	0.525±0.129	0.168	1.61E-04	5.89E-14	-0.359
cg05546038	16	67207033	*NOL3*	5'UTR	S_Shelf	0.698±0.054	0.531±0.205	0.168	3.91E-03	3.24E-06	-0.227
cg16785938	4	10022984	*SLC2A9*	1st exon	S_Shore	0.502±0.066	0.334±0.179	0.168	2.00E-03	3.22E-46	-0.626
cg12575928	11	22687660	*GAS2*	TSS1500	N_Shore	0.327±0.106	0.159±0.058	0.168	2.34E-03	6.90E-11	-0.314
cg06452665	13	43148436	*TNFSF11*	5'UTR	Island	0.256±0.168	0.088±0.122	0.168	2.83E-02	2.93E-06	-0.228
cg03534662	3	45636225	*LIMD1*	TSS200	S_Shore	0.279±0.081	0.111±0.042	0.168	3.88E-04	7.31E-09	-0.280
cg01330312	10	51567670	*NCOA4*	5'UTR/1st intron	S_Shelf	0.542±0.066	0.374±0.142	0.168	4.09E-04	3.71E-05	-0.202

cg14037218	1	150522367	*ADAMTSL4*	5'UTR	Island	0.413±0.1	0.246±0.073	0.167	1.49E-03	3.77E-10	-0.302
cg25274157	19	19626576	*TSSK6*	TSS200	Island	0.448±0.119	0.281±0.105	0.167	4.89E-03	1.19E-06	-0.237
cg20366479	2	217501125	*IGFBP2*	5'UTR/1st intron	S_Shelf	0.478±0.125	0.311±0.178	0.167	1.30E-02	4.87E-26	-0.488
cg10451262	8	124284176	*ZHX1*	5'UTR/1st intron	N_Shelf	0.364±0.131	0.197±0.072	0.167	8.20E-03	2.13E-13	-0.351
cg18354594	1	247276647	*C1orf229*	TSS1500	N_Shelf	0.506±0.155	0.339±0.193	0.167	3.18E-02	8.05E-10	-0.297
cg20664247	1	2345475	*PEX10*	TSS1500	S_Shore	0.408±0.087	0.242±0.061	0.166	5.87E-04	2.02E-06	-0.232
cg06738887	15	60688010	*ANXA2*	5'UTR	N_Shelf	0.324±0.153	0.158±0.086	0.166	1.80E-02	1.34E-09	-0.293
cg11242907	17	45919419	*SCRN2*	TSS1500	S_Shore	0.362±0.121	0.196±0.074	0.166	5.44E-03	6.33E-10	-0.298
cg09322432	22	42527611	*CYP2D6*	TSS1500	S_Shore	0.718±0.069	0.552±0.163	0.166	1.27E-03	2.67E-13	-0.350
cg06444781	12	121416510	*HNF1A*	5'UTR/1st exon	Island	0.538±0.108	0.372±0.064	0.166	2.69E-03	1.94E-21	-0.445
cg08172037	1	92414985	*BRDT*	5'UTR/1st exon	Island	0.861±0.06	0.695±0.199	0.166	3.85E-03	3.10E-12	-0.335
cg06477464	5	68790044	*OCLN*	5'UTR/1st intron	S_Shore	0.525±0.092	0.359±0.074	0.166	8.49E-04	6.00E-08	-0.263
cg11123545	5	139939040	*SRA1*	TSS1500	S_Shore	0.739±0.048	0.574±0.144	0.166	2.28E-04	1.40E-07	-0.256
cg06041363	22	46663418	*TTC38*	TSS1500	N_Shore	0.259±0.094	0.093±0.04	0.166	1.32E-03	2.26E-10	-0.306
cg07452306	2	73299297	*SFXN5*	TSS1500	S_Shore	0.342±0.097	0.177±0.048	0.165	1.49E-03	1.73E-14	-0.366
cg03065614	6	44096542	*TMEM63B*	5'UTR/1st intron	S_Shore	0.581±0.128	0.416±0.104	0.165	8.14E-03	1.29E-07	-0.257
cg03065614	6	44096542	*MRPL14*	TSS1500	S_Shore	0.581±0.128	0.416±0.104	0.165	8.14E-03	4.32E-06	-0.224
cg09063149	1	151882779	*THEM4*	TSS1500	S_Shore	0.385±0.119	0.22±0.117	0.165	5.95E-03	1.83E-09	-0.291
cg04617640	21	46874243	*COL18A1*	TSS1500	N_Shore	0.369±0.103	0.205±0.057	0.165	2.12E-03	1.69E-07	-0.254
cg14396214	20	62367998	*LIME1*	5'UTR/1st exon	N_Shore	0.429±0.08	0.265±0.153	0.165	1.52E-03	3.65E-21	-0.442
cg20772512	1	38022359	*DNALI1*	TSS200	N_Shore	0.281±0.107	0.117±0.076	0.165	2.68E-03	4.49E-10	-0.301
cg19898448	4	125631415	*ANKRD50*	5'UTR/1st intron	N_Shore	0.681±0.092	0.516±0.181	0.164	5.52E-03	7.32E-13	-0.344
cg03123541	1	95699097	*RWDD3*	TSS1500	N_Shore	0.295±0.092	0.131±0.053	0.164	1.05E-03	2.24E-05	-0.207
cg10990993	3	37033632	*EPM2AIP1*	1st exon	N_Shore	0.394±0.152	0.23±0.1	0.164	1.90E-02	4.73E-08	-0.265
cg16121744	21	46875149	*COL18A1*	TSS1500	N_Shore	0.456±0.1	0.291±0.064	0.164	1.79E-03	1.78E-05	-0.210
cg24731625	1	247276552	*C1orf229*	TSS1500	N_Shelf	0.355±0.105	0.191±0.082	0.164	2.45E-03	4.11E-08	-0.266
cg24855045	10	18938449	*NSUN6*	5'UTR/1st intron	N_Shelf	0.415±0.085	0.251±0.054	0.164	6.07E-04	9.96E-11	-0.312
cg17950169	1	226067900	*TMEM63A*	5'UTR/1st intron	N_Shore	0.48±0.123	0.316±0.162	0.164	1.13E-02	1.54E-08	-0.274
cg12633680	7	150779047	*FASTK*	TSS1500	Island	0.387±0.095	0.223±0.056	0.164	1.28E-03	3.95E-05	-0.201
cg01960280	6	100015610	*CCNC*	5'UTR/1st intron	N_Shore	0.304±0.099	0.14±0.052	0.164	1.82E-03	4.84E-09	-0.283
cg22379576	1	173790504	*CENPL*	5'UTR	N_Shelf	0.476±0.085	0.312±0.117	0.164	7.72E-04	1.75E-08	-0.273
cg12089204	11	1318605	*TOLLIP*	5'UTR/1st intron	S_Shore	0.845±0.068	0.681±0.206	0.164	6.08E-03	4.30E-10	-0.301
cg06520846	1	42921582	*ZMYND12*	1st exon	N_Shore	0.352±0.156	0.188±0.137	0.164	2.47E-02	4.47E-28	-0.505
cg25587233	9	131872005	*CRAT*	5'UTR/1st intron	N_Shore	0.42±0.1	0.256±0.057	0.164	1.87E-03	4.83E-10	-0.300
cg02968557	17	40275126	*HSPB9*	1st exon	Island	0.455±0.12	0.292±0.079	0.164	5.66E-03	1.55E-14	-0.366
cg09886641	15	69223018	*SPESP1*	1st exon	Island	0.623±0.124	0.459±0.124	0.164	8.02E-03	1.18E-08	-0.276
cg21157873	1	151486487	*CGN*	5'UTR/1st intron	S_Shelf	0.392±0.124	0.229±0.073	0.163	6.72E-03	2.14E-09	-0.289
cg06850526	17	79269886	*SLC38A10*	TSS1500	S_Shore	0.41±0.107	0.246±0.072	0.163	2.87E-03	4.40E-08	-0.266
cg21993406	5	68484783	*CENPH*	TSS1500	N_Shore	0.406±0.11	0.242±0.122	0.163	4.21E-03	2.78E-05	-0.205
cg23016776	10	99081496	*FRAT1*	1st exon/3'UTR	S_Shore	0.561±0.172	0.397±0.172	0.163	4.29E-02	6.78E-08	-0.262
cg25318278	11	50257830	*LOC441601*	TSS200	S_Shore	0.444±0.052	0.281±0.095	0.163	1.10E-05	6.51E-10	-0.298
cg08833432	20	60719824	*SS18L1*	5'UTR/1st intron	S_Shore	0.314±0.076	0.151±0.093	0.163	2.18E-04	3.64E-24	-0.471
cg11520395	1	227924115	*SNAP47*	5'UTR	S_Shore	0.443±0.123	0.28±0.098	0.163	6.76E-03	5.14E-07	-0.244
cg11520395	1	227924115	*JMJD4*	TSS1500	S_Shore	0.443±0.123	0.28±0.098	0.163	6.76E-03	9.28E-07	-0.239
cg00007221	1	43999020	*PTPRF*	5'UTR	S_Shore	0.568±0.071	0.405±0.127	0.163	3.89E-04	3.14E-08	-0.268
cg02249841	11	2397784	*CD81*	5'UTR/1st intron	N_Shore	0.474±0.143	0.312±0.128	0.163	1.71E-02	8.55E-07	-0.240
cg24631526	20	62367961	*LIME1*	5'UTR/1st exon	N_Shore	0.393±0.126	0.23±0.198	0.163	2.02E-02	1.26E-16	-0.392
cg14276120	9	71792936	*TJP2*	5'UTR	S_Shelf	0.718±0.062	0.555±0.168	0.163	1.50E-03	6.11E-18	-0.408
cg02365780	2	101437872	*NPAS2*	5'UTR/1st intron	S_Shore	0.429±0.117	0.267±0.121	0.163	5.97E-03	5.53E-18	-0.408
cg18998365	5	142781532	*NR3C1*	5'UTR/1st intron	N_Shore	0.29±0.077	0.127±0.053	0.163	2.74E-04	1.13E-06	-0.237
cg01492909	11	2397811	*CD81*	5'UTR/1st intron	N_Shore	0.536±0.131	0.374±0.164	0.163	1.56E-02	1.06E-09	-0.295
cg00270789	16	46919075	*GPT2*	5'UTR/1st exon	S_Shore	0.258±0.094	0.096±0.041	0.162	1.45E-03	1.27E-09	-0.293
cg06468619	19	50084552	*PRRG2*	TSS200	S_Shore	0.478±0.111	0.315±0.122	0.162	4.68E-03	2.38E-27	-0.499
cg16806077	6	31513198	*NFKBIL1*	TSS1500	S_Shelf	0.673±0.118	0.511±0.157	0.162	9.63E-03	3.96E-05	-0.201
cg19421368	20	45280288	*SLC13A3*	5'UTR	S_Shore	0.376±0.137	0.214±0.135	0.162	1.48E-02	7.54E-11	-0.314
cg24332710	1	46016173	*AKR1A1*	TSS1500	N_Shore	0.303±0.12	0.141±0.071	0.162	5.89E-03	8.77E-14	-0.356
cg10162914	4	170943016	*MFAP3L*	5'UTR/1st intron	N_Shelf	0.778±0.053	0.616±0.149	0.162	4.66E-04	2.54E-07	-0.251
cg25399743	8	27348177	*EPHX2*	TSS1500	N_Shore	0.258±0.1	0.096±0.039	0.162	2.23E-03	2.83E-08	-0.269
cg14234104	11	69931467	*ANO1*	TSS200	N_Shelf	0.709±0.082	0.547±0.199	0.162	7.42E-03	6.74E-14	-0.358
cg00410034	22	32869991	*FBXO7*	TSS1500	N_Shore	0.433±0.094	0.271±0.056	0.162	1.29E-03	5.82E-10	-0.299
cg05812089	11	19736648	*OC100126784*	TSS1500	S_Shore	0.329±0.088	0.168±0.117	0.162	1.14E-03	4.18E-07	-0.246
cg18529243	11	58911115	*FAM111A*	5'UTR	S_Shore	0.42±0.116	0.258±0.133	0.162	6.55E-03	2.71E-13	-0.350
cg01759155	14	103388797	*AMN*	TSS200	N_Shore	0.671±0.14	0.509±0.132	0.161	1.64E-02	4.97E-08	-0.265
cg01496970	7	102388500	*FAM185A*	TSS1500	N_Shore	0.395±0.075	0.234±0.067	0.161	2.01E-04	6.42E-07	-0.242
cg04700814	17	43224180	*HEXIM1*	TSS1500	N_Shore	0.752±0.092	0.591±0.233	0.161	1.86E-02	2.60E-05	-0.206
cg23940614	15	84115479	*SH3GL3*	TSS1500	N_Shore	0.479±0.089	0.318±0.195	0.161	7.98E-03	4.65E-06	-0.223
cg04996195	7	87105398	*ABCB4*	TSS1500	S_Shore	0.338±0.159	0.177±0.182	0.161	3.74E-02	9.12E-27	-0.494
cg07418892	6	52150456	*MCM3*	TSS1500	S_Shore	0.421±0.164	0.26±0.078	0.161	2.80E-02	9.98E-07	-0.238
cg12258811	1	7844735	*PER3*	5'UTR	Island	0.35±0.086	0.189±0.064	0.161	6.81E-04	1.48E-11	-0.324
cg02549492	5	95066568	*RHOBTB3*	TSS1500	N_Shore	0.277±0.098	0.116±0.061	0.161	1.80E-03	1.57E-13	-0.353
cg15662251	1	26197855	*PAQR7*	TSS200	N_Shelf	0.499±0.047	0.338±0.145	0.160	3.33E-04	3.62E-05	-0.202
cg13459303	7	150499276	*TMEM176B*	TSS1500	S_Shore	0.555±0.112	0.395±0.155	0.160	7.98E-03	3.58E-19	-0.421
cg22790931	7	39017455	*POU6F2*	TSS200	S_Shore	0.79±0.091	0.63±0.241	0.160	2.17E-02	1.91E-05	-0.209
cg06070002	1	45986120	*PRDX1*	5'UTR/1st intron	N_Shore	0.478±0.129	0.318±0.138	0.160	1.24E-02	3.84E-17	-0.399
cg13811417	9	25677628	*TUSC1*	1st exon/3'UTR	Island	0.244±0.128	0.084±0.082	0.160	9.33E-03	1.57E-15	-0.379
cg10110998	6	30639987	*DHX16*	5'UTR/1st intron	N_Shore	0.409±0.091	0.25±0.053	0.159	1.19E-03	5.97E-08	-0.263
cg06913600	2	43020682	*HAAO*	TSS1500	S_Shore	0.383±0.074	0.223±0.062	0.159	2.04E-04	1.88E-05	-0.209
cg22833478	11	2925623	*SLC22A18AS*	TSS1500	S_Shore	0.513±0.109	0.353±0.109	0.159	4.26E-03	1.64E-11	-0.324
cg15978899	6	30882994	*VARS2*	5'UTR	S_Shore	0.565±0.088	0.406±0.131	0.159	1.63E-03	3.93E-06	-0.225
cg21107581	19	49258805	*FGF21*	TSS200	S_Shelf	0.365±0.081	0.206±0.058	0.159	4.98E-04	1.08E-06	-0.238
cg08702413	5	125931275	*ALDH7A1*	TSS200	S_Shore	0.22±0.121	0.061±0.042	0.159	7.06E-03	6.02E-07	-0.243
cg05542967	2	234667988	*UGT1A1*	TSS1500	S_Shelf	0.868±0.076	0.709±0.185	0.159	4.76E-03	1.41E-10	-0.309
cg24110063	16	31439752	*COX6A2*	TSS200	S_Shore	0.566±0.1	0.407±0.211	0.159	1.56E-02	1.03E-19	-0.427
cg08886823	16	68270882	*ESRP2*	TSS1500	Island	0.278±0.102	0.12±0.038	0.159	2.86E-03	1.26E-05	-0.213
cg02578321	1	247275872	*C1orf229*	TSS200	N_Shelf	0.632±0.113	0.473±0.168	0.159	1.08E-02	1.39E-14	-0.367
cg22425466	9	140115987	*RNF208*	TSS1500	S_Shore	0.593±0.117	0.434±0.127	0.159	7.59E-03	4.84E-09	-0.283
cg22250546	19	33791370	*CEBPA*	1st exon/3'UTR	N_Shore	0.335±0.095	0.177±0.049	0.159	1.70E-03	2.73E-10	-0.305

cg17342132	5	142780254	*NR3C1*	5'UTR	N_Shore	0.526±0.09	0.368±0.163	0.158	4.30E-03	4.57E-10	-0.301
cg13886554	2	112895695	*FBLN7*	TSS1500	N_Shore	0.334±0.169	0.176±0.164	0.158	4.41E-02	5.40E-09	-0.282
cg25393494	17	17109936	*PLD6*	TSS1500	S_Shore	0.314±0.102	0.155±0.045	0.158	2.77E-03	1.93E-05	-0.209
cg26071135	11	843943	*TSPAN4*	5'UTR/1st intron	S_Shore	0.299±0.114	0.141±0.125	0.158	6.52E-03	4.88E-11	-0.317
cg19583655	17	79869752	*PCYT2*	TSS1500	Island	0.548±0.108	0.39±0.198	0.158	1.52E-02	2.89E-07	-0.249
cg13814664	13	36788926	*SOHLH2*	TSS200	Island	0.693±0.126	0.535±0.18	0.158	1.91E-02	2.58E-07	-0.251
cg16432009	22	37955936	*CDC42EP1*	TSS1500	N_Shore	0.64±0.112	0.482±0.123	0.158	5.73E-03	6.04E-13	-0.345
cg27639142	3	126072941	*KLF15*	5'UTR/1st intron	N_Shore	0.416±0.08	0.259±0.056	0.158	4.96E-04	1.20E-22	-0.457
cg07507951	1	247096025	*AHCTF1*	TSS1500	S_Shore	0.469±0.099	0.311±0.17	0.158	7.13E-03	1.15E-06	-0.237
cg03137177	21	44488499	*CBS*	5'UTR/1st exon	S_Shelf	0.343±0.113	0.186±0.069	0.157	4.89E-03	5.69E-10	-0.299
cg00414835	10	48439452	*GDF10*	TSS1500	S_Shore	0.577±0.128	0.42±0.213	0.157	2.99E-02	1.46E-07	-0.255
cg10516359	11	45827763	*SLC35C1*	1st exon	S_Shore	0.502±0.077	0.346±0.056	0.157	3.76E-04	5.08E-07	-0.244
cg24068468	10	5488535	*NET1*	1st exon	Island	0.322±0.111	0.165±0.06	0.157	4.51E-03	8.94E-07	-0.239
cg26163463	5	139926773	*EIF4EBP3*	TSS1500	N_Shore	0.437±0.091	0.28±0.048	0.157	1.39E-03	9.94E-06	-0.216
cg01483656	19	21264896	*ZNF714*	TSS200	N_Shore	0.383±0.133	0.226±0.134	0.156	1.55E-02	2.50E-05	-0.206
cg13488078	8	27469338	*CLU*	5'UTR/1st intron	N_Shelf	0.42±0.096	0.263±0.06	0.156	1.90E-03	6.24E-06	-0.221
cg20229931	21	47647656	*LSS*	5'UTR/1st intron	N_Shore	0.468±0.105	0.312±0.101	0.156	3.61E-03	1.81E-20	-0.435
cg07549924	4	54036	*ZNF595*	5'UTR/1st intron	S_Shore	0.438±0.076	0.281±0.098	0.156	3.64E-04	7.95E-18	-0.407
cg08950537	10	95361933	*RBP4*	TSS1500	S_Shore	0.277±0.081	0.121±0.042	0.156	6.80E-04	8.52E-07	-0.240
cg24326021	19	56188459	*EPN1*	5'UTR/1st exon	S_Shore	0.735±0.028	0.579±0.212	0.156	6.56E-03	2.29E-05	-0.207
cg12055459	2	97652489	*FAM178B*	TSS200	S_Shore	0.796±0.112	0.64±0.209	0.156	2.18E-02	1.04E-22	-0.458
cg07907506	3	100210582	*TMEM45A*	TSS1500	N_Shore	0.491±0.147	0.335±0.178	0.156	3.29E-02	4.60E-09	-0.284
cg24904992	17	77994779	*TBC1D16*	5'UTR/1st intron	N_Shelf	0.576±0.033	0.421±0.118	0.156	3.50E-05	3.21E-11	-0.319
cg05235151	6	35108114	*TCP11*	5'UTR	N_Shore	0.622±0.164	0.467±0.144	0.156	3.92E-02	5.58E-08	-0.264
cg03114748	20	35973098	*SRC*	5'UTR/1st exon	N_Shore	0.501±0.119	0.345±0.09	0.156	7.40E-03	1.84E-05	-0.210
cg01768131	17	79869861	*PCYT2*	TSS1500	S_Shore	0.441±0.114	0.286±0.144	0.156	8.93E-03	1.64E-09	-0.291
cg03849279	16	67751821	*GFOD2*	5'UTR/1st intron	N_Shore	0.713±0.127	0.558±0.205	0.156	2.78E-02	3.14E-18	-0.411
cg01378222	16	28622494	*SULT1A1*	TSS1500	N_Shelf	0.673±0.142	0.518±0.151	0.156	2.42E-02	2.51E-15	-0.377
cg12712768	12	121416315	*HNF1A*	TSS200	N_Shore	0.412±0.092	0.256±0.057	0.156	1.48E-03	3.43E-25	-0.481
cg17469265	4	39183353	*WDR19*	TSS1500	N_Shore	0.366±0.12	0.211±0.063	0.155	7.34E-03	8.58E-08	-0.260
cg11770325	17	42081179	*NAGS*	TSS1500	N_Shore	0.74±0.045	0.585±0.136	0.155	2.40E-04	1.01E-31	-0.534
cg07620544	16	31225357	*TRIM72*	5'UTR/1st exon	N_Shore	0.685±0.068	0.53±0.213	0.155	1.07E-02	4.15E-06	-0.225
cg15062470	2	27718710	*GCKR*	TSS1500	S_Shore	0.389±0.096	0.234±0.06	0.155	1.96E-03	9.95E-10	-0.295
cg15062470	2	27718710	*FNDC4*	TSS1500	S_Shore	0.389±0.096	0.234±0.06	0.155	1.96E-03	1.48E-09	-0.292
cg20988073	3	125900093	*ALDH1L1*	TSS1500	S_Shore	0.313±0.116	0.158±0.037	0.155	6.55E-03	1.88E-14	-0.365
cg07869651	5	177581603	*NHP2*	TSS1500	S_Shore	0.46±0.121	0.305±0.133	0.155	1.06E-02	5.91E-07	-0.243
cg13659813	19	39322968	*ECH1*	TSS1500	S_Shore	0.254±0.078	0.099±0.035	0.155	6.10E-04	9.96E-09	-0.278
cg12113740	18	52625368	*CCDC68*	5'UTR/1st intron	N_Shore	0.62±0.16	0.465±0.164	0.155	4.08E-02	3.36E-19	-0.422
cg26760212	20	62601743	*ZNF512B*	TSS1500	S_Shore	0.381±0.1	0.227±0.093	0.154	2.67E-03	2.00E-05	-0.208
cg02034910	16	67207062	*NOL3*	5'UTR	S_Shelf	0.605±0.074	0.451±0.131	0.154	9.79E-04	1.30E-06	-0.236
cg05017199	7	150497065	*TMEM176B*	5'UTR/1st intron	Island	0.26±0.1	0.106±0.048	0.154	2.84E-03	2.42E-10	-0.305
cg05017199	7	150497065	*TMEM176A*	TSS1500	Island	0.26±0.1	0.106±0.048	0.154	2.84E-03	3.49E-10	-0.303
cg00171459	7	5554215	*FBXL18*	TSS1500	S_Shore	0.526±0.092	0.372±0.07	0.154	1.53E-03	3.23E-07	-0.249
cg27650171	6	30881579	*VARS2*	TSS1500	Island	0.259±0.068	0.105±0.042	0.154	1.71E-04	9.77E-10	-0.295
cg21086870	15	41836937	*RPAP1*	TSS1500	S_Shore	0.665±0.13	0.511±0.242	0.154	4.69E-02	2.53E-07	-0.251
cg17778313	19	42811489	*PRR19*	5'UTR/1st intron	S_Shore	0.746±0.075	0.592±0.197	0.154	8.45E-03	6.73E-11	-0.314
cg00946598	1	44117093	*KDM4A*	5'UTR/1st intron	S_Shore	0.567±0.15	0.413±0.164	0.154	3.36E-02	3.63E-09	-0.285
cg05731808	2	173293392	*ITGA6*	5'UTR/1st intron	S_Shore	0.334±0.121	0.181±0.066	0.154	8.45E-03	1.88E-06	-0.232
cg09596674	3	46607350	*LRRC2*	5'UTR/1st intron	Island	0.351±0.106	0.197±0.127	0.154	5.36E-03	6.35E-22	-0.450
cg07700644	11	116663804	*APOA5*	TSS1500	S_Shelf	0.554±0.085	0.4±0.148	0.153	3.06E-03	1.16E-09	-0.294
cg02528159	16	3021099	*PAQR4*	5'UTR/1st intron	S_Shore	0.406±0.078	0.253±0.067	0.153	4.33E-04	1.20E-06	-0.237
cg27531553	1	6615604	*NOL9*	TSS1500	S_Shore	0.612±0.066	0.459±0.138	0.153	8.17E-04	1.96E-05	-0.209
cg04306507	14	55594613	*LGALS3*	TSS1500	N_Shore	0.55±0.106	0.397±0.145	0.153	7.11E-03	5.06E-06	-0.223
cg04270652	16	28608894	*SULT1A2*	TSS1500	N_Shelf	0.582±0.168	0.429±0.12	0.153	4.13E-02	5.38E-18	-0.408
cg07804260	11	2397977	*CD81*	5'UTR/1st intron	N_Shore	0.521±0.166	0.368±0.158	0.153	4.61E-02	2.69E-14	-0.363
cg18229134	5	176516040	*FGFR4*	5'UTR/1st intron	S_Shore	0.527±0.155	0.374±0.098	0.153	2.88E-02	1.11E-20	-0.437
cg14101500	11	2925594	*SLC22A18AS*	TSS1500	S_Shore	0.419±0.085	0.266±0.056	0.153	9.73E-04	1.09E-11	-0.326
cg10763288	11	71158925	*DHCR7*	5'UTR/1st intron	N_Shore	0.334±0.076	0.182±0.046	0.153	4.51E-04	2.19E-06	-0.231
cg25017060	14	103389017	*AMN*	5'UTR/1st exon	Island	0.297±0.071	0.144±0.043	0.153	2.57E-04	1.07E-07	-0.258
cg08376824	20	5987738	*CRLS1*	5'UTR/1st intron	S_Shore	0.303±0.068	0.151±0.044	0.153	1.78E-04	6.13E-09	-0.281
cg05463325	1	229406534	*RAB4A*	TSS1500	N_Shore	0.421±0.081	0.268±0.064	0.153	6.47E-04	8.38E-10	-0.296
cg05925894	1	85931301	*DDAH1*	5'UTR	S_Shore	0.295±0.073	0.143±0.042	0.152	3.40E-04	1.54E-07	-0.255
cg25882597	20	39969542	*LPIN3*	1st exon	S_Shore	0.36±0.082	0.207±0.062	0.152	7.08E-04	6.18E-15	-0.372
cg10672416	12	123718706	*C12orf65*	5'UTR/1st intron	S_Shore	0.408±0.11	0.255±0.12	0.152	6.58E-03	8.52E-09	-0.279
cg05745457	4	164394816	*TKTL2*	1st exon	Island	0.816±0.079	0.664±0.18	0.152	6.31E-03	1.26E-05	-0.213
cg26069068	8	125741265	*MTSS1*	TSS1500	Island	0.392±0.09	0.24±0.076	0.152	1.39E-03	1.74E-05	-0.210
cg14101638	12	121416612	*HNF1A*	1st exon	Island	0.493±0.106	0.342±0.058	0.152	4.33E-03	6.83E-29	-0.512
cg02078292	11	2397655	*CD81*	5'UTR/1st intron	N_Shore	0.519±0.155	0.368±0.19	0.152	4.72E-02	1.40E-17	-0.404
cg12571493	4	174256943	*HMGB2*	TSS1500	S_Shore	0.362±0.093	0.21±0.084	0.152	1.88E-03	2.98E-09	-0.287
cg17716930	19	51017331	*ASPDH*	TSS200	S_Shelf	0.371±0.127	0.22±0.09	0.151	1.17E-02	1.86E-05	-0.209
cg10857558	4	90035421	*TIGD2*	1st exon	S_Shelf	0.343±0.121	0.192±0.053	0.151	9.23E-03	7.00E-07	-0.242
cg02047661	3	51976883	*PARP3*	5'UTR/1st intron	S_Shore	0.359±0.089	0.208±0.046	0.151	1.55E-03	1.01E-06	-0.238
cg23916044	11	62472988	*BSCL2*	5'UTR	N_Shelf	0.553±0.128	0.402±0.132	0.151	1.54E-02	8.43E-17	-0.395
cg13423770	1	156338775	*RHBG*	TSS1500	Island	0.36±0.074	0.209±0.07	0.151	2.94E-04	9.70E-09	-0.278
cg06916670	1	202829945	*LOC148709*	TSS1500	N_Shore	0.511±0.108	0.359±0.17	0.151	1.26E-02	1.19E-25	-0.485
cg01566404	1	204121925	*ETNK2*	TSS1500	S_Shore	0.73±0.074	0.579±0.169	0.151	4.15E-03	8.03E-22	-0.449
cg18344745	4	157997079	*GLRB*	TSS1500	N_Shore	0.471±0.143	0.321±0.167	0.151	3.18E-02	7.49E-07	-0.241
cg14595683	8	77592946	*ZFHX4*	TSS1500	S_Shelf	0.331±0.104	0.181±0.081	0.151	3.86E-03	1.98E-17	-0.402
cg00213123	15	75019070	*CYP1A1*	TSS1500	Island	0.251±0.107	0.101±0.034	0.151	5.01E-03	3.32E-05	-0.203
cg13891189	9	116164001	*ALAD*	TSS1500	S_Shore	0.325±0.062	0.174±0.058	0.151	6.95E-05	2.25E-15	-0.377
cg24649332	20	45280359	*SLC13A3*	5'UTR	S_Shore	0.48±0.154	0.329±0.113	0.151	3.12E-02	3.12E-07	-0.249
cg20968678	2	216948982	*TMEM169*	5'UTR	S_Shelf	0.614±0.099	0.463±0.175	0.150	1.06E-02	7.41E-21	-0.439
cg18908681	5	115177683	*ATG12*	TSS200	Island	0.449±0.036	0.299±0.087	0.150	2.09E-06	1.31E-07	-0.256
cg00363750	19	51017311	*ASPDH*	TSS200	S_Shelf	0.333±0.118	0.183±0.069	0.150	7.99E-03	8.79E-07	-0.239
cg27578754	7	11012967	*PHF14*	TSS1500	N_Shore	0.513±0.11	0.363±0.072	0.150	5.65E-03	6.38E-09	-0.281
cg05346140	17	40275057	*HSPB9*	1st exon	Island	0.516±0.12	0.366±0.101	0.150	9.58E-03	1.18E-16	-0.393
cg16267850	1	46859791	*FAAH*	TSS200	Island	0.244±0.096	0.094±0.035	0.150	2.78E-03	1.53E-10	-0.309

cg12893736	17	72429636	*GPRC5C*	5'UTR/1st intron	S_Shore	0.479±0.092	0.329±0.138	0.150	3.87E-03	3.85E-23	-0.462
cg19623519	1	247276096	*C1orf229*	TSS1500	N_Shelf	0.71±0.087	0.559±0.162	0.150	5.60E-03	1.68E-09	-0.291
cg24657085	17	37860344	*ERBB2*	5'UTR	S_Shelf	0.651±0.106	0.501±0.187	0.150	1.62E-02	6.29E-22	-0.450
cg25526519	15	91499369	*RCCD1*	5'UTR	N_Shore	0.564±0.144	0.414±0.177	0.150	3.69E-02	1.48E-12	-0.339
cg07100411	11	64884229	*ZNHIT2*	1st exon	S_Shelf	0.381±0.086	0.232±0.057	0.150	1.20E-03	6.38E-07	-0.242
cg12729838	8	27469186	*CLU*	1st exon	N_Shelf	0.409±0.095	0.259±0.059	0.150	2.33E-03	4.99E-06	-0.223
cg22707016	15	41098847	*ZFYVE19*	TSS1500	N_Shore	0.709±0.083	0.56±0.117	0.149	1.53E-03	1.50E-05	-0.211
cg05098551	2	179344725	*PLEKHA3*	TSS1500	N_Shore	0.35±0.106	0.201±0.126	0.149	6.43E-03	7.66E-06	-0.218
cg05437692	4	149362435	*NR3C2*	5'UTR/1st intron	N_Shore	0.311±0.101	0.162±0.04	0.149	3.85E-03	1.14E-07	-0.258
cg13410390	1	155232677	*SCAMP3*	TSS1500	S_Shore	0.622±0.091	0.472±0.152	0.149	5.40E-03	2.45E-18	-0.412
cg25417405	15	50474129	*SLC27A2*	TSS1500	N_Shore	0.272±0.085	0.123±0.047	0.149	1.24E-03	1.34E-06	-0.236
cg13503413	10	60937257	*PHYHIPL*	5'UTR/1st exon	S_Shore	0.256±0.109	0.107±0.028	0.149	5.92E-03	7.21E-08	-0.262
cg08095700	3	184972153	*EHHADH*	TSS1500	S_Shore	0.278±0.091	0.13±0.096	0.149	1.98E-03	7.51E-07	-0.241
cg18808261	3	18464935	*SATB1*	5'UTR	N_Shelf	0.426±0.148	0.278±0.143	0.149	3.24E-02	1.52E-19	-0.425
cg14295062	9	139949108	*ENTPD2*	TSS1500	S_Shore	0.411±0.095	0.263±0.05	0.148	2.48E-03	2.45E-05	-0.206
cg10559803	1	178693897	*RALGPS2*	TSS1500	N_Shore	0.318±0.083	0.17±0.057	0.148	9.78E-04	9.44E-10	-0.296
cg03547797	11	22689644	*GAS2*	5'UTR/1st intron	S_Shore	0.33±0.091	0.181±0.113	0.148	2.55E-03	1.53E-23	-0.465
cg22590775	19	49891494	*CCDC155*	5'UTR/1st exon	Island	0.515±0.112	0.366±0.171	0.148	1.60E-02	8.73E-06	-0.217
cg09423181	17	55164762	*AKAP1*	5'UTR	S_Shore	0.694±0.158	0.546±0.161	0.148	4.63E-02	3.97E-11	-0.318
cg22446000	1	247275998	*C1orf229*	TSS1500	N_Shelf	0.612±0.096	0.464±0.169	0.148	9.72E-03	2.71E-15	-0.376
cg02310386	12	103311761	*PAH*	5'UTR/1st intron	S_Shore	0.309±0.073	0.161±0.043	0.148	4.40E-04	3.43E-08	-0.268
cg00244776	6	30883192	*VARS2*	5'UTR	S_Shore	0.472±0.073	0.325±0.128	0.147	1.23E-03	2.02E-07	-0.253
cg25027125	14	35182801	*CFL2*	5'UTR/1st intron	N_Shore	0.27±0.102	0.122±0.065	0.147	3.89E-03	1.61E-24	-0.474
cg10840135	22	42526619	*CYP2D6*	1st exon	S_Shore	0.462±0.071	0.315±0.097	0.147	3.87E-04	1.12E-10	-0.311
cg12464638	11	844400	*TSPAN4*	5'UTR/1st intron	S_Shore	0.381±0.108	0.234±0.146	0.147	1.00E-02	9.11E-13	-0.342
cg04338134	16	66969401	*CES2*	5'UTR/1st exon	N_Shore	0.287±0.081	0.139±0.054	0.147	9.00E-04	9.25E-16	-0.382
cg26104196	1	159914191	*IGSF9*	5'UTR/1st intron	N_Shore	0.524±0.112	0.377±0.16	0.147	1.46E-02	3.72E-14	-0.361
cg02449103	6	31627711	*C6orf47*	1st exon	N_Shore	0.246±0.101	0.099±0.05	0.147	3.97E-03	3.91E-11	-0.318
cg15803221	17	38473166	*RARA*	5'UTR/1st intron	Island	0.506±0.078	0.359±0.089	0.147	6.89E-04	1.69E-07	-0.254
cg27539986	1	6664103	*KLHL21*	TSS1500	S_Shore	0.21±0.091	0.063±0.036	0.147	2.20E-03	3.68E-05	-0.202
cg05955436	20	17660838	*RRBP1*	5'UTR/1st intron	N_Shore	0.306±0.147	0.159±0.115	0.147	2.99E-02	6.00E-12	-0.330
cg04525852	4	125632538	*ANKRD50*	5'UTR/1st intron	N_Shore	0.32±0.115	0.173±0.159	0.147	1.61E-02	4.67E-09	-0.284
cg18068140	6	18123164	*NHLRC1*	TSS1500	S_Shore	0.457±0.086	0.311±0.143	0.146	3.95E-03	5.81E-13	-0.345
cg24526899	14	54424149	*BMP4*	TSS1500	S_Shore	0.673±0.074	0.527±0.215	0.146	1.73E-02	2.07E-22	-0.455
cg24203376	10	115938868	*TDRD1*	TSS1500	N_Shore	0.744±0.049	0.598±0.186	0.146	5.38E-03	4.15E-08	-0.266
cg07940714	19	40024949	*EID2B*	TSS1500	S_Shore	0.638±0.06	0.492±0.127	0.146	5.35E-04	1.32E-05	-0.213
cg25170091	17	202716	*RPH3AL*	TSS200	N_Shelf	0.236±0.081	0.09±0.032	0.146	1.18E-03	2.33E-05	-0.207
cg12352315	17	74069707	*SRP68*	TSS1500	S_Shore	0.778±0.078	0.632±0.202	0.146	1.40E-02	3.98E-05	-0.201
cg24812103	8	101735630	*PABPC1*	TSS1500	S_Shore	0.401±0.137	0.256±0.181	0.146	3.69E-02	1.34E-09	-0.293
cg14518948	5	68484768	*CENPH*	TSS1500	N_Shore	0.47±0.121	0.324±0.146	0.146	1.74E-02	4.21E-05	-0.200
cg04104489	1	64063351	*PGM1*	5'UTR/1st intron	S_Shelf	0.708±0.048	0.562±0.152	0.146	1.28E-03	1.35E-18	-0.415
cg15162922	5	132162758	*SHROOM1*	5'UTR	S_Shore	0.81±0.054	0.665±0.156	0.146	1.87E-03	2.49E-32	-0.538
cg01394199	12	121416547	*HNF1A*	5'UTR/1st exon	Island	0.554±0.108	0.408±0.056	0.145	6.10E-03	9.74E-23	-0.458
cg20248866	17	79869748	*PCYT2*	TSS1500	Island	0.416±0.125	0.27±0.161	0.145	2.31E-02	2.73E-07	-0.250
cg19409187	6	123109903	*SMPDL3A*	TSS1500	N_Shore	0.269±0.101	0.123±0.04	0.145	4.27E-03	2.89E-05	-0.204
cg25823085	7	87105416	*ABCB4*	TSS1500	S_Shore	0.427±0.133	0.281±0.195	0.145	3.95E-02	2.38E-29	-0.516
cg23596673	6	24491098	*GPLD1*	TSS1500	N_Shelf	0.809±0.061	0.664±0.251	0.145	3.11E-02	1.29E-06	-0.236
cg27119222	11	2720229	*KCNQ1OT1*	1st exon	N_Shore	0.506±0.057	0.361±0.176	0.145	4.39E-03	5.33E-10	-0.300
cg16988185	17	77705025	*ENPP7*	1st exon	N_Shelf	0.574±0.103	0.429±0.086	0.145	4.78E-03	2.45E-23	-0.464
cg24984312	11	116707209	*APOA1*	5'UTR	S_Shore	0.367±0.108	0.222±0.041	0.145	6.16E-03	5.03E-06	-0.223
cg01953240	16	1129666	*SSTR5*	1st exon	Island	0.847±0.068	0.702±0.179	0.145	6.44E-03	6.47E-09	-0.281
cg25598083	14	74034893	*ACOT2*	TSS1500	N_Shore	0.397±0.114	0.252±0.08	0.145	8.51E-03	1.89E-06	-0.232
cg26989053	16	15148607	*NTAN1*	5'UTR/1st intron	N_Shore	0.614±0.134	0.469±0.132	0.145	2.34E-02	3.93E-09	-0.285
cg27395922	11	50257633	*LOC441601*	1st exon	Island	0.576±0.099	0.431±0.171	0.145	1.27E-02	9.55E-12	-0.327
cg04862321	6	30580928	*PPP1R10*	5'UTR	N_Shelf	0.32±0.088	0.175±0.091	0.145	1.77E-03	2.32E-05	-0.207
cg02124724	21	46875142	*COL18A1*	TSS1500	N_Shore	0.469±0.095	0.324±0.059	0.145	2.92E-03	2.73E-09	-0.288
cg23591595	17	18528638	*CCDC144B*	1st exon	Island	0.754±0.082	0.609±0.1	0.145	1.29E-03	2.48E-10	-0.305
cg10661769	6	30881484	*VARS2*	TSS1500	N_Shore	0.227±0.109	0.082±0.043	0.145	6.69E-03	5.39E-06	-0.222
cg01035826	11	125494680	*CHEK1*	TSS1500	N_Shore	0.642±0.109	0.497±0.2	0.145	2.58E-02	1.20E-09	-0.294
cg09715285	2	220108267	*GLB1L*	1st exon	N_Shore	0.443±0.111	0.299±0.16	0.144	1.57E-02	2.29E-11	-0.322
cg01084740	14	61748243	*TMEM30B*	5'UTR/1st exon	S_Shore	0.343±0.086	0.199±0.053	0.144	1.52E-03	1.37E-13	-0.354
cg27451508	17	19652257	*ALDH3A1*	TSS1500	S_Shelf	0.695±0.149	0.551±0.161	0.144	4.28E-02	2.17E-18	-0.413
cg07320140	16	75150611	*LDHD*	1st exon	S_Shore	0.249±0.076	0.105±0.04	0.144	7.31E-04	1.62E-05	-0.211
cg23906067	6	31939112	*STK19*	TSS1500	N_Shore	0.383±0.088	0.239±0.067	0.144	1.81E-03	8.77E-07	-0.239
cg27455017	7	150037988	*RARRES2*	5'UTR/1st intron	Island	0.391±0.064	0.247±0.051	0.144	1.42E-04	7.51E-06	-0.219
cg12598950	17	77704916	*ENPP7*	1st exon	N_Shelf	0.413±0.107	0.269±0.078	0.144	6.26E-03	2.72E-12	-0.335
cg18664866	14	75389200	*RPS6KL1*	TSS200	N_Shore	0.788±0.048	0.644±0.151	0.144	1.37E-03	2.26E-23	-0.464
cg00910127	6	44281749	*AARS2*	TSS1500	S_Shore	0.295±0.079	0.151±0.053	0.144	8.50E-04	4.29E-14	-0.361
cg21535580	1	204121899	*ETNK2*	TSS1500	S_Shore	0.584±0.092	0.441±0.141	0.144	5.75E-03	4.02E-30	-0.522
cg19632760	8	71581729	*XKR9*	5'UTR/1st exon	S_Shore	0.34±0.117	0.197±0.047	0.144	9.99E-03	3.84E-13	-0.348
cg19632760	8	71581729	*LACTB2*	TSS1500	S_Shore	0.34±0.117	0.197±0.047	0.144	9.99E-03	2.79E-08	-0.269
cg03337886	13	110436740	*IRS2*	1st exon	Island	0.639±0.095	0.496±0.162	0.144	9.80E-03	5.43E-09	-0.282
cg18555698	11	12699172	*TEAD1*	5'UTR	S_Shelf	0.741±0.119	0.598±0.216	0.144	4.04E-02	1.20E-09	-0.294
cg00206463	6	30179435	*TRIM26*	5'UTR/1st intron	N_Shore	0.308±0.071	0.164±0.057	0.143	3.73E-04	4.20E-17	-0.398
cg06848073	1	11713540	*FBXO44*	TSS1500	N_Shore	0.511±0.065	0.368±0.147	0.143	2.07E-03	7.78E-14	-0.357
cg20845050	8	146053666	*ZNF7*	5'UTR/1st intron	S_Shore	0.333±0.134	0.19±0.063	0.143	1.95E-02	3.31E-15	-0.375
cg17797591	17	63555244	*AXIN2*	5'UTR/1st intron	N_Shore	0.391±0.136	0.247±0.187	0.143	4.11E-02	2.28E-23	-0.464
cg12554573	3	51976667	*PARP3*	5'UTR/1st exon	S_Shore	0.351±0.099	0.208±0.047	0.143	4.14E-03	3.35E-11	-0.319
cg08784247	12	81329808	*LIN7A*	5'UTR/1st intron	N_Shore	0.294±0.096	0.151±0.047	0.143	3.47E-03	9.55E-06	-0.216
cg15711987	10	99393886	*MORN4*	5'UTR/1st intron	S_Shore	0.566±0.087	0.424±0.228	0.143	2.97E-02	3.32E-08	-0.268
cg13841742	4	48909110	*OCIAD2*	TSS1500	S_Shore	0.353±0.09	0.211±0.043	0.142	2.43E-03	8.38E-27	-0.495
cg27191921	22	43042910	*CYB5R3*	5'UTR/1st exon	N_Shelf	0.371±0.1	0.229±0.109	0.142	5.56E-03	1.96E-05	-0.209
cg15890754	2	173294093	*ITGA6*	5'UTR/1st intron	S_Shore	0.291±0.085	0.148±0.1	0.142	1.84E-03	2.11E-09	-0.290
cg05256304	11	116969870	*SIK3*	TSS1500	S_Shore	0.305±0.113	0.162±0.115	0.142	1.10E-02	1.77E-08	-0.273
cg16975639	2	233501753	*EFHD1*	5'UTR/1st intron	S_Shelf	0.287±0.058	0.145±0.123	0.142	5.49E-04	5.08E-06	-0.223
cg15008287	11	72492282	*STARD10*	5'UTR	Island	0.328±0.106	0.186±0.068	0.142	6.16E-03	1.78E-05	-0.210
cg18261491	1	46859780	*FAAH*	TSS200	Island	0.333±0.089	0.191±0.056	0.142	2.16E-03	3.90E-06	-0.225

cg21433933	5	132163083	*SHROOM1*	5'UTR	S_Shore	0.74±0.054	0.598±0.126	0.142	4.99E-04	6.20E-27	-0.496
cg14404746	9	140116137	*RNF208*	TSS1500	S_Shore	0.326±0.104	0.185±0.071	0.142	5.63E-03	2.28E-05	-0.207
cg06788278	1	178693891	*RALGPS2*	TSS1500	N_Shore	0.318±0.079	0.176±0.052	0.142	9.41E-04	3.20E-09	-0.286
cg15821095	3	51428079	*RBM15B*	TSS1500	N_Shelf	0.542±0.081	0.4±0.163	0.142	6.82E-03	6.30E-08	-0.263
cg03449125	11	76779064	*CAPN5*	5'UTR/1st intron	S_Shore	0.535±0.137	0.393±0.134	0.142	2.92E-02	1.32E-25	-0.484
cg10488031	13	110437561	*IRS2*	1st exon	Island	0.4±0.098	0.258±0.059	0.142	3.95E-03	1.66E-08	-0.274
cg08204159	19	54642290	*CNOT3*	5'UTR/1st intron	Island	0.33±0.103	0.188±0.075	0.141	5.45E-03	8.07E-06	-0.218
cg01057656	19	45281140	*CBLC*	5'UTR/1st exon	Island	0.178±0.088	0.037±0.03	0.141	2.44E-03	6.63E-07	-0.242
cg24058132	14	88459866	*GALC*	5'UTR/1st exon	S_Shore	0.383±0.144	0.242±0.128	0.141	3.40E-02	2.54E-13	-0.350
cg03035746	2	74727286	*LBX2*	TSS1500	N_Shelf	0.857±0.041	0.715±0.187	0.141	6.31E-03	1.90E-33	-0.546
cg00258873	6	30312687	*RPP21*	TSS1500	N_Shore	0.357±0.119	0.216±0.077	0.141	1.22E-02	7.40E-09	-0.280
cg16717713	14	100069657	*CCDC85C*	1st exon	Island	0.269±0.089	0.128±0.043	0.141	2.36E-03	3.48E-05	-0.202
cg12413156	20	62368256	*LIME1*	5'UTR/1st intron	N_Shore	0.582±0.117	0.44±0.188	0.141	3.00E-02	1.41E-25	-0.484
cg07150062	14	104552032	*ASPG*	5'UTR/1st exon	Island	0.229±0.122	0.088±0.105	0.141	1.52E-02	7.45E-07	-0.241
cg22534105	1	12078836	*MIIP*	TSS1500	N_Shore	0.676±0.139	0.535±0.187	0.141	4.67E-02	3.18E-06	-0.227
cg10761315	14	104552034	*ASPG*	5'UTR/1st exon	Island	0.245±0.116	0.104±0.075	0.141	1.06E-02	5.28E-06	-0.222
cg18849169	5	150399781	*GPX3*	TSS200	N_Shore	0.558±0.135	0.417±0.151	0.141	3.19E-02	7.67E-06	-0.218
cg16913124	3	50338253	*HYAL3*	TSS1500	S_Shore	0.861±0.018	0.721±0.168	0.141	2.52E-03	4.24E-07	-0.246
cg14977069	20	62367698	*LIME1*	5'UTR/1st intron	N_Shore	0.666±0.079	0.526±0.16	0.141	6.36E-03	4.39E-22	-0.451
cg20307496	22	37955927	*CDC42EP1*	TSS1500	N_Shore	0.574±0.102	0.433±0.12	0.141	7.36E-03	7.68E-12	-0.329
cg16469223	3	184095245	*THPO*	5'UTR	N_Shelf	0.616±0.066	0.476±0.134	0.140	1.52E-03	2.53E-29	-0.515
cg15117516	10	72576586	*SGPL1*	5'UTR	S_Shore	0.371±0.11	0.23±0.104	0.140	9.34E-03	1.26E-05	-0.213
cg20987431	8	124284393	*ZHX1*	5'UTR/1st intron	N_Shore	0.306±0.132	0.166±0.066	0.140	2.00E-02	7.55E-14	-0.357
cg09523691	5	115178810	*AP3S1*	5'UTR/1st intron	S_Shore	0.273±0.077	0.133±0.043	0.140	8.94E-04	1.36E-07	-0.256
cg09523691	5	115178810	*ATG12*	TSS1500	S_Shore	0.273±0.077	0.133±0.043	0.140	8.94E-04	9.13E-07	-0.239
cg21601837	3	125900065	*ALDH1L1*	TSS1500	S_Shore	0.234±0.13	0.094±0.028	0.140	1.86E-02	1.43E-14	-0.367
cg24754336	20	61494027	*TCFL5*	TSS1500	S_Shore	0.425±0.133	0.285±0.138	0.140	2.84E-02	6.87E-06	-0.220
cg08830139	5	140013652	*CD14*	TSS1500	S_Shore	0.564±0.108	0.424±0.167	0.140	1.92E-02	4.51E-09	-0.284
cg26233209	5	115178764	*AP3S1*	5'UTR/1st intron	S_Shore	0.253±0.073	0.113±0.045	0.140	6.44E-04	2.10E-06	-0.231
cg26233209	5	115178764	*ATG12*	TSS1500	S_Shore	0.253±0.073	0.113±0.045	0.140	6.44E-04	1.53E-05	-0.211
cg16190718	6	31939106	*STK19*	TSS1500	N_Shore	0.387±0.084	0.247±0.068	0.140	1.61E-03	3.08E-05	-0.204
cg15142477	18	43915662	*RNF165*	5'UTR/1st intron	S_Shore	0.391±0.064	0.251±0.158	0.139	3.97E-03	7.47E-08	-0.261
cg25341653	16	67233277	*ELMO3*	1st exon	Island	0.623±0.075	0.484±0.153	0.139	4.71E-03	3.70E-17	-0.399
cg14322224	1	85931226	*DDAH1*	5'UTR	S_Shore	0.262±0.067	0.123±0.04	0.139	3.67E-04	6.24E-07	-0.243
cg00622166	16	46919021	*GPT2*	TSS200	S_Shore	0.27±0.075	0.13±0.036	0.139	8.95E-04	2.23E-15	-0.377
cg07792006	1	20511417	*UBXN10*	TSS1500	N_Shore	0.587±0.053	0.448±0.083	0.139	4.80E-05	8.11E-19	-0.418
cg02478149	22	38219117	*GALR3*	TSS1500	N_Shore	0.402±0.076	0.263±0.089	0.139	9.19E-04	2.20E-05	-0.207
cg23661013	12	121415506	*HNF1A*	TSS1500	N_Shore	0.506±0.15	0.367±0.15	0.139	4.76E-02	1.39E-09	-0.293
cg00978570	1	16303299	*ZBTB17*	TSS1500	S_Shore	0.513±0.055	0.374±0.144	0.139	1.67E-03	5.25E-12	-0.331
cg15349474	11	125494878	*CHEK1*	TSS1500	N_Shore	0.674±0.1	0.535±0.169	0.138	1.67E-02	1.43E-06	-0.235
cg08733482	4	111395729	*ENPEP*	TSS1500	N_Shore	0.469±0.153	0.331±0.123	0.138	4.56E-02	1.00E-13	-0.356
cg19424531	8	65711522	*CYP7B1*	TSS200	Island	0.216±0.127	0.078±0.085	0.138	1.83E-02	5.75E-14	-0.359
cg10628205	1	61547131	*NFIA*	TSS1500	N_Shore	0.243±0.085	0.105±0.035	0.138	2.09E-03	1.06E-12	-0.341
cg26162794	17	18528568	*CCDC144B*	1st exon	Island	0.818±0.074	0.68±0.141	0.138	3.30E-03	8.09E-12	-0.328
cg04871131	7	94954202	*PON1*	TSS200	S_Shore	0.489±0.126	0.351±0.189	0.138	4.08E-02	2.93E-17	-0.400
cg18428265	2	101437805	*NPAS2*	5'UTR/1st intron	S_Shore	0.384±0.116	0.246±0.106	0.138	1.34E-02	2.28E-16	-0.389
cg24757346	19	35629022	*FXYD1*	TSS1500	N_Shelf	0.691±0.089	0.554±0.178	0.138	1.50E-02	1.59E-24	-0.475
cg10534904	14	65438215	*RAB15*	5'UTR/1st intron	N_Shore	0.286±0.098	0.149±0.085	0.138	4.87E-03	3.13E-05	-0.204
cg10549831	10	5488366	*NET1*	TSS200	N_Shore	0.234±0.094	0.097±0.04	0.137	4.01E-03	1.17E-06	-0.237
cg10589385	1	150898437	*SETDB1*	TSS1500	N_Shore	0.396±0.107	0.258±0.15	0.137	1.58E-02	1.81E-08	-0.273
cg04779796	3	11037353	*SLC6A1*	5'UTR/1st intron	S_Shore	0.629±0.096	0.492±0.162	0.137	1.35E-02	9.42E-31	-0.526
cg22517740	1	228870629	*RHOU*	TSS200	N_Shore	0.293±0.078	0.156±0.081	0.137	1.09E-03	8.34E-15	-0.370
cg24868305	1	225998730	*EPHX1*	5'UTR/1st intron	S_Shore	0.26±0.088	0.123±0.045	0.137	2.58E-03	1.00E-11	-0.327
cg25477769	12	121416566	*HNF1A*	5'UTR/1st exon	Island	0.574±0.098	0.437±0.045	0.137	4.88E-03	3.53E-23	-0.462
cg07697276	4	53025	*ZNF595*	TSS200	N_Shore	0.421±0.057	0.284±0.084	0.137	1.02E-04	3.83E-26	-0.489
cg13696409	9	73030641	*KLF9*	TSS1500	S_Shore	0.811±0.034	0.675±0.123	0.137	2.55E-04	6.75E-13	-0.344
cg00259046	11	50257771	*LOC441601*	TSS200	Island	0.559±0.118	0.423±0.131	0.136	1.93E-02	1.88E-14	-0.365
cg07190921	16	1845113	*IGFALS*	TSS1500	S_Shelf	0.796±0.125	0.659±0.19	0.136	4.25E-02	4.78E-10	-0.301
cg24516516	8	19678013	*INTS10*	5'UTR	S_Shelf	0.691±0.126	0.555±0.189	0.136	4.30E-02	1.20E-11	-0.326
cg14893129	17	78152051	*CARD14*	5'UTR	N_Shelf	0.813±0.045	0.677±0.146	0.136	1.55E-03	5.73E-06	-0.221
cg25463749	6	119401102	*FAM184A*	5'UTR/1st intron	S_Shore	0.576±0.083	0.439±0.14	0.136	5.52E-03	4.72E-10	-0.301
cg01455178	1	205648187	*SLC45A3*	5'UTR/1st intron	N_Shore	0.595±0.083	0.459±0.21	0.136	2.66E-02	1.72E-20	-0.435
cg18898325	22	42394382	*WBP2NL*	TSS1500	N_Shore	0.411±0.076	0.276±0.111	0.136	1.78E-03	6.24E-10	-0.299
cg08535938	1	161128985	*USP21*	TSS1500	N_Shore	0.323±0.08	0.187±0.06	0.136	1.32E-03	1.44E-09	-0.292
cg26651122	16	56641777	*MT2A*	TSS1500	N_Shore	0.385±0.044	0.249±0.062	0.135	4.22E-06	1.27E-10	-0.310
cg14834653	10	123353791	*FGFR2*	5'UTR/1st intron	N_Shelf	0.746±0.115	0.611±0.172	0.135	2.88E-02	7.32E-15	-0.371
cg27492942	17	36885965	*CISD3*	TSS1500	N_Shore	0.274±0.084	0.139±0.044	0.135	2.14E-03	4.03E-06	-0.225
cg12069042	3	48466700	*PLXNB1*	5'UTR	N_Shelf	0.556±0.089	0.421±0.127	0.135	5.88E-03	1.61E-24	-0.474
cg06099465	17	1631034	*WDR81*	1st exon	S_Shore	0.629±0.126	0.494±0.154	0.135	3.12E-02	8.46E-07	-0.240
cg07208344	7	150499362	*TMEM176B*	TSS1500	S_Shore	0.665±0.109	0.53±0.147	0.135	1.77E-02	2.77E-20	-0.433
cg20136951	1	2345400	*PEX10*	TSS1500	S_Shore	0.364±0.069	0.229±0.04	0.135	5.26E-04	1.12E-06	-0.237
cg14386312	19	58740861	*ZNF544*	5'UTR	S_Shore	0.432±0.071	0.297±0.095	0.135	8.21E-04	1.83E-17	-0.402
cg02864688	1	11752342	*MAD2L2*	TSS1500	Island	0.387±0.113	0.253±0.108	0.135	1.38E-02	2.14E-05	-0.208
cg15335139	3	50242325	*SLC38A3*	TSS1500	N_Shore	0.383±0.091	0.248±0.109	0.135	4.85E-03	3.39E-41	-0.597
cg09616053	16	2256512	*MLST8*	5'UTR	S_Shore	0.629±0.077	0.495±0.149	0.135	5.97E-03	9.75E-08	-0.259
cg11953516	2	27719501	*GCKR*	TSS1500	S_Shore	0.236±0.092	0.101±0.029	0.135	4.02E-03	2.46E-05	-0.206
cg16691944	3	44519652	*ZNF445*	TSS1500	S_Shore	0.496±0.119	0.361±0.157	0.135	2.71E-02	1.06E-10	-0.311
cg12599673	15	71408847	*CT62*	TSS1500	S_Shore	0.443±0.112	0.309±0.151	0.134	2.11E-02	5.71E-07	-0.243
cg11738921	7	1753396	*ELFN1*	5'UTR/1st intron	N_Shore	0.367±0.069	0.233±0.062	0.134	4.46E-04	1.13E-09	-0.294
cg17017284	1	161128717	*USP21*	TSS1500	N_Shore	0.439±0.113	0.304±0.154	0.134	2.30E-02	6.27E-09	-0.281
cg16300509	10	102280155	*SEC31B*	TSS1500	S_Shore	0.85±0.043	0.716±0.173	0.134	5.70E-03	3.41E-09	-0.286
cg20682326	7	100844173	*MOGAT3*	5'UTR/1st exon	N_Shore	0.312±0.094	0.178±0.059	0.134	4.23E-03	4.84E-10	-0.300
cg00618323	5	176515533	*FGFR4*	5'UTR/1st intron	S_Shore	0.318±0.082	0.184±0.043	0.134	1.88E-03	3.31E-13	-0.348
cg22580353	19	45146900	*PVR*	TSS200	N_Shore	0.331±0.066	0.197±0.078	0.134	3.90E-04	4.21E-05	-0.200
cg13779868	10	5488436	*NET1*	TSS200	N_Shore	0.234±0.11	0.1±0.043	0.134	1.03E-02	5.00E-07	-0.245
cg12079303	1	61547163	*NFIA*	TSS1500	N_Shore	0.21±0.084	0.077±0.031	0.133	2.51E-03	4.00E-12	-0.333
cg12718440	10	99393576	*MORN4*	5'UTR/1st intron	S_Shore	0.381±0.113	0.248±0.182	0.133	3.33E-02	2.19E-15	-0.377

cg03109701	12	117174758	RNFT2	TSS1500	N_Shore	0.419±0.099	0.286±0.09	0.133	6.53E-03	1.19E-05	-0.214
cg14038878	9	86431343	GKAP1	5'UTR	N_Shore	0.395±0.107	0.262±0.078	0.133	9.42E-03	1.72E-12	-0.338
cg00621899	7	100486423	UFSP1	1st exon/3'UTR	S_Shore	0.596±0.075	0.463±0.16	0.133	8.09E-03	8.74E-07	-0.239
cg00372249	4	185745711	ACSL1	5'UTR/1st intron	N_Shore	0.389±0.136	0.256±0.071	0.133	2.83E-02	3.20E-11	-0.319
cg03368690	16	27281055	NSMCE1	TSS1500	S_Shore	0.798±0.028	0.665±0.199	0.133	1.22E-02	6.60E-07	-0.242
cg06911974	5	172260464	ERGIC1	TSS1500	N_Shore	0.702±0.111	0.569±0.175	0.133	2.92E-02	1.23E-10	-0.310
cg14981637	1	95287127	SLC44A3	5'UTR/1st intron	S_Shore	0.458±0.113	0.325±0.146	0.133	2.19E-02	5.61E-13	-0.345
cg26763362	17	18266135	SHMT1	5'UTR/1st intron	N_Shore	0.256±0.076	0.123±0.027	0.133	1.41E-03	3.29E-05	-0.203
cg07845392	17	79678158	SLC25A10	TSS1500	S_Shelf	0.383±0.092	0.25±0.145	0.133	1.03E-02	4.92E-12	-0.332
cg27203641	17	40275359	HSPB9	1st exon/3'UTR	Island	0.573±0.136	0.44±0.103	0.133	3.18E-02	8.86E-20	-0.428
cg19313402	1	220102307	SLC30A10	TSS1500	S_Shore	0.385±0.091	0.252±0.086	0.133	4.05E-03	1.68E-20	-0.436
cg14791413	12	129338870	GLT1D1	5'UTR/1st intron	S_Shore	0.28±0.105	0.148±0.05	0.133	8.75E-03	1.31E-15	-0.380
cg00767496	15	45671279	GATM	5'UTR	S_Shore	0.327±0.099	0.195±0.162	0.133	1.84E-02	6.37E-06	-0.220
cg19747992	1	11713643	FBXO44	TSS1500	N_Shore	0.671±0.077	0.538±0.205	0.133	2.52E-02	5.32E-20	-0.430
cg14414415	6	3848634	FAM50B	TSS1500	N_Shore	0.621±0.112	0.488±0.134	0.132	1.85E-02	9.37E-07	-0.239
cg03605420	5	74162809	FAM169A	TSS1500	S_Shore	0.32±0.074	0.188±0.048	0.132	9.77E-04	1.35E-11	-0.325
cg08015496	1	160314061	COPA	TSS1500	S_Shore	0.415±0.126	0.283±0.144	0.132	3.21E-02	1.03E-06	-0.238
cg06204638	18	12656910	SPIRE1	TSS200	N_Shore	0.221±0.117	0.089±0.069	0.132	1.54E-02	2.44E-08	-0.270
cg03162994	18	23713729	PSMA8	TSS200	Island	0.613±0.129	0.481±0.165	0.132	4.18E-02	2.70E-05	-0.205
cg10158679	6	30883074	VARS2	5'UTR	S_Shore	0.626±0.088	0.494±0.127	0.132	6.56E-03	5.63E-06	-0.222
cg09826056	1	78511360	GIPC2	TSS1500	N_Shore	0.303±0.09	0.171±0.067	0.132	3.57E-03	5.13E-16	-0.385
cg19008097	5	140012986	CD14	5'UTR/1st exon	S_Shore	0.232±0.075	0.1±0.049	0.132	1.13E-03	1.38E-09	-0.293
cg05660005	1	145507663	RBM8A	5'UTR/1st exon	Island	0.298±0.086	0.166±0.084	0.132	3.06E-03	3.02E-10	-0.304
cg15698299	3	52233019	ALAS1	5'UTR	S_Shore	0.351±0.077	0.219±0.054	0.131	1.28E-03	2.64E-09	-0.288
cg03992976	22	36903496	FOXRED2	TSS1500	S_Shore	0.3±0.078	0.169±0.046	0.131	1.56E-03	1.21E-06	-0.236
cg25462903	11	47206835	PACSIN3	5'UTR/1st intron	N_Shore	0.508±0.135	0.377±0.125	0.131	3.67E-02	3.52E-25	-0.480
cg23603036	1	12675777	DHRS3	5'UTR/1st intron	N_Shore	0.266±0.086	0.135±0.037	0.131	3.13E-03	2.06E-09	-0.290
cg17359753	19	40790165	AKT2	5'UTR/1st intron	N_Shore	0.31±0.071	0.18±0.061	0.130	7.43E-04	6.07E-06	-0.221
cg00208734	13	36788843	SOHLH2	TSS200	Island	0.727±0.122	0.596±0.179	0.130	4.35E-02	4.08E-07	-0.246
cg15585294	11	22687641	GAS2	TSS1500	N_Shore	0.242±0.066	0.112±0.028	0.130	6.46E-04	3.18E-10	-0.303
cg14035045	3	50606289	C3orf18	5'UTR/1st intron	S_Shore	0.314±0.056	0.184±0.054	0.130	8.84E-05	1.09E-08	-0.277
cg14035045	3	50606289	HEMK1	TSS1500	S_Shore	0.314±0.056	0.184±0.054	0.130	8.84E-05	1.47E-07	-0.255
cg16958594	6	30882708	VARS2	5'UTR/1st intron	S_Shore	0.324±0.095	0.193±0.074	0.130	5.41E-03	4.32E-08	-0.266
cg18905161	6	84742998	MRAP2	TSS1500	N_Shore	0.289±0.079	0.158±0.046	0.130	1.72E-03	1.92E-05	-0.209
cg06055845	19	40788787	AKT2	5'UTR/1st intron	N_Shore	0.584±0.081	0.454±0.14	0.130	6.73E-03	4.21E-07	-0.246
cg22919728	3	126242490	CHST13	TSS1500	N_Shore	0.315±0.063	0.185±0.047	0.130	2.98E-04	2.61E-07	-0.250
cg25553916	5	177558755	RMND5B	5'UTR	S_Shore	0.454±0.098	0.324±0.093	0.130	7.29E-03	2.06E-11	-0.322
cg07147033	1	1549615	MIB2	TSS1500	N_Shore	0.339±0.109	0.209±0.096	0.130	1.33E-02	2.93E-07	-0.249
cg14528319	19	14607713	GIPC1	TSS1500	S_Shore	0.608±0.109	0.478±0.187	0.130	3.74E-02	2.59E-05	-0.206
cg17749374	4	2797201	SH3BP2	5'UTR/1st intron	S_Shelf	0.685±0.081	0.555±0.149	0.130	8.86E-03	2.38E-08	-0.271
cg21167159	11	2018154	H19	1st exon	Island	0.749±0.065	0.619±0.183	0.130	1.42E-02	2.11E-05	-0.208
cg13127231	13	111806949	ARHGEF7	5'UTR	S_Shore	0.279±0.134	0.15±0.09	0.130	3.14E-02	3.58E-05	-0.202
cg07343027	17	80024200	DUS1L	TSS1500	S_Shore	0.438±0.133	0.308±0.144	0.130	4.17E-02	2.85E-08	-0.269
cg18881641	6	170103498	WDR27	TSS1500	S_Shore	0.38±0.093	0.251±0.161	0.130	1.72E-02	4.75E-08	-0.265
cg00717678	17	1554577	RILP	TSS1500	S_Shore	0.632±0.091	0.502±0.124	0.130	7.82E-03	1.20E-06	-0.237
cg13663218	12	72666976	TRHDE	1st exon	Island	0.267±0.137	0.138±0.073	0.129	3.38E-02	3.67E-08	-0.267
cg08331960	16	2076597	SLC9A3R2	TSS1500	N_Shore	0.305±0.041	0.176±0.048	0.129	3.62E-06	1.15E-05	-0.214
cg12355677	6	168479787	FRMD1	5'UTR/1st exon	S_Shelf	0.719±0.06	0.59±0.194	0.129	1.77E-02	3.84E-05	-0.201
cg23964386	9	114362249	PTGR1	TSS1500	S_Shore	0.26±0.07	0.131±0.035	0.129	8.92E-04	3.08E-06	-0.228
cg09447578	7	21582444	DNAH11	TSS1500	Island	0.339±0.087	0.21±0.122	0.129	6.61E-03	2.26E-09	-0.289
cg01103827	21	40554221	PSMG1	5'UTR/1st intron	N_Shore	0.31±0.101	0.181±0.068	0.129	8.06E-03	7.76E-12	-0.329
cg06776976	1	212588580	TMEM206	TSS1500	S_Shore	0.27±0.129	0.141±0.101	0.128	3.00E-02	1.11E-05	-0.215
cg23396786	2	73299151	SFXN5	TSS1500	Island	0.22±0.074	0.092±0.033	0.128	1.37E-03	1.15E-08	-0.276
cg17696194	11	58941051	DTX4	5'UTR/1st intron	S_Shore	0.295±0.095	0.167±0.073	0.128	6.21E-03	2.06E-10	-0.307
cg03484180	3	49967517	MON1A	TSS200	S_Shore	0.175±0.066	0.047±0.023	0.128	7.87E-04	2.90E-05	-0.204
cg12600843	6	30179881	TRIM26	5'UTR/1st intron	N_Shore	0.322±0.096	0.194±0.046	0.128	6.47E-03	2.15E-15	-0.377
cg04622125	21	46876658	COL18A1	1st exon	Island	0.553±0.084	0.425±0.052	0.128	2.91E-03	4.76E-10	-0.301
cg24659758	10	71891123	AIFM2	5'UTR/1st intron	N_Shore	0.552±0.112	0.424±0.163	0.128	3.16E-02	1.70E-36	-0.568
cg03551406	16	56715756	MT1X	TSS1500	N_Shore	0.49±0.068	0.363±0.091	0.128	9.09E-04	1.14E-05	-0.214
cg00451635	16	10675030	EMP2	TSS1500	S_Shore	0.369±0.082	0.241±0.075	0.128	2.61E-03	1.98E-08	-0.272
cg01475325	11	76498701	TSKU	5'UTR/1st intron	S_Shelf	0.5±0.085	0.372±0.165	0.128	1.59E-02	4.83E-35	-0.558
cg01293143	20	62688326	TCEA2	5'UTR/1st exon	N_Shore	0.412±0.103	0.284±0.126	0.128	1.50E-02	2.30E-07	-0.252
cg27481594	15	43623282	ADAL	5'UTR/1st intron	S_Shore	0.258±0.105	0.13±0.051	0.128	1.07E-02	3.97E-13	-0.347
cg27481594	15	43623282	LCMT2	TSS1500	S_Shore	0.258±0.105	0.13±0.051	0.128	1.07E-02	2.92E-05	-0.204
cg01620164	2	164590272	FIGN	5'UTR/1st intron	N_Shelf	0.269±0.133	0.141±0.12	0.128	3.83E-02	1.71E-05	-0.210
cg20823695	1	2345410	PEX10	TSS1500	S_Shore	0.272±0.067	0.145±0.035	0.127	7.04E-04	2.97E-05	-0.204
cg12133118	17	19436770	SLC47A1	TSS1500	N_Shore	0.356±0.141	0.229±0.074	0.127	4.09E-02	1.10E-26	-0.494
cg01756799	17	40715281	COASY	1st exon	N_Shelf	0.351±0.083	0.225±0.058	0.127	2.92E-03	1.23E-07	-0.257
cg00262344	18	23713741	PSMA8	TSS200	Island	0.678±0.121	0.551±0.149	0.126	3.56E-02	4.81E-08	-0.265
cg16499834	12	131355834	RAN	TSS1500	N_Shore	0.408±0.108	0.282±0.11	0.126	1.66E-02	4.79E-06	-0.223
cg14091631	1	225117076	DNAH14	TSS1500	N_Shore	0.315±0.089	0.189±0.075	0.126	4.46E-03	5.99E-06	-0.221
cg03634777	6	160512657	LOC729603	TSS1500	S_Shore	0.765±0.035	0.639±0.149	0.126	2.76E-03	4.51E-07	-0.246
cg13120726	4	164394822	TKTL2	1st exon	Island	0.78±0.079	0.654±0.156	0.126	1.18E-02	2.39E-06	-0.230
cg03077712	17	7138739	DVL2	TSS1500	N_Shelf	0.659±0.057	0.533±0.182	0.126	1.43E-02	2.96E-10	-0.304
cg16345272	6	30875382	GTF2H4	TSS1500	N_Shore	0.49±0.093	0.364±0.059	0.126	5.82E-03	1.32E-23	-0.467
cg05118916	1	55505063	PCSK9	TSS1500	Island	0.246±0.079	0.12±0.041	0.126	2.34E-03	3.39E-05	-0.203
cg13357341	1	150849964	ARNT	TSS1500	S_Shore	0.404±0.098	0.278±0.127	0.126	1.39E-02	1.10E-11	-0.326
cg05370752	21	40756954	WRB	5'UTR/1st intron	N_Shore	0.666±0.089	0.54±0.105	0.126	6.36E-03	7.22E-11	-0.314
cg04140212	2	85829138	TMEM150A	5'UTR	N_Shore	0.258±0.083	0.133±0.044	0.125	3.01E-03	6.81E-07	-0.242
cg24012708	9	116138128	HDHD3	5'UTR/1st exon	N_Shore	0.535±0.067	0.409±0.037	0.125	8.00E-04	1.34E-18	-0.415
cg24121733	19	7953984	LRRC8E	5'UTR/1st intron	S_Shore	0.629±0.068	0.503±0.153	0.125	8.06E-03	5.28E-10	-0.300
cg25821245	3	46608056	LRRC2	TSS200	S_Shore	0.216±0.07	0.091±0.032	0.125	1.16E-03	5.08E-08	-0.264
cg02433278	17	37856230	ERBB2	1st exon	N_Shore	0.18±0.099	0.056±0.023	0.125	9.04E-03	1.49E-05	-0.212
cg01530032	17	19435805	SLC47A1	TSS1500	N_Shore	0.541±0.074	0.416±0.208	0.125	3.45E-02	7.23E-31	-0.527
cg23055772	7	94954438	PON1	TSS1500	S_Shore	0.578±0.095	0.454±0.19	0.125	3.59E-02	2.04E-12	-0.337
cg12638737	1	204120607	ETNK2	5'UTR/1st intron	Island	0.28±0.127	0.156±0.05	0.125	2.75E-02	9.24E-24	-0.468
cg26527984	17	73761562	GALK1	TSS1500	S_Shore	0.341±0.08	0.216±0.075	0.125	2.57E-03	4.96E-08	-0.265

cg23333072	17	37856228	*ERBB2*	5'UTR	N_Shore	0.205±0.091	0.081±0.041	0.125	5.55E-03	1.13E-06	-0.237
cg08878368	16	51183363	*SALL1*	5'UTR/1st intron	N_Shore	0.2±0.081	0.075±0.03	0.125	2.99E-03	1.13E-08	-0.277
cg06232807	16	51185132	*SALL1*	1st exon	Island	0.248±0.074	0.123±0.038	0.124	1.63E-03	2.35E-07	-0.251
cg00162862	16	15151269	*NTAN1*	TSS1500	S_Shore	0.723±0.095	0.599±0.156	0.124	2.06E-02	4.32E-16	-0.386
cg18176482	1	161128720	*USP21*	TSS1500	N_Shore	0.436±0.112	0.312±0.178	0.124	4.32E-02	7.24E-12	-0.329
cg04653308	9	131872018	*CRAT*	5'UTR/1st intron	N_Shore	0.334±0.071	0.21±0.036	0.124	1.26E-03	4.07E-07	-0.246
cg15198068	22	19843949	*GNB1L*	TSS1500	S_Shore	0.571±0.044	0.447±0.187	0.124	1.50E-02	2.89E-05	-0.204
cg17095929	11	64883911	*ZNHIT2*	1st exon/3'UTR	S_Shelf	0.529±0.108	0.405±0.124	0.124	2.09E-02	1.00E-08	-0.278
cg21231302	9	140116093	*RNF208*	TSS1500	S_Shore	0.481±0.12	0.357±0.115	0.124	2.89E-02	5.11E-09	-0.283
cg20229788	2	27718498	*GCKR*	TSS1500	S_Shore	0.216±0.071	0.093±0.036	0.124	1.25E-03	5.30E-10	-0.300
cg20229788	2	27718498	*FNDC4*	TSS1500	S_Shore	0.216±0.071	0.093±0.036	0.124	1.25E-03	3.60E-07	-0.248
cg00425827	5	131346715	*ACSL6*	5'UTR/1st intron	N_Shore	0.674±0.084	0.551±0.212	0.123	4.44E-02	2.89E-20	-0.433
cg01560900	1	241804128	*OPN3*	TSS1500	S_Shore	0.435±0.124	0.312±0.156	0.123	4.57E-02	1.40E-07	-0.256
cg07056626	19	51845446	*VSIG10L*	TSS200	S_Shelf	0.433±0.094	0.31±0.101	0.123	9.15E-03	5.29E-07	-0.244
cg01252455	5	175294907	*CPLX2*	5'UTR	N_Shelf	0.404±0.082	0.28±0.059	0.123	3.18E-03	3.30E-05	-0.203
cg13714540	6	137366432	*IL20RA*	TSS1500	S_Shore	0.825±0.075	0.702±0.219	0.123	4.56E-02	3.83E-07	-0.247
cg20271029	1	46859845	*FAAH*	TSS200	Island	0.203±0.073	0.081±0.036	0.122	1.62E-03	1.63E-07	-0.255
cg14465583	9	114362289	*PTGR1*	TSS1500	S_Shore	0.207±0.082	0.085±0.047	0.122	3.45E-03	8.05E-07	-0.240
cg22378853	20	36148154	*BLCAP*	5'UTR	N_Shelf	0.436±0.122	0.314±0.162	0.122	4.91E-02	1.27E-06	-0.236
cg11835347	1	113248232	*RHOC*	5'UTR/1st intron	N_Shore	0.264±0.116	0.142±0.073	0.122	2.20E-02	7.33E-12	-0.329
cg03214087	7	75624699	*TMEM120A*	TSS1500	S_Shore	0.563±0.062	0.441±0.12	0.122	2.38E-03	1.81E-15	-0.378
cg02825171	6	168720183	*DACT2*	1st exon	Island	0.273±0.082	0.151±0.077	0.122	3.50E-03	5.75E-08	-0.263
cg00586317	12	122149765	*TMEM120B*	TSS1500	N_Shore	0.201±0.084	0.079±0.034	0.122	4.04E-03	1.36E-05	-0.212
cg04794268	8	145690383	*CYHR1*	5'UTR	S_Shelf	0.352±0.125	0.23±0.078	0.122	3.07E-02	8.28E-09	-0.279
cg04794268	8	145690383	*KIFC2*	TSS1500	S_Shelf	0.352±0.125	0.23±0.078	0.122	3.07E-02	2.62E-07	-0.250
cg21230493	8	26722496	*ADRA1A*	1st exon	Island	0.241±0.088	0.12±0.076	0.121	5.51E-03	3.86E-13	-0.348
cg10242476	3	46619364	*TDGF1*	5'UTR/1st exon	S_Shore	0.754±0.077	0.633±0.079	0.121	2.48E-03	5.49E-07	-0.244
cg07998951	7	152456579	*ACTR3B*	TSS1500	N_Shore	0.289±0.075	0.168±0.11	0.121	3.98E-03	1.05E-07	-0.258
cg26767614	19	13136168	*NFIX*	1st exon	Island	0.86±0.03	0.739±0.201	0.121	2.20E-02	6.61E-24	-0.469
cg08314408	16	30457493	*SEPHS2*	TSS200	S_Shore	0.198±0.067	0.078±0.025	0.121	1.19E-03	3.84E-08	-0.267
cg19494100	6	18387313	*RNF144B*	TSS1500	N_Shore	0.416±0.083	0.295±0.1	0.121	5.38E-03	8.23E-09	-0.279
cg09772831	19	12175160	*ZNF844*	TSS1500	N_Shore	0.43±0.071	0.309±0.205	0.121	3.65E-02	8.51E-13	-0.343
cg26383537	19	41817029	*CCDC97*	5'UTR/1st intron	S_Shore	0.63±0.089	0.509±0.152	0.121	1.93E-02	1.11E-07	-0.258
cg23036171	1	54359138	*DIO1*	TSS1500	S_Shelf	0.763±0.073	0.642±0.148	0.120	1.05E-02	2.73E-31	-0.531
cg27065979	13	52734134	*NEK3*	TSS1500	S_Shore	0.782±0.106	0.661±0.181	0.120	1.92E-02	1.96E-11	-0.323
cg15796819	17	19651190	*ALDH3A1*	5'UTR/1st intron	S_Shelf	0.348±0.079	0.228±0.097	0.120	4.09E-03	5.31E-11	-0.316
cg09944616	17	80275880	*CD7*	TSS1500	N_Shelf	0.695±0.073	0.575±0.171	0.120	1.91E-02	1.08E-05	-0.215
cg01136471	16	16313867	*ABCC6*	5'UTR	N_Shelf	0.735±0.058	0.615±0.101	0.120	9.09E-04	6.38E-09	-0.281
cg20774846	8	105479420	*DPYS*	TSS200	S_Shore	0.248±0.084	0.128±0.036	0.120	4.44E-03	2.23E-07	-0.252
cg17228942	11	7041930	*ZNF214*	TSS1500	Island	0.204±0.112	0.084±0.029	0.120	1.89E-02	8.70E-10	-0.296
cg17228942	11	7041930	*NLRP14*	5'UTR/1st exon	Island	0.204±0.112	0.084±0.029	0.120	1.89E-02	4.11E-07	-0.246
cg08248285	14	35182889	*CFL2*	5'UTR/1st exon	N_Shore	0.221±0.085	0.102±0.048	0.120	4.61E-03	1.45E-13	-0.353
cg06646021	1	229406520	*RAB4A*	TSS1500	N_Shore	0.331±0.067	0.212±0.06	0.120	9.37E-04	1.93E-12	-0.338
cg03964111	7	150498493	*TMEM176A*	5'UTR/1st intron	S_Shore	0.27±0.072	0.15±0.04	0.120	1.66E-03	3.54E-22	-0.452
cg03964111	7	150498493	*TMEM176B*	TSS1500	S_Shore	0.27±0.072	0.15±0.04	0.120	1.66E-03	1.58E-21	-0.446
cg06754987	1	44444250	*B4GALT2*	TSS1500	N_Shore	0.625±0.088	0.505±0.118	0.120	1.02E-02	8.47E-10	-0.296
cg00586623	16	11037122	*CLEC16A*	TSS1500	S_Shore	0.81±0.075	0.69±0.179	0.120	2.48E-02	1.06E-05	-0.215
cg02250594	18	55103353	*ONECUT2*	1st exon	Island	0.192±0.08	0.073±0.024	0.119	3.66E-03	3.98E-07	-0.247
cg03743982	16	75150833	*LDHD*	TSS200	S_Shore	0.239±0.072	0.12±0.034	0.119	1.81E-03	3.48E-13	-0.348
cg22523743	4	53115	*ZNF595*	TSS200	N_Shore	0.423±0.063	0.303±0.058	0.119	5.70E-04	5.13E-10	-0.300
cg04176995	6	30586468	*PPP1R10*	TSS1500	S_Shore	0.22±0.085	0.101±0.032	0.119	4.87E-03	7.34E-07	-0.241
cg00537979	21	46876731	*COL18A1*	1st exon	Island	0.634±0.087	0.515±0.059	0.119	5.50E-03	1.41E-06	-0.235
cg10919177	3	44519663	*ZNF445*	TSS1500	S_Shore	0.428±0.095	0.309±0.123	0.119	1.56E-02	2.15E-08	-0.271
cg11649376	12	81473234	*ACSS3*	5'UTR/1st intron	S_Shore	0.34±0.052	0.221±0.125	0.119	2.21E-03	9.46E-34	-0.549
cg11713493	17	17110295	*PLD6*	TSS1500	S_Shore	0.359±0.088	0.24±0.047	0.119	6.20E-03	8.96E-06	-0.217
cg14749417	12	6419509	*PLEKHG6*	TSS1500	N_Shore	0.437±0.055	0.319±0.059	0.118	1.99E-04	3.37E-05	-0.203
cg11553245	8	17355443	*SLC7A2*	5'UTR/1st intron	S_Shore	0.238±0.038	0.12±0.026	0.118	1.08E-05	4.19E-06	-0.224
cg06418113	18	43915901	*RNF165*	5'UTR/1st intron	S_Shore	0.414±0.082	0.296±0.2	0.118	4.35E-02	6.41E-16	-0.384
cg19386379	11	844284	*TSPAN4*	5'UTR/1st intron	S_Shore	0.25±0.076	0.132±0.092	0.118	3.51E-03	7.90E-09	-0.279
cg06346993	6	44093974	*MRPL14*	5'UTR/1st exon	N_Shore	0.31±0.071	0.192±0.069	0.118	1.69E-03	1.82E-07	-0.254
cg06346993	6	44093974	*TMEM63B*	TSS1500	N_Shore	0.31±0.071	0.192±0.069	0.118	1.69E-03	1.12E-05	-0.215
cg08390696	13	99405102	*SLC15A1*	TSS200	Island	0.332±0.076	0.215±0.051	0.118	2.45E-03	9.06E-07	-0.239
cg20253700	16	2099508	*TSC2*	5'UTR	S_Shore	0.689±0.07	0.571±0.151	0.118	1.19E-02	7.80E-06	-0.218
cg05901765	5	68710818	*MARVELD2*	TSS200	Island	0.221±0.07	0.104±0.043	0.117	1.50E-03	3.63E-05	-0.202
cg06314202	16	1128070	*SSTR5*	5'UTR/1st intron	N_Shore	0.613±0.067	0.495±0.138	0.117	7.50E-03	6.20E-08	-0.263
cg04552852	11	844390	*TSPAN4*	5'UTR/1st intron	S_Shore	0.336±0.1	0.219±0.129	0.117	2.22E-02	9.79E-15	-0.369
cg25715816	2	70529513	*FAM136A*	TSS1500	S_Shore	0.61±0.088	0.492±0.166	0.117	2.83E-02	3.66E-07	-0.247
cg06707765	8	11604273	*GATA4*	5'UTR	N_Shelf	0.563±0.097	0.446±0.108	0.117	1.50E-02	1.33E-05	-0.213
cg16175725	12	121416796	*HNF1A*	1st exon	Island	0.542±0.093	0.425±0.056	0.117	8.77E-03	4.14E-25	-0.480
cg14576824	1	213224402	*RPS6KC1*	TSS200	N_Shore	0.229±0.124	0.112±0.092	0.117	3.60E-02	4.91E-07	-0.245
cg04410756	1	78956268	*PTGFR*	TSS1500	N_Shore	0.365±0.075	0.248±0.124	0.117	7.49E-03	5.04E-06	-0.223
cg02275418	3	15372726	*SH3BP5*	5'UTR/1st intron	N_Shore	0.524±0.11	0.407±0.121	0.117	2.83E-02	2.47E-09	-0.288
cg04207166	3	10546830	*ATP2B2*	5'UTR	N_Shore	0.296±0.063	0.179±0.046	0.117	7.26E-04	3.97E-12	-0.333
cg22014398	5	179230866	*MGAT4B*	TSS1500	N_Shore	0.314±0.088	0.197±0.051	0.117	6.37E-03	5.93E-24	-0.469
cg23731030	17	37856217	*ERBB2*	5'UTR	N_Shore	0.177±0.087	0.06±0.029	0.117	6.58E-03	6.11E-07	-0.243
cg02219360	11	2721409	*KCNQ1OT1*	TSS200	Island	0.459±0.037	0.342±0.128	0.117	1.79E-03	8.63E-08	-0.260
cg16454130	7	73245439	*CLDN4*	5'UTR/1st exon	Island	0.431±0.082	0.314±0.092	0.117	5.81E-03	1.23E-13	-0.354
cg12582959	19	56159199	*CCDC106*	5'UTR/1st exon	S_Shelf	0.401±0.129	0.285±0.09	0.117	4.28E-02	2.69E-24	-0.472
cg17971649	20	36153225	*BLCAP*	5'UTR/1st intron	S_Shore	0.745±0.027	0.629±0.186	0.117	1.80E-02	1.14E-10	-0.311
cg26860257	19	42755793	*ERF*	5'UTR/1st intron	N_Shore	0.682±0.059	0.566±0.127	0.116	4.11E-03	3.62E-05	-0.202
cg26034769	18	55714670	*NEDD4L*	5'UTR/1st exon	S_Shelf	0.482±0.116	0.366±0.131	0.116	3.87E-02	2.29E-11	-0.322
cg26477576	10	89419292	*PAPSS2*	TSS200	N_Shore	0.267±0.105	0.151±0.094	0.116	1.99E-02	7.00E-14	-0.358
cg06191390	2	73299285	*SFXN5*	TSS1500	S_Shore	0.207±0.071	0.092±0.034	0.116	2.05E-03	7.91E-13	-0.343
cg16488174	1	16971585	*MST1P2*	TSS1500	N_Shore	0.369±0.069	0.253±0.046	0.116	1.45E-03	6.28E-06	-0.220
cg09305680	8	117778069	*UTP23*	TSS1500	N_Shore	0.27±0.104	0.155±0.096	0.116	1.97E-02	9.49E-11	-0.312
cg22883889	20	61583686	*SLC17A9*	TSS1500	N_Shore	0.277±0.079	0.162±0.05	0.116	3.60E-03	8.70E-10	-0.296
cg07224067	17	48555565	*RSAD1*	TSS1500	N_Shore	0.545±0.077	0.43±0.11	0.115	6.23E-03	9.63E-08	-0.259

cg18278486	6	26987575	*OC10027074*	1st exon	N_Shore	0.255±0.086	0.139±0.038	0.115	6.16E-03	1.40E-11	-0.325
cg14391622	3	124931956	*SLC12A8*	TSS1500	S_Shore	0.315±0.094	0.2±0.1	0.115	1.32E-02	3.23E-09	-0.286
cg05337019	1	16062361	*SLC25A34*	TSS1500	S_Shelf	0.366±0.081	0.251±0.09	0.115	5.54E-03	3.02E-13	-0.349
cg06230674	18	20716991	*CABLES1*	5'UTR/1st intron	Island	0.315±0.059	0.2±0.083	0.115	7.31E-04	6.26E-17	-0.396
cg22975712	22	38218945	*GALR3*	TSS1500	N_Shore	0.674±0.079	0.559±0.18	0.115	3.33E-02	5.71E-06	-0.221
cg26277102	3	75834282	*ZNF717*	5'UTR/1st intron	Island	0.365±0.076	0.25±0.083	0.115	3.63E-03	2.73E-09	-0.288
cg24952744	3	10547584	*ATP2B2*	5'UTR	S_Shore	0.28±0.071	0.166±0.062	0.115	1.93E-03	6.33E-14	-0.358
cg25970491	22	19279922	*CLTCL1*	TSS1500	S_Shore	0.291±0.08	0.177±0.042	0.115	4.24E-03	3.45E-05	-0.203
cg16301036	12	118808298	*TAOK3*	5'UTR/1st intron	N_Shore	0.703±0.047	0.588±0.18	0.115	2.01E-02	2.28E-09	-0.289
cg25213362	12	51236634	*TMPRSS12*	TSS200	Island	0.656±0.097	0.542±0.124	0.115	2.11E-02	2.68E-06	-0.229
cg11851129	12	57624530	*SHMT2*	5'UTR/1st intron	S_Shore	0.349±0.074	0.234±0.05	0.114	2.54E-03	2.77E-06	-0.229
cg10372473	19	10735594	*SLC44A2*	TSS1500	N_Shore	0.467±0.057	0.352±0.138	0.114	6.49E-03	4.88E-06	-0.223
cg27549208	7	43965451	*UBE2D4*	TSS1500	N_Shore	0.249±0.075	0.135±0.043	0.114	2.79E-03	4.30E-06	-0.224
cg08687251	14	74003780	*HEATR4*	5'UTR/1st intron	N_Shore	0.369±0.071	0.254±0.042	0.114	1.99E-03	4.44E-16	-0.386
cg08687251	14	74003780	*ACOT1*	TSS200	N_Shore	0.369±0.071	0.254±0.042	0.114	1.99E-03	1.70E-13	-0.352
cg20165416	10	89264080	*MINPP1*	TSS200	N_Shore	0.182±0.067	0.068±0.025	0.114	1.70E-03	2.46E-07	-0.251
cg02711479	1	36849024	*STK40*	5'UTR/1st intron	N_Shelf	0.871±0.038	0.757±0.179	0.114	1.77E-02	7.25E-15	-0.371
cg16642695	8	67343633	*ADHFE1*	TSS1500	N_Shore	0.321±0.119	0.207±0.058	0.114	3.12E-02	3.18E-13	-0.349
cg02998861	19	1491701	*PCSK4*	TSS1500	Island	0.271±0.078	0.157±0.043	0.114	3.65E-03	2.61E-07	-0.250
cg10974128	10	93393509	*PPP1R3C*	TSS1500	S_Shore	0.284±0.073	0.17±0.072	0.114	2.67E-03	1.18E-05	-0.214
cg18607308	16	66969500	*CES2*	5'UTR/1st exon	Island	0.337±0.057	0.223±0.066	0.113	4.23E-04	2.67E-16	-0.389
cg24107665	8	144372932	*ZNF696*	TSS1500	S_Shore	0.183±0.087	0.069±0.03	0.113	7.36E-03	1.18E-08	-0.276
cg02712878	3	138327370	*FAIM*	TSS1500	N_Shore	0.35±0.1	0.236±0.057	0.113	1.49E-02	1.46E-05	-0.212
cg20277416	11	64879061	*TM7SF2*	TSS1500	Island	0.644±0.097	0.53±0.133	0.113	2.49E-02	2.13E-10	-0.306
cg18608017	10	73158072	*CDH23*	5'UTR/1st intron	S_Shore	0.268±0.058	0.155±0.038	0.113	5.15E-04	2.21E-06	-0.231
cg05453016	16	15073109	*PDXDC1*	5'UTR/1st intron	S_Shelf	0.391±0.075	0.278±0.067	0.113	3.27E-03	1.08E-10	-0.311
cg01925521	4	166299123	*CPE*	TSS1500	N_Shore	0.752±0.069	0.639±0.205	0.113	4.80E-02	1.54E-05	-0.211
cg27295373	1	220102277	*SLC30A10*	TSS1500	S_Shore	0.363±0.105	0.25±0.088	0.113	2.13E-02	2.62E-18	-0.412
cg01194538	22	43043270	*CYB5R3*	TSS1500	N_Shore	0.414±0.111	0.301±0.13	0.113	3.76E-02	9.38E-08	-0.259
cg24996280	8	143481281	*TSNARE1*	5'UTR/1st intron	N_Shelf	0.542±0.079	0.429±0.18	0.113	3.56E-02	4.12E-20	-0.431
cg14326805	9	71737756	*TJP2*	5'UTR/1st intron	S_Shore	0.226±0.077	0.113±0.04	0.113	3.70E-03	6.33E-07	-0.242
cg12013321	6	53410182	*GCLC*	TSS1500	S_Shore	0.193±0.079	0.081±0.03	0.113	4.64E-03	2.16E-06	-0.231
cg09284708	6	39901897	*MOCS1*	5'UTR/1st exon	Island	0.187±0.091	0.074±0.04	0.113	9.36E-03	7.10E-10	-0.298
cg01322405	21	40757317	*WRB*	5'UTR/1st intron	N_Shore	0.698±0.082	0.586±0.091	0.113	7.29E-03	6.87E-14	-0.358
cg05399330	1	55464530	*BSND*	TSS200	S_Shore	0.365±0.111	0.253±0.086	0.112	2.77E-02	5.64E-06	-0.222
cg10883038	1	115211984	*DENND2C*	5'UTR/1st intron	N_Shore	0.212±0.078	0.099±0.037	0.112	4.25E-03	4.73E-08	-0.265
cg01156834	17	36763024	*SRCIN1*	TSS1500	Island	0.235±0.074	0.122±0.052	0.112	2.77E-03	4.09E-10	-0.302
cg19491035	12	12502846	*MANSC1*	5'UTR/1st intron	N_Shore	0.27±0.109	0.158±0.127	0.112	3.54E-02	3.27E-09	-0.286
cg25008263	14	89884608	*FOXN3*	5'UTR/1st intron	S_Shore	0.255±0.077	0.143±0.051	0.112	3.82E-03	8.31E-10	-0.297
cg17303833	10	97889448	*ZNF518A*	TSS1500	N_Shore	0.345±0.086	0.233±0.145	0.112	2.22E-02	1.99E-07	-0.253
cg26220336	20	37554591	*FAM83D*	TSS1500	N_Shore	0.374±0.077	0.261±0.095	0.112	5.63E-03	1.64E-06	-0.234
cg16575104	5	10308246	*CMBL*	TSS200	S_Shore	0.197±0.098	0.084±0.032	0.112	1.42E-02	5.17E-28	-0.505
cg05483406	8	146013229	*ZNF34*	TSS1500	N_Shelf	0.377±0.087	0.265±0.117	0.112	1.43E-02	2.68E-13	-0.350
cg10061906	8	146013430	*ZNF34*	TSS1500	N_Shelf	0.472±0.063	0.36±0.176	0.112	2.59E-02	4.07E-06	-0.225
cg20234205	11	64492155	*NRXN2*	TSS1500	S_Shore	0.846±0.028	0.734±0.19	0.112	2.49E-02	7.16E-09	-0.280
cg02853107	4	41984968	*DCAF4L1*	1st exon	S_Shore	0.855±0.069	0.743±0.077	0.112	2.14E-03	7.86E-10	-0.297
cg22979368	2	217501581	*IGFBP2*	5'UTR/1st intron	S_Shelf	0.343±0.057	0.231±0.074	0.112	5.41E-04	1.75E-09	-0.291
cg03163767	16	66969506	*CES2*	5'UTR/1st exon	Island	0.386±0.059	0.274±0.078	0.112	7.91E-04	3.04E-14	-0.363
cg27119919	11	60684332	*TMEM109*	5'UTR/1st intron	S_Shelf	0.641±0.054	0.529±0.145	0.112	8.79E-03	2.61E-09	-0.288
cg13078931	1	53397458	*SCP2*	5'UTR/1st intron	S_Shelf	0.797±0.061	0.685±0.158	0.112	1.59E-02	3.34E-09	-0.286
cg13072940	3	49967521	*MON1A*	TSS200	S_Shore	0.21±0.055	0.099±0.025	0.112	4.93E-04	2.25E-06	-0.231
cg02409177	8	26721870	*ADRA1A*	1st exon	Island	0.294±0.107	0.182±0.081	0.112	2.35E-02	3.56E-16	-0.387
cg20396069	17	46908122	*CALCOCO2*	TSS1500	N_Shore	0.518±0.102	0.406±0.144	0.112	3.62E-02	7.13E-09	-0.280
cg06565604	17	78964771	*CHMP6*	TSS1500	N_Shore	0.43±0.095	0.318±0.091	0.111	1.54E-02	4.66E-06	-0.223
cg16910047	4	39368611	*RFC1*	TSS1500	S_Shore	0.614±0.052	0.503±0.048	0.111	2.20E-04	2.68E-14	-0.363
cg11329129	11	705692	*EPS8L2*	TSS1500	N_Shore	0.382±0.088	0.271±0.048	0.111	8.38E-03	2.70E-07	-0.250
cg06197377	1	55505061	*PCSK9*	TSS1500	Island	0.22±0.078	0.109±0.039	0.111	4.53E-03	5.34E-06	-0.222
cg05278955	5	76381538	*ZBED3*	5'UTR/1st intron	N_Shore	0.77±0.081	0.659±0.167	0.111	3.17E-02	2.32E-16	-0.389
cg02420927	3	12703582	*RAF1*	5'UTR/1st intron	N_Shore	0.315±0.05	0.204±0.084	0.111	4.04E-04	4.94E-07	-0.245
cg19631779	15	63339148	*TPM1*	TSS1500	N_Shore	0.766±0.054	0.655±0.126	0.111	4.56E-03	2.65E-16	-0.389
cg24315973	4	106631934	*GSTCD*	5'UTR/1st intron	S_Shore	0.812±0.029	0.702±0.186	0.110	2.45E-02	7.48E-11	-0.314
cg10760299	15	45669010	*GATM*	5'UTR	N_Shore	0.254±0.103	0.144±0.143	0.110	3.89E-02	7.55E-15	-0.370
cg13947469	7	63505871	*ZNF727*	5'UTR/1st exon	N_Shore	0.23±0.086	0.12±0.064	0.110	7.99E-03	1.80E-05	-0.210
cg22499139	6	29759718	*HCG4*	1st exon	N_Shore	0.592±0.104	0.482±0.132	0.110	3.59E-02	1.66E-11	-0.324
cg27618939	8	71581788	*XKR9*	5'UTR/1st intron	S_Shore	0.292±0.09	0.182±0.058	0.110	1.01E-02	5.28E-16	-0.385
cg27618939	8	71581788	*LACTB2*	TSS1500	S_Shore	0.292±0.09	0.182±0.058	0.110	1.01E-02	1.44E-05	-0.212
cg07189401	11	19736333	*OC10012678*	TSS200	S_Shore	0.232±0.1	0.122±0.101	0.109	2.27E-02	4.89E-09	-0.283
cg18535283	22	43044071	*CYB5R3*	TSS1500	N_Shore	0.612±0.094	0.503±0.123	0.109	2.41E-02	1.02E-05	-0.215
cg16562724	16	68270631	*ESRP2*	TSS1500	Island	0.281±0.075	0.172±0.024	0.109	4.26E-03	2.73E-05	-0.205
cg09706243	11	67121634	*POLD4*	TSS1500	S_Shore	0.215±0.031	0.106±0.034	0.109	1.00E-06	1.40E-05	-0.212
cg14268714	9	5186162	*INSL6*	TSS1500	S_Shore	0.781±0.066	0.672±0.141	0.109	1.30E-02	2.31E-16	-0.389
cg19766489	1	78511344	*GIPC2*	TSS1500	N_Shore	0.303±0.102	0.194±0.101	0.109	2.49E-02	5.03E-24	-0.470
cg13460740	22	21369777	*P2RX6*	5'UTR/1st intron	S_Shore	0.718±0.07	0.61±0.164	0.109	2.63E-02	4.66E-07	-0.245
cg10580335	3	125803859	*SLC41A3*	5'UTR/1st intron	S_Shore	0.843±0.047	0.734±0.182	0.108	2.76E-02	1.63E-09	-0.292
cg19362196	1	26233615	*STMN1*	TSS1500	Island	0.444±0.109	0.335±0.144	0.108	4.98E-02	1.91E-05	-0.209
cg04439776	10	71907412	*TYSND1*	TSS1500	S_Shore	0.499±0.062	0.391±0.155	0.108	1.78E-02	5.00E-12	-0.332
cg25569514	22	46549404	*PPARA*	5'UTR	S_Shore	0.872±0.015	0.763±0.177	0.108	1.92E-02	7.86E-08	-0.261
cg24854914	7	89784790	*STEAP1*	5'UTR/1st intron	S_Shore	0.699±0.108	0.591±0.142	0.108	4.70E-02	1.60E-34	-0.554
cg04037228	3	45636386	*LIMD1*	1st exon	S_Shore	0.304±0.078	0.196±0.04	0.108	5.31E-03	5.37E-11	-0.316
cg12074985	10	99080756	*FRAT1*	1st exon/3'UTR	Island	0.219±0.062	0.111±0.025	0.108	1.43E-03	4.21E-06	-0.224
cg20728156	2	217499344	*IGFBP2*	5'UTR/1st intron	S_Shore	0.25±0.08	0.142±0.047	0.108	5.75E-03	1.19E-09	-0.294
cg10376731	10	103113307	*BTRC*	TSS1500	N_Shore	0.799±0.061	0.691±0.167	0.108	2.40E-02	1.41E-05	-0.212
cg02835421	1	44411569	*IPO13*	TSS1500	N_Shore	0.251±0.1	0.143±0.047	0.108	1.88E-02	6.71E-10	-0.298
cg01444643	1	55504848	*PCSK9*	TSS1500	N_Shore	0.537±0.034	0.429±0.139	0.108	5.43E-03	1.39E-29	-0.517
cg04548483	8	145597148	*ADCK5*	TSS1500	N_Shore	0.221±0.094	0.113±0.048	0.108	1.38E-02	7.56E-08	-0.261
cg06649737	1	161068715	*KLHDC9*	1st exon	Island	0.245±0.074	0.137±0.07	0.108	4.24E-03	1.59E-15	-0.379
cg19695041	8	38615330	*TACC1*	5'UTR/1st intron	S_Shore	0.328±0.072	0.221±0.099	0.107	5.83E-03	2.16E-05	-0.208

cg01802294	4	17516550	*CLRN2*	TSS1500	S_Shelf	0.747±0.069	0.64±0.162	0.107	2.63E-02	1.85E-13	-0.352
cg18168672	20	61905223	*ARFGAP1*	5'UTR/1st intron	S_Shore	0.315±0.064	0.208±0.051	0.107	1.57E-03	4.34E-07	-0.246
cg26052586	18	20717411	*CABLES1*	5'UTR/1st intron	S_Shore	0.459±0.083	0.352±0.18	0.107	4.92E-02	3.22E-32	-0.537
cg14487271	8	87354422	*WWP1*	TSS1500	N_Shore	0.237±0.079	0.13±0.041	0.107	5.85E-03	2.40E-05	-0.206
cg13340636	3	147110667	*ZIC4*	TSS1500	Island	0.228±0.101	0.121±0.084	0.107	2.30E-02	6.03E-09	-0.282
cg05726600	7	150498594	*TMEM176A*	5'UTR/1st intron	S_Shore	0.192±0.058	0.086±0.031	0.107	9.06E-04	4.95E-15	-0.373
cg05726600	7	150498594	*TMEM176B*	TSS1500	S_Shore	0.192±0.058	0.086±0.031	0.107	9.06E-04	3.57E-14	-0.362
cg14220634	9	97020667	*ZNF169*	TSS1500	N_Shore	0.508±0.105	0.402±0.141	0.107	4.66E-02	1.29E-05	-0.213
cg07176589	8	77596382	*ZFHX4*	5'UTR/1st intron	S_Shore	0.252±0.088	0.146±0.094	0.107	1.40E-02	2.00E-19	-0.424
cg06418871	5	68710831	*MARVELD2*	TSS200	Island	0.199±0.078	0.092±0.049	0.106	5.80E-03	1.68E-05	-0.210
cg13555772	11	705748	*EPS8L2*	TSS1500	N_Shore	0.311±0.059	0.205±0.048	0.106	9.35E-04	2.71E-07	-0.250
cg15006681	22	38199759	*H1F0*	TSS1500	N_Shore	0.482±0.083	0.376±0.131	0.106	2.17E-02	6.68E-20	-0.429
cg24338748	17	34947916	*DHRS11*	TSS1500	N_Shore	0.199±0.076	0.093±0.04	0.106	4.87E-03	5.96E-07	-0.243
cg20785560	1	78511235	*GIPC2*	TSS1500	N_Shore	0.264±0.1	0.158±0.135	0.106	3.85E-02	1.43E-23	-0.466
cg00751332	18	20714332	*CABLES1*	TSS1500	N_Shore	0.279±0.092	0.174±0.054	0.106	1.38E-02	2.59E-05	-0.206
cg05093315	11	18127958	*SAAL1*	TSS1500	S_Shore	0.448±0.09	0.343±0.087	0.106	1.50E-02	3.98E-12	-0.333
cg00103329	1	204121923	*ETNK2*	TSS1500	S_Shore	0.71±0.061	0.604±0.137	0.105	1.18E-02	1.62E-23	-0.465
cg17935233	1	156338826	*RHBG*	TSS200	Island	0.273±0.052	0.168±0.046	0.105	3.32E-04	2.76E-07	-0.250
cg20687553	17	79876287	*SIRT7*	TSS1500	S_Shore	0.504±0.057	0.399±0.107	0.105	3.49E-03	6.38E-18	-0.408
cg03514351	1	65991765	*LEPR*	5'UTR	Island	0.178±0.09	0.073±0.033	0.105	1.24E-02	1.30E-06	-0.236
cg07644368	5	115152785	*CDO1*	TSS1500	S_Shore	0.188±0.074	0.083±0.031	0.105	4.61E-03	1.95E-22	-0.455
cg19607845	6	52934457	*FBXO9*	5'UTR/1st intron	S_Shelf	0.459±0.088	0.354±0.133	0.105	2.73E-02	2.86E-06	-0.228
cg07850418	3	147110378	*ZIC4*	TSS200	Island	0.236±0.093	0.131±0.094	0.105	1.91E-02	1.07E-07	-0.258
cg18727948	4	53233	*ZNF595*	5'UTR/1st exon	Island	0.626±0.054	0.521±0.133	0.105	8.71E-03	1.30E-45	-0.623
cg14338345	9	22447679	*DMRTA1*	1st exon	Island	0.251±0.092	0.147±0.044	0.105	1.47E-02	2.35E-11	-0.321
cg02097479	17	34891843	*MYO19*	TSS1500	S_Shore	0.301±0.079	0.196±0.145	0.105	2.64E-02	9.39E-08	-0.259
cg25492569	3	46608025	*LRRC2*	5'UTR/1st exon	S_Shore	0.211±0.056	0.106±0.036	0.104	7.40E-04	1.89E-05	-0.209
cg02900253	1	6673709	*PHF13*	TSS200	Island	0.324±0.074	0.22±0.079	0.104	5.66E-03	2.19E-05	-0.207
cg10124440	12	6960295	*CDCA3*	5'UTR/1st intron	N_Shore	0.386±0.064	0.282±0.08	0.104	2.59E-03	8.53E-14	-0.357
cg01419539	4	53010	*ZNF595*	TSS200	N_Shore	0.415±0.037	0.311±0.063	0.104	3.06E-05	1.08E-26	-0.494
cg19248564	22	43042879	*CYB5R3*	5'UTR/1st exon	N_Shelf	0.372±0.083	0.268±0.11	0.104	1.64E-02	6.55E-07	-0.242
cg12100463	14	105259826	*AKT1*	5'UTR/1st exon	N_Shore	0.782±0.067	0.678±0.134	0.104	1.47E-02	4.64E-10	-0.301
cg16759783	18	77746937	*TXNL4A*	5'UTR	N_Shore	0.347±0.096	0.243±0.091	0.104	2.28E-02	1.01E-08	-0.278
cg00423487	19	4455957	*UBXN6*	TSS1500	N_Shore	0.417±0.078	0.313±0.082	0.104	7.90E-03	1.36E-06	-0.235
cg01717201	17	48174262	*PDK2*	5'UTR	S_Shore	0.605±0.051	0.502±0.113	0.104	3.85E-03	1.98E-20	-0.435
cg12206103	15	90319516	*MESP2*	TSS200	N_Shore	0.252±0.062	0.149±0.046	0.103	1.62E-03	2.79E-09	-0.287
cg16981943	10	91458029	*FLJ37201*	TSS1500	N_Shelf	0.82±0.047	0.717±0.16	0.103	2.00E-02	1.41E-05	-0.212
cg00525011	16	122031	*RHBDF1*	5'UTR/1st intron	Island	0.259±0.059	0.156±0.042	0.103	1.15E-03	2.45E-05	-0.206
cg17125688	2	20646181	*RHOB*	TSS1500	N_Shore	0.156±0.101	0.053±0.032	0.103	2.34E-02	1.13E-17	-0.405
cg06553312	19	50706723	*MYH14*	TSS200	N_Shore	0.22±0.069	0.117±0.072	0.103	3.83E-03	1.09E-07	-0.258
cg13722127	7	150037890	*RARRES2*	5'UTR/1st intron	Island	0.29±0.049	0.187±0.04	0.103	2.73E-04	5.94E-08	-0.263
cg06010659	5	1634505	*LOC728613*	TSS1500	S_Shore	0.298±0.089	0.195±0.134	0.103	3.19E-02	1.97E-12	-0.337
cg01017689	5	76376266	*ZBED3*	5'UTR/1st intron	S_Shelf	0.805±0.047	0.702±0.152	0.103	1.61E-02	7.19E-10	-0.298
cg05451722	8	145065385	*GRINA*	5'UTR	Island	0.246±0.094	0.143±0.076	0.103	1.92E-02	9.91E-08	-0.259
cg21649442	16	82044960	*SDR42E1*	5'UTR/1st intron	Island	0.225±0.084	0.123±0.045	0.103	1.01E-02	6.85E-19	-0.418
cg16387467	18	72166016	*CNDP2*	5'UTR/1st intron	S_Shore	0.297±0.085	0.194±0.063	0.103	1.17E-02	2.71E-05	-0.205
cg03805684	11	61561219	*FEN1*	5'UTR/1st intron	S_Shore	0.356±0.076	0.253±0.108	0.102	1.27E-02	1.16E-13	-0.355
cg20322299	2	118849751	*INSIG2*	5'UTR	S_Shelf	0.808±0.023	0.706±0.164	0.102	1.83E-02	5.03E-24	-0.470
cg18153630	13	99405124	*SLC15A1*	TSS200	S_Shore	0.207±0.059	0.105±0.033	0.102	1.25E-03	6.48E-08	-0.262
cg18734428	15	84322277	*ADAMTSL3*	TSS1500	N_Shore	0.169±0.089	0.067±0.034	0.102	1.36E-02	2.80E-05	-0.205
cg26478401	10	90749920	*FAS*	TSS1500	N_Shore	0.236±0.073	0.134±0.084	0.102	6.63E-03	3.66E-12	-0.334
cg14874337	19	38864187	*PSMD8*	TSS1500	N_Shore	0.757±0.093	0.655±0.148	0.102	4.52E-02	1.02E-07	-0.259
cg26921612	22	32150828	*DEPDC5*	5'UTR/1st intron	S_Shore	0.193±0.055	0.091±0.022	0.102	9.72E-04	4.27E-09	-0.284
cg03802191	1	234615169	*TARBP1*	TSS1500	S_Shore	0.352±0.084	0.251±0.118	0.102	2.20E-02	8.30E-11	-0.313
cg00747849	22	19165386	*SLC25A1*	5'UTR	Island	0.239±0.062	0.137±0.045	0.102	1.83E-03	9.36E-10	-0.296
cg23712458	17	189002	*RPH3AL*	5'UTR/1st intron	S_Shelf	0.444±0.056	0.343±0.102	0.101	3.49E-03	4.02E-07	-0.247
cg05029558	8	71581784	*XKR9*	5'UTR/1st intron	S_Shore	0.288±0.083	0.186±0.06	0.101	1.03E-02	4.11E-12	-0.333
cg04532834	19	4535188	*PLIN5*	5'UTR/1st exon	Island	0.316±0.066	0.215±0.041	0.101	2.76E-03	7.54E-10	-0.297
cg20303399	8	26722965	*ADRA1A*	1st exon	Island	0.232±0.056	0.131±0.053	0.101	8.36E-04	2.24E-08	-0.271
cg11136751	3	46608069	*LRRC2*	TSS200	S_Shore	0.171±0.057	0.07±0.023	0.101	1.32E-03	6.59E-07	-0.242
cg27021512	13	47371635	*ESD*	TSS1500	S_Shore	0.204±0.06	0.103±0.031	0.100	1.65E-03	1.59E-08	-0.274
cg00860380	1	54871988	*SSBP3*	5'UTR/1st exon	N_Shore	0.403±0.047	0.303±0.086	0.100	8.66E-04	7.51E-06	-0.219
cg14993491	1	55504971	*PCSK9*	TSS1500	N_Shore	0.218±0.072	0.118±0.04	0.100	4.84E-03	2.56E-06	-0.229
cg07181881	2	170591239	*KLHL23*	5'UTR/1st intron	S_Shore	0.172±0.073	0.072±0.036	0.100	5.55E-03	1.30E-05	-0.213
cg21235334	20	31351171	*DNMT3B*	5'UTR/1st intron	Island	0.197±0.057	0.097±0.032	0.100	1.26E-03	1.21E-05	-0.214

[a]Probe IDs for the Infinium MethylationEPIC BeadChip (Illumina). [b]National Center for Biotechnology Information (NCBI) Database (Genome Build 37/hg19). [c]TSS1500, from 200 bp upstream of the transcription start site (TSS) to 1500 bp upstream of it; TSS200, from the TSS to 200 bp upstream of it; UTR, untranslated region; Gene body, second exon and downstream on the RefSeq database (http://www.ncbi.nlm.nih.gov/refseq/). [d]N-Shelf, 2000 bp region 5' adjacent to N-Shore; N-Shore, 2000 bp region 5' adjacent to CpG island; S-Shore, 2000 bp region 3' adjacent to CpG island; S-Shelf, 2000 bp region 3' adjacent to SShore based on the UCSC Genome Browser (https://genome.ucsc.edu/). [e]Evaluated using the Cancer Genome Atlas database (https://www.cancer.gov/about-nci/organization/ccg/research/structural-genomics/tcga).

b The 2,093 probes showing DNA hypomethylation in Cluster I in comparison with Cluster II ($\Delta\beta_{I-II}$<-0.1).

ProbeID[a]	Chromosome	Position[b]	Gene symbol	Annotation[c]	CpG type[d]	DNA methylatio levels (β value)					
						Cluster I (mean±SD)	Cluster II (mean±SD)	$\Delta\beta_{I-II}$	Welch's T-test	Inverse correlation with mRNA expression levels[e]	
									P	*P*	r
cg07759570	19	35491951	*GRAMD1A*	5'UTR/1st intron	Island	0.148±0.118	0.683±0.245	-0.535	9.94E-08	3.63E-25	-0.480
cg18056600	17	4642047	*ZMYND15*	TSS1500	TSS1500	0.23±0.113	0.723±0.075	-0.493	5.65E-07	1.66E-16	-0.391
cg15346168	14	24836194	*NFATC4*	5'UTR/1st exon	N_Shore	0.263±0.208	0.752±0.194	-0.489	8.57E-05	8.72E-09	-0.279
cg02716776	19	3178413	*S1PR4*	TSS1500	N_Shore	0.322±0.193	0.788±0.11	-0.466	1.20E-04	2.77E-18	-0.412
cg19841506	17	4642056	*ZMYND15*	TSS1500	Island	0.213±0.091	0.679±0.108	-0.466	4.48E-09	4.12E-17	-0.398
cg01434608	16	66637927	*CMTM3*	5'UTR/1st exon	N_Shore	0.198±0.15	0.655±0.24	-0.457	7.90E-06	8.47E-12	-0.328
cg20780953	19	11450511	*RAB3D*	TSS200	S_Shore	0.237±0.117	0.686±0.077	-0.448	2.05E-06	3.22E-36	-0.566
cg09866102	16	31214134	*PYCARD*	TSS200	Island	0.191±0.23	0.637±0.247	-0.446	5.02E-04	7.00E-09	-0.280
cg22417733	6	153303409	*FBXO5*	5'UTR/1st intron	Island	0.204±0.145	0.646±0.244	-0.442	9.82E-06	5.48E-15	-0.372
cg00651829	22	23413066	*GNAZ*	5'UTR/1st intron	Island	0.085±0.119	0.523±0.261	-0.438	4.77E-06	3.04E-10	-0.304
cg06154084	10	102792249	*PDZD7*	TSS1500	S_Shelf	0.213±0.191	0.64±0.253	-0.427	1.72E-04	5.94E-13	-0.345

cg21207028	20	327619	*NRSN2*	1st exon	Island	0.284±0.189	0.705±0.174	-0.421	1.44E-04	1.20E-45	-0.623
cg17225129	6	33244755	*B3GALT4*	TSS200	Island	0.378±0.231	0.794±0.135	-0.416	9.82E-04	3.11E-48	-0.637
cg05896042	19	11450090	*RAB3D*	5'UTR/1st intron	Island	0.346±0.187	0.76±0.067	-0.414	3.27E-04	2.24E-48	-0.638
cg26381352	6	33244799	*B3GALT4*	TSS200	Island	0.342±0.196	0.753±0.117	-0.411	3.33E-04	1.63E-48	-0.638
cg09816471	16	11761860	*SNN*	TSS1500	N_Shore	0.307±0.129	0.712±0.094	-0.405	9.02E-06	1.69E-08	-0.273
cg05015913	8	38645613	*TACC1*	5'UTR	Island	0.344±0.163	0.749±0.12	-0.404	7.09E-05	2.12E-05	-0.208
cg08613144	2	133428836	*LYPD1*	5'UTR/1st exon	Island	0.314±0.166	0.718±0.153	-0.403	6.58E-05	3.12E-16	-0.388
cg18302606	19	39904402	*PLEKHG2*	5'UTR/1st intron	Island	0.332±0.129	0.734±0.116	-0.402	5.82E-06	3.00E-26	-0.490
cg10380585	1	211500971	*TRAF5*	5'UTR/1st intron	S_Shore	0.298±0.205	0.697±0.198	-0.399	4.69E-04	6.62E-36	-0.564
cg26348348	2	133428993	*LYPD1*	5'UTR/1st exon	S_Shore	0.383±0.204	0.779±0.127	-0.397	5.57E-04	9.07E-20	-0.428
cg02996471	19	3178512	*S1PR4*	TSS1500	N_Shore	0.412±0.181	0.808±0.078	-0.396	3.11E-04	2.33E-18	-0.413
cg08730348	16	31214307	*PYCARD*	TSS1500	S_Shore	0.187±0.124	0.583±0.114	-0.396	4.37E-06	1.17E-16	-0.393
cg08713365	20	327623	*NRSN2*	1st exon	Island	0.314±0.21	0.709±0.172	-0.395	6.17E-04	3.94E-50	-0.647
cg05554936	19	11450261	*RAB3D*	5'UTR/1st exon	Island	0.248±0.177	0.643±0.101	-0.395	2.15E-04	2.33E-25	-0.482
cg23493132	19	54370796	*MYADM*	5'UTR/1st intron	N_Shore	0.333±0.139	0.725±0.1	-0.392	2.36E-05	1.99E-22	-0.455
cg02454890	15	90544015	*ZNF710*	TSS1500	S_Shore	0.332±0.237	0.724±0.258	-0.392	1.89E-03	2.16E-12	-0.337
cg02064724	14	74179953	*PNMA1*	1st exon	N_Shore	0.251±0.14	0.643±0.099	-0.391	2.62E-05	4.49E-29	-0.513
cg03096126	18	56531159	*ZNF532*	5'UTR/1st intron	Island	0.315±0.228	0.702±0.159	-0.388	1.35E-03	2.15E-27	-0.500
cg10523105	14	74178976	*PNMA1*	1st exon/3'UTR	N_Shore	0.367±0.163	0.749±0.074	-0.382	1.85E-04	7.13E-38	-0.577
cg17752112	19	11450515	*RAB3D*	TSS200	S_Shore	0.204±0.138	0.585±0.137	-0.381	1.72E-05	8.16E-28	-0.503
cg08047482	19	11449933	*RAB3D*	5'UTR/1st intron	N_Shore	0.368±0.087	0.749±0.072	-0.381	2.04E-07	3.20E-49	-0.642
cg21583565	2	174219336	*CDCA7*	TSS1500	N_Shore	0.3±0.117	0.677±0.212	-0.377	6.57E-06	1.67E-19	-0.425
cg05214748	16	31214335	*PYCARD*	TSS1500	S_Shore	0.182±0.096	0.559±0.136	-0.377	1.63E-07	8.15E-21	-0.439
cg13880094	19	35491738	*GRAMD1A*	5'UTR/1st intron	Island	0.115±0.086	0.491±0.236	-0.376	4.35E-06	2.64E-19	-0.423
cg14502728	4	78978537	*FRAS1*	TSS200	N_Shore	0.209±0.208	0.585±0.322	-0.376	1.94E-03	1.73E-11	-0.323
cg20664636	17	1959596	*HIC1*	5'UTR/1st intron	Island	0.452±0.137	0.828±0.054	-0.376	6.95E-05	3.04E-06	-0.228
cg14335894	17	47075281	*IGF2BP1*	1st exon	Island	0.137±0.08	0.512±0.233	-0.375	3.26E-06	4.45E-08	-0.266
cg05907835	16	31214346	*PYCARD*	TSS1500	S_Shore	0.159±0.078	0.534±0.136	-0.375	1.08E-08	9.45E-21	-0.438
cg05852537	20	33759753	*PROCR*	5'UTR/1st exon	N_Shelf	0.294±0.159	0.668±0.13	-0.374	9.64E-05	5.21E-22	-0.451
cg23350336	10	70321554	*TET1*	5'UTR/1st intron	S_Shore	0.28±0.182	0.654±0.238	-0.374	3.81E-04	4.08E-06	-0.225
cg00555905	17	79140481	*AATK*	TSS1500	S_Shore	0.345±0.179	0.712±0.178	-0.366	3.08E-04	2.10E-16	-0.390
cg20530204	6	33244709	*B3GALT4*	TSS1500	Island	0.267±0.166	0.631±0.139	-0.363	1.77E-04	3.49E-45	-0.620
cg16904330	3	119299162	*ADPRH*	5'UTR/1st intron	S_Shore	0.392±0.161	0.754±0.063	-0.362	2.69E-04	7.11E-38	-0.577
cg16831623	20	6750617	*BMP2*	5'UTR/1st intron	S_Shore	0.281±0.125	0.643±0.2	-0.362	1.52E-05	1.04E-09	-0.295
cg08543966	19	35646150	*FXYD5*	1st exon	S_Shore	0.276±0.186	0.638±0.131	-0.362	5.06E-04	1.14E-11	-0.326
cg11153694	5	141017797	*RELL2*	1st exon	S_Shore	0.272±0.162	0.634±0.109	-0.362	1.87E-04	8.25E-09	-0.279
cg08584377	7	5632269	*FSCN1*	TSS200	N_Shore	0.302±0.179	0.661±0.229	-0.359	4.67E-04	2.74E-06	-0.229
cg05744675	16	88717723	*CYBA*	TSS1500	N_Shelf	0.251±0.105	0.608±0.17	-0.357	1.70E-06	2.05E-25	-0.483
cg20766090	11	2159131	*IGF2*	5'UTR	Island	0.258±0.075	0.614±0.176	-0.356	1.80E-07	1.17E-21	-0.447
cg07000334	12	54785286	*ZNF385A*	TSS200	S_Shore	0.439±0.169	0.793±0.069	-0.355	4.26E-04	5.17E-10	-0.300
cg20331595	20	327607	*NRSN2*	1st exon	Island	0.162±0.132	0.517±0.174	-0.355	2.14E-05	2.06E-32	-0.539
cg23676480	20	25039812	*ACSS1*	TSS1500	S_Shore	0.359±0.189	0.712±0.123	-0.353	7.39E-04	1.45E-07	-0.256
cg13354988	2	263656	*SH3YL1*	5'UTR/1st intron	Island	0.386±0.155	0.738±0.073	-0.352	2.25E-04	1.38E-36	-0.568
cg25731261	19	47736018	*BBC3*	5'UTR/1st exon	S_Shore	0.382±0.092	0.734±0.097	-0.352	3.95E-07	2.58E-09	-0.288
cg20118643	15	60690852	*ANXA2*	TSS1500	Island	0.363±0.168	0.712±0.175	-0.350	2.62E-04	4.49E-18	-0.409
cg06236559	18	45458303	*SMAD2*	TSS1500	S_Shore	0.444±0.223	0.793±0.124	-0.350	2.46E-03	1.25E-10	-0.310
cg12100791	16	31214417	*PYCARD*	TSS1500	S_Shore	0.379±0.12	0.727±0.112	-0.349	1.14E-05	2.39E-18	-0.412
cg03294491	18	45458243	*SMAD2*	TSS1500	S_Shore	0.424±0.194	0.772±0.12	-0.348	9.90E-04	3.88E-08	-0.267
cg02441711	17	80291105	*SECTM1*	5'UTR/1st intron	Island	0.383±0.14	0.73±0.064	-0.348	1.25E-04	2.61E-15	-0.376
cg08831594	10	71211006	*TSPAN15*	TSS1500	N_Shore	0.144±0.141	0.491±0.199	-0.347	7.03E-05	1.14E-11	-0.326
cg10881225	2	9984929	*TAF1B*	5'UTR/1st intron	S_Shore	0.451±0.16	0.797±0.062	-0.346	3.63E-04	3.35E-11	-0.319
cg15472071	1	16085984	*FBLIM1*	5'UTR	S_Shore	0.381±0.142	0.727±0.109	-0.346	7.82E-05	9.92E-31	-0.526
cg11353035	17	6926414	*BCL6B*	5'UTR/1st exon	Island	0.376±0.115	0.722±0.084	-0.346	1.29E-05	1.38E-07	-0.256
cg07473175	12	47474262	*AMIGO2*	TSS1500	S_Shore	0.288±0.203	0.633±0.186	-0.346	1.34E-03	2.75E-27	-0.499
cg11032268	7	5632272	*FSCN1*	TSS200	N_Shore	0.212±0.144	0.558±0.259	-0.346	2.48E-04	1.19E-05	-0.214
cg07354371	1	43736391	*TMEM125*	5'UTR	N_Shelf	0.351±0.13	0.696±0.065	-0.345	6.77E-05	2.83E-33	-0.545
cg17983217	6	31698223	*DDAH2*	TSS1500	Island	0.414±0.205	0.758±0.068	-0.344	1.87E-03	1.01E-14	-0.369
cg17655624	12	54785320	*ZNF385A*	TSS1500	S_Shore	0.427±0.189	0.77±0.065	-0.344	1.14E-03	6.44E-10	-0.298
cg08096786	16	57571099	*CCDC102A*	TSS1500	S_Shore	0.431±0.225	0.774±0.058	-0.342	3.39E-03	5.73E-17	-0.397
cg24046411	16	88717646	*CYBA*	TSS200	N_Shelf	0.433±0.155	0.774±0.043	-0.341	3.69E-04	8.46E-44	-0.613
cg09142578	17	79140486	*AATK*	TSS1500	S_Shore	0.369±0.166	0.71±0.167	-0.341	2.88E-04	7.77E-18	-0.407
cg16335762	16	66637919	*CMTM3*	5'UTR/1st exon	N_Shore	0.326±0.169	0.666±0.206	-0.341	4.09E-04	2.12E-13	-0.351
cg13682140	17	41978218	*MPP2*	5'UTR	S_Shore	0.329±0.151	0.67±0.152	-0.341	1.26E-04	5.55E-09	-0.282
cg00067414	6	151188081	*MTHFD1L*	1st exon	Island	0.276±0.204	0.616±0.139	-0.340	1.61E-03	2.19E-26	-0.491
cg19725553	7	43797898	*BLVRA*	TSS1500	Island	0.335±0.183	0.675±0.124	-0.339	7.40E-04	2.97E-11	-0.320
cg24337786	19	39108672	*MAP4K1*	5'UTR/1st exon	N_Shore	0.429±0.196	0.768±0.082	-0.338	1.48E-03	3.45E-20	-0.432
cg24940138	17	41363741	*TMEM106A*	TSS200	Island	0.309±0.272	0.646±0.261	-0.338	1.11E-02	2.60E-43	-0.610
cg13916266	17	7787658	*CHD3*	TSS1500	N_Shore	0.248±0.119	0.586±0.177	-0.337	1.54E-05	7.78E-27	-0.495
cg11059617	14	61191084	*SIX4*	TSS1500	S_Shore	0.122±0.168	0.458±0.35	-0.336	2.97E-03	1.19E-06	-0.237
cg04355435	1	43736290	*TMEM125*	5'UTR	N_Shelf	0.318±0.117	0.655±0.06	-0.336	3.67E-05	8.61E-29	-0.511
cg13640200	12	47474202	*AMIGO2*	TSS1500	S_Shore	0.232±0.139	0.568±0.175	-0.336	6.89E-05	2.32E-26	-0.491
cg08794157	17	15164627	*PMP22*	5'UTR/1st intron	Island	0.362±0.112	0.695±0.186	-0.332	1.29E-05	8.85E-08	-0.260
cg19660812	8	38617447	*TACC1*	5'UTR/1st intron	S_Shelf	0.37±0.13	0.702±0.174	-0.332	4.13E-05	4.13E-08	-0.266
cg16725050	2	220117771	*TUBA4A*	5'UTR/1st intron	N_Shore	0.193±0.113	0.525±0.282	-0.332	2.65E-04	1.12E-33	-0.548
cg02183170	14	55595698	*LGALS3*	TSS1500	Island	0.356±0.204	0.688±0.176	-0.332	1.83E-03	2.56E-32	-0.538
cg20728696	11	2158702	*IGF2*	5'UTR	N_Shore	0.23±0.113	0.561±0.183	-0.331	1.31E-05	6.98E-16	-0.383
cg08296680	17	80186275	*SLC16A3*	TSS1500	Island	0.427±0.18	0.758±0.097	-0.331	8.91E-04	2.20E-08	-0.271
cg19156220	6	33244752	*B3GALT4*	TSS200	Island	0.484±0.214	0.815±0.077	-0.331	2.91E-03	4.14E-57	-0.680
cg13163919	15	70388014	*TLE3*	TSS1500	Island	0.362±0.149	0.693±0.101	-0.331	1.94E-04	6.03E-09	-0.282
cg11199810	1	150123146	*PLEKHO1*	5'UTR	S_Shore	0.447±0.109	0.778±0.071	-0.331	1.61E-05	8.12E-17	-0.395
cg22502206	15	60690826	*ANXA2*	TSS1500	Island	0.343±0.176	0.674±0.214	-0.331	7.57E-04	5.31E-18	-0.408
cg02787991	17	80291405	*SECTM1*	5'UTR/1st intron	Island	0.385±0.16	0.715±0.044	-0.330	5.62E-04	6.34E-14	-0.358
cg15786280	4	15779879	*CD38*	TSS200	N_Shore	0.315±0.125	0.644±0.158	-0.329	2.75E-05	2.20E-15	-0.377
cg11131549	1	211500852	*TRAF5*	5'UTR/1st intron	S_Shore	0.256±0.177	0.584±0.192	-0.328	7.43E-04	2.15E-33	-0.546
cg03870138	16	88717850	*CYBA*	TSS1500	N_Shelf	0.245±0.088	0.573±0.154	-0.328	6.60E-07	2.71E-25	-0.481
cg02086310	20	25039719	*ACSS1*	TSS1500	S_Shore	0.365±0.092	0.691±0.106	-0.327	7.05E-07	5.90E-08	-0.263
cg15988792	20	25039314	*ACSS1*	TSS1500	S_Shore	0.359±0.139	0.685±0.067	-0.326	1.85E-04	9.52E-13	-0.342

cg26705574	14	24836219	*NFATC4*	5'UTR/1st exon	N_Shore	0.269±0.147	0.594±0.166	-0.326	1.55E-04	1.47E-08	-0.275
cg06744119	10	88730599	*AGAP11*	5'UTR/1st exon	S_Shore	0.402±0.163	0.726±0.113	-0.324	4.40E-04	3.06E-10	-0.304
cg15026277	17	41363789	*TMEM106A*	TSS200	Island	0.268±0.261	0.59±0.26	-0.323	1.18E-02	1.91E-42	-0.605
cg03710719	19	36389795	*NFKBID*	5'UTR/1st intron	N_Shore	0.378±0.171	0.7±0.103	-0.323	7.23E-04	8.50E-15	-0.370
cg18923721	17	8868669	*PIK3R5*	5'UTR/1st intron	Island	0.305±0.126	0.628±0.141	-0.322	3.45E-05	1.83E-13	-0.352
cg10246531	16	88717628	*CYBA*	TSS200	N_Shelf	0.509±0.135	0.831±0.042	-0.322	2.05E-04	1.88E-36	-0.567
cg04153784	7	127672235	*LRRC4*	TSS1500	Island	0.35±0.112	0.671±0.106	-0.322	1.22E-05	9.53E-10	-0.296
cg15543281	6	33245181	*B3GALT4*	5'UTR/1st exon	Island	0.468±0.195	0.789±0.064	-0.321	2.06E-03	6.64E-67	-0.719
cg05057777	12	104697631	*EID3*	5'UTR/1st exon	Island	0.325±0.176	0.647±0.184	-0.321	8.27E-04	1.92E-17	-0.402
cg18861421	19	11450590	*RAB3D*	TSS1500	S_Shore	0.378±0.056	0.699±0.072	-0.321	5.35E-10	2.85E-28	-0.507
cg04212150	18	56531132	*ZNF532*	5'UTR/1st intron	Island	0.269±0.145	0.59±0.147	-0.321	4.17E-04	3.01E-25	-0.481
cg11772919	6	33245328	*B3GALT4*	1st exon	Island	0.493±0.176	0.814±0.054	-0.321	1.16E-03	5.49E-64	-0.708
cg03565081	14	23478970	*C14orf93*	5'UTR/1st intron	S_Shelf	0.455±0.169	0.775±0.097	-0.321	7.11E-04	9.97E-08	-0.259
cg05381423	19	5340713	*PTPRS*	5'UTR/1st intron	Island	0.299±0.159	0.619±0.234	-0.320	6.15E-04	4.13E-08	-0.266
cg13969273	16	69760564	*NQO1*	5'UTR/1st exon	S_Shore	0.243±0.179	0.563±0.274	-0.320	2.06E-03	6.89E-26	-0.487
cg11073571	4	78978565	*FRAS1*	TSS200	N_Shore	0.158±0.174	0.478±0.29	-0.320	2.15E-03	8.49E-07	-0.240
cg08116775	8	135726595	*ZFAT*	TSS1500	S_Shore	0.502±0.134	0.822±0.054	-0.320	1.76E-04	1.14E-05	-0.214
cg14897419	10	134121413	*STK32C*	5'UTR/1st exon	Island	0.192±0.174	0.511±0.274	-0.319	1.85E-03	7.68E-07	-0.241
cg26110130	1	42128455	*HIVEP3*	5'UTR	Island	0.353±0.206	0.672±0.167	-0.319	2.62E-03	1.96E-07	-0.253
cg14185025	14	100259341	*EML1*	TSS1500	Island	0.21±0.163	0.528±0.22	-0.318	6.69E-04	1.05E-05	-0.215
cg18107893	19	33166844	*RGS9BP*	5'UTR/1st exon	Island	0.31±0.165	0.627±0.138	-0.317	5.25E-04	3.70E-08	-0.267
cg18107893	19	33166844	*ANKRD27*	TSS1500	Island	0.31±0.165	0.627±0.138	-0.317	5.25E-04	2.18E-05	-0.208
cg21211480	17	41363774	*TMEM106A*	TSS200	Island	0.259±0.257	0.576±0.283	-0.317	1.32E-02	4.59E-42	-0.602
cg17322655	2	242802127	*PDCD1*	TSS1500	N_Shelf	0.384±0.149	0.7±0.156	-0.316	2.26E-04	3.16E-09	-0.287
cg07533239	12	6422131	*PLEKHG6*	5'UTR/1st exon	S_Shelf	0.339±0.097	0.655±0.137	-0.316	2.14E-06	1.50E-05	-0.211
cg09587549	16	31214126	*PYCARD*	TSS200	Island	0.25±0.149	0.565±0.198	-0.316	2.89E-04	1.59E-08	-0.274
cg00588621	12	104697620	*EID3*	5'UTR/1st exon	Island	0.364±0.191	0.679±0.185	-0.316	1.73E-03	7.42E-18	-0.407
cg10521852	19	19739820	*LPAR2*	TSS1500	S_Shore	0.473±0.173	0.788±0.095	-0.315	9.58E-04	1.62E-20	-0.436
cg20785674	1	150123490	*PLEKHO1*	5'UTR	S_Shore	0.345±0.072	0.66±0.114	-0.315	3.36E-08	2.99E-23	-0.463
cg26259171	19	49255779	*FUT1*	5'UTR	S_Shore	0.405±0.136	0.721±0.072	-0.315	1.82E-04	2.26E-10	-0.306
cg24008544	17	41363899	*TMEM106A*	5'UTR/1st exon	Island	0.287±0.233	0.602±0.241	-0.315	7.12E-03	3.00E-43	-0.609
cg23859630	6	32160455	*GPSM3*	5'UTR	N_Shelf	0.336±0.078	0.65±0.186	-0.314	2.92E-06	2.94E-12	-0.335
cg17382541	19	54370839	*MYADM*	5'UTR/1st intron	N_Shore	0.351±0.163	0.665±0.149	-0.314	4.96E-04	3.92E-14	-0.361
cg13038241	7	5632281	*FSCN1*	TSS200	N_Shore	0.343±0.152	0.657±0.189	-0.314	3.25E-04	2.21E-07	-0.252
cg03780927	11	118781150	*BCL9L*	5'UTR/1st exon	Island	0.393±0.156	0.707±0.199	-0.314	4.41E-04	3.72E-15	-0.374
cg18348142	12	47474055	*AMIGO2*	TSS1500	Island	0.264±0.192	0.577±0.259	-0.314	2.92E-03	1.55E-19	-0.425
cg16367511	2	74425523	*MTHFD2*	TSS200	Island	0.408±0.161	0.721±0.074	-0.313	6.60E-04	4.83E-50	-0.646
cg16977706	11	2160129	*IGF2*	5'UTR	Island	0.419±0.178	0.731±0.151	-0.312	1.06E-03	8.23E-20	-0.428
cg13544946	9	35729779	*TLN1*	5'UTR/1st intron	N_Shelf	0.439±0.138	0.751±0.099	-0.311	1.61E-04	6.06E-11	-0.315
cg24941681	20	33759921	*PROCR*	5'UTR/1st exon	N_Shelf	0.351±0.146	0.662±0.09	-0.311	2.82E-04	8.38E-26	-0.486
cg25287474	9	14693538	*ZDHHC21*	1st exon	Island	0.223±0.262	0.534±0.196	-0.311	1.23E-02	5.95E-06	-0.221
cg09513380	13	100622253	*ZIC5*	TSS1500	Island	0.32±0.176	0.63±0.099	-0.310	1.18E-03	1.40E-14	-0.367
cg08640923	2	220117945	*TUBA4A*	5'UTR/1st intron	Island	0.142±0.14	0.452±0.305	-0.309	1.67E-03	4.11E-29	-0.514
cg05270634	17	41177445	*RND2*	1st exon	S_Shore	0.426±0.183	0.735±0.153	-0.308	1.40E-03	2.58E-20	-0.434
cg10195295	12	21809973	*LDHB*	5'UTR/1st intron	N_Shore	0.349±0.168	0.657±0.119	-0.308	8.03E-04	4.04E-27	-0.497
cg25296938	7	98976900	*ARPC1B*	5'UTR/1st intron	S_Shelf	0.34±0.197	0.648±0.143	-0.308	2.42E-03	2.53E-14	-0.364
cg13251484	14	74180217	*PNMA1*	1st exon	N_Shore	0.28±0.113	0.588±0.1	-0.308	2.40E-05	3.24E-27	-0.498
cg27165794	14	74179674	*PNMA1*	1st exon	N_Shore	0.399±0.135	0.707±0.055	-0.307	2.43E-04	1.01E-37	-0.576
cg09043518	17	80290106	*SECTM1*	5'UTR/1st intron	N_Shore	0.516±0.175	0.823±0.063	-0.307	1.39E-03	6.16E-16	-0.384
cg24874612	17	4689765	*VMO1*	TSS200	S_Shelf	0.367±0.148	0.675±0.072	-0.307	4.06E-04	2.60E-05	-0.206
cg08343042	2	135476907	*TMEM163*	TSS1500	Island	0.355±0.259	0.662±0.185	-0.307	1.22E-02	3.31E-06	-0.227
cg18222083	17	41364007	*TMEM106A*	5'UTR/1st intron	Island	0.396±0.268	0.703±0.267	-0.307	1.78E-02	4.50E-43	-0.608
cg08396863	2	220117599	*TUBA4A*	5'UTR/1st intron	N_Shore	0.214±0.114	0.521±0.272	-0.307	4.70E-04	2.05E-38	-0.580
cg14302224	10	105210980	*CALHM2*	5'UTR	N_Shore	0.253±0.133	0.559±0.207	-0.306	2.04E-04	4.31E-07	-0.246
cg13334650	11	6440065	*APBB1*	5'UTR/1st intron	Island	0.347±0.168	0.652±0.224	-0.306	1.17E-03	5.12E-10	-0.300
cg17260706	11	118782879	*BCL9L*	TSS1500	S_Shore	0.417±0.086	0.723±0.055	-0.306	4.39E-06	3.37E-19	-0.422
cg22708738	19	50316212	*FUZ*	5'UTR/1st intron	S_Shelf	0.449±0.171	0.755±0.129	-0.306	9.48E-04	2.87E-12	-0.335
cg08377924	17	41363628	*TMEM106A*	TSS1500	N_Shore	0.315±0.259	0.62±0.243	-0.305	1.47E-02	5.23E-42	-0.602
cg25559421	5	157003155	*ADAM19*	TSS1500	S_Shore	0.337±0.155	0.642±0.191	-0.305	5.30E-04	2.56E-08	-0.270
cg19664267	6	33245163	*B3GALT4*	5'UTR/1st exon	Island	0.526±0.19	0.831±0.05	-0.304	2.53E-03	3.37E-68	-0.724
cg24406344	2	97167519	*NEURL3*	5'UTR	S_Shore	0.303±0.083	0.607±0.109	-0.304	4.20E-07	3.85E-06	-0.225
cg20577019	1	150123077	*PLEKHO1*	5'UTR/1st exon	Island	0.424±0.051	0.727±0.056	-0.304	9.40E-10	8.22E-12	-0.328
cg13438961	1	41250071	*KCNQ4*	1st exon	Island	0.486±0.127	0.789±0.076	-0.303	1.27E-04	5.99E-07	-0.243
cg21766592	19	47288066	*SLC1A5*	5'UTR/1st exon	N_Shelf	0.18±0.087	0.483±0.16	-0.303	2.43E-06	5.63E-18	-0.408
cg18054578	19	47735960	*BBC3*	5'UTR/1st exon	S_Shore	0.102±0.035	0.405±0.179	-0.303	1.18E-06	9.33E-07	-0.239
cg06315390	22	45704914	*FAM118A*	1st exon	N_Shore	0.522±0.146	0.825±0.068	-0.303	4.30E-04	3.81E-05	-0.201
cg09874289	19	17008193	*CPAMD8*	TSS1500	Island	0.159±0.107	0.462±0.189	-0.303	3.32E-05	3.12E-06	-0.227
cg24191285	17	73893206	*TRIM65*	TSS200	Island	0.39±0.14	0.692±0.122	-0.303	2.00E-04	8.58E-14	-0.357
cg08135379	12	47474763	*AMIGO2*	TSS1500	S_Shore	0.414±0.174	0.715±0.104	-0.301	1.30E-03	1.40E-26	-0.493
cg13848707	10	70321243	*TET1*	5'UTR/1st intron	S_Shore	0.282±0.144	0.583±0.223	-0.301	5.26E-04	1.95E-09	-0.290
cg18721397	5	32584912	*SUB1*	TSS1500	N_Shore	0.509±0.213	0.809±0.066	-0.300	4.94E-03	4.69E-10	-0.301
cg10633838	6	33245359	*B3GALT4*	1st exon	Island	0.429±0.128	0.73±0.05	-0.300	2.02E-04	1.52E-57	-0.681
cg27278017	20	33759645	*PROCR*	TSS200	N_Shelf	0.423±0.16	0.723±0.056	-0.300	9.42E-04	8.36E-31	-0.527
cg00930615	15	60690667	*ANXA2*	TSS1500	N_Shore	0.31±0.176	0.61±0.261	-0.300	2.76E-03	6.66E-17	-0.396
cg03321508	20	25039707	*ACSS1*	TSS1500	S_Shore	0.366±0.116	0.665±0.123	-0.299	3.13E-05	7.78E-10	-0.297
cg19256731	20	1874527	*SIRPA*	TSS1500	N_Shore	0.544±0.208	0.844±0.061	-0.299	4.46E-03	1.54E-11	-0.324
cg11348442	2	220117784	*TUBA4A*	5'UTR/1st intron	N_Shore	0.186±0.156	0.486±0.271	-0.299	1.79E-03	1.41E-34	-0.554
cg06605158	20	33759940	*PROCR*	5'UTR/1st exon	N_Shelf	0.374±0.154	0.672±0.099	-0.299	5.60E-04	4.98E-27	-0.496
cg12798097	20	44034258	*DBNDD2*	TSS1500	N_Shore	0.277±0.101	0.575±0.132	-0.299	6.81E-06	2.81E-15	-0.376
cg22902177	3	129324717	*PLXND1*	1st exon	Island	0.314±0.241	0.612±0.205	-0.299	1.05E-02	1.66E-07	-0.254
cg12773325	11	2160560	*IGF2*	5'UTR	Island	0.414±0.24	0.713±0.195	-0.299	9.92E-03	1.14E-13	-0.355
cg26598152	16	69760928	*NQO1*	TSS1500	S_Shore	0.182±0.125	0.48±0.263	-0.299	6.47E-04	4.93E-28	-0.505
cg08438690	3	52279403	*PPM1M*	TSS1500	N_Shore	0.526±0.129	0.825±0.038	-0.298	2.57E-04	3.80E-14	-0.361
cg16336494	6	151188259	*MTHFD1L*	1st exon	S_Shore	0.457±0.121	0.755±0.05	-0.298	1.48E-04	2.40E-26	-0.491
cg00054525	16	88717587	*CYBA*	TSS200	N_Shelf	0.381±0.123	0.679±0.055	-0.298	1.53E-04	1.07E-38	-0.582
cg07003632	14	100259329	*EML1*	TSS1500	Island	0.253±0.121	0.55±0.204	-0.298	1.37E-04	1.37E-07	-0.256
cg06461306	7	767490	*PRKAR1B*	TSS1500	S_Shore	0.313±0.163	0.61±0.205	-0.297	1.05E-03	2.64E-11	-0.321

cg25587662	18	10525447	*NAPG*	TSS1500	N_Shore	0.492±0.148	0.789±0.077	-0.297	5.13E-04	3.59E-16	-0.387
cg20276750	3	52279555	*PPM1M*	TSS1500	N_Shore	0.327±0.186	0.624±0.113	-0.297	2.14E-03	6.21E-27	-0.496
cg03786933	16	69760418	*NQO1*	5'UTR/1st exon	Island	0.262±0.205	0.558±0.268	-0.297	6.48E-03	9.79E-16	-0.382
cg09137630	19	47618017	*ZC3H4*	TSS1500	S_Shelf	0.37±0.14	0.666±0.092	-0.297	2.94E-04	2.24E-06	-0.231
cg07839457	16	57023022	*NLRC5*	TSS1500	N_Shore	0.302±0.166	0.599±0.183	-0.297	1.02E-03	2.93E-35	-0.559
cg27176870	19	38754930	*SPINT2*	TSS200	Island	0.308±0.19	0.604±0.118	-0.296	2.52E-03	7.87E-24	-0.468
cg10708675	16	69760758	*NQO1*	TSS1500	S_Shore	0.232±0.163	0.529±0.275	-0.296	2.53E-03	7.45E-29	-0.512
cg02467765	4	78978708	*FRAS1*	TSS200	N_Shore	0.201±0.136	0.497±0.235	-0.296	5.62E-04	6.94E-09	-0.280
cg06933574	5	134370282	*PITX1*	TSS1500	S_Shelf	0.254±0.245	0.549±0.228	-0.296	1.27E-02	1.10E-07	-0.258
cg19764436	22	23413260	*GNAZ*	5'UTR/1st intron	Island	0.237±0.096	0.533±0.156	-0.296	7.41E-06	1.65E-23	-0.465
cg19074496	2	220117868	*TUBA4A*	5'UTR/1st intron	N_Shore	0.117±0.094	0.413±0.288	-0.296	6.96E-04	3.86E-30	-0.522
cg20923245	12	104697419	*EID3*	TSS200	Island	0.295±0.159	0.59±0.211	-0.295	1.01E-03	9.12E-14	-0.356
cg15768226	20	55965245	*RBM38*	TSS1500	S_Shore	0.5±0.205	0.795±0.056	-0.295	4.51E-03	3.61E-14	-0.362
cg19548479	17	41363737	*TMEM106A*	TSS200	Island	0.332±0.248	0.627±0.238	-0.295	1.40E-02	2.46E-43	-0.610
cg18696027	15	45002597	*B2M*	TSS1500	N_Shore	0.509±0.226	0.804±0.101	-0.295	7.22E-03	8.20E-24	-0.468
cg24049348	8	17433926	*PDGFRL*	TSS200	N_Shore	0.283±0.163	0.577±0.254	-0.295	2.00E-03	7.09E-12	-0.329
cg02448597	6	32160605	*GPSM3*	5'UTR	N_Shelf	0.476±0.116	0.77±0.045	-0.294	1.19E-04	4.48E-49	-0.641
cg00157962	1	109102212	*FAM102B*	TSS1500	N_Shore	0.469±0.115	0.763±0.096	-0.294	4.70E-05	1.30E-13	-0.354
cg18087943	11	2160540	*IGF2*	5'UTR	Island	0.407±0.237	0.701±0.208	-0.294	1.04E-02	4.01E-17	-0.398
cg18486258	6	42110750	*C6orf132*	TSS200	S_Shore	0.367±0.153	0.661±0.144	-0.294	5.18E-04	1.05E-17	-0.405
cg17441401	1	53794150	*LRP8*	TSS1500	S_Shore	0.303±0.172	0.596±0.209	-0.294	1.68E-03	5.91E-12	-0.330
cg11854806	10	82116365	*DYDC1*	5'UTR/1st intron	Island	0.393±0.118	0.686±0.172	-0.293	6.85E-05	8.91E-12	-0.328
cg17510056	20	8112972	*PLCB1*	5'UTR/1st exon	Island	0.1±0.212	0.393±0.32	-0.293	1.23E-02	3.59E-08	-0.267
cg07611334	20	327811	*NRSN2*	5'UTR/1st intron	Island	0.216±0.144	0.509±0.197	-0.293	4.67E-04	1.43E-27	-0.501
cg01186777	19	6531016	*TNFSF9*	5'UTR/1st exon	Island	0.196±0.168	0.488±0.244	-0.292	2.20E-03	1.30E-08	-0.276
cg18055007	6	31698226	*DDAH2*	TSS1500	Island	0.501±0.192	0.792±0.054	-0.292	3.39E-03	1.58E-18	-0.414
cg00630164	1	41250062	*KCNQ4*	1st exon	Island	0.561±0.13	0.853±0.06	-0.292	2.52E-04	6.70E-08	-0.262
cg03817911	12	104697389	*EID3*	TSS200	Island	0.31±0.195	0.602±0.251	-0.292	5.00E-03	2.84E-13	-0.349
cg27098900	6	33245128	*B3GALT4*	5'UTR/1st exon	Island	0.463±0.211	0.754±0.059	-0.291	5.54E-03	3.38E-63	-0.705
cg10572943	21	34775045	*IFNGR2*	TSS200	N_Shore	0.482±0.093	0.774±0.054	-0.291	1.49E-05	2.28E-16	-0.389
cg23320056	1	155948742	*ARHGEF2*	5'UTR/1st exon	S_Shore	0.339±0.167	0.63±0.158	-0.291	1.12E-03	7.96E-64	-0.708
cg00820919	2	43863839	*PLEKHH2*	TSS1500	N_Shore	0.337±0.148	0.628±0.133	-0.291	4.23E-04	7.58E-09	-0.280
cg07156249	6	32822911	*TAP1*	TSS1500	S_Shore	0.24±0.169	0.53±0.193	-0.290	1.48E-03	2.50E-18	-0.412
cg00124375	6	31698218	*DDAH2*	TSS1500	Island	0.418±0.194	0.708±0.066	-0.290	3.59E-03	2.46E-15	-0.377
cg26365553	11	47290613	*MADD*	TSS1500	N_Shore	0.239±0.145	0.528±0.209	-0.290	6.16E-04	5.70E-10	-0.299
cg26876834	16	2013573	*SNHG9*	TSS1500	N_Shore	0.489±0.217	0.779±0.236	-0.290	8.17E-03	2.18E-06	-0.231
cg02226939	17	28619481	*BLMH*	TSS1500	S_Shore	0.252±0.157	0.541±0.167	-0.289	7.47E-04	3.73E-10	-0.302
cg10864952	7	65671987	*TPST1*	5'UTR/1st intron	S_Shore	0.33±0.102	0.62±0.182	-0.289	3.25E-05	5.74E-26	-0.487
cg25277632	10	14995476	*DCLRE1C*	5'UTR/1st intron	N_Shore	0.205±0.165	0.494±0.238	-0.289	2.00E-03	8.14E-41	-0.595
cg26251270	1	53067832	*GPX7*	TSS1500	N_Shore	0.211±0.091	0.5±0.171	-0.289	1.08E-05	8.36E-44	-0.613
cg13365340	6	33245342	*B3GALT4*	1st exon	Island	0.457±0.163	0.746±0.055	-0.289	1.33E-03	1.19E-64	-0.711
cg27297851	19	36389833	*NFKBID*	5'UTR/1st intron	N_Shore	0.443±0.161	0.732±0.107	-0.289	9.76E-04	1.85E-19	-0.425
cg24497361	11	3858493	*RHOG*	5'UTR/1st intron	N_Shelf	0.458±0.141	0.747±0.062	-0.289	4.81E-04	5.97E-17	-0.396
cg20715295	19	42807159	*PAFAH1B3*	TSS1500	N_Shelf	0.446±0.076	0.734±0.085	-0.288	3.42E-07	3.05E-06	-0.228
cg06767766	10	70321383	*TET1*	5'UTR/1st intron	S_Shore	0.332±0.169	0.62±0.21	-0.288	1.77E-03	1.87E-10	-0.307
cg22797644	6	34206491	*HMGA1*	5'UTR	S_Shore	0.456±0.105	0.744±0.108	-0.288	1.71E-05	6.31E-28	-0.504
cg23624705	1	92952655	*GFI1*	TSS1500	S_Shore	0.443±0.137	0.73±0.128	-0.288	2.50E-04	6.92E-15	-0.371
cg17215278	1	117114263	*CD58*	TSS1500	S_Shore	0.493±0.187	0.781±0.09	-0.288	2.90E-03	3.40E-05	-0.203
cg26357885	14	65006204	*HSPA2*	TSS1500	N_Shore	0.294±0.121	0.581±0.17	-0.287	1.01E-04	2.16E-07	-0.252
cg24000908	5	1113358	*SLC12A7*	TSS1500	S_Shore	0.45±0.117	0.737±0.113	-0.287	5.79E-05	3.54E-08	-0.267
cg00859858	1	37944257	*ZC3H12A*	5'UTR	S_Shelf	0.279±0.118	0.566±0.223	-0.287	2.88E-04	9.90E-10	-0.295
cg25387487	5	157003181	*ADAM19*	TSS1500	S_Shore	0.302±0.16	0.588±0.208	-0.287	1.27E-03	1.23E-09	-0.294
cg05106294	11	12031097	*DKK3*	TSS1500	S_Shore	0.414±0.191	0.701±0.13	-0.287	3.14E-03	9.41E-09	-0.278
cg19268695	1	111743411	*DENND2D*	TSS200	N_Shelf	0.39±0.126	0.676±0.134	-0.286	1.19E-04	3.15E-30	-0.522
cg25207447	6	43043831	*PTK7*	TSS1500	N_Shore	0.371±0.13	0.658±0.124	-0.286	1.52E-04	3.99E-18	-0.410
cg02183671	4	15779999	*CD38*	1st exon	Island	0.388±0.204	0.674±0.166	-0.286	4.83E-03	1.34E-11	-0.325
cg07461772	16	68482809	*SMPD3*	TSS1500	Island	0.356±0.205	0.642±0.165	-0.286	5.10E-03	4.04E-06	-0.225
cg18996663	14	55595768	*LGALS3*	TSS200	Island	0.219±0.181	0.505±0.189	-0.286	2.55E-03	8.21E-18	-0.406
cg08986368	11	2159122	*IGF2*	5'UTR	Island	0.415±0.157	0.7±0.107	-0.285	8.80E-04	4.04E-26	-0.489
cg06641285	17	76921829	*TIMP2*	TSS1500	Island	0.365±0.188	0.65±0.225	-0.285	4.07E-03	1.49E-08	-0.274
cg04643969	11	63753210	*OTUB1*	TSS200	N_Shore	0.361±0.096	0.645±0.16	-0.284	1.36E-05	6.33E-10	-0.298
cg10025300	2	135476909	*TMEM163*	TSS1500	Island	0.301±0.172	0.585±0.176	-0.284	1.76E-03	3.81E-05	-0.201
cg11856711	12	54785309	*ZNF385A*	TSS1500	S_Shore	0.368±0.143	0.652±0.104	-0.284	4.19E-04	6.34E-08	-0.263
cg25424659	19	11450592	*RAB3D*	TSS1500	S_Shore	0.424±0.048	0.708±0.073	-0.284	2.27E-10	1.10E-26	-0.494
cg17300736	11	2162475	*IGF2*	5'UTR	Island	0.468±0.109	0.752±0.154	-0.284	3.75E-05	1.01E-09	-0.295
cg01520924	14	65006170	*HSPA2*	TSS1500	N_Shore	0.268±0.12	0.552±0.181	-0.284	1.36E-04	1.50E-05	-0.211
cg08341874	17	4689655	*VMO1*	5'UTR/1st exon	S_Shelf	0.378±0.146	0.662±0.071	-0.284	6.56E-04	1.41E-05	-0.212
cg10169539	14	51296612	*NIN*	5'UTR	N_Shore	0.15±0.063	0.433±0.224	-0.284	6.04E-05	2.79E-08	-0.269
cg27657537	22	20863762	*MED15*	5'UTR/1st intron	S_Shore	0.249±0.073	0.532±0.16	-0.283	2.17E-06	3.24E-20	-0.433
cg06638433	17	47075175	*IGF2BP1*	1st exon	Island	0.352±0.138	0.635±0.144	-0.283	3.05E-04	1.22E-15	-0.380
cg15087147	7	127672169	*LRRC4*	TSS1500	Island	0.377±0.109	0.66±0.086	-0.283	4.33E-05	2.52E-10	-0.305
cg10162691	17	41177409	*RND2*	1st exon	S_Shore	0.443±0.177	0.725±0.153	-0.282	2.15E-03	1.35E-21	-0.447
cg11537406	11	124932624	*SLC37A2*	TSS1500	N_Shore	0.479±0.229	0.76±0.117	-0.282	9.93E-03	8.92E-06	-0.217
cg26392828	14	61191075	*SIX4*	TSS1500	S_Shore	0.1±0.115	0.381±0.269	-0.281	1.02E-03	4.94E-07	-0.245
cg26607748	9	35690226	*TPM2*	TSS200	Island	0.421±0.069	0.702±0.077	-0.281	1.52E-07	2.38E-15	-0.377
cg22212414	21	34775032	*IFNGR2*	TSS200	N_Shore	0.544±0.112	0.825±0.053	-0.281	1.14E-04	2.19E-15	-0.377
cg08662017	11	12031093	*DKK3*	TSS1500	S_Shore	0.373±0.137	0.653±0.148	-0.281	2.99E-04	7.51E-10	-0.297
cg07730848	5	6379108	*MED10*	TSS1500	S_Shore	0.451±0.183	0.732±0.122	-0.281	2.70E-03	1.14E-05	-0.214
cg08148261	12	21810960	*LDHB*	TSS1500	S_Shore	0.428±0.139	0.708±0.073	-0.280	4.85E-04	4.30E-27	-0.497
cg23681213	14	74180391	*PNMA1*	5'UTR/1st exon	N_Shore	0.171±0.123	0.451±0.173	-0.280	1.70E-04	3.52E-30	-0.522
cg02245612	19	5293611	*PTPRS*	5'UTR/1st intron	S_Shelf	0.173±0.146	0.453±0.244	-0.280	1.56E-03	7.43E-11	-0.314
cg16996144	1	67217950	*TCTEX1D1*	TSS200	N_Shore	0.383±0.197	0.663±0.096	-0.279	4.58E-03	2.14E-09	-0.289
cg05973866	6	33244715	*B3GALT4*	TSS1500	Island	0.162±0.096	0.441±0.14	-0.279	1.10E-05	3.07E-27	-0.498
cg27522060	15	65360366	*RASL12*	5'UTR/1st exon	Island	0.336±0.053	0.615±0.159	-0.279	8.70E-07	3.89E-07	-0.247
cg25849281	1	8937077	*ENO1*	5'UTR/1st intron	N_Shore	0.352±0.155	0.631±0.158	-0.278	9.35E-04	7.83E-33	-0.542
cg08218101	4	15704393	*BST1*	TSS200	N_Shore	0.356±0.14	0.634±0.181	-0.278	5.03E-04	6.49E-30	-0.520
cg18503260	8	86157410	*CA13*	TSS1500	N_Shore	0.306±0.189	0.584±0.171	-0.278	3.81E-03	1.57E-05	-0.211

cg08378442	2	220117704	*TUBA4A*	5'UTR/1st intron	N_Shore	0.134±0.089	0.412±0.263	-0.278	5.71E-04	4.71E-33	-0.544
cg18670767	22	45706726	*FAM118A*	5'UTR	S_Shore	0.267±0.114	0.544±0.17	-0.277	9.09E-05	1.10E-12	-0.341
cg26418880	12	56123972	*CD63*	TSS1500	S_Shore	0.44±0.164	0.717±0.064	-0.277	1.73E-03	3.74E-08	-0.267
cg02589695	1	206730999	*RASSF5*	1st exon	S_Shore	0.414±0.134	0.691±0.095	-0.277	3.19E-04	4.05E-05	-0.201
cg02774862	10	70321580	*TET1*	5'UTR/1st intron	S_Shore	0.381±0.169	0.658±0.197	-0.277	2.14E-03	2.62E-08	-0.270
cg14608156	11	2160554	*IGF2*	5'UTR	Island	0.424±0.245	0.701±0.197	-0.277	1.67E-02	6.11E-15	-0.372
cg26569469	1	110753895	*KCNC4*	1st exon	Island	0.168±0.113	0.444±0.246	-0.277	6.34E-04	1.27E-05	-0.213
cg17372806	11	63448356	*RTN3*	TSS1500	N_Shore	0.467±0.099	0.744±0.087	-0.276	1.78E-05	3.81E-09	-0.285
cg15274684	21	48054897	*PRMT2*	TSS1500	N_Shore	0.219±0.112	0.496±0.25	-0.276	6.98E-04	2.07E-06	-0.231
cg19731268	10	73724697	*CHST3*	5'UTR/1st intron	Island	0.238±0.106	0.515±0.207	-0.276	1.61E-04	1.66E-06	-0.233
cg23305567	19	54369576	*MYADM*	5'UTR/1st exon	N_Shelf	0.481±0.13	0.757±0.07	-0.276	3.31E-04	4.05E-11	-0.318
cg26806924	20	33760017	*PROCR*	1st exon	N_Shelf	0.324±0.128	0.6±0.108	-0.276	2.07E-04	6.27E-24	-0.469
cg09350274	5	137610396	*GFRA3*	TSS200	S_Shore	0.5±0.145	0.776±0.061	-0.276	8.19E-04	2.24E-18	-0.413
cg09507934	7	102072549	*ORAI2*	TSS1500	N_Shore	0.536±0.125	0.812±0.047	-0.276	3.21E-04	3.10E-11	-0.320
cg21837069	1	1369466	*VWA1*	TSS1500	N_Shore	0.463±0.094	0.739±0.044	-0.275	3.53E-05	1.80E-16	-0.391
cg07435294	11	65554356	*OVOL1*	TSS200	Island	0.226±0.157	0.502±0.223	-0.275	1.90E-03	2.14E-05	-0.208
cg03524572	20	62284488	*STMN3*	TSS1500	Island	0.427±0.256	0.702±0.192	-0.275	2.03E-02	4.20E-09	-0.284
cg11257888	1	24828096	*RCAN3*	TSS1500	N_Shore	0.549±0.1	0.824±0.079	-0.275	2.47E-05	1.74E-06	-0.233
cg25666919	7	102074615	*ORAI2*	5'UTR/1st intron	S_Shore	0.367±0.061	0.642±0.102	-0.275	2.44E-08	2.46E-30	-0.523
cg06421614	17	7121116	*DLG4*	TSS200	S_Shelf	0.324±0.092	0.599±0.125	-0.275	6.67E-06	1.36E-16	-0.392
cg04868764	15	41137019	*SPINT1*	1st exon	Island	0.275±0.157	0.55±0.165	-0.274	1.19E-03	1.53E-20	-0.436
cg09516362	16	69760459	*NQO1*	5'UTR/1st exon	Island	0.138±0.129	0.412±0.243	-0.274	1.05E-03	1.82E-14	-0.366
cg06228542	12	56123961	*CD63*	TSS1500	S_Shore	0.5±0.178	0.774±0.059	-0.274	3.08E-03	2.53E-07	-0.251
cg17483510	3	179168677	*GNB4*	5'UTR/1st intron	N_Shore	0.456±0.135	0.73±0.103	-0.273	3.72E-04	4.03E-31	-0.529
cg15510249	3	182970850	*B3GNT5*	TSS200	N_Shore	0.211±0.146	0.484±0.269	-0.273	2.83E-03	5.89E-18	-0.408
cg19457477	19	39108609	*MAP4K1*	5'UTR/1st exon	N_Shore	0.473±0.168	0.746±0.097	-0.273	1.93E-03	5.25E-18	-0.409
cg09238801	14	74179101	*PNMA1*	1st exon/3'UTR	N_Shore	0.451±0.131	0.725±0.06	-0.273	4.10E-04	1.30E-36	-0.568
cg25364343	18	21719286	*CABYR*	5'UTR/1st intron	Island	0.338±0.226	0.61±0.27	-0.272	1.70E-02	8.42E-06	-0.217
cg11864490	20	33759605	*PROCR*	TSS200	N_Shelf	0.357±0.155	0.63±0.098	-0.272	1.13E-03	1.27E-18	-0.415
cg20388732	17	40439433	*STAT5A*	TSS1500	N_Shore	0.481±0.055	0.754±0.077	-0.272	4.68E-09	2.01E-30	-0.524
cg11036831	10	82116382	*DYDC1*	5'UTR/1st intron	Island	0.349±0.089	0.621±0.149	-0.272	8.53E-06	2.21E-11	-0.322
cg21663122	6	26250936	*HIST1H2BH*	TSS1500	S_Shelf	0.446±0.141	0.717±0.153	-0.272	5.36E-04	1.24E-05	-0.213
cg03204605	4	7941837	*AFAP1*	TSS200	Island	0.318±0.201	0.59±0.258	-0.271	9.76E-03	1.02E-18	-0.416
cg24674635	20	6750696	*BMP2*	5'UTR/1st intron	S_Shore	0.27±0.109	0.542±0.187	-0.271	1.29E-04	1.07E-09	-0.295
cg16890093	6	32813084	*PSMB8*	TSS1500	S_Shore	0.504±0.15	0.775±0.09	-0.271	9.52E-04	1.81E-21	-0.445
cg04524477	17	41363597	*TMEM106A*	TSS1500	N_Shore	0.307±0.223	0.578±0.25	-0.271	1.48E-02	4.55E-38	-0.578
cg17936488	9	134151519	*FAM78A*	1st exon	N_Shore	0.469±0.145	0.74±0.065	-0.271	8.74E-04	1.04E-35	-0.562
cg02914427	16	29674184	*SPN*	TSS1500	N_Shore	0.543±0.13	0.814±0.043	-0.271	4.98E-04	2.49E-45	-0.621
cg09332655	6	43044537	*PTK7*	TSS200	Island	0.412±0.173	0.682±0.059	-0.270	2.83E-03	3.71E-34	-0.551
cg08619932	19	49200058	*FUT2*	5'UTR/1st intron	Island	0.145±0.128	0.414±0.231	-0.270	9.44E-04	1.46E-07	-0.255
cg24110050	1	67217853	*TCTEX1D1*	TSS1500	N_Shore	0.38±0.211	0.649±0.154	-0.270	8.33E-03	6.14E-12	-0.330
cg20721738	22	23487104	*RAB36*	TSS1500	N_Shore	0.378±0.163	0.648±0.11	-0.269	1.65E-03	1.70E-07	-0.254
cg09735908	16	88770558	*RNF166*	TSS1500	N_Shore	0.53±0.144	0.799±0.053	-0.269	9.61E-04	5.38E-09	-0.282
cg21872037	17	6617470	*SLC13A5*	TSS1500	Island	0.453±0.114	0.722±0.072	-0.269	1.25E-04	9.43E-06	-0.216
cg08589721	1	1370722	*VWA1*	TSS200	N_Shelf	0.247±0.097	0.516±0.23	-0.269	3.21E-04	5.39E-06	-0.222
cg06230736	10	8096650	*GATA3*	TSS200	N_Shelf	0.423±0.156	0.691±0.14	-0.269	1.24E-03	6.88E-06	-0.220
cg03049782	17	41363891	*TMEM106A*	5'UTR/1st exon	Island	0.317±0.208	0.586±0.213	-0.269	9.27E-03	3.30E-43	-0.609
cg09431416	17	75137873	*SEC14L1*	5'UTR	S_Shore	0.349±0.168	0.617±0.17	-0.268	2.30E-03	3.49E-16	-0.387
cg15229275	19	46800054	*HIF3A*	TSS1500	Island	0.324±0.131	0.591±0.201	-0.268	6.26E-04	1.68E-06	-0.233
cg08864105	1	111743020	*DENND2D*	1st exon	N_Shelf	0.541±0.101	0.808±0.083	-0.268	3.07E-05	3.44E-28	-0.506
cg10740054	19	18548004	*ISYNA1*	5'UTR/1st exon	Island	0.185±0.133	0.452±0.234	-0.267	1.25E-03	3.82E-08	-0.267
cg08857729	1	67217826	*TCTEX1D1*	TSS1500	N_Shore	0.455±0.207	0.722±0.077	-0.267	7.83E-03	1.11E-12	-0.341
cg00919591	22	23524136	*BCR*	1st exon	Island	0.448±0.075	0.715±0.081	-0.267	8.32E-07	3.42E-09	-0.286
cg14695804	20	25039210	*ACSS1*	TSS1500	Island	0.141±0.118	0.408±0.226	-0.267	6.43E-04	1.14E-07	-0.258
cg26351104	1	51796558	*TTC39A*	TSS1500	Island	0.155±0.097	0.422±0.215	-0.267	2.08E-04	1.67E-14	-0.366
cg17090968	12	46663694	*SLC38A1*	TSS1500	S_Shore	0.52±0.197	0.787±0.11	-0.267	5.92E-03	3.74E-25	-0.480
cg13484546	1	16084939	*FBLIM1*	5'UTR	N_Shore	0.295±0.169	0.562±0.234	-0.266	4.02E-03	1.77E-09	-0.291
cg17655614	16	68770944	*CDH1*	TSS1500	N_Shore	0.383±0.126	0.649±0.133	-0.266	2.21E-04	4.09E-09	-0.285
cg15991309	17	80291688	*SECTM1*	5'UTR/1st exon	Island	0.4±0.162	0.666±0.178	-0.266	1.95E-03	5.17E-06	-0.222
cg02739870	5	1113320	*SLC12A7*	TSS1500	S_Shore	0.445±0.125	0.712±0.147	-0.266	2.24E-04	6.65E-08	-0.262
cg03721195	5	137610448	*GFRA3*	TSS200	S_Shore	0.4±0.121	0.666±0.071	-0.266	2.43E-04	2.90E-08	-0.269
cg09975039	9	132082957	*C9orf106*	TSS1500	Island	0.12±0.076	0.386±0.295	-0.266	1.80E-03	3.19E-15	-0.375
cg27176614	16	88717989	*CYBA*	TSS1500	N_Shelf	0.306±0.069	0.572±0.151	-0.266	2.37E-06	3.19E-27	-0.498
cg10983208	10	73848320	*SPOCK2*	5'UTR	S_Shore	0.288±0.122	0.553±0.194	-0.266	3.75E-04	2.88E-07	-0.250
cg04111789	6	41908262	*CCND3*	5'UTR	N_Shore	0.55±0.13	0.816±0.064	-0.265	4.49E-04	5.57E-18	-0.408
cg22975671	17	6926269	*BCL6B*	TSS200	Island	0.304±0.108	0.569±0.125	-0.265	5.31E-05	4.01E-08	-0.266
cg00702126	1	1369793	*VWA1*	TSS1500	N_Shore	0.458±0.105	0.724±0.057	-0.265	9.53E-05	4.46E-16	-0.386
cg10633601	1	47696701	*TAL1*	5'UTR	Island	0.375±0.184	0.64±0.174	-0.265	4.32E-03	1.28E-07	-0.257
cg17589633	1	203595283	*ATP2B4*	TSS1500	N_Shelf	0.294±0.185	0.559±0.171	-0.265	4.51E-03	2.05E-11	-0.322
cg23258881	1	150123037	*PLEKHO1*	5'UTR/1st exon	Island	0.408±0.041	0.673±0.081	-0.265	8.80E-11	3.51E-10	-0.303
cg26078793	14	65006222	*HSPA2*	TSS1500	N_Shore	0.239±0.11	0.503±0.179	-0.265	1.49E-04	7.48E-07	-0.241
cg22557662	19	38747374	*PPP1R14A*	TSS200	Island	0.426±0.135	0.691±0.105	-0.264	4.53E-04	4.97E-07	-0.245
cg12630147	10	70321574	*TET1*	5'UTR/1st intron	S_Shore	0.386±0.176	0.65±0.204	-0.264	4.09E-03	5.06E-10	-0.300
cg27173151	1	203598330	*ATP2B4*	5'UTR/1st intron	N_Shore	0.438±0.103	0.703±0.065	-0.264	6.55E-05	2.37E-11	-0.321
cg12005186	11	63973952	*FERMT3*	TSS200	N_Shore	0.444±0.1	0.708±0.064	-0.264	5.24E-05	5.41E-37	-0.571
cg21138405	5	131827807	*IRF1*	TSS1500	S_Shore	0.528±0.136	0.792±0.051	-0.264	7.26E-04	1.98E-18	-0.413
cg21620540	11	12031314	*DKK3*	TSS1500	S_Shore	0.454±0.093	0.718±0.128	-0.264	1.17E-05	4.53E-11	-0.317
cg05384271	19	6464029	*CRB3*	5'UTR/1st exon	S_Shelf	0.521±0.174	0.785±0.047	-0.264	3.41E-03	3.75E-06	-0.226
cg26325723	20	25039792	*ACSS1*	TSS1500	S_Shore	0.389±0.148	0.653±0.131	-0.264	9.53E-04	2.65E-08	-0.270
cg23108728	2	220117875	*TUBA4A*	5'UTR/1st intron	N_Shore	0.125±0.085	0.389±0.247	-0.264	5.25E-04	2.06E-22	-0.455
cg22079161	4	7940579	*AFAP1*	5'UTR/1st intron	Island	0.426±0.167	0.689±0.11	-0.264	2.28E-03	6.02E-16	-0.384
cg15565032	15	68871412	*CORO2B*	5'UTR	Island	0.392±0.192	0.655±0.17	-0.263	5.79E-03	2.10E-05	-0.208
cg27391934	1	1369948	*VWA1*	TSS1500	N_Shore	0.483±0.078	0.746±0.064	-0.263	3.08E-06	7.79E-14	-0.357
cg26421140	14	65006053	*HSPA2*	TSS1500	N_Shore	0.439±0.15	0.701±0.152	-0.263	1.14E-03	1.86E-05	-0.209
cg07740579	17	76124173	*TMC6*	5'UTR/1st intron	N_Shelf	0.179±0.149	0.442±0.2	-0.263	1.58E-03	9.63E-13	-0.342
cg16875629	19	36389575	*NFKBID*	5'UTR	N_Shore	0.488±0.083	0.75±0.072	-0.263	5.66E-06	1.88E-15	-0.378
cg05191006	16	69760966	*NQO1*	TSS1500	S_Shore	0.124±0.109	0.387±0.247	-0.263	9.72E-04	1.11E-20	-0.437

cg20961591	19	38755588	*SPINT2*	1st exon	Island	0.3±0.142	0.562±0.123	-0.263	7.23E-04	7.76E-36	-0.563
cg01062691	16	11763141	*SNN*	5'UTR/1st intron	Island	0.203±0.134	0.465±0.197	-0.262	8.05E-04	1.24E-09	-0.294
cg06616710	10	99473224	*MARVELD1*	TSS1500	Island	0.419±0.058	0.681±0.066	-0.262	3.58E-08	4.14E-36	-0.565
cg00569673	6	32160462	*GPSM3*	5'UTR	N_Shelf	0.422±0.114	0.684±0.078	-0.262	1.43E-04	1.78E-32	-0.539
cg09354037	6	32813531	*PSMB8*	TSS1500	S_Shore	0.531±0.13	0.792±0.064	-0.262	5.21E-04	1.84E-11	-0.323
cg09445448	9	134151854	*FAM78A*	5'UTR/1st exon	Island	0.327±0.055	0.588±0.178	-0.261	1.01E-05	5.58E-07	-0.244
cg27062617	16	67281337	*FHOD1*	5'UTR/1st exon	Island	0.144±0.142	0.405±0.222	-0.261	1.69E-03	1.55E-05	-0.211
cg22775000	9	103235181	*TMEFF1*	TSS1500	Island	0.402±0.209	0.663±0.158	-0.261	9.42E-03	5.60E-09	-0.282
cg23859635	2	42795262	*MTA3*	5'UTR	N_Shore	0.541±0.138	0.802±0.084	-0.261	6.80E-04	1.13E-28	-0.510
cg02951552	1	156051441	*MEX3A*	1st exon	N_Shore	0.351±0.137	0.612±0.174	-0.261	7.55E-04	1.58E-26	-0.492
cg04118306	8	24772350	*NEFM*	1st exon	Island	0.363±0.142	0.623±0.121	-0.261	7.37E-04	9.17E-06	-0.217
cg01414934	1	26605969	*SH3BGRL3*	TSS1500	N_Shore	0.397±0.118	0.657±0.081	-0.260	1.99E-04	5.43E-20	-0.430
cg00731785	4	15704680	*BST1*	5'UTR/1st exon	Island	0.347±0.196	0.607±0.148	-0.260	6.65E-03	3.09E-30	-0.522
cg13501527	9	123640474	*PHF19*	TSS1500	S_Shore	0.515±0.14	0.775±0.14	-0.260	6.92E-04	2.38E-16	-0.389
cg19858017	1	9884719	*CLSTN1*	TSS200	S_Shore	0.243±0.112	0.503±0.221	-0.260	5.97E-04	2.11E-08	-0.272
cg26595520	7	127671033	*LRRC4*	TSS200	N_Shore	0.478±0.045	0.737±0.054	-0.260	6.31E-10	6.95E-20	-0.429
cg12760508	6	111200500	*AMD1*	5'UTR/1st intron	S_Shelf	0.436±0.183	0.696±0.229	-0.260	6.81E-03	1.32E-12	-0.340
cg02457623	1	203599089	*ATP2B4*	5'UTR/1st intron	S_Shore	0.414±0.107	0.674±0.1	-0.260	6.75E-05	1.91E-07	-0.253
cg08802990	12	32260376	*BICD1*	1st exon	S_Shore	0.164±0.127	0.423±0.216	-0.259	9.41E-04	6.91E-20	-0.429
cg04292549	19	39108723	*MAP4K1*	TSS200	N_Shore	0.334±0.075	0.593±0.177	-0.259	2.21E-05	2.57E-14	-0.364
cg08302480	19	54369133	*MYADM*	5'UTR/1st intron	N_Shelf	0.503±0.047	0.762±0.051	-0.259	2.87E-09	4.94E-10	-0.300
cg13424870	17	73083812	*SLC16A5*	TSS1500	N_Shore	0.347±0.041	0.606±0.117	-0.259	1.98E-08	1.31E-17	-0.404
cg16469046	2	99062999	*INPP4A*	5'UTR/1st intron	S_Shore	0.588±0.18	0.847±0.05	-0.259	4.44E-03	1.20E-16	-0.393
cg01720186	3	182970846	*B3GNT5*	TSS200	N_Shore	0.199±0.132	0.458±0.261	-0.259	2.70E-03	1.39E-16	-0.392
cg04184297	17	80186266	*SLC16A3*	TSS1500	Island	0.451±0.17	0.71±0.091	-0.259	2.94E-03	1.01E-09	-0.295
cg06782692	10	134121419	*STK32C*	5'UTR/1st exon	Island	0.138±0.123	0.397±0.216	-0.259	8.03E-04	3.55E-07	-0.248
cg25301767	12	49731393	*C1QL4*	TSS1500	S_Shore	0.546±0.099	0.805±0.064	-0.259	5.72E-05	2.11E-05	-0.208
cg23297477	16	66638293	*CMTM3*	5'UTR/1st exon	Island	0.283±0.18	0.542±0.243	-0.259	7.37E-03	1.03E-09	-0.295
cg04482110	17	41364121	*TMEM106A*	5'UTR/1st intron	Island	0.367±0.19	0.625±0.184	-0.258	6.48E-03	2.70E-45	-0.621
cg03573679	7	2445727	*CHST12*	5'UTR/1st intron	S_Shore	0.471±0.107	0.729±0.108	-0.258	6.77E-05	7.78E-11	-0.313
cg21642245	3	195623347	*TNK2*	5'UTR/1st intron	S_Shore	0.512±0.1	0.77±0.048	-0.258	8.61E-05	6.81E-07	-0.242
cg11543397	8	120220094	*MAL2*	TSS1500	N_Shore	0.351±0.164	0.609±0.199	-0.258	3.14E-03	8.96E-06	-0.217
cg12614029	11	2160564	*IGF2*	5'UTR	Island	0.447±0.221	0.705±0.185	-0.258	1.43E-02	4.99E-14	-0.360
cg21962901	19	18549134	*ISYNA1*	TSS1500	Island	0.352±0.151	0.609±0.086	-0.258	1.43E-03	8.24E-18	-0.406
cg06665109	19	54369436	*MYADM*	5'UTR/1st exon	N_Shelf	0.59±0.123	0.848±0.047	-0.258	4.37E-04	8.27E-13	-0.343
cg14973995	4	2935692	*MFSD10*	5'UTR/1st exon	S_Shore	0.266±0.15	0.523±0.183	-0.258	1.64E-03	1.02E-11	-0.327
cg17300047	13	49108353	*RCBTB2*	TSS1500	S_Shore	0.534±0.131	0.791±0.082	-0.257	5.09E-04	6.40E-06	-0.220
cg02561912	20	50158202	*NFATC2*	5'UTR/1st intron	N_Shore	0.084±0.047	0.341±0.178	-0.257	1.08E-05	4.05E-08	-0.266
cg02756056	6	32813582	*PSMB8*	TSS1500	S_Shore	0.464±0.132	0.721±0.082	-0.257	5.40E-04	8.96E-22	-0.448
cg17795059	22	45704902	*FAM118A*	1st exon	N_Shore	0.464±0.122	0.721±0.055	-0.257	4.09E-04	8.23E-07	-0.240
cg12624792	22	38714001	*CSNK1E*	5'UTR/1st exon	S_Shore	0.307±0.126	0.564±0.15	-0.257	3.58E-04	3.24E-11	-0.319
cg19403104	19	50031246	*RCN3*	5'UTR/1st exon	S_Shelf	0.489±0.113	0.746±0.065	-0.257	1.95E-04	7.93E-09	-0.279
cg27453857	17	4689640	*VMO1*	1st exon	S_Shelf	0.534±0.111	0.79±0.051	-0.257	2.03E-04	1.02E-05	-0.215
cg17787134	17	47073522	*IGF2BP1*	TSS1500	Island	0.198±0.116	0.455±0.221	-0.257	7.60E-04	3.47E-09	-0.286
cg00537121	11	67055633	*ANKRD13D*	TSS1500	N_Shore	0.432±0.104	0.689±0.077	-0.257	7.42E-05	6.39E-20	-0.429
cg23344780	19	48829487	*EMP3*	5'UTR/1st intron	N_Shelf	0.319±0.174	0.575±0.123	-0.256	3.51E-03	1.94E-11	-0.323
cg06334134	7	142986693	*CASP2*	5'UTR/1st exon	S_Shore	0.199±0.154	0.455±0.222	-0.256	3.07E-03	7.26E-09	-0.280
cg03085719	11	3860569	*RHOG*	5'UTR/1st intron	N_Shore	0.451±0.111	0.707±0.066	-0.256	1.70E-04	2.48E-23	-0.463
cg09115984	16	31214426	*PYCARD*	TSS1500	S_Shore	0.293±0.085	0.549±0.184	-0.256	6.09E-05	3.36E-13	-0.348
cg22029587	2	233925001	*INPP5D*	5'UTR/1st exon	N_Shore	0.276±0.164	0.532±0.192	-0.256	3.13E-03	1.89E-08	-0.273
cg15083233	9	112810402	*AKAP2*	TSS1500	N_Shore	0.562±0.115	0.817±0.045	-0.256	2.96E-04	2.76E-08	-0.269
cg08347042	19	45579378	*ZNF296*	1st exon	N_Shelf	0.121±0.16	0.377±0.199	-0.256	2.88E-03	1.57E-07	-0.255
cg12253437	6	32813448	*PSMB8*	TSS1500	S_Shore	0.476±0.129	0.731±0.062	-0.256	5.71E-04	4.08E-11	-0.318
cg03585419	19	54369681	*MYADM*	5'UTR/1st intron	N_Shelf	0.399±0.126	0.655±0.176	-0.256	4.99E-04	2.39E-10	-0.305
cg16519300	1	16084830	*FBLIM1*	5'UTR	N_Shore	0.4±0.15	0.655±0.124	-0.256	1.31E-03	1.30E-17	-0.404
cg12666727	1	42128487	*HIVEP3*	5'UTR	Island	0.394±0.247	0.649±0.211	-0.256	2.60E-02	1.41E-07	-0.256
cg13391638	7	112430772	*TMEM168*	TSS1500	S_Shore	0.342±0.181	0.597±0.199	-0.256	5.88E-03	1.59E-11	-0.324
cg10865498	20	35975022	*SRC*	5'UTR/1st intron	S_Shore	0.552±0.168	0.807±0.052	-0.255	3.29E-03	7.64E-46	-0.624
cg21225170	5	137610391	*GFRA3*	TSS200	S_Shore	0.493±0.126	0.748±0.067	-0.255	4.52E-04	4.40E-17	-0.398
cg21504064	17	41363502	*TMEM106A*	TSS1500	N_Shore	0.305±0.221	0.56±0.209	-0.255	1.62E-02	1.60E-36	-0.568
cg19099850	14	55595958	*LGALS3*	5'UTR/1st exon	Island	0.295±0.26	0.551±0.271	-0.255	3.86E-02	3.89E-11	-0.318
cg19136673	6	32813337	*PSMB8*	TSS1500	S_Shore	0.482±0.121	0.737±0.065	-0.255	3.44E-04	1.86E-12	-0.338
cg21026022	18	21719566	*CABYR*	5'UTR/1st intron	S_Shore	0.39±0.213	0.645±0.271	-0.255	1.95E-02	4.20E-08	-0.266
cg26403843	5	158634085	*RNF145*	5'UTR/1st intron	N_Shelf	0.43±0.151	0.685±0.193	-0.255	2.01E-03	3.98E-28	-0.506
cg16771783	10	82116392	*DYDC1*	5'UTR/1st intron	Island	0.387±0.104	0.641±0.135	-0.255	6.06E-05	1.60E-13	-0.353
cg04879832	16	88717456	*CYBA*	5'UTR/1st exon	Island	0.497±0.165	0.751±0.048	-0.254	3.13E-03	7.84E-38	-0.576
cg02227453	22	50919482	*ADM2*	TSS1500	Island	0.502±0.101	0.756±0.112	-0.254	4.13E-05	2.82E-07	-0.250
cg19663795	16	30393854	*SEPT1*	1st exon	S_Shelf	0.528±0.124	0.782±0.051	-0.254	4.95E-04	6.69E-37	-0.570
cg13167664	11	2158987	*IGF2*	5'UTR	Island	0.168±0.13	0.422±0.252	-0.254	2.53E-03	8.45E-14	-0.357
cg06367659	22	50919817	*ADM2*	TSS1500	Island	0.186±0.186	0.44±0.242	-0.254	9.53E-03	1.36E-12	-0.340
cg03946324	10	14995581	*DCLRE1C*	5'UTR/1st intron	N_Shore	0.147±0.15	0.401±0.279	-0.254	6.42E-03	4.33E-33	-0.544
cg17332573	6	26250934	*HIST1H2BH*	TSS1500	S_Shelf	0.47±0.138	0.724±0.139	-0.253	7.53E-04	4.09E-06	-0.225
cg09418984	19	54369447	*MYADM*	5'UTR/1st exon	N_Shelf	0.612±0.123	0.865±0.035	-0.253	5.53E-04	1.46E-11	-0.325
cg25450266	19	35494294	*GRAMD1A*	5'UTR/1st intron	S_Shelf	0.473±0.124	0.725±0.094	-0.253	3.46E-04	5.20E-25	-0.479
cg24641737	1	111743271	*DENND2D*	5'UTR/1st exon	N_Shelf	0.435±0.11	0.687±0.136	-0.252	1.14E-04	1.19E-30	-0.526
cg02553663	17	80291775	*SECTM1*	5'UTR/1st exon	Island	0.466±0.189	0.718±0.163	-0.252	6.73E-03	7.01E-06	-0.219
cg05347898	6	24358304	*DCDC2*	5'UTR/1st intron	N_Shore	0.322±0.226	0.574±0.245	-0.252	2.21E-02	3.35E-06	-0.227
cg05306745	17	76921845	*TIMP2*	TSS1500	Island	0.46±0.219	0.712±0.142	-0.252	1.39E-02	7.73E-12	-0.329
cg26546113	1	182810711	*DHX9*	5'UTR/1st intron	S_Shore	0.428±0.188	0.68±0.195	-0.252	7.61E-03	1.33E-08	-0.275
cg04515001	6	24358236	*DCDC2*	5'UTR/1st exon	N_Shore	0.395±0.219	0.646±0.221	-0.251	1.80E-02	2.86E-07	-0.250
cg00123104	1	9884732	*CLSTN1*	TSS200	S_Shore	0.356±0.122	0.608±0.16	-0.251	3.70E-04	5.41E-07	-0.244
cg15551881	9	123688715	*TRAF1*	5'UTR	N_Shelf	0.608±0.118	0.859±0.036	-0.251	4.51E-04	1.59E-35	-0.561
cg26165146	12	27484656	*ARNTL2*	TSS1500	N_Shore	0.512±0.1	0.763±0.141	-0.251	5.42E-05	2.62E-17	-0.401
cg03802185	7	101927846	*SH2B2*	TSS1500	N_Shore	0.537±0.119	0.789±0.05	-0.251	3.97E-04	8.24E-10	-0.297
cg26371512	7	112430779	*TMEM168*	TSS1500	S_Shore	0.305±0.181	0.556±0.186	-0.251	6.01E-03	7.35E-14	-0.357
cg02988947	17	61778813	*LIMD2*	TSS1500	S_Shore	0.498±0.122	0.749±0.05	-0.251	4.97E-04	3.43E-20	-0.432
cg26517849	11	2161141	*IGF2*	5'UTR	Island	0.45±0.088	0.701±0.086	-0.251	1.18E-05	2.54E-20	-0.434

cg11591325	5	76012591	*F2R*	5'UTR/1st intron	S_Shore	0.203±0.077	0.454±0.221	-0.251	2.79E-04	4.03E-07	-0.247
cg05995220	20	55964998	*RBM38*	TSS1500	S_Shore	0.336±0.167	0.587±0.046	-0.251	3.53E-03	6.33E-10	-0.299
cg06055013	10	116853664	*ATRNL1*	1st exon	Island	0.166±0.115	0.417±0.268	-0.250	2.81E-03	2.12E-08	-0.272
cg00676801	2	191876673	*STAT1*	5'UTR	N_Shore	0.423±0.141	0.673±0.174	-0.250	1.25E-03	2.02E-27	-0.500
cg01848457	12	104697983	*TXNRD1*	5'UTR	Island	0.392±0.188	0.642±0.193	-0.250	7.98E-03	1.79E-25	-0.483
cg01848457	12	104697983	*EID3*	1st exon	Island	0.392±0.188	0.642±0.193	-0.250	7.98E-03	5.04E-24	-0.470
cg06679087	10	99473206	*MARVELD1*	TSS1500	Island	0.61±0.077	0.86±0.041	-0.250	1.42E-05	2.45E-50	-0.648
cg17729941	1	153607617	*S100A13*	TSS1500	S_Shore	0.384±0.166	0.634±0.165	-0.250	3.46E-03	4.35E-06	-0.224
cg09877117	19	47735882	*BBC3*	5'UTR/1st exon	S_Shore	0.234±0.066	0.484±0.112	-0.250	4.46E-07	3.31E-07	-0.248
cg08291098	20	62285239	*STMN3*	TSS1500	S_Shore	0.384±0.189	0.634±0.199	-0.249	8.38E-03	2.44E-08	-0.270
cg00651401	3	195623338	*TNK2*	5'UTR/1st intron	S_Shore	0.488±0.119	0.737±0.086	-0.249	2.92E-04	9.80E-06	-0.216
cg00757933	6	29760864	*HCG4*	TSS200	Island	0.209±0.107	0.458±0.224	-0.249	7.94E-04	7.04E-09	-0.280
cg13184736	1	68299409	*GNG12*	TSS1500	S_Shore	0.392±0.184	0.64±0.155	-0.249	6.22E-03	8.13E-11	-0.313
cg02044895	11	58343318	*LPXN*	5'UTR/1st exon	N_Shelf	0.543±0.135	0.792±0.055	-0.249	9.99E-04	3.48E-41	-0.597
cg27373972	6	33244788	*B3GALT4*	TSS200	Island	0.434±0.149	0.683±0.075	-0.249	1.69E-03	3.12E-62	-0.701
cg02349096	19	11450198	*RAB3D*	5'UTR/1st exon	Island	0.383±0.152	0.632±0.09	-0.249	1.88E-03	3.41E-35	-0.559
cg26120958	11	6439806	*APBB1*	5'UTR/1st intron	N_Shore	0.412±0.113	0.661±0.056	-0.249	2.73E-04	6.00E-17	-0.396
cg26173847	7	127671017	*LRRC4*	TSS200	N_Shore	0.556±0.07	0.804±0.055	-0.248	2.32E-06	9.09E-20	-0.428
cg25338707	11	63973846	*FERMT3*	TSS1500	N_Shore	0.433±0.085	0.681±0.05	-0.248	2.74E-05	1.22E-42	-0.606
cg07818563	16	16043225	*ABCC1*	TSS1500	Island	0.192±0.14	0.44±0.197	-0.248	1.74E-03	6.63E-09	-0.281
cg08480068	16	29674959	*SPN*	5'UTR/1st intron	N_Shore	0.628±0.103	0.876±0.061	-0.248	1.21E-04	1.15E-45	-0.623
cg10483825	12	21810450	*LDHB*	5'UTR/1st exon	N_Shore	0.402±0.123	0.649±0.099	-0.248	3.56E-04	2.21E-20	-0.434
cg26888530	4	7941792	*AFAP1*	TSS200	Island	0.302±0.234	0.55±0.306	-0.248	3.72E-02	3.51E-17	-0.399
cg23066808	7	2446566	*CHST12*	5'UTR/1st intron	S_Shelf	0.492±0.108	0.739±0.083	-0.248	1.36E-04	1.04E-09	-0.295
cg04625862	3	48341880	*NME6*	5'UTR/1st intron	N_Shore	0.563±0.13	0.811±0.04	-0.248	8.70E-04	1.10E-06	-0.237
cg08231730	11	63753169	*OTUB1*	TSS200	N_Shore	0.388±0.151	0.635±0.155	-0.247	1.90E-03	2.88E-09	-0.287
cg17243289	18	45458021	*SMAD2*	TSS1500	Island	0.333±0.14	0.579±0.15	-0.247	1.11E-03	6.41E-08	-0.262
cg01086895	11	6676515	*DCHS1*	5'UTR/1st intron	Island	0.061±0.058	0.308±0.259	-0.247	1.02E-03	2.25E-05	-0.207
cg13102585	17	42386208	*RUNDC3A*	1st exon	Island	0.402±0.087	0.648±0.1	-0.247	1.19E-05	3.41E-05	-0.203
cg23264429	10	90642003	*STAMBPL1*	5'UTR/1st intron	S_Shore	0.518±0.136	0.764±0.104	-0.246	8.77E-04	1.61E-18	-0.414
cg02571816	19	38747378	*PPP1R14A*	TSS200	Island	0.517±0.114	0.763±0.063	-0.246	2.90E-04	8.25E-10	-0.297
cg00619207	1	111743368	*DENND2D*	TSS200	N_Shelf	0.411±0.107	0.656±0.138	-0.246	1.27E-04	1.70E-25	-0.483
cg00259834	3	16553499	*RFTN1*	5'UTR/1st intron	N_Shore	0.176±0.131	0.422±0.208	-0.246	1.58E-03	3.71E-49	-0.642
cg06385449	11	63974123	*FERMT3*	TSS200	N_Shore	0.505±0.116	0.75±0.048	-0.245	3.92E-04	8.83E-51	-0.650
cg22740783	2	27341458	*CGREF1*	5'UTR/1st intron	N_Shore	0.403±0.184	0.648±0.252	-0.245	1.21E-02	2.42E-24	-0.473
cg09088834	20	25565460	*NINL*	5'UTR/1st intron	Island	0.335±0.115	0.58±0.156	-0.245	2.90E-04	2.21E-11	-0.322
cg17644208	2	25142391	*ADCY3*	5'UTR/1st intron	N_Shore	0.23±0.1	0.475±0.161	-0.245	1.12E-04	2.32E-05	-0.207
cg18633684	12	104697387	*EID3*	TSS200	Island	0.419±0.187	0.664±0.184	-0.245	8.43E-03	2.01E-14	-0.365
cg19837790	10	97804137	*CCNJ*	5'UTR	Island	0.306±0.116	0.551±0.132	-0.245	2.40E-04	2.15E-19	-0.424
cg01837574	17	7836610	*CNTROB*	1st exon	S_Shelf	0.56±0.17	0.805±0.073	-0.245	4.30E-03	1.57E-05	-0.211
cg16672562	19	46801672	*HIF3A*	5'UTR/1st exon	S_Shore	0.577±0.219	0.822±0.135	-0.244	1.62E-02	7.73E-11	-0.313
cg07921503	7	2445843	*CHST12*	5'UTR/1st intron	S_Shore	0.497±0.105	0.741±0.051	-0.244	1.88E-04	3.62E-13	-0.348
cg13087076	10	82116405	*DYDC1*	5'UTR/1st intron	Island	0.39±0.108	0.634±0.148	-0.244	1.67E-04	5.67E-14	-0.359
cg04477789	16	30935822	*FBXL19*	5'UTR/1st intron	S_Shore	0.404±0.14	0.649±0.159	-0.244	1.28E-03	8.64E-17	-0.394
cg06233293	2	47596185	*EPCAM*	TSS200	N_Shore	0.162±0.126	0.407±0.245	-0.244	2.80E-03	1.19E-09	-0.294
cg16644366	16	29674903	*SPN*	5'UTR/1st intron	N_Shore	0.517±0.103	0.761±0.054	-0.244	1.58E-04	6.75E-23	-0.459
cg07086380	5	118691033	*TNFAIP8*	5'UTR	N_Shore	0.611±0.108	0.856±0.041	-0.244	2.82E-04	1.53E-24	-0.475
cg02614129	5	134733615	*H2AFY*	5'UTR/1st intron	N_Shore	0.266±0.138	0.509±0.24	-0.244	3.57E-03	6.53E-09	-0.281
cg09025501	16	88717472	*CYBA*	5'UTR/1st exon	Island	0.469±0.155	0.712±0.054	-0.243	2.72E-03	2.67E-31	-0.531
cg03431846	14	24641183	*REC8*	TSS200	Island	0.521±0.095	0.764±0.061	-0.243	6.81E-05	3.37E-12	-0.334
cg06023570	2	7058545	*RNF144A*	5'UTR/1st intron	S_Shore	0.337±0.133	0.58±0.212	-0.243	1.93E-03	1.50E-11	-0.324
cg27252395	6	24358327	*DCDC2*	5'UTR/1st intron	N_Shore	0.335±0.214	0.577±0.192	-0.243	1.73E-02	9.95E-07	-0.238
cg13177786	11	10472099	*AMPD3*	5'UTR/1st intron	Island	0.166±0.25	0.409±0.261	-0.243	4.07E-02	5.51E-09	-0.282
cg11033273	11	35966115	*LDLRAD3*	5'UTR/1st intron	Island	0.38±0.106	0.623±0.115	-0.243	1.08E-04	2.00E-09	-0.290
cg18442524	5	137610372	*GFRA3*	TSS200	S_Shore	0.561±0.124	0.804±0.047	-0.243	6.94E-04	8.41E-23	-0.458
cg09546802	5	145215546	*PRELID2*	TSS1500	S_Shore	0.493±0.132	0.735±0.092	-0.243	7.88E-04	1.06E-06	-0.238
cg26700716	2	183730940	*FRZB*	1st exon	N_Shore	0.459±0.096	0.702±0.1	-0.243	4.06E-05	8.34E-10	-0.296
cg06632214	11	314074	*IFITM1*	5'UTR/1st exon	S_Shelf	0.367±0.194	0.609±0.306	-0.243	2.39E-02	6.95E-37	-0.570
cg16415340	11	2160964	*IGF2*	5'UTR	Island	0.562±0.063	0.805±0.078	-0.243	2.11E-07	8.43E-20	-0.428
cg18534491	14	24641155	*REC8*	TSS200	Island	0.554±0.102	0.796±0.067	-0.242	1.12E-04	3.50E-09	-0.286
cg20583073	19	3178759	*S1PR4*	5'UTR/1st exon	Island	0.475±0.081	0.717±0.069	-0.242	9.62E-06	3.51E-21	-0.442
cg24432073	4	76555634	*CDKL2*	5'UTR/1st exon	Island	0.41±0.133	0.652±0.111	-0.242	7.71E-04	1.73E-06	-0.233
cg05697726	6	32812933	*PSMB8*	TSS1500	S_Shore	0.487±0.12	0.729±0.069	-0.242	4.56E-04	1.09E-16	-0.393
cg13165140	6	32813522	*PSMB8*	TSS1500	S_Shore	0.481±0.117	0.724±0.067	-0.242	3.75E-04	8.83E-14	-0.356
cg26301953	6	32164503	*GPSM3*	TSS1500	S_Shore	0.405±0.107	0.647±0.121	-0.242	1.28E-04	1.93E-08	-0.272
cg03684062	19	3179364	*S1PR4*	1st exon	Island	0.485±0.097	0.727±0.068	-0.242	7.63E-05	2.15E-27	-0.500
cg17924813	4	15704844	*BST1*	1st exon	Island	0.375±0.157	0.617±0.116	-0.242	2.63E-03	5.08E-34	-0.551
cg27573591	7	127672152	*LRRC4*	TSS1500	Island	0.416±0.087	0.658±0.079	-0.242	1.88E-05	1.65E-16	-0.391
cg14042635	17	40440916	*STAT5A*	5'UTR	Island	0.465±0.099	0.706±0.089	-0.242	6.44E-05	3.38E-20	-0.432
cg19954537	2	97204401	*ARID5A*	5'UTR/1st intron	S_Shore	0.568±0.112	0.81±0.044	-0.242	3.58E-04	1.28E-06	-0.236
cg16777510	17	40440022	*STAT5A*	5'UTR/1st exon	N_Shore	0.418±0.114	0.659±0.1	-0.242	2.25E-04	4.99E-20	-0.431
cg14565781	1	45274513	*BTBD19*	1st exon	S_Shelf	0.487±0.159	0.729±0.121	-0.241	2.86E-03	6.22E-13	-0.345
cg03752203	8	103874399	*AZIN1*	5'UTR/1st intron	N_Shore	0.525±0.187	0.766±0.2	-0.241	9.85E-03	9.89E-12	-0.327
cg24819835	4	15780011	*CD38*	1st exon	Island	0.407±0.145	0.648±0.095	-0.241	1.61E-03	4.42E-13	-0.347
cg13816423	6	41513166	*FOXP4*	TSS1500	N_Shore	0.456±0.128	0.697±0.163	-0.241	7.88E-04	2.04E-08	-0.272
cg09154880	6	32813715	*PSMB8*	TSS1500	S_Shore	0.613±0.152	0.854±0.053	-0.241	2.59E-03	4.86E-16	-0.385
cg21549285	21	42799141	*MX1*	5'UTR	S_Shore	0.424±0.13	0.664±0.182	-0.241	1.16E-03	3.09E-15	-0.375
cg08146256	14	21734410	*HNRNPC*	5'UTR/1st intron	N_Shelf	0.464±0.168	0.705±0.188	-0.241	5.43E-03	1.98E-12	-0.337
cg14951497	2	191875807	*STAT1*	5'UTR	N_Shelf	0.507±0.11	0.747±0.158	-0.241	2.60E-04	1.02E-25	-0.485
cg06379589	16	88767124	*RNF166*	5'UTR	S_Shelf	0.47±0.121	0.711±0.062	-0.241	5.52E-04	4.00E-07	-0.247
cg06472476	19	54369471	*MYADM*	5'UTR/1st exon	N_Shelf	0.423±0.117	0.664±0.065	-0.241	3.99E-04	9.10E-13	-0.342
cg17733616	17	73083887	*SLC16A5*	5'UTR/1st exon	Island	0.484±0.075	0.725±0.063	-0.241	4.76E-06	8.72E-24	-0.468
cg12580445	5	121414634	*LOX*	TSS1500	S_Shore	0.387±0.13	0.628±0.191	-0.241	1.35E-03	1.01E-05	-0.216
cg19000647	2	11885321	*LPIN1*	TSS1500	N_Shore	0.525±0.162	0.765±0.095	-0.240	3.35E-03	3.62E-05	-0.202
cg26010734	19	15344046	*EPHX3*	TSS1500	N_Shore	0.466±0.152	0.707±0.133	-0.240	2.29E-03	3.60E-09	-0.286
cg06357561	17	40439501	*STAT5A*	TSS1500	N_Shore	0.49±0.041	0.73±0.092	-0.240	2.52E-09	2.07E-32	-0.539
cg21397588	1	203598954	*ATP2B4*	5'UTR/1st intron	S_Shore	0.47±0.111	0.71±0.055	-0.240	2.99E-04	5.56E-07	-0.244

cg08843517	16	88717464	*CYBA*	5'UTR/1st exon	Island	0.603±0.172	0.842±0.034	-0.240	5.45E-03	8.85E-36	-0.563
cg19808978	19	50031273	*RCN3*	5'UTR/1st exon	S_Shelf	0.456±0.099	0.696±0.071	-0.240	9.38E-05	9.89E-09	-0.278
cg21152671	9	123690586	*TRAF1*	5'UTR/1st intron	N_Shore	0.352±0.108	0.592±0.191	-0.240	5.06E-04	1.31E-05	-0.213
cg26588045	19	41283090	*RAB4B*	TSS1500	N_Shore	0.348±0.094	0.587±0.16	-0.240	9.60E-05	1.74E-06	-0.233
cg26614816	12	104697532	*EID3*	5'UTR/1st exon	Island	0.426±0.221	0.665±0.182	-0.240	2.04E-02	5.01E-15	-0.373
cg07912766	18	45458698	*SMAD2*	TSS1500	S_Shore	0.559±0.238	0.798±0.112	-0.239	2.54E-02	2.87E-08	-0.269
cg20690774	11	45202557	*PRDM11*	5'UTR/1st intron	Island	0.265±0.188	0.504±0.194	-0.239	1.02E-02	3.09E-10	-0.304
cg15774391	17	40439628	*STAT5A*	5'UTR/1st exon	N_Shore	0.363±0.075	0.602±0.117	-0.239	3.87E-06	8.31E-30	-0.519
cg11102782	19	18549136	*ISYNA1*	TSS1500	Island	0.447±0.144	0.686±0.089	-0.239	1.66E-03	1.09E-20	-0.437
cg24255928	10	97802966	*CCNJ*	TSS200	Island	0.43±0.268	0.669±0.146	-0.239	4.21E-02	5.13E-29	-0.513
cg27121758	19	3178742	*S1PR4*	5'UTR/1st exon	Island	0.483±0.11	0.722±0.051	-0.239	3.05E-04	1.35E-29	-0.517
cg10729426	19	58038585	*ZNF549*	TSS200	Island	0.334±0.26	0.573±0.246	-0.239	4.68E-02	2.32E-53	-0.662
cg18645906	4	15704599	*BST1*	5'UTR/1st exon	N_Shore	0.487±0.167	0.726±0.125	-0.239	4.29E-03	5.06E-30	-0.521
cg26620176	19	8117047	*CCL25*	TSS1500	S_Shore	0.555±0.121	0.794±0.086	-0.239	4.62E-04	2.44E-06	-0.230
cg16686733	20	25566563	*NINL*	TSS1500	S_Shore	0.225±0.129	0.463±0.237	-0.238	3.16E-03	1.85E-07	-0.253
cg19863210	8	142139002	*DENND3*	5'UTR/1st intron	Island	0.237±0.196	0.475±0.226	-0.238	1.54E-02	1.14E-05	-0.214
cg09863247	20	44539966	*PLTP*	TSS1500	N_Shore	0.434±0.095	0.672±0.109	-0.238	4.40E-05	8.32E-13	-0.343
cg02654940	6	34206400	*HMGA1*	5'UTR	S_Shore	0.56±0.116	0.798±0.065	-0.238	4.17E-04	7.68E-30	-0.519
cg01059100	16	31214607	*PYCARD*	TSS1500	S_Shore	0.325±0.075	0.563±0.151	-0.238	1.73E-05	4.00E-19	-0.421
cg03776194	16	88770966	*RNF166*	TSS1500	N_Shore	0.591±0.122	0.829±0.057	-0.238	6.39E-04	2.62E-14	-0.363
cg25767433	6	33386985	*SYNGAP1*	TSS1500	S_Shore	0.574±0.115	0.811±0.059	-0.238	4.11E-04	2.74E-14	-0.363
cg09091424	11	67057304	*ANKRD13D*	5'UTR	S_Shore	0.262±0.117	0.499±0.153	-0.237	4.24E-04	1.67E-37	-0.574
cg07516252	14	24641201	*REC8*	TSS200	Island	0.476±0.095	0.713±0.059	-0.237	8.23E-05	6.93E-13	-0.344
cg02780269	17	80292252	*SECTM1*	TSS1500	Island	0.573±0.131	0.81±0.035	-0.237	1.26E-03	1.92E-23	-0.465
cg19808205	1	67217984	*TCTEX1D1*	TSS200	N_Shore	0.452±0.177	0.689±0.15	-0.236	6.62E-03	2.11E-14	-0.365
cg13380204	17	40441155	*STAT5A*	5'UTR	S_Shore	0.552±0.099	0.788±0.072	-0.236	9.78E-05	2.78E-18	-0.412
cg06889086	7	55638230	*VOPP1*	5'UTR/1st intron	N_Shore	0.506±0.119	0.742±0.102	-0.236	4.02E-04	1.11E-21	-0.447
cg03559682	11	6439864	*APBB1*	5'UTR/1st intron	Island	0.433±0.149	0.669±0.122	-0.236	2.18E-03	9.48E-11	-0.312
cg27306119	2	9144605	*MBOAT2*	TSS1500	Island	0.331±0.181	0.567±0.16	-0.236	7.93E-03	1.67E-10	-0.308
cg20125091	1	92952641	*GFI1*	TSS1500	S_Shore	0.387±0.179	0.623±0.187	-0.236	8.50E-03	1.25E-06	-0.236
cg18426551	17	42147617	*G6PC3*	TSS1500	N_Shore	0.471±0.048	0.707±0.083	-0.236	7.09E-09	6.56E-07	-0.242
cg07462448	10	115441840	*CASP7*	5'UTR/1st intron	S_Shore	0.594±0.137	0.83±0.044	-0.236	1.62E-03	2.17E-05	-0.208
cg04517263	9	123689193	*TRAF1*	5'UTR/1st intron	N_Shore	0.642±0.121	0.878±0.041	-0.236	7.58E-04	1.60E-27	-0.501
cg01321189	12	32259338	*BICD1*	TSS1500	Island	0.193±0.104	0.429±0.245	-0.236	2.15E-03	1.29E-10	-0.310
cg01647936	11	63974772	*FERMT3*	5'UTR/1st intron	N_Shore	0.365±0.096	0.6±0.054	-0.236	1.09E-04	2.61E-37	-0.573
cg00284173	2	24273093	*FKBP1B*	5'UTR/1st intron	Island	0.132±0.119	0.368±0.212	-0.235	1.59E-03	1.13E-09	-0.294
cg20585869	8	24772333	*NEFM*	1st exon	Island	0.355±0.133	0.59±0.098	-0.235	1.01E-03	4.42E-06	-0.224
cg01657207	3	44690019	*ZNF35*	TSS1500	N_Shore	0.415±0.164	0.65±0.204	-0.235	6.29E-03	5.66E-19	-0.419
cg02134976	6	42110740	*C6orf132*	TSS200	S_Shore	0.38±0.181	0.615±0.186	-0.235	9.07E-03	1.56E-18	-0.414
cg11706729	6	32813154	*PSMB8*	TSS1500	S_Shore	0.36±0.124	0.595±0.121	-0.235	5.94E-04	4.79E-25	-0.479
cg21210090	4	15704429	*BST1*	TSS200	N_Shore	0.407±0.148	0.642±0.141	-0.235	2.34E-03	1.46E-36	-0.568
cg14417329	17	80186273	*SLC16A3*	TSS1500	Island	0.453±0.157	0.687±0.082	-0.234	3.34E-03	4.11E-08	-0.266
cg00255919	5	131827918	*IRF1*	TSS1500	S_Shore	0.505±0.128	0.739±0.078	-0.234	8.58E-04	1.34E-23	-0.466
cg08461586	10	97802977	*CCNJ*	TSS200	Island	0.318±0.155	0.552±0.121	-0.234	3.06E-03	7.37E-31	-0.527
cg24765602	14	65006071	*HSPA2*	TSS1500	N_Shore	0.399±0.137	0.633±0.164	-0.234	1.67E-03	1.92E-05	-0.209
cg02657012	6	32812965	*PSMB8*	TSS1500	S_Shore	0.546±0.122	0.78±0.064	-0.234	6.79E-04	3.21E-16	-0.388
cg07682037	11	63974153	*FERMT3*	5'UTR/1st exon	N_Shore	0.474±0.118	0.708±0.047	-0.234	6.29E-04	1.44E-48	-0.639
cg25384897	6	32813544	*PSMB8*	TSS1500	S_Shore	0.511±0.142	0.745±0.063	-0.234	1.91E-03	1.61E-14	-0.366
cg02346790	3	122296409	*PARP15*	TSS200	N_Shore	0.503±0.181	0.737±0.179	-0.234	9.09E-03	2.87E-27	-0.499
cg10402417	11	67171476	*TBC1D10C*	1st exon	S_Shelf	0.496±0.113	0.73±0.06	-0.234	3.87E-04	1.35E-24	-0.475
cg26769927	16	29674972	*SPN*	5'UTR/1st intron	N_Shore	0.642±0.109	0.876±0.056	-0.234	3.11E-04	1.35E-44	-0.617
cg10421029	16	30936028	*FBXL19*	5'UTR/1st exon	S_Shore	0.446±0.102	0.68±0.131	-0.234	1.20E-04	3.43E-13	-0.348
cg02931058	2	239047245	*KLHL30*	TSS200	N_Shelf	0.55±0.078	0.783±0.095	-0.234	5.57E-06	1.73E-10	-0.308
cg15694715	11	118402280	*TMEM25*	5'UTR/1st intron	S_Shore	0.526±0.2	0.76±0.181	-0.234	1.47E-02	1.89E-23	-0.465
cg01286655	11	12031099	*DKK3*	TSS1500	S_Shore	0.483±0.146	0.717±0.131	-0.233	2.11E-03	8.29E-10	-0.297
cg21195185	4	76555782	*CDKL2*	TSS200	Island	0.455±0.14	0.688±0.119	-0.233	1.52E-03	9.55E-06	-0.216
cg16396284	6	33245537	*B3GALT4*	1st exon	Island	0.542±0.107	0.775±0.054	-0.233	2.84E-04	3.90E-20	-0.432
cg09293391	6	144386418	*PLAGL1*	TSS1500	S_Shore	0.544±0.059	0.777±0.092	-0.233	1.81E-07	2.94E-05	-0.204
cg25139493	1	39957400	*BMP8A*	5'UTR/1st exon	Island	0.429±0.097	0.661±0.168	-0.233	2.00E-04	9.38E-07	-0.239
cg08755490	11	65554678	*OVOL1*	5'UTR/1st exon	Island	0.151±0.171	0.384±0.185	-0.232	7.20E-03	1.00E-05	-0.216
cg19564367	4	7941852	*AFAP1*	TSS200	Island	0.409±0.142	0.641±0.23	-0.232	4.87E-03	5.99E-19	-0.419
cg16425038	14	24641194	*REC8*	TSS200	Island	0.623±0.095	0.855±0.06	-0.232	9.39E-05	4.71E-11	-0.317
cg12060422	10	102821684	*KAZALD1*	1st exon	Island	0.441±0.226	0.673±0.277	-0.232	3.87E-02	1.01E-47	-0.634
cg09216670	10	22999381	*PIP4K2A*	5'UTR/1st intron	N_Shelf	0.531±0.143	0.763±0.047	-0.232	2.26E-03	8.99E-17	-0.394
cg19056772	17	7341616	*FGF11*	1st exon	N_Shore	0.525±0.082	0.756±0.048	-0.232	3.57E-05	2.86E-16	-0.388
cg12091732	10	82116446	*DYDC1*	5'UTR/1st intron	Island	0.395±0.118	0.626±0.172	-0.232	7.67E-04	1.59E-10	-0.308
cg19337852	19	38755293	*SPINT2*	5'UTR/1st exon	Island	0.408±0.2	0.639±0.128	-0.232	1.35E-02	4.52E-25	-0.479
cg23683674	11	73020562	*ARHGEF17*	1st exon	Island	0.288±0.173	0.52±0.18	-0.231	7.64E-03	3.95E-10	-0.302
cg26477549	12	106642937	*CKAP4*	TSS1500	S_Shore	0.298±0.123	0.529±0.232	-0.231	3.01E-03	9.03E-15	-0.369
cg11857033	17	29297552	*RNF135*	TSS1500	N_Shore	0.475±0.122	0.706±0.108	-0.231	5.87E-04	5.39E-30	-0.521
cg06479653	18	10525737	*NAPG*	TSS200	N_Shore	0.156±0.065	0.386±0.223	-0.231	5.54E-04	2.18E-07	-0.252
cg22821981	3	122296435	*PARP15*	TSS200	N_Shore	0.445±0.191	0.676±0.19	-0.231	1.33E-02	2.45E-20	-0.434
cg25636933	4	56816405	*CEP135*	5'UTR/1st intron	S_Shore	0.519±0.103	0.749±0.169	-0.231	3.25E-04	1.00E-09	-0.295
cg23369564	1	150123764	*PLEKHO1*	5'UTR	S_Shore	0.539±0.111	0.77±0.046	-0.231	4.60E-04	5.47E-41	-0.596
cg17149644	11	126225276	*ST3GAL4*	TSS1500	N_Shore	0.11±0.07	0.34±0.255	-0.230	1.83E-03	8.26E-06	-0.218
cg17037101	11	2161314	*IGF2*	5'UTR	Island	0.539±0.048	0.769±0.07	-0.230	7.24E-09	2.72E-16	-0.388
cg25563256	17	7341641	*FGF11*	1st exon	N_Shore	0.516±0.078	0.747±0.081	-0.230	7.89E-06	5.37E-14	-0.359
cg13860281	7	55638724	*VOPP1*	5'UTR/1st intron	N_Shore	0.353±0.109	0.583±0.138	-0.230	2.87E-04	5.87E-20	-0.430
cg21941030	19	50031017	*RCN3*	5'UTR/1st exon	S_Shelf	0.593±0.109	0.823±0.037	-0.230	4.46E-04	1.86E-11	-0.323
cg09302355	11	63974131	*FERMT3*	TSS200	N_Shore	0.468±0.103	0.698±0.052	-0.230	2.44E-04	1.69E-51	-0.653
cg20317872	1	111743202	*DENND2D*	5'UTR/1st exon	N_Shelf	0.596±0.101	0.826±0.059	-0.230	1.92E-04	1.53E-35	-0.561
cg12222132	4	78978705	*FRAS1*	TSS200	N_Shore	0.171±0.101	0.4±0.211	-0.229	1.03E-03	6.09E-08	-0.263
cg01322214	7	25219198	*C7orf31*	5'UTR/1st intron	N_Shore	0.57±0.158	0.799±0.097	-0.229	3.95E-03	8.74E-22	-0.449
cg21410080	21	43639862	*ABCG1*	5'UTR	Island	0.31±0.194	0.539±0.242	-0.229	2.02E-02	7.26E-07	-0.241
cg21097090	5	118693764	*TNFAIP8*	5'UTR	S_Shelf	0.634±0.127	0.863±0.038	-0.229	1.24E-03	2.20E-16	-0.390
cg26596719	7	142986429	*CASP2*	TSS1500	S_Shore	0.483±0.146	0.712±0.12	-0.229	2.38E-03	5.92E-12	-0.330
cg08599448	19	11689614	*ACP5*	5'UTR/1st intron	Island	0.22±0.128	0.449±0.206	-0.229	2.44E-03	9.51E-18	-0.406

cg00753478	12	21810489	*LDHB*	5'UTR/1st exon	Island	0.394±0.148	0.622±0.189	-0.229	3.93E-03	4.59E-12	-0.332
cg01667702	17	7836604	*CNTROB*	1st exon	S_Shelf	0.453±0.185	0.682±0.147	-0.229	1.02E-02	9.10E-06	-0.217
cg00252032	6	43043815	*PTK7*	TSS1500	N_Shore	0.347±0.108	0.575±0.165	-0.229	4.34E-04	1.02E-19	-0.427
cg24152605	19	57050359	*ZFP28*	5'UTR/1st exon	Island	0.411±0.241	0.639±0.259	-0.229	4.64E-02	5.48E-36	-0.564
cg12640109	11	62104720	*ASRGL1*	TSS200	N_Shore	0.332±0.125	0.56±0.178	-0.228	1.35E-03	6.99E-17	-0.396
cg04949489	12	21810092	*LDHB*	5'UTR/1st intron	N_Shore	0.282±0.174	0.51±0.162	-0.228	7.87E-03	6.37E-19	-0.419
cg04262471	6	33245585	*B3GALT4*	1st exon	S_Shore	0.429±0.119	0.658±0.116	-0.228	5.37E-04	1.01E-15	-0.382
cg14188639	11	2161544	*IGF2*	5'UTR	Island	0.587±0.106	0.815±0.067	-0.228	2.60E-04	1.61E-14	-0.366
cg13462576	15	74726520	*SEMA7A*	TSS1500	S_Shore	0.322±0.139	0.55±0.115	-0.228	1.74E-03	6.41E-11	-0.315
cg25323057	10	88730481	*AGAP11*	TSS200	S_Shore	0.428±0.106	0.656±0.162	-0.228	3.72E-04	1.06E-09	-0.295
cg16341159	17	7121370	*DLG4*	TSS1500	S_Shelf	0.311±0.094	0.539±0.1	-0.228	5.77E-05	4.28E-12	-0.333
cg20532370	3	139258730	*RBP1*	TSS1500	Island	0.276±0.19	0.504±0.213	-0.228	1.60E-02	9.84E-09	-0.278
cg14654468	17	80186336	*SLC16A3*	5'UTR/1st exon	Island	0.462±0.176	0.69±0.126	-0.228	7.70E-03	2.58E-06	-0.229
cg13194425	17	7341936	*FGF11*	TSS1500	N_Shore	0.564±0.127	0.792±0.069	-0.228	1.03E-03	3.32E-18	-0.411
cg19790294	16	88717755	*CYBA*	TSS1500	N_Shelf	0.384±0.118	0.612±0.12	-0.228	4.91E-04	1.34E-33	-0.548
cg23511432	5	137610255	*GFRA3*	TSS200	Island	0.502±0.101	0.729±0.063	-0.228	1.81E-04	1.09E-15	-0.381
cg26015401	19	49436967	*DHDH*	5'UTR/1st exon	Island	0.245±0.211	0.472±0.187	-0.228	2.19E-02	4.95E-07	-0.245
cg00544337	10	82116203	*DYDC1*	5'UTR/1st intron	Island	0.539±0.09	0.766±0.055	-0.227	7.81E-05	3.59E-07	-0.248
cg27576946	17	73893202	*TRIM65*	TSS200	Island	0.276±0.156	0.503±0.153	-0.227	4.25E-03	2.56E-16	-0.389
cg14165142	20	3778655	*CDC25B*	5'UTR	S_Shore	0.352±0.153	0.579±0.198	-0.227	5.39E-03	1.40E-34	-0.554
cg24088438	11	63974229	*FERMT3*	5'UTR/1st exon	N_Shore	0.438±0.113	0.665±0.055	-0.227	5.34E-04	3.05E-44	-0.615
cg09072560	1	32802218	*MARCKSL1*	TSS1500	Island	0.286±0.156	0.513±0.202	-0.227	6.16E-03	1.21E-16	-0.393
cg26493353	9	91793721	*SHC3*	TSS200	S_Shore	0.111±0.095	0.338±0.264	-0.227	3.89E-03	1.52E-07	-0.255
cg08028295	11	64612633	*CDC42BPG*	TSS1500	Island	0.193±0.137	0.42±0.181	-0.227	2.51E-03	8.86E-16	-0.382
cg09477407	12	104697545	*EID3*	5'UTR/1st exon	Island	0.375±0.192	0.601±0.153	-0.227	1.31E-02	2.99E-15	-0.376
cg18473090	19	49436786	*DHDH*	TSS200	Island	0.146±0.147	0.373±0.206	-0.227	4.91E-03	1.42E-06	-0.235
cg00338735	4	53728038	*RASL11B*	TSS1500	Island	0.419±0.2	0.646±0.194	-0.227	1.82E-02	1.31E-06	-0.236
cg20781880	19	3179741	*S1PR4*	1st exon	Island	0.421±0.07	0.648±0.131	-0.226	7.60E-06	2.09E-14	-0.365
cg11877254	19	10222051	*P2RY11*	TSS200	N_Shelf	0.557±0.121	0.783±0.059	-0.226	8.55E-04	3.29E-09	-0.286
cg19241689	6	33245516	*B3GALT4*	1st exon	Island	0.433±0.094	0.66±0.068	-0.226	9.02E-05	2.19E-14	-0.364
cg14995562	22	38714166	*CSNK1E*	TSS1500	S_Shore	0.389±0.077	0.615±0.099	-0.226	8.55E-06	4.19E-20	-0.431
cg05985737	1	17306885	*MFAP2*	5'UTR/1st intron	Island	0.19±0.117	0.416±0.194	-0.226	1.41E-03	7.93E-06	-0.218
cg10154122	19	38754855	*SPINT2*	TSS1500	Island	0.344±0.186	0.57±0.175	-0.226	1.21E-02	1.79E-18	-0.414
cg03125427	16	88717482	*CYBA*	5'UTR/1st exon	Island	0.528±0.203	0.754±0.112	-0.226	1.62E-02	3.23E-28	-0.506
cg18016254	3	122296505	*PARP15*	5'UTR/1st exon	N_Shore	0.383±0.176	0.609±0.206	-0.226	1.13E-02	3.05E-14	-0.363
cg17691156	8	59572928	*NSMAF*	TSS1500	S_Shore	0.295±0.162	0.521±0.188	-0.226	6.79E-03	1.16E-06	-0.237
cg04053242	20	1246772	*SNPH*	TSS200	Island	0.191±0.15	0.417±0.181	-0.226	4.27E-03	1.60E-10	-0.308
cg09079275	5	157003083	*ADAM19*	TSS1500	S_Shore	0.264±0.129	0.49±0.198	-0.226	2.41E-03	2.68E-07	-0.250
cg18125510	14	100841768	*WARS*	5'UTR/1st exon	N_Shore	0.187±0.146	0.412±0.233	-0.226	7.06E-03	2.52E-09	-0.288
cg16882373	7	26191458	*NFE2L3*	TSS1500	N_Shore	0.569±0.159	0.794±0.099	-0.225	4.46E-03	9.17E-24	-0.468
cg27125093	19	3179828	*S1PR4*	1st exon	Island	0.599±0.106	0.824±0.057	-0.225	3.28E-04	1.22E-22	-0.457
cg17745097	1	11863365	*MTHFR*	5'UTR/1st intron	N_Shelf	0.487±0.112	0.712±0.057	-0.225	5.12E-04	1.07E-05	-0.215
cg02288341	5	137610226	*GFRA3*	5'UTR/1st exon	Island	0.513±0.093	0.738±0.066	-0.225	9.01E-05	4.05E-15	-0.374
cg20337103	11	118781778	*BCL9L*	TSS200	S_Shore	0.328±0.125	0.553±0.261	-0.225	6.80E-03	1.43E-08	-0.275
cg03359362	19	47289611	*SLC1A5*	5'UTR/1st intron	N_Shore	0.216±0.126	0.441±0.217	-0.225	3.13E-03	9.64E-23	-0.458
cg20695297	19	3178844	*S1PR4*	1st exon	Island	0.587±0.105	0.812±0.067	-0.225	2.62E-04	1.12E-24	-0.476
cg21387418	6	33244775	*B3GALT4*	TSS200	Island	0.306±0.118	0.531±0.082	-0.225	6.03E-04	4.65E-59	-0.688
cg18581405	19	3180035	*S1PR4*	1st exon/3'UTR	S_Shore	0.474±0.134	0.699±0.121	-0.225	1.52E-03	1.17E-40	-0.594
cg22070406	18	55019708	*ST8SIA3*	TSS200	Island	0.472±0.075	0.697±0.08	-0.225	6.10E-06	2.20E-07	-0.252
cg13266096	11	62366806	*MTA2*	5'UTR	N_Shore	0.301±0.116	0.526±0.132	-0.225	5.40E-04	7.81E-17	-0.395
cg07846167	1	16084758	*FBLIM1*	5'UTR	N_Shore	0.329±0.132	0.554±0.164	-0.225	1.82E-03	7.22E-15	-0.371
cg20967139	12	54785055	*ZNF385A*	5'UTR/1st exon	Island	0.532±0.105	0.756±0.064	-0.225	2.76E-04	1.73E-07	-0.254
cg06126335	7	73497616	*LIMK1*	TSS1500	N_Shore	0.477±0.132	0.702±0.079	-0.225	1.44E-03	1.68E-07	-0.254
cg13928782	11	2162445	*IGF2*	5'UTR	Island	0.539±0.075	0.763±0.076	-0.224	6.88E-06	4.84E-11	-0.317
cg18628371	14	24641189	*REC8*	TSS200	Island	0.539±0.091	0.763±0.049	-0.224	1.08E-04	2.81E-08	-0.269
cg03280026	7	112430707	*TMEM168*	TSS1500	S_Shore	0.327±0.139	0.552±0.151	-0.224	2.28E-03	3.93E-12	-0.333
cg14525270	1	43766647	*TIE1*	5'UTR/1st exon	N_Shelf	0.557±0.127	0.781±0.055	-0.224	1.28E-03	6.50E-09	-0.281
cg19166302	3	122296613	*PARP15*	1st exon	Island	0.427±0.186	0.651±0.183	-0.224	1.33E-02	2.22E-07	-0.252
cg06575572	22	45705265	*FAM118A*	1st exon	N_Shore	0.394±0.106	0.619±0.113	-0.224	2.30E-04	5.17E-08	-0.264
cg17950095	7	27224700	*HOXA11*	1st exon	N_Shore	0.358±0.067	0.582±0.145	-0.224	1.63E-05	1.26E-05	-0.213
cg14788334	1	26606110	*SH3BGRL3*	TSS200	N_Shore	0.414±0.093	0.638±0.126	-0.224	8.52E-05	8.95E-16	-0.382
cg08145723	11	130029478	*ST14*	TSS1500	N_Shore	0.394±0.215	0.618±0.19	-0.224	2.61E-02	1.66E-17	-0.403
cg16408081	11	67205869	*PTPRCAP*	TSS1500	S_Shelf	0.583±0.08	0.806±0.084	-0.224	1.43E-05	5.68E-41	-0.596
cg06905823	22	39638316	*PDGFB*	TSS1500	Island	0.086±0.065	0.309±0.241	-0.224	1.43E-03	7.57E-06	-0.219
cg26117023	2	74782096	*DOK1*	5'UTR/1st intron	Island	0.284±0.087	0.507±0.175	-0.224	2.27E-04	1.70E-05	-0.210
cg19989295	14	24641077	*REC8*	TSS200	Island	0.556±0.099	0.779±0.072	-0.223	1.55E-04	3.12E-08	-0.268
cg25607249	19	47288039	*SLC1A5*	5'UTR/1st exon	N_Shelf	0.231±0.106	0.455±0.114	-0.223	2.40E-04	7.62E-21	-0.439
cg02732252	16	3068014	*CLDN6*	5'UTR/1st intron	Island	0.536±0.079	0.759±0.063	-0.223	1.99E-05	7.03E-08	-0.262
cg25262528	6	86158972	*NT5E*	TSS1500	N_Shore	0.403±0.147	0.626±0.205	-0.223	5.35E-03	1.02E-11	-0.327
cg18213979	17	29297873	*RNF135*	TSS200	N_Shore	0.382±0.158	0.605±0.185	-0.223	6.25E-03	3.07E-47	-0.632
cg23950233	6	33245739	*B3GALT4*	1st exon	S_Shore	0.514±0.122	0.737±0.107	-0.223	7.86E-04	7.68E-06	-0.218
cg01320573	6	33387003	*SYNGAP1*	TSS1500	S_Shore	0.588±0.109	0.811±0.038	-0.223	5.49E-04	1.17E-14	-0.368
cg12044599	11	67206308	*PTPRCAP*	TSS1500	S_Shelf	0.689±0.102	0.912±0.064	-0.223	2.41E-04	4.43E-49	-0.641
cg03665078	5	118689961	*TNFAIP8*	5'UTR	N_Shore	0.61±0.107	0.833±0.029	-0.223	5.34E-04	7.21E-28	-0.504
cg06594281	17	73083692	*SLC16A5*	TSS1500	N_Shore	0.487±0.1	0.709±0.035	-0.223	3.24E-04	5.96E-32	-0.536
cg14588642	11	67139423	*CLCF1*	5'UTR/1st intron	N_Shore	0.354±0.151	0.576±0.232	-0.223	8.61E-03	3.53E-07	-0.248
cg23963071	6	2901712	*SERPINB9*	5'UTR/1st intron	N_Shore	0.45±0.092	0.672±0.076	-0.223	7.78E-05	1.41E-05	-0.212
cg21647257	19	36523945	*CLIP3*	TSS1500	S_Shore	0.398±0.06	0.62±0.106	-0.222	7.44E-07	1.05E-05	-0.215
cg10829727	17	73084309	*SLC16A5*	5'UTR/1st intron	Island	0.444±0.065	0.666±0.083	-0.222	1.01E-06	7.28E-19	-0.418
cg01602621	19	11689813	*ACP5*	5'UTR/1st exon	S_Shore	0.387±0.138	0.61±0.24	-0.222	6.91E-03	2.25E-21	-0.444
cg04249754	1	32715483	*LCK*	TSS1500	S_Shore	0.433±0.045	0.655±0.08	-0.222	7.06E-09	2.35E-12	-0.336
cg08089518	17	39941714	*JUP*	5'UTR/1st intron	N_Shore	0.307±0.133	0.53±0.223	-0.222	4.69E-03	3.66E-10	-0.302
cg08747889	6	43043786	*PTK7*	TSS1500	N_Shore	0.456±0.138	0.678±0.148	-0.222	2.20E-03	1.24E-23	-0.466
cg04490178	19	3179545	*S1PR4*	1st exon	Island	0.491±0.096	0.713±0.088	-0.222	1.03E-04	2.52E-20	-0.434
cg23979458	17	80291982	*SECTM1*	TSS200	Island	0.405±0.169	0.628±0.135	-0.222	7.22E-03	2.90E-05	-0.204
cg15777261	2	73462569	*CCT7*	5'UTR/1st intron	S_Shore	0.494±0.114	0.716±0.149	-0.222	6.50E-04	2.02E-20	-0.435
cg22812275	6	159240814	*EZR*	TSS1500	S_Shore	0.376±0.121	0.598±0.192	-0.222	1.84E-03	4.45E-13	-0.347

cg08167706	7	134144106	*AKR1B1*	TSS200	S_Shore	0.455±0.218	0.677±0.177	-0.222	2.75E-02	1.33E-09	-0.293
cg18345635	17	80186080	*SLC16A3*	TSS1500	N_Shore	0.441±0.073	0.663±0.089	-0.222	4.98E-06	1.25E-10	-0.310
cg02701825	17	73083632	*SLC16A5*	TSS1500	N_Shore	0.531±0.109	0.752±0.064	-0.222	4.33E-04	5.57E-28	-0.504
cg07773769	3	52280329	*PPM1M*	5'UTR/1st exon	S_Shore	0.253±0.119	0.474±0.155	-0.222	9.26E-04	1.56E-26	-0.492
cg26574680	1	235811860	*GNG4*	5'UTR/1st intron	N_Shelf	0.361±0.139	0.583±0.242	-0.222	7.47E-03	2.24E-05	-0.207
cg17471836	5	118691007	*TNFAIP8*	5'UTR	N_Shore	0.664±0.115	0.886±0.027	-0.221	8.60E-04	9.10E-20	-0.428
cg10465028	11	63753203	*OTUB1*	TSS200	N_Shore	0.438±0.093	0.66±0.165	-0.221	2.49E-04	2.59E-10	-0.305
cg21462428	12	4381448	*CCND2*	TSS1500	Island	0.374±0.169	0.595±0.217	-0.221	1.20E-02	1.28E-05	-0.213
cg19430780	19	38397004	*SIPA1L3*	TSS1500	Island	0.223±0.097	0.444±0.199	-0.221	8.94E-04	1.75E-05	-0.210
cg04986504	11	12031266	*DKK3*	TSS1500	S_Shore	0.65±0.115	0.871±0.059	-0.221	6.58E-04	7.37E-12	-0.329
cg07519235	16	19894978	*GPRC5B*	5'UTR/1st intron	Island	0.48±0.242	0.701±0.219	-0.221	4.65E-02	1.08E-12	-0.341
cg04569429	17	37024625	*LASP1*	TSS1500	N_Shore	0.166±0.059	0.387±0.25	-0.221	2.00E-03	2.25E-05	-0.207
cg11087049	14	51296586	*NIN*	5'UTR	N_Shore	0.21±0.087	0.431±0.215	-0.221	1.05E-03	2.45E-09	-0.288
cg15350899	11	118781763	*BCL9L*	TSS200	S_Shore	0.416±0.129	0.637±0.239	-0.221	5.78E-03	2.18E-10	-0.306
cg01406381	19	47288263	*SLC1A5*	5'UTR/1st intron	N_Shelf	0.169±0.09	0.39±0.139	-0.221	9.45E-05	6.85E-20	-0.429
cg21054441	15	68871409	*CORO2B*	5'UTR	Island	0.385±0.132	0.605±0.162	-0.220	2.09E-03	1.15E-06	-0.237
cg00508334	7	23510438	*IGF2BP3*	TSS1500	S_Shore	0.081±0.094	0.301±0.218	-0.220	1.42E-03	2.16E-08	-0.271
cg05935571	3	32023257	*OSBPL10*	5'UTR/1st exon	Island	0.113±0.059	0.333±0.25	-0.220	2.03E-03	1.28E-05	-0.213
cg09375033	3	137833634	*DZIP1L*	5'UTR/1st intron	N_Shore	0.485±0.161	0.705±0.197	-0.220	8.38E-03	4.98E-30	-0.521
cg00792966	6	21595983	*SOX4*	1st exon	N_Shore	0.179±0.193	0.399±0.216	-0.220	2.08E-02	2.78E-12	-0.335
cg11375102	16	1583810	*TMEM204*	5'UTR/1st exon	Island	0.503±0.105	0.723±0.05	-0.220	3.98E-04	2.13E-09	-0.290
cg26018685	12	46663660	*SLC38A1*	TSS1500	S_Shore	0.493±0.178	0.712±0.182	-0.220	1.21E-02	7.18E-26	-0.487
cg15825065	8	133687344	*LRRC6*	1st exon	N_Shore	0.347±0.117	0.566±0.19	-0.220	1.67E-03	1.61E-10	-0.308
cg15681358	10	99474521	*MARVELD1*	1st exon/3'UTR	S_Shore	0.529±0.102	0.749±0.034	-0.220	4.02E-04	1.43E-33	-0.547
cg24865779	21	34775042	*IFNGR2*	TSS200	N_Shore	0.573±0.079	0.792±0.044	-0.220	4.33E-05	3.18E-17	-0.400
cg25163476	11	2161586	*IGF2*	5'UTR	Island	0.466±0.111	0.686±0.073	-0.220	4.67E-04	5.17E-13	-0.346
cg23734137	15	96868114	*NR2F2*	TSS1500	S_Shore	0.52±0.125	0.739±0.082	-0.219	1.15E-03	1.66E-06	-0.233
cg02674352	11	61275826	*LRRC10B*	TSS1500	Island	0.308±0.128	0.527±0.204	-0.219	3.36E-03	2.84E-05	-0.205
cg09130035	12	21810139	*LDHB*	5'UTR/1st intron	N_Shore	0.345±0.113	0.564±0.134	-0.219	5.37E-04	1.24E-32	-0.541
cg17280299	5	132082690	*CCNI2*	TSS1500	N_Shore	0.428±0.205	0.647±0.193	-0.219	2.41E-02	1.30E-19	-0.426
cg13500576	1	51796588	*TTC39A*	TSS1500	Island	0.114±0.061	0.333±0.188	-0.219	1.94E-04	6.40E-10	-0.298
cg03717367	9	139980219	*MAN1B1*	TSS1500	N_Shore	0.47±0.119	0.688±0.083	-0.219	8.03E-04	9.42E-09	-0.278
cg22538054	12	95941988	*USP44*	5'UTR/1st exon	Island	0.475±0.159	0.694±0.091	-0.219	5.32E-03	4.12E-07	-0.246
cg12476763	19	58111647	*ZNF530*	5'UTR/1st intron	Island	0.189±0.147	0.408±0.182	-0.219	4.82E-03	5.46E-12	-0.331
cg01346718	22	38713874	*CSNK1E*	5'UTR/1st intron	S_Shore	0.546±0.143	0.765±0.093	-0.219	2.75E-03	1.62E-16	-0.391
cg22032521	11	125757328	*HYLS1*	5'UTR/1st exon	Island	0.2±0.094	0.418±0.202	-0.219	9.79E-04	1.31E-08	-0.275
cg20326704	10	70321770	*TET1*	5'UTR/1st intron	S_Shore	0.366±0.101	0.584±0.221	-0.218	2.08E-03	1.17E-09	-0.294
cg03059018	14	61190965	*SIX4*	TSS200	S_Shore	0.122±0.14	0.34±0.262	-0.218	1.13E-02	4.67E-06	-0.223
cg15523958	2	43864929	*PLEKHH2*	5'UTR/1st intron	Island	0.307±0.118	0.525±0.138	-0.218	8.50E-04	2.99E-09	-0.287
cg22245273	19	4302448	*TMIGD2*	TSS200	N_Shelf	0.451±0.12	0.669±0.104	-0.218	8.04E-04	7.69E-12	-0.329
cg15743533	20	815345	*FAM110A*	5'UTR/1st intron	S_Shore	0.45±0.158	0.668±0.157	-0.218	6.03E-03	1.45E-26	-0.493
cg20717612	12	21810983	*LDHB*	TSS1500	S_Shore	0.357±0.07	0.575±0.177	-0.218	1.51E-04	4.24E-13	-0.347
cg20064928	10	70586817	*STOX1*	TSS1500	N_Shore	0.102±0.078	0.319±0.15	-0.218	6.63E-05	5.53E-09	-0.282
cg01294686	1	26606093	*SH3BGRL3*	TSS200	N_Shore	0.273±0.13	0.491±0.198	-0.218	3.31E-03	1.10E-12	-0.341
cg05337743	19	54369571	*MYADM*	5'UTR/1st exon	N_Shelf	0.44±0.104	0.658±0.081	-0.218	2.65E-04	2.19E-10	-0.306
cg06340276	4	89204787	*PPM1K*	5'UTR/1st intron	N_Shore	0.146±0.134	0.364±0.208	-0.218	4.52E-03	5.04E-08	-0.264
cg27457191	7	77429766	*PHTF2*	5'UTR/1st intron	S_Shore	0.618±0.097	0.835±0.039	-0.217	2.90E-04	7.36E-12	-0.329
cg15955046	2	3643682	*COLEC11*	5'UTR/1st intron	S_Shore	0.428±0.058	0.646±0.084	-0.217	2.99E-07	1.30E-17	-0.404
cg14667273	1	1369934	*VWA1*	TSS1500	N_Shore	0.412±0.074	0.629±0.077	-0.217	8.05E-06	3.79E-15	-0.374
cg13545212	17	6617192	*SLC13A5*	TSS1500	Island	0.464±0.109	0.681±0.103	-0.217	3.66E-04	3.01E-07	-0.249
cg14462645	6	52226698	*PAQR8*	TSS1500	N_Shore	0.482±0.12	0.699±0.087	-0.217	8.85E-04	7.40E-10	-0.297
cg26653147	15	71146819	*LARP6*	TSS1500	Island	0.212±0.13	0.429±0.218	-0.217	4.69E-03	8.57E-11	-0.313
cg06437004	12	21810458	*LDHB*	5'UTR/1st exon	N_Shore	0.358±0.121	0.575±0.126	-0.217	9.52E-04	9.69E-17	-0.394
cg26537639	16	88717374	*CYBA*	1st exon	Island	0.568±0.104	0.784±0.036	-0.217	4.88E-04	3.04E-43	-0.609
cg00920691	19	12075601	*ZNF763*	TSS1500	N_Shore	0.317±0.118	0.534±0.228	-0.217	4.06E-03	1.85E-22	-0.455
cg24709033	7	27224568	*HOXA11*	1st exon	N_Shore	0.517±0.113	0.733±0.101	-0.217	5.36E-04	2.81E-07	-0.250
cg02283238	5	118691126	*TNFAIP8*	5'UTR	N_Shore	0.619±0.117	0.836±0.032	-0.217	1.11E-03	5.70E-26	-0.487
cg05776388	17	72983414	*CDR2L*	TSS1500	N_Shore	0.32±0.159	0.536±0.211	-0.216	1.01E-02	4.21E-06	-0.224
cg21487207	12	54410576	*HOXC6*	TSS200	S_Shore	0.172±0.095	0.388±0.205	-0.216	1.22E-03	1.21E-05	-0.214
cg26043257	4	15780238	*CD38*	1st exon	Island	0.318±0.221	0.534±0.21	-0.216	3.61E-02	1.53E-05	-0.211
cg22070243	2	172378774	*CYBRD1*	5'UTR/1st exon	N_Shore	0.19±0.13	0.406±0.275	-0.216	1.21E-02	1.13E-10	-0.311
cg27365208	19	19778986	*ZNF101*	5'UTR/1st exon	N_Shore	0.496±0.097	0.712±0.041	-0.216	2.76E-04	1.14E-09	-0.294
cg22289360	9	79073908	*GCNT1*	5'UTR/1st intron	Island	0.412±0.078	0.628±0.146	-0.216	5.98E-05	1.43E-07	-0.256
cg24437535	1	150121441	*PLEKHO1*	TSS1500	N_Shore	0.439±0.119	0.655±0.151	-0.216	1.13E-03	1.45E-09	-0.292
cg00244040	14	61788914	*PRKCH*	1st exon	Island	0.33±0.11	0.546±0.148	-0.216	6.53E-04	5.26E-07	-0.244
cg17276021	1	16084445	*FBLIM1*	5'UTR/1st intron	N_Shore	0.436±0.115	0.652±0.148	-0.215	8.68E-04	4.59E-25	-0.479
cg01122251	10	14995625	*DCLRE1C*	5'UTR/1st intron	N_Shore	0.141±0.136	0.356±0.302	-0.215	1.93E-02	7.92E-24	-0.468
cg26998044	17	8869155	*PIK3R5*	TSS200	Island	0.287±0.194	0.502±0.211	-0.215	2.30E-02	3.51E-05	-0.202
cg24366657	11	2160932	*IGF2*	5'UTR	Island	0.471±0.064	0.686±0.173	-0.215	1.14E-04	1.13E-19	-0.427
cg04263436	6	33245467	*B3GALT4*	1st exon	Island	0.61±0.095	0.825±0.051	-0.215	2.19E-04	2.21E-22	-0.454
cg01735277	15	75077691	*CSK*	5'UTR	S_Shelf	0.598±0.141	0.813±0.059	-0.215	3.13E-03	5.92E-22	-0.450
cg13755546	2	74425580	*MTHFD2*	TSS200	Island	0.402±0.191	0.617±0.16	-0.215	1.70E-02	4.06E-27	-0.497
cg01857475	12	104697193	*EID3*	TSS1500	N_Shore	0.296±0.179	0.511±0.265	-0.215	2.57E-02	2.15E-11	-0.322
cg26134147	14	24036908	*AP1G2*	5'UTR/1st exon	Island	0.135±0.033	0.35±0.227	-0.215	9.57E-04	5.58E-14	-0.359
cg17355191	9	136039576	*GBGT1*	TSS1500	Island	0.552±0.169	0.767±0.088	-0.215	8.13E-03	9.82E-28	-0.502
cg21270243	2	216176248	*ATIC*	TSS1500	N_Shore	0.06±0.057	0.275±0.284	-0.215	6.03E-03	5.99E-11	-0.315
cg16864139	2	43864212	*PLEKHH2*	TSS1500	N_Shore	0.11±0.118	0.325±0.197	-0.215	2.41E-03	5.44E-06	-0.222
cg27262236	20	55965077	*RBM38*	TSS1500	S_Shore	0.305±0.183	0.519±0.181	-0.215	1.57E-02	8.47E-07	-0.240
cg02397514	1	935139	*HES4*	1st exon	Island	0.115±0.162	0.33±0.25	-0.215	1.65E-02	7.96E-06	-0.218
cg17957827	17	79895524	*PYCR1*	TSS1500	N_Shelf	0.16±0.139	0.374±0.229	-0.215	7.75E-03	1.13E-11	-0.326
cg15957394	4	7941823	*AFAP1*	TSS200	Island	0.36±0.194	0.575±0.273	-0.214	3.44E-02	2.55E-18	-0.412
cg17644557	6	86159103	*NT5E*	TSS200	N_Shore	0.272±0.112	0.487±0.213	-0.214	2.73E-03	3.22E-08	-0.268
cg15073853	19	18549131	*ISYNA1*	TSS1500	Island	0.471±0.154	0.685±0.062	-0.214	5.22E-03	4.99E-21	-0.441
cg26090107	11	60718882	*SLC15A3*	1st exon	Island	0.325±0.211	0.539±0.204	-0.214	3.12E-02	2.04E-05	-0.208
cg16289449	11	63974162	*FERMT3*	5'UTR/1st exon	N_Shore	0.431±0.106	0.645±0.051	-0.214	5.19E-04	2.56E-44	-0.616
cg05693489	6	32813441	*PSMB8*	TSS1500	S_Shore	0.469±0.119	0.683±0.059	-0.214	1.09E-03	1.15E-17	-0.405
cg20826709	11	67171145	*TBC1D10C*	TSS1500	S_Shelf	0.556±0.098	0.769±0.052	-0.214	2.85E-04	2.37E-19	-0.423

cg16541026	3	49026934	*P4HTM*	TSS1500	N_Shore	0.362±0.103	0.575±0.169	-0.214	6.80E-04	2.57E-12	-0.336
cg03549705	16	29674618	*SPN*	5'UTR/1st exon	N_Shore	0.608±0.103	0.822±0.035	-0.214	4.94E-04	4.69E-37	-0.571
cg13634319	12	56323727	*DGKA*	TSS1500	S_Shelf	0.525±0.141	0.739±0.081	-0.214	2.95E-03	6.62E-11	-0.314
cg24859158	1	26605913	*SH3BGRL3*	TSS1500	N_Shore	0.436±0.107	0.649±0.077	-0.214	4.07E-04	2.12E-17	-0.402
cg11530914	16	67281528	*FHOD1*	TSS200	Island	0.16±0.096	0.373±0.201	-0.214	1.25E-03	1.71E-06	-0.233
cg18198306	6	153303350	*FBXO5*	5'UTR/1st intron	N_Shore	0.177±0.108	0.391±0.225	-0.214	3.30E-03	1.59E-07	-0.255
cg21333861	6	33244976	*B3GALT4*	5'UTR/1st exon	Island	0.513±0.203	0.727±0.081	-0.213	2.07E-02	6.48E-57	-0.679
cg19269039	1	111743200	*DENND2D*	5'UTR/1st exon	N_Shelf	0.566±0.061	0.779±0.085	-0.213	7.63E-07	1.54E-37	-0.574
cg24890054	2	18059752	*KCNS3*	5'UTR/1st intron	Island	0.158±0.181	0.371±0.272	-0.213	2.91E-02	6.42E-09	-0.281
cg20250722	6	26522136	*HCG11*	1st exon	Island	0.511±0.107	0.724±0.039	-0.213	6.33E-04	3.01E-41	-0.598
cg20299703	1	65731886	*DNAJC6*	5'UTR/1st intron	S_Shore	0.399±0.129	0.612±0.265	-0.213	1.12E-02	1.52E-08	-0.274
cg03719155	19	11689880	*ACP5*	TSS1500	S_Shore	0.451±0.101	0.664±0.193	-0.213	1.23E-03	1.90E-26	-0.492
cg27039625	6	86159096	*NT5E*	TSS1500	N_Shore	0.353±0.128	0.566±0.189	-0.213	3.28E-03	1.11E-10	-0.311
cg03947026	1	52832320	*CC2D1B*	TSS1500	S_Shore	0.286±0.124	0.498±0.192	-0.213	2.92E-03	4.20E-06	-0.224
cg25998680	6	43044239	*PTK7*	1st exon	Island	0.442±0.174	0.654±0.068	-0.212	1.03E-02	4.41E-20	-0.431
cg13191049	15	99645663	*SYNM*	1st exon	Island	0.453±0.185	0.665±0.101	-0.212	1.39E-02	6.82E-16	-0.384
cg09118312	12	21810630	*LDHB*	5'UTR/1st exon	Island	0.374±0.185	0.586±0.207	-0.212	2.03E-02	5.10E-12	-0.331
cg01131866	5	61601529	*KIF2A*	TSS1500	N_Shore	0.271±0.091	0.483±0.132	-0.212	1.44E-04	3.29E-06	-0.227
cg16681526	1	6526469	*TNFRSF25*	TSS1500	S_Shore	0.547±0.129	0.759±0.093	-0.212	1.70E-03	3.03E-19	-0.422
cg05417285	12	117176367	*RNFT2*	5'UTR/1st intron	Island	0.116±0.128	0.328±0.242	-0.212	7.92E-03	4.83E-06	-0.223
cg05832051	19	54369469	*MYADM*	5'UTR/1st exon	N_Shelf	0.522±0.096	0.734±0.07	-0.212	1.90E-04	2.67E-12	-0.336
cg25885803	11	47290281	*MADD*	TSS1500	N_Shore	0.394±0.096	0.606±0.092	-0.212	1.52E-04	2.49E-05	-0.206
cg20780880	6	8103084	*EEF1E1*	TSS1500	S_Shore	0.416±0.139	0.628±0.211	-0.212	6.70E-03	1.80E-07	-0.254
cg00618865	3	129324699	*PLXND1*	1st exon	Island	0.268±0.156	0.48±0.19	-0.212	8.82E-03	2.92E-08	-0.269
cg03189210	6	33245474	*B3GALT4*	1st exon	Island	0.524±0.094	0.735±0.06	-0.212	1.82E-04	3.06E-20	-0.433
cg09300114	17	73084329	*SLC16A5*	5'UTR/1st intron	Island	0.62±0.092	0.832±0.038	-0.211	2.20E-04	2.88E-27	-0.499
cg27583010	16	30198505	*LOC606724*	TSS1500	S_Shelf	0.36±0.151	0.572±0.261	-0.211	1.66E-02	7.83E-06	-0.218
cg12065285	1	155946346	*ARHGEF2*	5'UTR/1st intron	N_Shore	0.357±0.123	0.569±0.153	-0.211	1.71E-03	2.48E-43	-0.610
cg13528854	4	15704795	*BST1*	1st exon	Island	0.378±0.159	0.589±0.118	-0.211	6.69E-03	3.74E-30	-0.522
cg06737981	22	23523942	*BCR*	1st exon	Island	0.535±0.072	0.746±0.063	-0.211	5.14E-05	1.38E-11	-0.325
cg00337466	10	135090565	*ADAM8*	TSS200	S_Shore	0.473±0.071	0.684±0.065	-0.211	9.23E-06	2.16E-09	-0.289
cg12297409	10	44101714	*ZNF485*	TSS200	N_Shore	0.327±0.086	0.538±0.164	-0.211	2.64E-04	1.40E-05	-0.212
cg04566159	19	11689715	*ACP5*	5'UTR/1st exon	Island	0.433±0.129	0.644±0.165	-0.211	2.61E-03	5.25E-25	-0.479
cg00236576	17	81039406	*METRNL*	5'UTR/1st intron	Island	0.379±0.112	0.59±0.119	-0.211	6.20E-04	1.01E-06	-0.238
cg00516222	17	27949795	*CORO6*	TSS1500	Island	0.431±0.186	0.642±0.188	-0.211	1.91E-02	7.29E-07	-0.241
cg17001399	14	61191008	*SIX4*	TSS200	S_Shore	0.083±0.1	0.294±0.239	-0.211	4.10E-03	3.93E-05	-0.201
cg15379633	22	23487586	*RAB36*	1st exon	Island	0.5±0.138	0.711±0.075	-0.211	2.88E-03	4.58E-14	-0.360
cg26922780	16	88769443	*RNF166*	5'UTR/1st exon	N_Shelf	0.675±0.081	0.886±0.056	-0.211	5.16E-05	6.99E-14	-0.358
cg17416730	6	33245541	*B3GALT4*	1st exon	Island	0.484±0.094	0.694±0.045	-0.211	2.57E-04	6.48E-26	-0.487
cg17319374	2	135476880	*TMEM163*	TSS1500	Island	0.314±0.196	0.525±0.138	-0.211	2.02E-02	4.46E-07	-0.246
cg22963915	19	3785855	*MATK*	5'UTR/1st intron	Island	0.248±0.171	0.459±0.225	-0.211	1.77E-02	1.14E-07	-0.258
cg15695852	2	43864466	*PLEKHH2*	5'UTR/1st exon	Island	0.101±0.122	0.311±0.244	-0.210	7.43E-03	8.07E-06	-0.218
cg10167849	6	34206152	*HMGA1*	5'UTR	Island	0.268±0.151	0.478±0.185	-0.210	7.49E-03	4.20E-40	-0.591
cg07759394	11	134201954	*GLB1L2*	5'UTR/1st exon	Island	0.458±0.223	0.668±0.205	-0.210	4.11E-02	3.37E-08	-0.268
cg00353923	7	127671044	*LRRC4*	TSS200	N_Shore	0.61±0.058	0.82±0.04	-0.210	2.71E-06	1.36E-21	-0.447
cg18856652	2	43864913	*PLEKHH2*	5'UTR/1st intron	Island	0.278±0.137	0.488±0.214	-0.210	6.90E-03	2.49E-08	-0.270
cg19759135	5	179221059	*LTC4S*	5'UTR/1st exon	N_Shore	0.576±0.092	0.786±0.056	-0.210	1.66E-04	6.77E-13	-0.344
cg08335767	6	34206495	*HMGA1*	5'UTR	S_Shore	0.339±0.089	0.548±0.118	-0.210	9.79E-05	6.76E-32	-0.535
cg23373626	5	141031295	*FCHSD1*	TSS1500	S_Shore	0.265±0.137	0.474±0.246	-0.210	1.09E-02	3.53E-26	-0.489
cg00113050	10	102756314	*LZTS2*	TSS1500	N_Shore	0.163±0.056	0.373±0.187	-0.209	2.52E-04	9.62E-13	-0.342
cg20792833	11	67205195	*PTPRCAP*	TSS200	S_Shore	0.582±0.107	0.791±0.047	-0.209	6.45E-04	7.15E-49	-0.640
cg09039664	20	44034247	*DBNDD2*	TSS1500	N_Shore	0.289±0.086	0.498±0.108	-0.209	6.37E-05	1.96E-09	-0.290
cg06741043	15	68871574	*CORO2B*	5'UTR	Island	0.269±0.163	0.478±0.16	-0.209	9.37E-03	3.26E-06	-0.227
cg24776469	2	174219348	*CDCA7*	TSS1500	N_Shore	0.278±0.117	0.487±0.215	-0.209	4.07E-03	1.39E-13	-0.354
cg11205006	22	26875499	*HPS4*	1st exon	N_Shelf	0.375±0.163	0.583±0.239	-0.209	1.78E-02	4.87E-06	-0.223
cg03465499	11	68781279	*MRGPRF*	TSS1500	S_Shelf	0.578±0.089	0.786±0.062	-0.208	1.28E-04	1.25E-11	-0.326
cg27274542	20	57267680	*NPEPL1*	TSS200	Island	0.322±0.109	0.531±0.207	-0.208	2.74E-03	6.40E-16	-0.384
cg20001829	11	118402342	*TMEM25*	5'UTR/1st intron	S_Shore	0.275±0.195	0.483±0.235	-0.208	3.16E-02	5.92E-38	-0.577
cg25589890	6	150284777	*ULBP1*	TSS1500	Island	0.27±0.223	0.478±0.181	-0.208	3.97E-02	5.73E-06	-0.221
cg03946762	12	54785043	*ZNF385A*	5'UTR/1st exon	Island	0.559±0.094	0.767±0.039	-0.208	2.92E-04	5.83E-09	-0.282
cg12314713	17	47074576	*IGF2BP1*	TSS200	Island	0.231±0.119	0.439±0.279	-0.208	1.38E-02	1.49E-05	-0.212
cg11104418	17	76125211	*TMC6*	5'UTR/1st intron	N_Shelf	0.063±0.081	0.271±0.25	-0.208	4.30E-03	9.29E-07	-0.239
cg23985447	5	118691066	*TNFAIP8*	5'UTR	N_Shore	0.487±0.116	0.694±0.124	-0.208	9.68E-04	1.67E-22	-0.456
cg04738224	19	15618961	*CYP4F22*	TSS1500	N_Shore	0.386±0.071	0.593±0.155	-0.207	9.81E-05	1.17E-12	-0.341
cg06945399	7	127671195	*LRRC4*	TSS200	Island	0.438±0.078	0.645±0.063	-0.207	3.45E-05	1.69E-15	-0.379
cg18132212	4	40751843	*NSUN7*	TSS200	N_Shore	0.383±0.135	0.59±0.12	-0.207	2.81E-03	1.05E-23	-0.467
cg03004599	9	114937770	*SUSD1*	TSS200	Island	0.273±0.159	0.48±0.231	-0.207	1.59E-02	6.81E-18	-0.407
cg06800301	1	16766912	*NECAP2*	TSS1500	N_Shore	0.372±0.077	0.579±0.102	-0.207	2.28E-05	8.37E-07	-0.240
cg13675837	17	54910204	*DGKE*	TSS1500	N_Shore	0.492±0.116	0.698±0.15	-0.207	1.30E-03	1.14E-22	-0.457
cg16013543	1	154300241	*ATP8B2*	5'UTR/1st intron	N_Shore	0.531±0.082	0.738±0.047	-0.207	8.92E-05	1.88E-11	-0.323
cg09025324	6	116691685	*DSE*	5'UTR	N_Shore	0.518±0.097	0.724±0.159	-0.207	5.29E-04	6.48E-23	-0.460
cg16832267	2	18060102	*KCNS3*	5'UTR/1st intron	Island	0.184±0.191	0.39±0.281	-0.206	4.11E-02	1.84E-08	-0.273
cg25826226	15	41953061	*MGA*	5'UTR/1st intron	N_Shore	0.321±0.154	0.528±0.146	-0.206	6.88E-03	4.14E-08	-0.266
cg13628514	12	110271439	*TRPV4*	TSS1500	Island	0.441±0.068	0.648±0.121	-0.206	1.43E-05	1.59E-18	-0.414
cg07397616	22	38035109	*SH3BP1*	TSS1500	N_Shore	0.464±0.121	0.67±0.146	-0.206	1.71E-03	2.38E-27	-0.499
cg22105702	4	78978722	*FRAS1*	TSS200	N_Shore	0.126±0.107	0.332±0.226	-0.206	4.32E-03	1.29E-07	-0.257
cg16013670	1	241804175	*OPN3*	TSS1500	S_Shore	0.626±0.181	0.832±0.112	-0.206	1.49E-02	3.06E-23	-0.463
cg04994405	1	65731836	*DNAJC6*	5'UTR/1st intron	Island	0.415±0.095	0.621±0.189	-0.206	1.19E-03	4.14E-09	-0.284
cg19779810	17	39969264	*FKBP10*	5'UTR/1st exon	S_Shore	0.419±0.158	0.624±0.141	-0.206	8.08E-03	8.61E-22	-0.449
cg00032205	8	98290372	*TSPYL5*	TSS200	Island	0.578±0.104	0.783±0.049	-0.205	5.79E-04	3.29E-16	-0.387
cg22891070	19	46801642	*HIF3A*	5'UTR/1st exon	S_Shore	0.609±0.18	0.814±0.081	-0.205	1.44E-02	9.78E-07	-0.238
cg26751513	16	1542910	*TELO2*	TSS1500	N_Shore	0.576±0.11	0.781±0.073	-0.205	7.46E-04	1.07E-05	-0.215
cg09059904	5	76012757	*F2R*	5'UTR/1st intron	S_Shore	0.429±0.125	0.635±0.182	-0.205	3.39E-03	1.12E-07	-0.258
cg13894021	22	23487383	*RAB36*	TSS200	Island	0.203±0.119	0.408±0.163	-0.205	1.96E-03	6.56E-06	-0.220
cg02874942	19	11689649	*ACP5*	5'UTR/1st intron	Island	0.309±0.174	0.515±0.235	-0.205	2.31E-02	1.72E-16	-0.391
cg15366353	6	116691382	*DSE*	5'UTR	N_Shore	0.486±0.084	0.692±0.126	-0.205	9.12E-05	1.41E-21	-0.446
cg03001305	17	40439492	*STAT5A*	TSS1500	N_Shore	0.548±0.042	0.753±0.063	-0.205	7.06E-09	7.47E-39	-0.583

cg24049629	3	50376475	*RASSF1*	5'UTR/1st intron	S_Shore	0.622±0.114	0.828±0.039	-0.205	1.23E-03	3.61E-05	-0.202
cg15658426	5	114631545	*CCDC112*	5'UTR/1st intron	N_Shore	0.527±0.121	0.732±0.126	-0.205	1.50E-03	5.62E-22	-0.450
cg14578363	17	42785078	*DBF4B*	TSS1500	N_Shore	0.695±0.132	0.9±0.025	-0.205	3.14E-03	7.50E-09	-0.280
cg06479032	20	1246769	*SNPH*	TSS200	Island	0.19±0.133	0.395±0.164	-0.205	3.70E-03	7.92E-11	-0.313
cg07960067	2	9144629	*MBOAT2*	TSS1500	Island	0.434±0.166	0.639±0.112	-0.205	9.71E-03	1.78E-13	-0.352
cg13438834	22	45706530	*FAM118A*	5'UTR	Island	0.168±0.123	0.373±0.22	-0.205	6.22E-03	2.53E-12	-0.336
cg12169661	7	73183394	*CLDN3*	1st exon/3'UTR	Island	0.41±0.123	0.615±0.196	-0.205	3.99E-03	1.72E-16	-0.391
cg03999361	19	49436835	*DHDH*	TSS200	Island	0.215±0.178	0.42±0.202	-0.205	1.98E-02	2.19E-05	-0.207
cg26800162	12	21810808	*LDHB*	TSS200	S_Shore	0.265±0.189	0.469±0.254	-0.205	3.48E-02	8.78E-08	-0.260
cg03452174	17	27045113	*RAB34*	5'UTR/1st intron	N_Shore	0.475±0.074	0.68±0.099	-0.205	1.57E-05	5.71E-29	-0.512
cg00690082	17	40438511	*STAT5A*	TSS1500	N_Shore	0.552±0.083	0.756±0.063	-0.205	7.50E-05	1.05E-18	-0.416
cg05432996	19	49244004	*RASIP1*	TSS200	S_Shore	0.624±0.098	0.829±0.064	-0.205	3.24E-04	3.46E-09	-0.286
cg05444816	11	2162406	*IGF2*	5'UTR	Island	0.487±0.078	0.692±0.1	-0.204	2.79E-05	2.88E-12	-0.335
cg14188111	1	153608034	*S100A13*	TSS1500	S_Shore	0.502±0.155	0.706±0.076	-0.204	6.81E-03	8.08E-07	-0.240
cg20035459	16	3068707	*CLDN6*	TSS1500	S_Shore	0.576±0.127	0.78±0.035	-0.204	2.43E-03	3.95E-11	-0.318
cg01667319	11	2161128	*IGF2*	5'UTR	Island	0.539±0.084	0.744±0.052	-0.204	1.02E-04	1.61E-18	-0.414
cg26582768	10	102792043	*SFXN3*	5'UTR/1st intron	S_Shelf	0.247±0.18	0.451±0.254	-0.204	3.06E-02	9.93E-09	-0.278
cg19759788	1	16084493	*FBLIM1*	5'UTR/1st intron	N_Shore	0.286±0.112	0.49±0.231	-0.204	5.87E-03	4.67E-12	-0.332
cg06520331	10	93171002	*HECTD2*	5'UTR/1st intron	S_Shore	0.361±0.106	0.565±0.166	-0.204	1.16E-03	3.76E-17	-0.399
cg02493211	11	118781633	*BCL9L*	TSS200	Island	0.34±0.15	0.544±0.254	-0.204	1.81E-02	4.37E-08	-0.266
cg00558804	16	88769761	*RNF166*	5'UTR/1st exon	N_Shelf	0.602±0.12	0.806±0.049	-0.204	1.66E-03	2.72E-09	-0.288
cg21769117	6	31705273	*CLIC1*	TSS1500	N_Shelf	0.503±0.073	0.707±0.12	-0.204	2.58E-05	1.35E-32	-0.540
cg13999415	5	81047848	*SSBP2*	TSS1500	Island	0.449±0.164	0.653±0.109	-0.204	9.52E-03	4.48E-06	-0.224
cg23982858	12	95941869	*USP44*	5'UTR/1st exon	N_Shore	0.387±0.139	0.591±0.156	-0.204	4.68E-03	2.91E-07	-0.249
cg07302848	2	174219502	*CDCA7*	TSS200	N_Shore	0.332±0.141	0.536±0.205	-0.204	8.44E-03	2.88E-16	-0.388
cg16882226	2	101034257	*CHST10*	TSS200	Island	0.323±0.225	0.527±0.168	-0.204	4.34E-02	1.09E-27	-0.502
cg09629657	5	179220795	*LTC4S*	TSS200	N_Shore	0.369±0.113	0.572±0.102	-0.204	8.45E-04	4.46E-06	-0.224
cg06075311	10	105210937	*CALHM2*	5'UTR	N_Shore	0.276±0.088	0.479±0.207	-0.203	1.76E-03	1.78E-07	-0.254
cg17181653	6	144417537	*SF3B5*	TSS1500	S_Shore	0.508±0.179	0.711±0.181	-0.203	1.89E-02	3.77E-06	-0.226
cg21667878	11	2160980	*IGF2*	5'UTR	Island	0.641±0.095	0.844±0.073	-0.203	2.31E-04	1.28E-22	-0.457
cg11714334	22	39638092	*PDGFB*	TSS1500	N_Shore	0.159±0.061	0.362±0.223	-0.203	1.75E-03	1.39E-06	-0.235
cg08412885	6	31649479	*LY6G5C*	TSS1500	N_Shore	0.433±0.148	0.636±0.141	-0.203	6.10E-03	1.68E-05	-0.210
cg07149296	20	36889389	*KIAA1755*	TSS1500	Island	0.444±0.209	0.647±0.152	-0.203	3.22E-02	4.52E-08	-0.265
cg23327334	5	176855304	*GRK6*	5'UTR/1st intron	S_Shore	0.611±0.102	0.814±0.05	-0.203	5.46E-04	2.20E-07	-0.252
cg00917251	1	24829987	*RCAN3*	5'UTR/1st intron	S_Shore	0.203±0.095	0.405±0.239	-0.202	5.02E-03	2.59E-24	-0.473
cg13492227	17	7341436	*FGF11*	TSS1500	N_Shore	0.491±0.105	0.693±0.051	-0.202	6.86E-04	1.99E-20	-0.435
cg06342490	2	42795193	*MTA3*	5'UTR	N_Shore	0.628±0.13	0.83±0.027	-0.202	3.06E-03	3.80E-19	-0.421
cg10181419	2	264120	*SH3YL1*	1st exon	Island	0.455±0.176	0.657±0.058	-0.202	1.40E-02	1.03E-37	-0.576
cg00463982	16	1583984	*TMEM204*	5'UTR	Island	0.563±0.087	0.765±0.048	-0.202	1.66E-04	2.86E-10	-0.304
cg04378886	19	50031395	*RCN3*	5'UTR/1st intron	S_Shelf	0.563±0.098	0.765±0.042	-0.202	4.83E-04	1.65E-13	-0.353
cg15131282	2	233925030	*INPP5D*	5'UTR/1st exon	N_Shore	0.301±0.15	0.502±0.17	-0.202	8.14E-03	3.68E-10	-0.302
cg14064762	9	123688745	*TRAF1*	5'UTR	N_Shelf	0.457±0.174	0.658±0.126	-0.202	1.41E-02	3.64E-22	-0.452
cg10604168	15	65360370	*RASL12*	5'UTR/1st exon	Island	0.58±0.087	0.781±0.071	-0.202	1.14E-04	1.86E-09	-0.291
cg01932734	3	50376409	*RASSF1*	5'UTR/1st intron	S_Shore	0.491±0.107	0.693±0.081	-0.202	6.44E-04	2.13E-07	-0.252
cg09325164	1	32537942	*TMEM39B*	5'UTR/1st exon	N_Shore	0.34±0.077	0.541±0.161	-0.201	2.38E-04	1.35E-05	-0.213
cg08177833	16	88717537	*CYBA*	TSS200	N_Shelf	0.434±0.16	0.635±0.09	-0.201	8.59E-03	3.01E-34	-0.552
cg07866131	15	41850280	*TYRO3*	TSS1500	N_Shore	0.192±0.111	0.394±0.142	-0.201	1.13E-03	9.40E-15	-0.369
cg05095158	11	13030873	*RASSF10*	TSS200	Island	0.468±0.114	0.669±0.118	-0.201	1.11E-03	4.05E-06	-0.225
cg25107289	6	29760856	*HCG4*	TSS200	Island	0.264±0.107	0.465±0.196	-0.201	2.71E-03	6.07E-08	-0.263
cg23635429	10	116852470	*ATRNL1*	TSS1500	Island	0.223±0.14	0.424±0.271	-0.201	2.04E-02	4.66E-11	-0.317
cg26980244	8	24772513	*NEFM*	5'UTR/1st exon	Island	0.471±0.155	0.671±0.109	-0.201	7.55E-03	1.89E-09	-0.290
cg07638935	10	102821427	*KAZALD1*	1st exon	Island	0.327±0.16	0.527±0.234	-0.201	1.99E-02	6.19E-36	-0.564
cg19734163	7	138720989	*ZC3HAV1L*	TSS1500	Island	0.377±0.193	0.578±0.216	-0.201	3.24E-02	1.77E-19	-0.425
cg15331996	10	73848830	*SPOCK2*	TSS1500	S_Shore	0.571±0.113	0.771±0.086	-0.201	9.87E-04	3.09E-09	-0.287
cg21039495	6	29760823	*HCG4*	1st exon	Island	0.061±0.067	0.262±0.245	-0.201	4.07E-03	2.94E-08	-0.269
cg07075347	7	44104860	*PGAM2*	1st exon	Island	0.596±0.156	0.797±0.079	-0.200	7.82E-03	2.71E-20	-0.433
cg09515953	19	38747355	*PPP1R14A*	TSS200	Island	0.469±0.113	0.669±0.062	-0.200	1.14E-03	6.93E-06	-0.219
cg25246431	11	12398874	*PARVA*	TSS1500	N_Shore	0.401±0.158	0.601±0.202	-0.200	1.42E-02	2.13E-07	-0.252
cg24543696	11	12031046	*DKK3*	TSS1500	S_Shore	0.474±0.148	0.675±0.14	-0.200	6.65E-03	7.65E-10	-0.297
cg23668631	17	3796936	*CAMKK1*	TSS1500	S_Shore	0.417±0.062	0.617±0.117	-0.200	8.27E-06	1.52E-05	-0.211
cg15508379	11	2162438	*IGF2*	5'UTR	Island	0.497±0.062	0.697±0.086	-0.200	2.53E-06	1.02E-09	-0.295
cg12535569	11	67205291	*PTPRCAP*	TSS200	S_Shore	0.553±0.115	0.753±0.062	-0.200	1.32E-03	1.80E-40	-0.593
cg10337079	11	2158697	*IGF2*	5'UTR	N_Shore	0.183±0.078	0.383±0.157	-0.200	2.34E-04	2.46E-12	-0.336
cg03734595	18	9333974	*TWSG1*	TSS1500	N_Shore	0.322±0.192	0.522±0.171	-0.200	2.58E-02	4.35E-08	-0.266
cg05683808	7	157133186	*DNAJB6*	5'UTR/1st intron	S_Shelf	0.458±0.135	0.658±0.106	-0.200	3.38E-03	9.05E-06	-0.217
cg01697163	22	38035301	*SH3BP1*	TSS1500	N_Shore	0.231±0.102	0.43±0.18	-0.200	1.64E-03	5.40E-22	-0.451
cg23953820	6	30851051	*DDR1*	5'UTR/1st intron	N_Shore	0.664±0.19	0.864±0.015	-0.200	2.05E-02	1.44E-45	-0.622
cg19002337	11	2160904	*IGF2*	5'UTR	Island	0.395±0.049	0.594±0.156	-0.200	5.90E-05	1.94E-19	-0.424
cg00445824	19	18548929	*ISYNA1*	1st exon	Island	0.41±0.192	0.61±0.195	-0.200	2.90E-02	1.84E-10	-0.307
cg21844450	20	8112956	*PLCB1*	5'UTR/1st exon	Island	0.1±0.139	0.299±0.255	-0.200	1.71E-02	8.15E-09	-0.279
cg21306329	17	73083876	*SLC16A5*	5'UTR/1st exon	Island	0.587±0.062	0.786±0.052	-0.200	5.16E-06	3.67E-24	-0.471
cg10289074	14	24682916	*CHMP4A*	TSS1500	S_Shore	0.312±0.137	0.512±0.107	-0.200	3.81E-03	5.07E-17	-0.397
cg22669060	21	34774882	*IFNGR2*	TSS1500	N_Shore	0.578±0.119	0.777±0.059	-0.200	1.70E-03	1.27E-10	-0.310
cg22326553	12	124155458	*TCTN2*	TSS1500	N_Shore	0.399±0.151	0.598±0.178	-0.200	9.78E-03	5.88E-08	-0.263
cg03404662	9	114937740	*SUSD1*	TSS200	Island	0.333±0.163	0.533±0.208	-0.199	1.75E-02	5.39E-21	-0.441
cg21159568	7	2445331	*CHST12*	5'UTR/1st intron	S_Shore	0.64±0.123	0.839±0.043	-0.199	2.22E-03	8.51E-12	-0.328
cg00219653	16	28937476	*RABEP2*	TSS1500	S_Shore	0.214±0.113	0.413±0.248	-0.199	9.62E-03	9.79E-06	-0.216
cg09903090	6	41910019	*CCND3*	5'UTR	S_Shore	0.174±0.144	0.373±0.2	-0.199	9.94E-03	4.72E-07	-0.245
cg00930606	22	31740944	*PATZ1*	1st exon	N_Shore	0.561±0.086	0.76±0.052	-0.199	1.63E-04	2.33E-10	-0.306
cg12074545	3	44690014	*ZNF35*	TSS1500	N_Shore	0.508±0.14	0.707±0.205	-0.199	9.45E-03	5.56E-14	-0.359
cg27541691	17	40811050	*TUBG2*	TSS1500	N_Shore	0.314±0.134	0.513±0.139	-0.199	3.92E-03	2.22E-05	-0.207
cg04540406	16	46655675	*SHCBP1*	TSS1500	S_Shore	0.328±0.102	0.527±0.214	-0.199	3.81E-03	9.09E-16	-0.382
cg14810385	20	25039658	*ACSS1*	TSS1500	S_Shore	0.475±0.158	0.673±0.166	-0.198	1.12E-02	2.21E-06	-0.231
cg26394257	8	38964993	*ADAM32*	TSS200	N_Shore	0.537±0.124	0.736±0.134	-0.198	2.36E-03	5.31E-08	-0.264
cg19209385	6	53659379	*LRRC1*	TSS1500	Island	0.12±0.068	0.318±0.238	-0.198	3.68E-03	2.74E-09	-0.288
cg13301014	19	38754768	*SPINT2*	TSS1500	N_Shore	0.395±0.162	0.593±0.087	-0.198	9.93E-03	4.88E-17	-0.397
cg15832662	11	63448437	*RTN3*	TSS1500	N_Shore	0.221±0.103	0.419±0.215	-0.198	4.14E-03	1.17E-15	-0.381

cg02075791	10	93174498	*HECTD2*	5'UTR/1st intron	S_Shelf	0.649±0.091	0.847±0.04	-0.198	3.15E-04	1.76E-05	-0.210
cg21234561	12	104697526	*EID3*	5'UTR/1st exon	Island	0.339±0.201	0.536±0.189	-0.198	3.54E-02	5.55E-14	-0.359
cg17922695	17	75451809	*SEPT9*	5'UTR/1st intron	S_Shelf	0.62±0.103	0.818±0.048	-0.198	7.03E-04	2.11E-14	-0.365
cg07090813	1	120254330	*PHGDH*	TSS200	N_Shore	0.254±0.119	0.451±0.161	-0.198	2.69E-03	1.76E-05	-0.210
cg17445812	3	36986805	*TRANK1*	TSS1500	Island	0.462±0.161	0.659±0.098	-0.197	9.90E-03	8.99E-06	-0.217
cg17491622	1	1369616	*VWA1*	TSS1500	N_Shore	0.417±0.098	0.614±0.067	-0.197	4.21E-04	2.98E-10	-0.304
cg00937742	2	133429299	*LYPD1*	TSS1500	S_Shore	0.396±0.157	0.593±0.175	-0.197	1.22E-02	1.12E-16	-0.393
cg24419391	7	73183516	*CLDN3*	1st exon/3'UTR	Island	0.522±0.153	0.72±0.223	-0.197	1.69E-02	5.45E-20	-0.430
cg09456782	13	114146038	*TMCO3*	5'UTR/1st intron	Island	0.392±0.167	0.589±0.214	-0.197	2.14E-02	1.68E-17	-0.403
cg09456782	13	114146038	*DCUN1D2*	TSS1500	Island	0.392±0.167	0.589±0.214	-0.197	2.14E-02	8.03E-06	-0.218
cg25256924	11	67205739	*PTPRCAP*	TSS1500	S_Shelf	0.628±0.126	0.825±0.049	-0.197	2.76E-03	6.12E-57	-0.679
cg00590036	6	158957433	*TMEM181*	TSS200	Island	0.61±0.063	0.807±0.073	-0.197	3.45E-06	1.47E-08	-0.275
cg24811864	18	55019711	*ST8SIA3*	TSS200	Island	0.534±0.073	0.73±0.036	-0.197	6.36E-05	1.01E-09	-0.295
cg26270195	6	33245553	*B3GALT4*	1st exon	Island	0.455±0.107	0.652±0.061	-0.197	8.79E-04	9.08E-22	-0.448
cg06788514	16	88767261	*RNF166*	5'UTR	S_Shelf	0.451±0.085	0.648±0.053	-0.197	1.44E-04	1.58E-11	-0.324
cg16780688	8	120220073	*MAL2*	TSS1500	N_Shore	0.447±0.17	0.644±0.198	-0.197	2.01E-02	7.57E-08	-0.261
cg23442853	3	122296488	*PARP15*	5'UTR/1st exon	N_Shore	0.357±0.14	0.554±0.154	-0.196	5.95E-03	3.08E-20	-0.433
cg02662658	17	34257571	*RDM1*	TSS1500	N_Shore	0.203±0.088	0.4±0.246	-0.196	6.54E-03	1.59E-06	-0.234
cg11541601	8	22437870	*PDLIM2*	TSS200	S_Shore	0.359±0.137	0.555±0.203	-0.196	9.30E-03	1.01E-05	-0.216
cg01850179	19	10340795	*S1PR2*	5'UTR/1st intron	Island	0.262±0.109	0.458±0.214	-0.196	5.16E-03	3.49E-13	-0.348
cg15720832	1	228291682	*C1orf35*	TSS1500	N_Shelf	0.441±0.153	0.637±0.246	-0.196	2.22E-02	3.35E-06	-0.227
cg02943676	7	767474	*PRKAR1B*	TSS1500	S_Shore	0.121±0.082	0.317±0.176	-0.196	7.28E-04	1.73E-08	-0.273
cg19586576	17	42906845	*GJC1*	5'UTR/1st intron	Island	0.347±0.192	0.542±0.178	-0.196	2.96E-02	4.78E-08	-0.265
cg17039022	1	203595145	*ATP2B4*	TSS1500	N_Shelf	0.201±0.151	0.397±0.241	-0.196	2.04E-02	1.01E-05	-0.216
cg18470295	17	8868834	*PIK3R5*	5'UTR/1st intron	Island	0.302±0.101	0.498±0.152	-0.196	9.85E-04	4.77E-12	-0.332
cg18556834	14	24641070	*REC8*	TSS200	Island	0.532±0.066	0.727±0.068	-0.196	7.79E-06	1.76E-09	-0.291
cg11680055	1	110230252	*GSTM1*	TSS200	Island	0.522±0.166	0.718±0.171	-0.196	1.58E-02	1.40E-18	-0.415
cg02313495	15	40398007	*BMF*	1st exon	N_Shelf	0.508±0.048	0.704±0.155	-0.196	6.90E-05	7.50E-09	-0.280
cg13050240	14	24809068	*RIPK3*	5'UTR/1st exon	S_Shore	0.415±0.136	0.611±0.097	-0.195	4.02E-03	1.55E-41	-0.599
cg23092823	1	53528612	*PODN*	5'UTR/1st intron	Island	0.466±0.07	0.661±0.092	-0.195	1.31E-05	4.00E-14	-0.361
cg25371169	8	38757772	*PLEKHA2*	TSS1500	N_Shore	0.422±0.086	0.617±0.168	-0.195	6.84E-04	1.08E-07	-0.258
cg11203985	16	89641054	*CPNE7*	TSS1500	Island	0.406±0.106	0.602±0.12	-0.195	8.19E-04	7.13E-07	-0.241
cg12136772	16	68481813	*SMPD3*	5'UTR/1st intron	Island	0.422±0.098	0.617±0.155	-0.195	8.74E-04	6.48E-07	-0.242
cg27619475	17	73083881	*SLC16A5*	5'UTR/1st exon	Island	0.519±0.084	0.714±0.042	-0.195	1.85E-04	3.82E-27	-0.497
cg23676439	4	155663836	*LRAT*	5'UTR/1st intron	S_Shore	0.291±0.177	0.486±0.213	-0.195	2.67E-02	5.40E-06	-0.222
cg18248284	19	13215151	*LYL1*	TSS1500	N_Shore	0.46±0.067	0.655±0.079	-0.195	8.79E-06	1.66E-10	-0.308
cg13408152	3	120170474	*FSTL1*	TSS1500	Island	0.512±0.074	0.707±0.066	-0.195	4.77E-04	6.63E-06	-0.221
cg26480069	2	172378791	*CYBRD1*	1st exon	N_Shore	0.15±0.129	0.345±0.276	-0.195	2.19E-02	2.33E-08	-0.271
cg08951271	6	30850543	*DDR1*	5'UTR/1st exon	N_Shore	0.564±0.176	0.759±0.131	-0.195	1.75E-02	8.54E-22	-0.449
cg21579399	2	11890700	*LPIN1*	5'UTR/1st intron	S_Shelf	0.479±0.144	0.673±0.119	-0.195	6.09E-03	3.48E-05	-0.202
cg13428480	1	160370208	*VANGL2*	TSS200	Island	0.572±0.123	0.766±0.071	-0.195	2.33E-03	2.47E-17	-0.401
cg11863717	10	73725041	*CHST3*	5'UTR/1st intron	S_Shore	0.312±0.172	0.506±0.259	-0.195	3.51E-02	4.88E-07	-0.245
cg27165884	10	99473085	*MARVELD1*	TSS1500	Island	0.545±0.079	0.74±0.132	-0.195	1.31E-04	7.75E-42	-0.601
cg03108070	6	33245490	*B3GALT4*	1st exon	Island	0.596±0.08	0.79±0.059	-0.194	8.28E-05	1.21E-13	-0.354
cg11600161	11	67171585	*TBC1D10C*	1st exon	S_Shelf	0.492±0.095	0.686±0.065	-0.194	3.57E-04	3.87E-11	-0.318
cg08358392	6	130339410	*L3MBTL3*	TSS1500	N_Shore	0.554±0.116	0.748±0.158	-0.194	2.51E-03	1.03E-11	-0.327
cg08599266	2	25142473	*ADCY3*	5'UTR/1st intron	Island	0.215±0.13	0.409±0.196	-0.194	7.57E-03	2.41E-06	-0.230
cg00505318	3	69130918	*UBA3*	TSS1500	N_Shelf	0.574±0.125	0.767±0.063	-0.194	2.71E-03	3.43E-06	-0.226
cg10961604	1	27709771	*CD164L2*	5'UTR/1st exon	Island	0.468±0.201	0.661±0.228	-0.194	4.59E-02	8.77E-06	-0.217
cg14989191	16	29912071	*SEZ6L2*	TSS1500	N_Shore	0.103±0.022	0.297±0.195	-0.194	6.10E-04	1.44E-09	-0.292
cg14989191	16	29912071	*ASPHD1*	TSS200	N_Shore	0.103±0.022	0.297±0.195	-0.194	6.10E-04	2.72E-07	-0.250
cg07549381	16	88769970	*RNF166*	5'UTR/1st exon	N_Shelf	0.439±0.115	0.633±0.094	-0.194	1.45E-03	1.82E-11	-0.323
cg20681068	20	3389535	*C20orf194*	TSS1500	S_Shore	0.075±0.053	0.268±0.251	-0.193	5.48E-03	1.11E-06	-0.237
cg15128226	4	123747672	*FGF2*	TSS1500	N_Shore	0.544±0.117	0.737±0.149	-0.193	2.42E-03	5.88E-06	-0.221
cg25032595	13	96204978	*CLDN10*	5'UTR/1st exon	Island	0.574±0.107	0.767±0.124	-0.193	1.00E-03	7.11E-08	-0.262
cg23844623	11	47290730	*MADD*	TSS1500	N_Shore	0.121±0.12	0.314±0.196	-0.193	5.62E-03	2.06E-08	-0.272
cg16341836	10	90641389	*STAMBPL1*	5'UTR/1st intron	S_Shore	0.379±0.153	0.573±0.223	-0.193	1.89E-02	1.51E-30	-0.525
cg17082634	15	41850503	*TYRO3*	TSS1500	N_Shore	0.341±0.103	0.534±0.141	-0.193	9.78E-04	3.26E-13	-0.349
cg09459982	6	33386556	*SYNGAP1*	TSS1500	S_Shore	0.571±0.128	0.764±0.057	-0.193	3.26E-03	1.58E-07	-0.255
cg16466899	7	23510693	*IGF2BP3*	TSS1500	S_Shore	0.071±0.026	0.263±0.205	-0.193	9.87E-04	6.13E-07	-0.243
cg15951557	5	137610421	*GFRA3*	TSS200	S_Shore	0.432±0.13	0.625±0.135	-0.193	3.86E-03	1.24E-11	-0.326
cg16321975	2	233924852	*INPP5D*	5'UTR/1st exon	N_Shore	0.381±0.142	0.574±0.155	-0.193	7.43E-03	1.18E-16	-0.393
cg13118545	7	26191599	*NFE2L3*	TSS1500	N_Shore	0.264±0.204	0.457±0.203	-0.193	4.33E-02	1.86E-13	-0.352
cg05723552	16	57026029	*NLRC5*	5'UTR/1st intron	S_Shelf	0.63±0.131	0.823±0.041	-0.193	4.00E-03	1.49E-43	-0.611
cg00927554	12	95941920	*USP44*	5'UTR/1st exon	Island	0.472±0.162	0.665±0.162	-0.193	1.45E-02	9.12E-10	-0.296
cg02102829	10	126849762	*CTBP2*	TSS1500	Island	0.153±0.174	0.345±0.234	-0.192	3.15E-02	4.47E-08	-0.266
cg03465320	6	32823078	*TAP1*	TSS1500	S_Shore	0.389±0.123	0.581±0.197	-0.192	6.43E-03	4.09E-15	-0.374
cg02825887	9	34591041	*CNTFR*	TSS1500	Island	0.248±0.124	0.44±0.217	-0.192	9.27E-03	1.97E-12	-0.337
cg20970369	1	111744108	*DENND2D*	TSS1500	N_Shelf	0.387±0.079	0.579±0.13	-0.192	1.42E-04	2.28E-19	-0.424
cg16990168	1	119526060	*TBX15*	5'UTR/1st intron	N_Shelf	0.597±0.12	0.789±0.072	-0.192	2.15E-03	2.07E-05	-0.208
cg07277869	16	69760850	*NQO1*	TSS1500	S_Shore	0.227±0.114	0.419±0.225	-0.192	8.46E-03	4.95E-25	-0.479
cg22162281	2	207139197	*ZDBF2*	TSS1500	N_Shore	0.395±0.184	0.587±0.216	-0.192	3.40E-02	1.97E-13	-0.352
cg15468095	16	31214401	*PYCARD*	TSS1500	S_Shore	0.115±0.057	0.307±0.124	-0.192	1.57E-05	3.00E-09	-0.287
cg15068733	17	7342846	*FGF11*	5'UTR/1st exon	Island	0.341±0.206	0.533±0.221	-0.192	4.97E-02	9.81E-10	-0.295
cg24917382	11	2160953	*IGF2*	5'UTR	Island	0.553±0.081	0.745±0.058	-0.192	1.09E-04	2.23E-20	-0.434
cg03703356	14	103989368	*CKB*	TSS200	Island	0.242±0.088	0.434±0.223	-0.191	4.55E-03	6.60E-06	-0.220
cg18404652	8	42009622	*AP3M2*	TSS1500	N_Shore	0.604±0.102	0.795±0.08	-0.191	6.74E-04	3.14E-05	-0.204
cg20486569	22	19512228	*CLDN5*	5'UTR/1st exon	Island	0.468±0.088	0.659±0.177	-0.191	1.26E-03	1.08E-05	-0.215
cg19295951	15	45421998	*DUOX1*	TSS200	Island	0.622±0.086	0.813±0.059	-0.191	1.96E-04	1.50E-06	-0.234
cg19295951	15	45421998	*DUOXA1*	5'UTR/1st exon	Island	0.622±0.086	0.813±0.059	-0.191	1.96E-04	1.86E-06	-0.232
cg03461967	17	27896173	*TP53I13*	5'UTR	S_Shore	0.351±0.141	0.542±0.196	-0.191	1.13E-02	1.93E-28	-0.508
cg18456459	18	9707753	*RAB31*	TSS1500	Island	0.513±0.164	0.704±0.111	-0.191	1.35E-02	2.69E-17	-0.400
cg20358834	11	66624256	*LRFN4*	TSS1500	Island	0.486±0.131	0.677±0.122	-0.191	4.03E-03	1.64E-20	-0.436
cg04075973	11	118781608	*BCL9L*	5'UTR/1st exon	Island	0.179±0.094	0.37±0.192	-0.191	2.44E-03	9.38E-08	-0.259
cg16087457	17	73893198	*TRIM65*	TSS200	Island	0.185±0.149	0.376±0.184	-0.191	1.26E-02	3.30E-17	-0.399
cg06873218	22	39637727	*PDGFB*	TSS1500	N_Shore	0.453±0.095	0.644±0.199	-0.191	2.98E-03	6.27E-11	-0.315
cg20465207	1	155948698	*ARHGEF2*	5'UTR/1st intron	S_Shore	0.182±0.175	0.372±0.202	-0.191	2.68E-02	7.14E-36	-0.563

cg02442640	17	75451779	*SEPT9*	5'UTR/1st intron	S_Shelf	0.599±0.096	0.789±0.049	-0.191	5.59E-04	7.05E-15	-0.371
cg24603490	12	47473995	*AMIGO2*	TSS1500	Island	0.189±0.124	0.379±0.222	-0.191	1.05E-02	1.43E-16	-0.392
cg02696067	6	30850977	*DDR1*	5'UTR/1st intron	N_Shore	0.533±0.167	0.724±0.123	-0.191	1.51E-02	5.79E-24	-0.469
cg11547273	4	44728844	*GNPDA2*	TSS200	S_Shore	0.222±0.13	0.413±0.243	-0.191	1.62E-02	1.29E-05	-0.213
cg23631538	1	111743537	*DENND2D*	TSS1500	N_Shelf	0.372±0.115	0.562±0.157	-0.190	2.72E-03	2.83E-14	-0.363
cg23778596	7	149411498	*KRBA1*	TSS1500	Island	0.326±0.215	0.516±0.143	-0.190	4.54E-02	4.02E-11	-0.318
cg19371526	11	2161341	*IGF2*	5'UTR	Island	0.499±0.085	0.689±0.077	-0.190	1.33E-04	8.13E-16	-0.383
cg22166739	11	73694868	*UCP2*	TSS1500	S_Shore	0.333±0.1	0.523±0.16	-0.190	1.41E-03	4.22E-27	-0.497
cg01800926	1	9884565	*CLSTN1*	5'UTR/1st exon	Island	0.16±0.073	0.351±0.234	-0.190	4.86E-03	2.30E-05	-0.207
cg07083464	7	27240758	*HOXA13*	TSS1500	N_Shelf	0.239±0.138	0.429±0.253	-0.190	2.16E-02	5.29E-06	-0.222
cg17394649	6	29760164	*HCG4*	1st exon	Island	0.146±0.15	0.336±0.317	-0.190	4.88E-02	4.32E-12	-0.332
cg11915650	11	2161009	*IGF2*	5'UTR	Island	0.475±0.115	0.665±0.104	-0.190	1.64E-03	2.34E-15	-0.377
cg04879387	16	16043101	*ABCC1*	TSS1500	N_Shore	0.138±0.086	0.328±0.167	-0.190	8.67E-04	6.92E-06	-0.219
cg03616357	4	156297942	*MAP9*	5'UTR/1st exon	Island	0.335±0.172	0.525±0.14	-0.190	1.84E-02	1.29E-14	-0.367
cg00844376	10	99473557	*MARVELD1*	5'UTR/1st exon	S_Shore	0.135±0.123	0.325±0.184	-0.190	8.33E-03	3.69E-09	-0.285
cg01466741	5	134733519	*H2AFY*	5'UTR/1st intron	N_Shore	0.338±0.121	0.528±0.18	-0.190	5.07E-03	7.30E-23	-0.459
cg15728256	1	40254184	*BMP8B*	5'UTR/1st exon	Island	0.418±0.15	0.608±0.159	-0.190	1.09E-02	1.66E-13	-0.353
cg06147863	11	47400113	*SPI1*	5'UTR/1st exon	S_Shore	0.512±0.108	0.702±0.103	-0.190	1.03E-03	1.67E-35	-0.561
cg14415844	14	91884659	*CCDC88C*	TSS1500	S_Shore	0.208±0.145	0.398±0.247	-0.190	2.31E-02	1.00E-28	-0.511
cg08469255	6	30851069	*DDR1*	5'UTR/1st intron	N_Shore	0.679±0.201	0.869±0.032	-0.190	3.24E-02	2.36E-43	-0.610
cg18660898	1	151030968	*CDC42SE1*	5'UTR/1st intron	N_Shore	0.348±0.16	0.538±0.206	-0.189	2.10E-02	5.19E-17	-0.397
cg18660898	1	151030968	*MLLT11*	TSS1500	N_Shore	0.348±0.16	0.538±0.206	-0.189	2.10E-02	2.24E-05	-0.207
cg02388253	9	123640363	*PHF19*	TSS1500	S_Shore	0.474±0.131	0.664±0.148	-0.189	5.19E-03	7.69E-16	-0.383
cg20822579	14	24809073	*RIPK3*	5'UTR/1st exon	S_Shore	0.436±0.133	0.625±0.078	-0.189	4.36E-03	1.63E-43	-0.611
cg04305621	17	73084263	*SLC16A5*	5'UTR/1st intron	Island	0.535±0.079	0.725±0.041	-0.189	1.46E-04	3.14E-31	-0.530
cg19021197	17	59476212	*TBX2*	TSS1500	Island	0.405±0.127	0.594±0.111	-0.189	3.30E-03	3.30E-07	-0.248
cg17081976	2	24347010	*PFN4*	TSS1500	S_Shore	0.304±0.135	0.493±0.218	-0.189	1.34E-02	8.40E-14	-0.357
cg08641278	10	73848764	*SPOCK2*	5'UTR/1st exon	S_Shore	0.399±0.121	0.588±0.17	-0.189	4.47E-03	4.16E-05	-0.200
cg03387723	1	41708464	*SCMH1*	TSS1500	S_Shore	0.623±0.149	0.812±0.098	-0.189	8.38E-03	6.20E-09	-0.281
cg04811945	4	15779709	*CD38*	TSS200	N_Shore	0.572±0.129	0.761±0.144	-0.189	4.60E-03	2.35E-17	-0.401
cg02652579	6	33386967	*SYNGAP1*	TSS1500	S_Shore	0.624±0.137	0.813±0.091	-0.189	5.21E-03	4.23E-10	-0.301
cg16483840	6	32812953	*PSMB8*	TSS1500	S_Shore	0.427±0.122	0.616±0.138	-0.189	3.21E-03	6.37E-26	-0.487
cg10856045	10	73848463	*SPOCK2*	5'UTR/1st exon	S_Shore	0.308±0.102	0.497±0.147	-0.189	1.27E-03	1.89E-05	-0.209
cg12120359	14	65006041	*HSPA2*	TSS1500	N_Shore	0.597±0.166	0.786±0.111	-0.189	1.50E-02	4.19E-06	-0.225
cg07338698	17	79895532	*PYCR1*	TSS1500	N_Shelf	0.186±0.157	0.375±0.221	-0.189	3.11E-02	1.25E-11	-0.328
cg02048416	2	74782684	*DOK1*	5'UTR	Island	0.439±0.088	0.627±0.162	-0.189	8.85E-04	1.18E-10	-0.311
cg18741908	3	169756818	*GPR160*	5'UTR	Island	0.615±0.082	0.804±0.092	-0.189	1.06E-04	2.20E-05	-0.207
cg26648103	11	66791718	*SYT12*	5'UTR/1st intron	S_Shore	0.292±0.087	0.481±0.137	-0.189	3.95E-04	3.23E-23	-0.462
cg17317439	19	12075960	*ZNF763*	5'UTR/1st exon	N_Shore	0.344±0.102	0.533±0.237	-0.189	9.24E-03	4.26E-32	-0.537
cg05871694	2	177053503	*HOXD1*	5'UTR/1st exon	Island	0.357±0.105	0.545±0.214	-0.189	6.21E-03	9.43E-07	-0.239
cg26886572	11	3009206	*NAP1L4*	5'UTR/1st intron	N_Shelf	0.594±0.107	0.783±0.139	-0.189	1.50E-03	8.76E-07	-0.239
cg00759427	20	62708630	*RGS19*	5'UTR/1st intron	N_Shelf	0.598±0.098	0.786±0.056	-0.188	6.63E-04	2.33E-08	-0.271
cg03529003	1	11994281	*PLOD1*	TSS1500	N_Shore	0.397±0.117	0.586±0.18	-0.188	4.66E-03	9.67E-06	-0.216
cg15604242	19	11450739	*RAB3D*	TSS1500	S_Shore	0.639±0.054	0.827±0.06	-0.188	1.05E-06	2.20E-26	-0.491
cg08352439	7	55637123	*VOPP1*	5'UTR/1st intron	N_Shelf	0.497±0.137	0.685±0.119	-0.188	5.70E-03	8.97E-11	-0.312
cg05661139	19	20011721	*ZNF93*	5'UTR/1st exon	N_Shore	0.134±0.143	0.322±0.267	-0.188	2.89E-02	9.70E-08	-0.259
cg07869548	16	47177603	*NETO2*	5'UTR/1st exon	Island	0.327±0.081	0.515±0.114	-0.188	1.39E-04	1.59E-11	-0.324
cg14825555	1	27190639	*SFN*	1st exon/3'UTR	S_Shore	0.449±0.137	0.637±0.159	-0.188	7.67E-03	4.17E-30	-0.521
cg00760992	12	50223054	*NCKAP5L*	TSS1500	S_Shore	0.491±0.161	0.679±0.145	-0.188	1.51E-02	4.14E-16	-0.386
cg08710629	16	87903292	*SLC7A5*	TSS200	Island	0.471±0.122	0.658±0.068	-0.188	2.75E-03	1.52E-09	-0.292
cg10037494	11	2161135	*IGF2*	5'UTR	Island	0.614±0.08	0.801±0.068	-0.188	9.87E-05	7.74E-21	-0.439
cg12870750	17	7791882	*CHD3*	TSS1500	S_Shelf	0.562±0.101	0.749±0.054	-0.188	8.67E-04	1.42E-05	-0.212
cg27366766	17	56565286	*HSF5*	1st exon	Island	0.708±0.08	0.895±0.05	-0.187	1.36E-04	3.80E-06	-0.225
cg10542975	11	67205096	*PTPRCAP*	5'UTR/1st exon	S_Shore	0.699±0.102	0.887±0.034	-0.187	1.10E-03	2.26E-52	-0.658
cg23175055	6	31704738	*CLIC1*	5'UTR/1st exon	N_Shelf	0.236±0.078	0.423±0.214	-0.187	3.37E-03	2.54E-16	-0.389
cg13516746	20	3389408	*C20orf194*	TSS1500	S_Shore	0.081±0.068	0.269±0.262	-0.187	9.89E-03	9.32E-06	-0.216
cg17199483	16	16042767	*ABCC1*	TSS1500	N_Shore	0.346±0.135	0.533±0.177	-0.187	8.73E-03	2.65E-10	-0.305
cg07639376	16	1584516	*TMEM204*	1st exon	Island	0.624±0.103	0.812±0.054	-0.187	9.47E-04	1.01E-12	-0.342
cg07341220	16	1583899	*TMEM204*	5'UTR/1st exon	Island	0.564±0.106	0.751±0.055	-0.187	1.17E-03	2.98E-10	-0.304
cg23114616	1	6526398	*TNFRSF25*	TSS200	S_Shore	0.551±0.099	0.738±0.149	-0.187	1.22E-03	1.70E-17	-0.403
cg13966383	17	73893209	*TRIM65*	TSS200	Island	0.292±0.094	0.479±0.134	-0.187	6.41E-04	5.15E-15	-0.373
cg13026729	6	159240774	*EZR*	TSS1500	Island	0.405±0.122	0.592±0.202	-0.187	8.13E-03	5.55E-14	-0.359
cg03702686	6	33244172	*B3GALT4*	TSS1500	N_Shore	0.654±0.074	0.841±0.039	-0.187	9.00E-05	9.82E-18	-0.405
cg09053081	20	44746392	*CD40*	TSS1500	N_Shore	0.076±0.072	0.263±0.262	-0.187	1.05E-02	9.41E-11	-0.312
cg17780956	4	156297616	*MAP9*	5'UTR/1st intron	Island	0.263±0.189	0.45±0.219	-0.187	4.21E-02	2.47E-10	-0.305
cg23030069	11	2162183	*IGF2*	5'UTR	Island	0.622±0.102	0.809±0.092	-0.187	7.63E-04	6.98E-11	-0.314
cg17518965	19	3178955	*S1PR4*	1st exon	Island	0.564±0.08	0.751±0.079	-0.187	9.13E-05	1.10E-27	-0.502
cg00499237	2	97169033	*NEURL3*	5'UTR	S_Shelf	0.657±0.187	0.843±0.044	-0.187	2.58E-02	5.23E-23	-0.460
cg15319451	1	15480748	*TMEM51*	5'UTR/1st intron	Island	0.196±0.125	0.383±0.249	-0.187	1.81E-02	1.01E-06	-0.238
cg06583813	20	62710295	*RGS19*	5'UTR/1st intron	N_Shore	0.194±0.124	0.381±0.123	-0.186	3.47E-03	3.04E-11	-0.320
cg06583813	20	62710295	*OPRL1*	TSS1500	N_Shore	0.194±0.124	0.381±0.123	-0.186	3.47E-03	3.06E-09	-0.287
cg17658002	19	11689997	*ACP5*	TSS1500	S_Shore	0.63±0.101	0.817±0.046	-0.186	9.30E-04	2.08E-18	-0.413
cg22338446	19	42390475	*ARHGEF1*	5'UTR/1st intron	S_Shore	0.547±0.105	0.733±0.081	-0.186	9.63E-04	5.33E-12	-0.331
cg10021349	19	49436805	*DHDH*	TSS200	Island	0.215±0.191	0.401±0.209	-0.186	4.17E-02	1.18E-05	-0.214
cg01595870	6	144386416	*PLAGL1*	TSS1500	S_Shore	0.653±0.098	0.839±0.059	-0.186	6.47E-04	1.99E-07	-0.253
cg05079794	7	128050616	*IMPDH1*	TSS1500	S_Shore	0.132±0.076	0.318±0.149	-0.186	3.33E-04	9.46E-08	-0.259
cg10465839	16	1584050	*TMEM204*	5'UTR	Island	0.533±0.102	0.719±0.043	-0.186	1.03E-03	7.10E-07	-0.241
cg02900356	16	31214494	*PYCARD*	TSS1500	S_Shore	0.212±0.05	0.398±0.146	-0.186	7.65E-05	2.13E-14	-0.365
cg21730993	6	86159210	*NT5E*	TSS200	N_Shore	0.155±0.14	0.341±0.28	-0.186	3.39E-02	1.08E-07	-0.258
cg00554413	1	59042113	*TACSTD2*	1st exon	Island	0.428±0.106	0.613±0.121	-0.186	1.22E-03	1.98E-07	-0.253
cg12405136	11	68781357	*MRGPRF*	TSS1500	S_Shelf	0.562±0.097	0.747±0.067	-0.186	5.78E-04	7.68E-08	-0.261
cg19366591	1	92951355	*GFI1*	5'UTR/1st intron	Island	0.323±0.17	0.509±0.192	-0.186	2.60E-02	1.70E-05	-0.210
cg10064339	11	73693792	*UCP2*	5'UTR/1st exon	Island	0.562±0.121	0.748±0.083	-0.186	2.77E-03	5.12E-10	-0.300
cg22522066	19	38754726	*SPINT2*	TSS1500	N_Shore	0.31±0.147	0.496±0.167	-0.186	1.21E-02	3.90E-19	-0.421
cg18741372	19	45906156	*PPP1R13L*	5'UTR/1st intron	N_Shelf	0.517±0.031	0.703±0.086	-0.185	3.50E-08	6.87E-08	-0.262
cg02017926	12	123754328	*CDK2AP1*	5'UTR/1st intron	N_Shore	0.639±0.088	0.824±0.039	-0.185	3.84E-04	8.54E-17	-0.395
cg13267747	5	141031176	*FCHSD1*	TSS200	Island	0.318±0.163	0.504±0.214	-0.185	2.65E-02	2.71E-22	-0.454

cg22184210	15	57883722	*GCOM1*	TSS1500	N_Shore	0.555±0.07	0.74±0.127	-0.185	8.35E-05	4.55E-17	-0.398
cg11394785	5	179221090	*LTC4S*	1st exon	N_Shore	0.538±0.082	0.723±0.037	-0.185	2.40E-04	7.30E-11	-0.314
cg07856138	16	29673465	*SPN*	TSS1500	N_Shelf	0.383±0.141	0.568±0.179	-0.185	1.13E-02	8.62E-09	-0.279
cg19024700	19	49340765	*HSD17B14*	TSS1500	Island	0.504±0.139	0.689±0.17	-0.185	1.00E-02	1.41E-14	-0.367
cg07871590	7	127671193	*LRRC4*	TSS200	Island	0.558±0.07	0.743±0.052	-0.185	4.19E-05	6.70E-21	-0.440
cg07571745	1	32715428	*LCK*	TSS1500	S_Shore	0.582±0.104	0.767±0.049	-0.185	1.20E-03	3.11E-28	-0.507
cg19796273	5	16936868	*MYO10*	TSS1500	S_Shore	0.226±0.128	0.411±0.172	-0.185	7.06E-03	3.96E-08	-0.267
cg06602086	16	1583883	*TMEM204*	5'UTR/1st exon	Island	0.624±0.086	0.809±0.049	-0.185	2.80E-04	3.86E-13	-0.348
cg26534477	17	42147600	*G6PC3*	TSS1500	N_Shore	0.57±0.066	0.754±0.052	-0.185	2.11E-05	2.10E-07	-0.252
cg19975933	11	74022477	*P4HA3*	1st exon	Island	0.412±0.1	0.597±0.13	-0.185	1.03E-03	2.94E-05	-0.204
cg06285727	11	72524028	*ATG16L2*	TSS1500	N_Shore	0.311±0.168	0.495±0.151	-0.185	2.02E-02	1.72E-05	-0.210
cg05180856	15	91428056	*FES*	5'UTR/1st intron	S_Shelf	0.509±0.112	0.693±0.099	-0.185	1.63E-03	1.17E-43	-0.613
cg09694722	11	2161318	*IGF2*	5'UTR	Island	0.397±0.061	0.581±0.104	-0.185	1.17E-05	1.21E-11	-0.326
cg16107628	1	159894061	*TAGLN2*	5'UTR/1st intron	N_Shore	0.215±0.097	0.399±0.231	-0.185	8.54E-03	3.04E-35	-0.559
cg19350469	4	15704453	*BST1*	TSS200	N_Shore	0.578±0.119	0.763±0.093	-0.185	2.51E-03	2.64E-46	-0.627
cg23524290	19	10542131	*PDE4A*	TSS1500	N_Shore	0.26±0.079	0.444±0.187	-0.184	1.72E-03	1.63E-15	-0.379
cg07054208	6	24358566	*DCDC2*	5'UTR/1st intron	N_Shore	0.454±0.182	0.639±0.239	-0.184	4.52E-02	1.51E-07	-0.255
cg08866634	5	175962338	*RNF44*	5'UTR/1st intron	N_Shore	0.471±0.154	0.655±0.168	-0.184	1.56E-02	2.22E-16	-0.390
cg13816999	11	12398883	*PARVA*	TSS1500	N_Shore	0.431±0.108	0.615±0.172	-0.184	3.36E-03	8.52E-08	-0.260
cg10142237	22	38714395	*CSNK1E*	TSS1500	S_Shore	0.569±0.106	0.753±0.044	-0.184	1.42E-03	7.24E-20	-0.429
cg13412498	9	36037340	*RECK*	5'UTR/1st intron	Island	0.234±0.163	0.419±0.263	-0.184	4.13E-02	5.21E-21	-0.441
cg22545138	7	23510005	*IGF2BP3*	TSS200	S_Shore	0.153±0.052	0.338±0.19	-0.184	9.92E-04	1.27E-09	-0.293
cg20294304	6	34203153	*HMGA1*	TSS1500	Island	0.624±0.085	0.808±0.06	-0.184	2.19E-04	1.27E-08	-0.276
cg19474029	19	35491595	*GRAMD1A*	5'UTR/1st intron	Island	0.023±0.033	0.207±0.181	-0.184	5.17E-04	4.74E-09	-0.283
cg14018690	3	111804832	*C3orf52*	TSS1500	N_Shore	0.463±0.123	0.647±0.15	-0.184	4.49E-03	2.90E-09	-0.287
cg10500147	12	6881601	*LAG3*	TSS200	N_Shore	0.375±0.135	0.559±0.138	-0.184	6.91E-03	3.73E-10	-0.302
cg03302259	19	11689855	*ACP5*	TSS1500	S_Shore	0.493±0.102	0.677±0.196	-0.184	4.55E-03	2.53E-25	-0.482
cg04529938	17	73084494	*SLC16A5*	5'UTR/1st intron	Island	0.595±0.09	0.779±0.035	-0.184	5.44E-04	2.50E-34	-0.553
cg08793208	16	66585449	*CKLF*	TSS1500	N_Shore	0.16±0.052	0.343±0.138	-0.184	5.45E-05	6.13E-16	-0.384
cg19348484	15	91413236	*FURIN*	5'UTR/1st intron	N_Shore	0.581±0.105	0.765±0.053	-0.183	1.26E-03	4.23E-17	-0.398
cg17213699	12	6881624	*LAG3*	TSS200	N_Shore	0.59±0.138	0.774±0.098	-0.183	6.58E-03	1.91E-12	-0.338
cg05729480	17	75276428	*SEPT9*	TSS1500	N_Shore	0.493±0.158	0.676±0.106	-0.183	1.36E-02	2.90E-18	-0.411
cg19994779	14	105482622	*CDCA4*	5'UTR/1st intron	N_Shelf	0.508±0.084	0.691±0.09	-0.183	1.67E-04	3.78E-12	-0.333
cg17024258	1	160370982	*VANGL2*	5'UTR/1st intron	S_Shore	0.208±0.065	0.391±0.164	-0.183	4.46E-04	2.39E-06	-0.230
cg17078116	8	24772344	*NEFM*	1st exon	Island	0.507±0.132	0.69±0.11	-0.183	5.40E-03	1.84E-07	-0.253
cg11230716	19	49436812	*DHDH*	TSS200	Island	0.192±0.173	0.375±0.214	-0.183	3.40E-02	9.79E-06	-0.216
cg15149596	11	19138954	*ZDHHC13*	5'UTR/1st intron	Island	0.157±0.08	0.34±0.286	-0.183	2.02E-02	3.32E-07	-0.248
cg05203776	11	2162582	*IGF2*	5'UTR	S_Shore	0.5±0.057	0.683±0.154	-0.183	1.93E-04	2.88E-08	-0.269
cg19878987	12	21810999	*LDHB*	TSS1500	S_Shore	0.51±0.117	0.693±0.097	-0.183	2.49E-03	3.63E-17	-0.399
cg06133110	7	30028307	*SCRN1*	5'UTR/1st intron	N_Shore	0.511±0.106	0.694±0.162	-0.182	2.71E-03	2.38E-13	-0.350
cg01073837	8	38757859	*PLEKHA2*	TSS1500	N_Shore	0.413±0.136	0.595±0.183	-0.182	1.12E-02	1.02E-05	-0.216
cg06330618	15	91428456	*FES*	1st exon	S_Shelf	0.541±0.08	0.723±0.081	-0.182	1.16E-04	1.03E-37	-0.576
cg17283781	6	52226707	*PAQR8*	TSS1500	N_Shore	0.471±0.147	0.653±0.123	-0.182	1.02E-02	2.25E-10	-0.306
cg24484339	8	86157525	*CA13*	TSS200	N_Shore	0.166±0.101	0.348±0.261	-0.182	1.73E-02	3.48E-05	-0.202
cg03432736	4	10119609	*WDR1*	TSS1500	S_Shore	0.53±0.079	0.712±0.086	-0.182	9.47E-05	2.20E-09	-0.289
cg02740606	11	67206418	*PTPRCAP*	TSS1500	S_Shelf	0.731±0.095	0.913±0.045	-0.182	7.20E-04	3.84E-47	-0.631
cg12897947	17	76125152	*TMC6*	5'UTR/1st intron	N_Shelf	0.131±0.108	0.313±0.246	-0.182	1.49E-02	1.60E-11	-0.324
cg03883348	19	45843826	*KLC3*	TSS200	N_Shore	0.394±0.128	0.576±0.191	-0.182	9.85E-03	5.89E-08	-0.263
cg19168338	16	4465731	*CORO7*	5'UTR/1st exon	N_Shore	0.26±0.104	0.442±0.186	-0.182	4.27E-03	1.59E-16	-0.391
cg09884451	4	2366831	*ZFYVE28*	TSS1500	Island	0.549±0.048	0.731±0.136	-0.182	4.28E-05	7.17E-12	-0.329
cg25429672	11	73692155	*UCP2*	5'UTR	N_Shore	0.571±0.121	0.753±0.11	-0.182	3.30E-03	4.01E-28	-0.506
cg03297007	12	123450869	*ABCB9*	5'UTR/1st exon	Island	0.12±0.095	0.301±0.201	-0.182	4.56E-03	7.89E-11	-0.313
cg14299675	9	91149046	*NXNL2*	TSS1500	N_Shore	0.374±0.177	0.556±0.204	-0.182	3.57E-02	3.56E-05	-0.202
cg04653522	11	87908631	*RAB38*	5'UTR/1st exon	S_Shore	0.312±0.142	0.494±0.191	-0.182	1.48E-02	8.27E-11	-0.313
cg00313981	1	228595062	*TRIM11*	TSS1500	S_Shore	0.195±0.087	0.377±0.228	-0.182	7.40E-03	2.31E-06	-0.230
cg16473141	14	24641501	*REC8*	5'UTR/1st exon	Island	0.622±0.082	0.803±0.042	-0.181	2.57E-04	1.10E-11	-0.326
cg13401339	2	24272928	*FKBP1B*	5'UTR/1st intron	Island	0.046±0.057	0.227±0.178	-0.181	7.43E-04	1.26E-08	-0.276
cg04539574	5	16936819	*MYO10*	TSS1500	S_Shore	0.11±0.068	0.291±0.144	-0.181	2.13E-04	1.28E-06	-0.236
cg03088990	20	44540278	*PLTP*	5'UTR/1st intron	N_Shore	0.439±0.104	0.62±0.087	-0.181	1.15E-03	1.01E-11	-0.327
cg04599026	7	96634763	*DLX6*	TSS1500	Island	0.564±0.089	0.745±0.192	-0.181	3.16E-03	9.72E-17	-0.394
cg14273126	19	859499	*CFD*	TSS200	N_Shelf	0.436±0.085	0.617±0.159	-0.181	1.01E-03	1.75E-05	-0.210
cg22706106	1	153607701	*S100A13*	TSS1500	S_Shore	0.371±0.143	0.551±0.172	-0.181	1.31E-02	1.82E-05	-0.209
cg17219326	3	44517846	*ZNF445*	5'UTR/1st intron	N_Shore	0.159±0.112	0.34±0.198	-0.181	7.20E-03	1.08E-09	-0.295
cg20622019	20	43279793	*ADA*	5'UTR/1st intron	N_Shore	0.453±0.137	0.634±0.138	-0.181	8.07E-03	3.49E-19	-0.422
cg13477101	7	1067302	*C7orf50*	5'UTR/1st intron	Island	0.564±0.104	0.744±0.06	-0.181	1.28E-03	1.33E-05	-0.213
cg27262415	11	67205166	*PTPRCAP*	TSS200	S_Shore	0.517±0.095	0.698±0.055	-0.180	6.60E-04	4.01E-28	-0.506
cg10815400	1	65210420	*RAVER2*	TSS1500	Island	0.13±0.091	0.311±0.222	-0.180	7.20E-03	2.09E-10	-0.306
cg09728392	16	66585799	*CKLF*	TSS1500	N_Shore	0.547±0.109	0.727±0.101	-0.180	1.61E-03	4.22E-16	-0.386
cg01016800	10	97802959	*CCNJ*	TSS200	Island	0.479±0.17	0.66±0.111	-0.180	2.08E-02	6.05E-26	-0.487
cg17084361	4	85503668	*CDS1*	TSS1500	Island	0.23±0.181	0.41±0.203	-0.180	3.97E-02	3.23E-06	-0.227
cg01733438	17	75276069	*SEPT9*	TSS1500	N_Shore	0.564±0.159	0.744±0.118	-0.180	1.60E-02	5.32E-22	-0.451
cg15393937	11	2161383	*IGF2*	5'UTR	Island	0.618±0.085	0.798±0.07	-0.180	2.28E-04	5.73E-21	-0.440
cg24517501	1	92952702	*GFI1*	TSS1500	S_Shore	0.663±0.097	0.843±0.071	-0.180	7.44E-04	1.85E-32	-0.539
cg02166532	11	2159853	*IGF2*	5'UTR	Island	0.265±0.084	0.445±0.144	-0.180	6.04E-04	4.33E-16	-0.386
cg01441777	22	38714416	*CSNK1E*	TSS1500	S_Shore	0.552±0.09	0.732±0.049	-0.180	5.00E-04	2.41E-18	-0.412
cg15679651	19	39108529	*MAP4K1*	1st exon	N_Shore	0.434±0.104	0.614±0.103	-0.180	1.27E-03	3.22E-15	-0.375
cg19854901	3	50376216	*RASSF1*	5'UTR/1st intron	S_Shore	0.681±0.099	0.86±0.027	-0.180	1.20E-03	8.49E-06	-0.217
cg17610931	22	31476670	*SMTN*	TSS1500	N_Shore	0.407±0.077	0.586±0.17	-0.180	1.10E-03	1.83E-07	-0.254
cg04597449	5	94955575	*GPR150*	TSS1500	N_Shore	0.486±0.139	0.665±0.198	-0.179	1.60E-02	1.22E-05	-0.214
cg01167556	12	56323639	*DGKA*	TSS1500	S_Shelf	0.505±0.149	0.684±0.162	-0.179	1.51E-02	1.04E-15	-0.381
cg21717369	6	8103092	*EEF1E1*	TSS1500	S_Shore	0.377±0.162	0.556±0.225	-0.179	3.33E-02	2.13E-06	-0.231
cg18512948	14	24641706	*REC8*	5'UTR	Island	0.654±0.08	0.833±0.039	-0.179	2.51E-04	5.63E-13	-0.345
cg12717594	9	36037454	*RECK*	5'UTR/1st intron	Island	0.218±0.162	0.397±0.237	-0.179	3.70E-02	3.83E-24	-0.471
cg12273325	22	31689831	*PIK3IP1*	TSS1500	S_Shelf	0.507±0.153	0.686±0.09	-0.179	1.28E-02	5.99E-11	-0.315
cg06434451	1	32802291	*MARCKSL1*	TSS1500	S_Shore	0.599±0.131	0.778±0.085	-0.179	5.49E-03	9.54E-12	-0.327
cg18396533	10	82116208	*DYDC1*	5'UTR/1st intron	Island	0.577±0.09	0.756±0.032	-0.179	6.31E-04	2.70E-07	-0.250
cg26870803	11	66624853	*LRFN4*	5'UTR/1st intron	Island	0.468±0.15	0.647±0.162	-0.179	1.55E-02	1.28E-17	-0.404

cg14614490	9	123637816	*PHF19*	5'UTR/1st intron	N_Shore	0.62±0.062	0.799±0.045	-0.179	1.91E-05	5.78E-22	-0.450
cg00556029	1	32802033	*MARCKSL1*	TSS200	Island	0.215±0.123	0.394±0.168	-0.179	6.80E-03	1.33E-12	-0.340
cg00220455	11	67171191	*TBC1D10C*	TSS1500	S_Shelf	0.529±0.101	0.708±0.047	-0.179	1.19E-03	9.81E-16	-0.382
cg14254748	1	62660421	*L1TD1*	TSS200	N_Shore	0.612±0.203	0.791±0.071	-0.179	4.19E-02	5.02E-07	-0.245
cg12419195	2	191272731	*MFSD6*	TSS1500	Island	0.036±0.009	0.215±0.26	-0.179	9.55E-03	9.47E-09	-0.278
cg12650926	6	33589118	*ITPR3*	TSS200	Island	0.143±0.11	0.322±0.217	-0.179	1.05E-02	8.76E-06	-0.217
cg22356324	10	71077947	*HK1*	5'UTR/1st exon	Island	0.523±0.112	0.702±0.143	-0.179	3.10E-03	2.33E-11	-0.321
cg18684951	5	141700490	*SPRY4*	5'UTR/1st intron	N_Shelf	0.403±0.065	0.582±0.147	-0.179	2.40E-04	1.01E-11	-0.327
cg12191938	10	88730946	*AGAP11*	5'UTR/1st intron	S_Shelf	0.58±0.118	0.759±0.12	-0.179	3.29E-03	1.92E-09	-0.290
cg02039987	2	157291262	*GPD2*	TSS1500	N_Shore	0.585±0.066	0.764±0.114	-0.179	4.87E-05	6.64E-15	-0.371
cg16260696	11	67171200	*TBC1D10C*	TSS1500	S_Shelf	0.542±0.103	0.721±0.079	-0.179	1.14E-03	1.13E-17	-0.405
cg03929741	13	107187681	*EFNB2*	TSS1500	Island	0.477±0.163	0.656±0.17	-0.179	2.33E-02	1.08E-05	-0.215
cg23655039	3	182972742	*B3GNT5*	5'UTR/1st intron	S_Shore	0.412±0.122	0.591±0.151	-0.179	5.52E-03	1.21E-28	-0.510
cg20931541	6	144386528	*PLAGL1*	TSS1500	S_Shore	0.657±0.113	0.836±0.117	-0.178	2.49E-03	1.57E-05	-0.211
cg05877528	22	26875552	*HPS4*	1st exon	N_Shelf	0.329±0.121	0.507±0.219	-0.178	1.41E-02	3.31E-05	-0.203
cg19154438	19	45825730	*CKM*	5'UTR/1st intron	N_Shore	0.607±0.139	0.785±0.132	-0.178	9.18E-03	9.15E-09	-0.278
cg03584056	19	4910798	*UHRF1*	5'UTR/1st exon	S_Shore	0.292±0.107	0.47±0.115	-0.178	1.74E-03	5.62E-16	-0.385
cg20038493	14	24809226	*RIPK3*	5'UTR/1st exon	S_Shore	0.416±0.144	0.594±0.133	-0.178	1.10E-02	2.79E-37	-0.573
cg25599269	19	12075882	*ZNF763*	5'UTR/1st exon	N_Shore	0.327±0.142	0.505±0.266	-0.178	3.66E-02	3.18E-21	-0.443
cg10106091	12	117176294	*RNFT2*	5'UTR/1st intron	Island	0.14±0.133	0.318±0.238	-0.178	2.33E-02	5.84E-08	-0.263
cg10106091	12	117176294	*C12orf49*	TSS1500	Island	0.14±0.133	0.318±0.238	-0.178	2.33E-02	1.53E-06	-0.234
cg02481294	4	15704550	*BST1*	TSS200	N_Shore	0.507±0.115	0.685±0.072	-0.178	2.63E-03	3.34E-29	-0.514
cg10137231	14	24641021	*REC8*	TSS1500	N_Shore	0.656±0.074	0.834±0.052	-0.178	1.02E-04	4.56E-12	-0.332
cg17397004	22	38863644	*KDELR3*	TSS1500	N_Shore	0.527±0.157	0.705±0.181	-0.178	2.24E-02	1.62E-14	-0.366
cg17146640	22	46933650	*CELSR1*	TSS1500	Island	0.174±0.158	0.352±0.204	-0.178	2.72E-02	2.91E-05	-0.204
cg05859777	11	2161102	*IGF2*	5'UTR	Island	0.573±0.096	0.751±0.127	-0.178	9.99E-04	5.69E-21	-0.440
cg11700824	10	88730823	*AGAP11*	5'UTR/1st intron	S_Shelf	0.336±0.166	0.513±0.183	-0.178	2.76E-02	4.19E-05	-0.200
cg25392995	22	50625926	*TRABD*	5'UTR/1st intron	N_Shelf	0.627±0.105	0.805±0.065	-0.178	1.48E-03	7.82E-06	-0.218
cg27404606	20	3747579	*C20orf27*	5'UTR/1st intron	N_Shore	0.269±0.105	0.446±0.141	-0.177	2.18E-03	2.61E-19	-0.423
cg01060026	3	129324501	*PLXND1*	1st exon	Island	0.345±0.167	0.522±0.204	-0.177	3.30E-02	6.23E-08	-0.263
cg16158807	9	95946891	*WNK2*	TSS1500	Island	0.323±0.193	0.5±0.162	-0.177	4.30E-02	1.93E-13	-0.352
cg20154403	19	37407486	*ZNF568*	5'UTR/1st exon	S_Shore	0.219±0.14	0.396±0.266	-0.177	3.67E-02	2.18E-29	-0.516
cg20154403	19	37407486	*ZNF829*	TSS1500	S_Shore	0.219±0.14	0.396±0.266	-0.177	3.67E-02	5.16E-17	-0.397
cg10798745	1	145715636	*CD160*	1st exon	S_Shore	0.635±0.117	0.812±0.042	-0.177	3.26E-03	4.44E-14	-0.360
cg01568973	1	53528991	*PODN*	5'UTR/1st intron	S_Shore	0.602±0.093	0.779±0.063	-0.177	6.36E-04	4.30E-10	-0.301
cg07409680	12	32260442	*BICD1*	1st exon	S_Shore	0.129±0.095	0.306±0.222	-0.177	8.94E-03	6.61E-22	-0.450
cg04230060	9	114937992	*SUSD1*	TSS1500	S_Shore	0.581±0.099	0.758±0.096	-0.177	9.48E-04	4.56E-17	-0.398
cg11863536	19	10543050	*PDE4A*	TSS200	Island	0.279±0.142	0.456±0.19	-0.177	1.68E-02	3.38E-14	-0.362
cg15387123	9	139891058	*CLIC3*	TSS200	S_Shore	0.336±0.121	0.513±0.156	-0.177	5.88E-03	8.09E-08	-0.261
cg04555966	7	44185986	*GCK*	TSS1500	Island	0.556±0.095	0.733±0.171	-0.177	2.64E-03	5.71E-08	-0.263
cg14692284	5	118693725	*TNFAIP8*	5'UTR	S_Shore	0.674±0.11	0.851±0.025	-0.177	2.51E-03	6.85E-12	-0.330
cg20381020	11	66624258	*LRFN4*	TSS1500	Island	0.34±0.111	0.517±0.125	-0.177	2.60E-03	1.74E-16	-0.391
cg06804564	1	90100070	*LRRC8C*	5'UTR/1st intron	S_Shore	0.335±0.159	0.511±0.248	-0.177	4.10E-02	1.16E-08	-0.276
cg12444861	6	144386275	*PLAGL1*	TSS1500	S_Shore	0.521±0.073	0.698±0.157	-0.176	6.62E-04	5.32E-07	-0.244
cg03075605	11	96123810	*JRKL*	5'UTR	S_Shore	0.29±0.064	0.466±0.25	-0.176	1.07E-02	1.24E-09	-0.294
cg06538345	19	49993186	*RPL13A*	5'UTR	S_Shelf	0.612±0.077	0.788±0.135	-0.176	3.65E-04	1.41E-16	-0.392
cg14534464	7	26191633	*NFE2L3*	TSS1500	N_Shore	0.197±0.16	0.373±0.228	-0.176	3.56E-02	2.98E-08	-0.269
cg19563932	6	31583915	*AIF1*	1st exon	N_Shelf	0.507±0.073	0.684±0.117	-0.176	1.35E-04	3.62E-07	-0.248
cg11848173	12	56121015	*CD63*	5'UTR	N_Shore	0.586±0.136	0.763±0.085	-0.176	7.38E-03	3.26E-21	-0.443
cg23553480	2	233925275	*INPP5D*	1st exon	Island	0.404±0.161	0.58±0.207	-0.176	3.09E-02	5.83E-08	-0.263
cg14405137	12	50219283	*NCKAP5L*	5'UTR/1st intron	N_Shelf	0.566±0.091	0.742±0.039	-0.176	6.99E-04	5.86E-09	-0.282
cg01394116	5	150081499	*RBM22*	TSS1500	S_Shore	0.521±0.111	0.698±0.153	-0.176	3.88E-03	2.16E-09	-0.289
cg11448683	1	45274099	*BTBD19*	TSS200	S_Shore	0.225±0.095	0.401±0.152	-0.176	1.78E-03	5.34E-12	-0.331
cg00045515	20	35723332	*RBL1*	5'UTR/1st intron	N_Shore	0.672±0.087	0.848±0.072	-0.176	3.59E-04	1.11E-09	-0.294
cg00186701	8	98290510	*TSPYL5*	TSS1500	S_Shore	0.598±0.093	0.774±0.051	-0.176	7.09E-04	1.55E-20	-0.436
cg14741691	19	40855329	*C19orf47*	TSS1500	S_Shore	0.558±0.182	0.734±0.17	-0.176	3.79E-02	6.43E-07	-0.242
cg07217954	7	1067459	*C7orf50*	5'UTR/1st intron	Island	0.483±0.082	0.659±0.138	-0.176	5.84E-04	4.03E-05	-0.201
cg02398682	1	32802228	*MARCKSL1*	TSS1500	Island	0.302±0.115	0.477±0.144	-0.176	4.19E-03	2.21E-16	-0.390
cg20737812	15	86336631	*KLHL25*	5'UTR/1st intron	N_Shore	0.488±0.128	0.664±0.17	-0.176	9.48E-03	5.12E-12	-0.331
cg02939139	12	113574204	*RASAL1*	TSS1500	S_Shore	0.305±0.096	0.48±0.21	-0.176	7.35E-03	5.16E-09	-0.283
cg13947995	15	91427821	*FES*	5'UTR/1st intron	S_Shelf	0.145±0.09	0.32±0.299	-0.176	3.30E-02	9.74E-24	-0.467
cg23676551	11	2161374	*IGF2*	5'UTR	Island	0.6±0.09	0.776±0.08	-0.176	4.79E-04	2.12E-18	-0.413
cg14183922	17	75371532	*SEPT9*	5'UTR	S_Shore	0.605±0.078	0.78±0.045	-0.175	2.15E-04	4.25E-06	-0.224
cg18809289	10	45869816	*ALOX5*	1st exon	Island	0.313±0.152	0.488±0.145	-0.175	1.65E-02	2.46E-06	-0.230
cg20051772	12	56325015	*DGKA*	5'UTR/1st exon	S_Shelf	0.229±0.118	0.405±0.261	-0.175	2.68E-02	3.35E-05	-0.203
cg18105842	17	7341440	*FGF11*	TSS1500	N_Shore	0.563±0.102	0.738±0.05	-0.175	1.49E-03	1.03E-22	-0.458
cg09671005	5	102594958	*C5orf30*	5'UTR/1st intron	Island	0.107±0.138	0.282±0.258	-0.175	3.47E-02	2.01E-07	-0.253
cg04332373	4	15779642	*CD38*	TSS1500	N_Shore	0.554±0.108	0.729±0.106	-0.175	2.04E-03	7.13E-14	-0.358
cg14399078	3	119298802	*ADPRH*	5'UTR/1st intron	Island	0.05±0.01	0.226±0.222	-0.175	3.89E-03	1.18E-08	-0.276
cg15052854	3	119298811	*ADPRH*	5'UTR/1st intron	Island	0.084±0.01	0.258±0.181	-0.175	7.63E-04	5.28E-11	-0.316
cg24690071	13	100635352	*ZIC2*	1st exon	N_Shore	0.322±0.124	0.497±0.231	-0.175	2.01E-02	4.72E-08	-0.265
cg16001384	8	134306801	*NDRG1*	5'UTR/1st intron	N_Shore	0.649±0.12	0.824±0.03	-0.174	4.22E-03	1.04E-29	-0.518
cg03564674	20	62715756	*OPRL1*	5'UTR/1st intron	Island	0.257±0.124	0.431±0.229	-0.174	1.98E-02	2.02E-05	-0.208
cg10622551	4	2366555	*ZFYVE28*	5'UTR/1st exon	Island	0.47±0.053	0.645±0.158	-0.174	3.51E-04	1.68E-06	-0.233
cg06869445	16	16043240	*ABCC1*	TSS200	Island	0.119±0.161	0.293±0.202	-0.174	3.12E-02	1.63E-05	-0.211
cg12642521	11	68781202	*MRGPRF*	TSS1500	S_Shelf	0.306±0.13	0.48±0.133	-0.174	7.63E-03	2.97E-05	-0.204
cg15048534	17	74381192	*SPHK1*	1st exon	Island	0.237±0.13	0.411±0.162	-0.174	9.80E-03	1.56E-08	-0.274
cg05373723	4	141347993	*CLGN*	5'UTR/1st intron	N_Shore	0.21±0.122	0.383±0.197	-0.174	1.22E-02	3.64E-17	-0.399
cg20948757	5	114632659	*CCDC112*	TSS1500	S_Shore	0.103±0.106	0.277±0.24	-0.174	1.71E-02	1.52E-12	-0.339
cg21589115	19	49867114	*TEAD2*	TSS1500	S_Shore	0.217±0.137	0.39±0.226	-0.174	2.51E-02	2.81E-16	-0.388
cg17079034	2	239047280	*KLHL30*	TSS200	N_Shelf	0.376±0.071	0.549±0.113	-0.174	1.16E-04	1.20E-08	-0.276
cg07474022	20	35974912	*SRC*	5'UTR/1st intron	Island	0.242±0.129	0.415±0.182	-0.173	1.25E-02	2.34E-23	-0.464
cg05554039	11	75379223	*MAP6*	1st exon	Island	0.25±0.144	0.424±0.157	-0.173	1.50E-02	4.37E-06	-0.224
cg04755561	16	3029641	*PKMYT1*	5'UTR	N_Shore	0.582±0.147	0.756±0.085	-0.173	1.21E-02	1.51E-23	-0.465
cg04528038	16	21171097	*TMEM159*	5'UTR/1st intron	S_Shore	0.494±0.12	0.668±0.097	-0.173	4.09E-03	7.87E-10	-0.297
cg12200124	5	179220820	*LTC4S*	TSS200	N_Shore	0.444±0.097	0.617±0.054	-0.173	1.05E-03	2.27E-09	-0.289
cg19913118	1	145471701	*ANKRD34A*	5'UTR	N_Shore	0.432±0.062	0.605±0.133	-0.173	1.42E-04	3.17E-06	-0.227

cg19194454	16	69760878	*NQO1*	TSS1500	S_Shore	0.26±0.15	0.433±0.22	-0.173	3.04E-02	1.98E-29	-0.516
cg02952008	1	228604065	*TRIM17*	5'UTR/1st intron	Island	0.32±0.151	0.493±0.168	-0.173	1.99E-02	1.02E-08	-0.277
cg02561771	2	102091318	*RFX8*	TSS200	S_Shore	0.527±0.11	0.7±0.104	-0.173	2.36E-03	4.72E-07	-0.245
cg06829266	16	446668	*NME4*	TSS1500	N_Shore	0.178±0.145	0.351±0.217	-0.173	2.70E-02	3.19E-14	-0.362
cg07313319	22	38714426	*CSNK1E*	TSS1500	S_Shore	0.557±0.09	0.73±0.046	-0.173	6.70E-04	7.68E-20	-0.429
cg01577187	2	106362832	*NCK2*	5'UTR/1st intron	S_Shore	0.194±0.097	0.367±0.223	-0.173	1.10E-02	1.54E-08	-0.274
cg02672854	6	26521696	*HCG11*	TSS1500	N_Shore	0.155±0.126	0.328±0.224	-0.173	2.01E-02	2.35E-08	-0.271
cg01525376	1	32716212	*LCK*	TSS1500	S_Shelf	0.457±0.067	0.63±0.064	-0.173	3.49E-05	2.71E-07	-0.250
cg05256605	5	121412184	*LOX*	5'UTR/1st intron	N_Shore	0.533±0.118	0.706±0.122	-0.173	4.21E-03	1.94E-08	-0.272
cg14473743	3	13462496	*NUP210*	TSS1500	S_Shore	0.478±0.099	0.651±0.119	-0.173	1.36E-03	4.50E-06	-0.224
cg26961808	7	128470913	*FLNC*	1st exon	Island	0.408±0.19	0.581±0.122	-0.173	4.05E-02	9.58E-09	-0.278
cg06718763	1	24828586	*RCAN3*	TSS1500	N_Shore	0.088±0.056	0.26±0.211	-0.173	4.06E-03	3.31E-07	-0.248
cg14540150	10	126849760	*CTBP2*	TSS1500	Island	0.183±0.159	0.355±0.201	-0.172	3.12E-02	3.54E-10	-0.303
cg07833467	22	50986511	*KLHDC7B*	1st exon	Island	0.591±0.087	0.763±0.119	-0.172	6.12E-04	3.95E-06	-0.225
cg23153655	2	10220816	*CYS1*	TSS1500	Island	0.39±0.166	0.562±0.186	-0.172	3.26E-02	5.88E-08	-0.263
cg05766251	17	37024760	*LASP1*	TSS1500	N_Shore	0.219±0.037	0.391±0.194	-0.172	1.75E-03	3.69E-08	-0.267
cg27366007	15	91479184	*UNC45A*	5'UTR	S_Shelf	0.456±0.143	0.628±0.146	-0.172	1.38E-02	1.24E-12	-0.340
cg03043696	1	1820656	*GNB1*	5'UTR/1st intron	N_Shore	0.393±0.085	0.565±0.141	-0.172	9.13E-04	8.65E-09	-0.279
cg26215406	3	182972625	*B3GNT5*	5'UTR/1st intron	Island	0.472±0.154	0.644±0.131	-0.172	1.81E-02	2.18E-36	-0.567
cg18660345	20	35169539	*MYL9*	TSS1500	Island	0.62±0.039	0.792±0.057	-0.172	2.26E-08	9.05E-06	-0.217
cg02010711	6	31126864	*TCF19*	5'UTR	S_Shore	0.236±0.061	0.407±0.192	-0.172	2.29E-03	1.78E-11	-0.323
cg12163132	17	59475917	*TBX2*	TSS1500	Island	0.596±0.087	0.768±0.055	-0.172	4.84E-04	7.37E-13	-0.344
cg12640394	10	82116596	*DYDC1*	TSS200	Island	0.48±0.106	0.652±0.179	-0.172	5.99E-03	1.46E-07	-0.255
cg04858616	1	40255010	*BMP8B*	TSS1500	Island	0.085±0.077	0.257±0.235	-0.172	1.07E-02	1.99E-05	-0.208
cg16116973	20	33759608	*PROCR*	TSS200	N_Shelf	0.32±0.076	0.492±0.098	-0.172	1.50E-04	6.66E-07	-0.242
cg04411841	16	58033649	*ZNF319*	5'UTR/1st exon	N_Shore	0.309±0.074	0.48±0.165	-0.172	1.22E-03	5.42E-06	-0.222
cg06362282	6	33245893	*B3GALT4*	1st exon	S_Shore	0.559±0.078	0.731±0.068	-0.172	1.76E-04	2.68E-06	-0.229
cg16684661	8	80678770	*HEY1*	TSS200	S_Shore	0.522±0.138	0.694±0.142	-0.171	1.17E-02	3.05E-14	-0.363
cg03664992	1	39957393	*BMP8A*	5'UTR/1st exon	Island	0.439±0.061	0.611±0.121	-0.171	7.82E-05	3.59E-07	-0.248
cg27024876	19	49867109	*TEAD2*	TSS1500	S_Shore	0.188±0.096	0.359±0.218	-0.171	1.03E-02	1.77E-13	-0.352
cg26687670	1	241804939	*OPN3*	TSS1500	S_Shore	0.656±0.153	0.828±0.074	-0.171	1.58E-02	5.36E-40	-0.590
cg16627090	7	44105050	*PGAM2*	1st exon	Island	0.497±0.145	0.668±0.19	-0.171	2.20E-02	5.53E-14	-0.359
cg24703660	18	21719549	*CABYR*	5'UTR/1st intron	Island	0.371±0.153	0.543±0.214	-0.171	3.24E-02	8.34E-08	-0.260
cg27206867	4	10119810	*WDR1*	TSS1500	S_Shore	0.649±0.097	0.82±0.039	-0.171	1.33E-03	2.52E-14	-0.364
cg23613162	1	27891416	*AHDC1*	5'UTR/1st intron	N_Shelf	0.553±0.07	0.724±0.063	-0.171	6.26E-05	2.38E-12	-0.336
cg02849309	6	144386280	*PLAGL1*	TSS1500	S_Shore	0.595±0.067	0.767±0.164	-0.171	9.35E-04	2.41E-08	-0.271
cg00948524	17	29297757	*RNF135*	TSS200	N_Shore	0.463±0.118	0.634±0.158	-0.171	6.90E-03	4.94E-55	-0.670
cg08662052	19	44764603	*ZNF233*	5'UTR/1st intron	S_Shore	0.625±0.144	0.796±0.115	-0.171	1.28E-02	5.75E-25	-0.479
cg13556663	20	1246779	*SNPH*	TSS200	Island	0.225±0.137	0.396±0.19	-0.171	1.81E-02	2.75E-10	-0.304
cg02533787	2	149895023	*LYPD6B*	5'UTR/1st exon	Island	0.233±0.116	0.403±0.217	-0.171	1.61E-02	6.97E-06	-0.219
cg16139227	12	123451304	*ABCB9*	TSS1500	S_Shore	0.167±0.071	0.338±0.212	-0.171	5.66E-03	3.72E-07	-0.247
cg04453471	10	30726531	*MAP3K8*	5'UTR	S_Shelf	0.325±0.105	0.496±0.265	-0.171	2.72E-02	1.07E-30	-0.526
cg09061993	1	211499620	*TRAF5*	TSS1500	N_Shore	0.679±0.107	0.85±0.035	-0.171	2.45E-03	5.94E-07	-0.243
cg15999165	5	102201421	*PAM*	5'UTR/1st intron	N_Shore	0.341±0.118	0.512±0.144	-0.171	5.79E-03	1.81E-08	-0.273
cg04012924	11	13690702	*FAR1*	5'UTR/1st intron	Island	0.304±0.144	0.474±0.199	-0.170	2.36E-02	1.28E-14	-0.367
cg02695343	12	6881595	*LAG3*	TSS200	N_Shore	0.496±0.121	0.666±0.088	-0.170	4.71E-03	4.72E-12	-0.332
cg27350307	19	8567502	*PRAM1*	5'UTR/1st exon	S_Shelf	0.477±0.116	0.648±0.113	-0.170	3.98E-03	2.77E-13	-0.350
cg17690322	11	67206434	*PTPRCAP*	TSS1500	S_Shelf	0.612±0.083	0.782±0.048	-0.170	3.86E-04	3.88E-34	-0.551
cg19839655	20	44746499	*CD40*	TSS1500	N_Shore	0.112±0.09	0.282±0.184	-0.170	4.28E-03	6.68E-10	-0.298
cg10090650	6	24357732	*DCDC2*	1st exon	N_Shelf	0.544±0.143	0.715±0.18	-0.170	1.94E-02	1.02E-17	-0.405
cg10090650	6	24357732	*KAAG1*	5'UTR/1st exon	N_Shelf	0.544±0.143	0.715±0.18	-0.170	1.94E-02	3.70E-11	-0.318
cg26253438	1	203598623	*ATP2B4*	5'UTR/1st intron	Island	0.52±0.066	0.69±0.066	-0.170	3.55E-05	1.14E-05	-0.214
cg24910675	9	130616917	*ENG*	5'UTR/1st exon	S_Shore	0.381±0.138	0.551±0.144	-0.170	1.25E-02	3.25E-15	-0.375
cg26954736	11	73693896	*UCP2*	TSS200	Island	0.37±0.103	0.54±0.114	-0.170	1.89E-03	8.43E-09	-0.279
cg13934236	22	43507609	*BIK*	5'UTR/1st intron	S_Shore	0.627±0.082	0.797±0.071	-0.170	2.84E-04	1.29E-21	-0.447
cg15353612	4	52904965	*SGCB*	TSS1500	S_Shore	0.382±0.168	0.552±0.119	-0.170	2.68E-02	1.96E-06	-0.232
cg13583230	14	24809297	*RIPK3*	TSS200	S_Shore	0.455±0.119	0.625±0.105	-0.170	4.61E-03	9.17E-31	-0.527
cg15311822	2	9144618	*MBOAT2*	TSS1500	Island	0.507±0.107	0.677±0.099	-0.170	2.27E-03	4.81E-22	-0.451
cg15790214	6	26522325	*HCG11*	1st exon	Island	0.627±0.068	0.797±0.051	-0.170	6.18E-05	5.80E-39	-0.584
cg08328777	15	45422004	*DUOX1*	TSS200	Island	0.65±0.077	0.82±0.063	-0.170	1.82E-04	1.52E-05	-0.211
cg08328777	15	45422004	*DUOXA1*	5'UTR/1st exon	Island	0.65±0.077	0.82±0.063	-0.170	1.82E-04	1.55E-05	-0.211
cg21838979	2	106681945	*C2orf40*	TSS200	N_Shore	0.526±0.112	0.695±0.074	-0.170	2.94E-03	2.11E-10	-0.306
cg02520639	8	82021210	*PAG1*	5'UTR/1st intron	N_Shelf	0.352±0.11	0.521±0.257	-0.170	2.71E-02	7.73E-11	-0.313
cg22162872	2	191272697	*MFSD6*	TSS1500	Island	0.042±0.014	0.211±0.209	-0.170	3.19E-03	3.67E-06	-0.226
cg23484599	11	31831137	*PAX6*	5'UTR	S_Shelf	0.683±0.163	0.852±0.042	-0.169	2.20E-02	1.69E-17	-0.403
cg11298343	19	41306150	*EGLN2*	5'UTR/1st intron	S_Shore	0.285±0.102	0.454±0.168	-0.169	4.61E-03	8.25E-11	-0.313
cg10414736	6	26521747	*HCG11*	TSS200	N_Shore	0.266±0.104	0.435±0.197	-0.169	9.01E-03	3.68E-14	-0.361
cg14112356	21	46348443	*ITGB2*	5'UTR/1st intron	N_Shelf	0.381±0.164	0.55±0.182	-0.169	3.31E-02	1.03E-05	-0.215
cg19279042	6	31550090	*LTB*	1st exon	S_Shore	0.493±0.088	0.661±0.115	-0.169	7.03E-04	2.82E-14	-0.363
cg09547767	1	39957387	*BMP8A*	5'UTR/1st exon	Island	0.261±0.054	0.429±0.1	-0.169	1.25E-05	1.29E-05	-0.213
cg10853949	17	80290217	*SECTM1*	5'UTR/1st intron	N_Shore	0.662±0.172	0.831±0.065	-0.169	2.78E-02	5.62E-14	-0.359
cg01117549	5	137668198	*CDC25C*	TSS1500	S_Shore	0.334±0.153	0.502±0.209	-0.169	3.30E-02	3.02E-06	-0.228
cg23905216	11	2161390	*IGF2*	5'UTR	Island	0.58±0.075	0.748±0.085	-0.168	1.37E-04	7.78E-19	-0.418
cg23323669	6	31126866	*TCF19*	5'UTR	S_Shore	0.125±0.068	0.293±0.229	-0.168	9.17E-03	6.65E-15	-0.371
cg01278873	19	44764101	*ZNF233*	5'UTR/1st exon	Island	0.504±0.144	0.672±0.12	-0.168	1.40E-02	1.26E-24	-0.475
cg25186143	17	635064	*FAM57A*	TSS1500	N_Shore	0.349±0.134	0.518±0.162	-0.168	1.37E-02	1.33E-05	-0.213
cg15391574	11	134201993	*GLB1L2*	1st exon	Island	0.435±0.176	0.603±0.126	-0.168	3.38E-02	3.38E-08	-0.268
cg07644807	16	88769854	*RNF166*	5'UTR/1st exon	N_Shelf	0.475±0.095	0.644±0.05	-0.168	1.13E-03	1.25E-12	-0.340
cg01481646	10	82217548	*TSPAN14*	5'UTR/1st intron	S_Shelf	0.457±0.092	0.625±0.091	-0.168	7.87E-04	6.86E-20	-0.429
cg22802813	12	95942761	*USP44*	5'UTR/1st intron	Island	0.502±0.099	0.67±0.105	-0.168	1.44E-03	2.66E-14	-0.363
cg04671742	12	6881997	*LAG3*	5'UTR/1st exon	N_Shore	0.68±0.088	0.848±0.03	-0.168	8.57E-04	4.37E-29	-0.513
cg10137837	17	6926742	*BCL6B*	5'UTR/1st intron	Island	0.509±0.083	0.677±0.056	-0.168	3.78E-04	1.11E-07	-0.258
cg01570972	2	10090656	*GRHL1*	TSS1500	N_Shore	0.52±0.095	0.688±0.167	-0.168	3.73E-03	4.18E-06	-0.225
cg08858210	8	98290361	*TSPYL5*	TSS200	Island	0.449±0.058	0.617±0.06	-0.168	9.95E-06	7.94E-17	-0.395
cg06981309	3	146260954	*PLSCR1*	5'UTR/1st intron	N_Shore	0.409±0.144	0.577±0.204	-0.168	2.71E-02	3.33E-31	-0.530
cg03328639	12	123451280	*ABCB9*	TSS1500	S_Shore	0.185±0.097	0.353±0.171	-0.168	4.36E-03	8.00E-09	-0.279
cg23752828	12	57941309	*DCTN2*	TSS1500	S_Shore	0.402±0.153	0.57±0.221	-0.168	3.72E-02	8.44E-07	-0.240

cg13954457	5	167956819	*FBLL1*	1st exon	Island	0.57±0.116	0.738±0.115	-0.168	4.54E-03	7.06E-16	-0.383
cg14457599	17	74381214	*SPHK1*	1st exon	Island	0.149±0.114	0.316±0.224	-0.168	1.88E-02	4.64E-07	-0.245
cg07596494	2	26205095	*KIF3C*	5'UTR/1st exon	Island	0.233±0.152	0.401±0.183	-0.168	2.68E-02	2.92E-05	-0.204
cg11874426	11	118781731	*BCL9L*	TSS200	Island	0.433±0.138	0.601±0.244	-0.167	3.71E-02	1.72E-06	-0.233
cg09970023	14	61191253	*SIX4*	TSS1500	S_Shore	0.477±0.12	0.644±0.219	-0.167	2.00E-02	7.46E-22	-0.449
cg16077257	15	71146853	*LARP6*	TSS1500	S_Shore	0.168±0.08	0.335±0.158	-0.167	1.63E-03	1.42E-09	-0.293
cg24519393	1	38470540	*FHL3*	5'UTR/1st intron	N_Shore	0.253±0.143	0.42±0.259	-0.167	4.61E-02	5.27E-26	-0.488
cg17293195	11	71237522	*KRTAP5-7*	TSS1500	S_Shelf	0.521±0.178	0.688±0.138	-0.167	3.80E-02	8.07E-07	-0.240
cg11425280	12	104851172	*CHST11*	5'UTR/1st exon	Island	0.138±0.114	0.305±0.231	-0.167	2.13E-02	1.32E-08	-0.275
cg12229979	19	17190259	*MYO9B*	5'UTR/1st intron	S_Shelf	0.669±0.08	0.836±0.04	-0.167	3.94E-04	2.40E-06	-0.230
cg25742326	1	901449	*PLEKHN1*	TSS1500	Island	0.515±0.072	0.682±0.109	-0.167	3.66E-04	7.09E-15	-0.371
cg21618521	6	33245770	*B3GALT4*	1st exon	S_Shore	0.54±0.09	0.706±0.063	-0.167	7.66E-04	2.35E-06	-0.230
cg08126211	6	24357720	*DCDC2*	1st exon	N_Shelf	0.394±0.153	0.561±0.206	-0.167	3.42E-02	1.70E-09	-0.291
cg08126211	6	24357720	*KAAG1*	5'UTR/1st exon	N_Shelf	0.394±0.153	0.561±0.206	-0.167	3.42E-02	5.08E-07	-0.244
cg24433016	6	80713408	*TTK*	TSS1500	N_Shore	0.54±0.142	0.707±0.178	-0.167	2.09E-02	2.42E-21	-0.444
cg13879483	12	95942907	*USP44*	5'UTR/1st intron	Island	0.652±0.122	0.818±0.103	-0.166	5.86E-03	7.83E-12	-0.329
cg23468927	11	67206263	*PTPRCAP*	TSS1500	S_Shelf	0.7±0.091	0.866±0.052	-0.166	8.73E-04	9.98E-41	-0.594
cg17848515	2	130940706	*SMPD4*	TSS1500	S_Shore	0.583±0.108	0.749±0.154	-0.166	5.17E-03	2.37E-21	-0.444
cg20805133	2	242802192	*PDCD1*	TSS1500	N_Shelf	0.208±0.134	0.374±0.257	-0.166	4.09E-02	2.25E-06	-0.231
cg22339338	1	159891638	*TAGLN2*	5'UTR/1st intron	N_Shelf	0.575±0.117	0.742±0.1	-0.166	4.53E-03	3.96E-10	-0.302
cg04767484	15	76629104	*ISL2*	5'UTR/1st exon	N_Shore	0.277±0.165	0.443±0.19	-0.166	3.88E-02	4.46E-07	-0.246
cg08935125	16	3068085	*CLDN6*	5'UTR/1st intron	Island	0.658±0.058	0.824±0.03	-0.166	3.59E-05	1.74E-07	-0.254
cg18912470	4	57848125	*POLR2B*	5'UTR/1st intron	S_Shelf	0.644±0.093	0.81±0.068	-0.166	9.13E-04	1.27E-05	-0.213
cg16397884	5	132082837	*CCNI2*	TSS1500	N_Shore	0.148±0.098	0.314±0.184	-0.166	6.60E-03	8.10E-10	-0.297
cg17021218	10	88731069	*AGAP11*	5'UTR/1st intron	S_Shelf	0.464±0.117	0.63±0.161	-0.166	8.27E-03	1.30E-05	-0.213
cg22790377	1	228291668	*C1orf35*	TSS1500	N_Shelf	0.413±0.142	0.579±0.251	-0.166	4.41E-02	1.07E-06	-0.238
cg06712013	12	49759545	*SPATS2*	TSS1500	N_Shore	0.597±0.08	0.762±0.092	-0.166	3.03E-04	1.44E-12	-0.339
cg01529538	14	23388837	*RBM23*	TSS1500	Island	0.008±0.008	0.174±0.202	-0.166	2.84E-03	2.41E-13	-0.350
cg16731157	1	24830054	*RCAN3*	5'UTR/1st intron	S_Shore	0.125±0.053	0.291±0.243	-0.166	1.23E-02	1.11E-20	-0.437
cg03840504	3	122296369	*PARP15*	TSS200	N_Shore	0.289±0.139	0.455±0.195	-0.166	2.36E-02	1.71E-14	-0.366
cg07884764	11	64107517	*CCDC88B*	TSS200	N_Shelf	0.609±0.116	0.774±0.075	-0.166	4.25E-03	5.08E-22	-0.451
cg13023677	5	137675418	*FAM53C*	5'UTR/1st intron	S_Shore	0.644±0.089	0.81±0.069	-0.166	7.21E-04	3.75E-06	-0.226
cg19872463	12	6881629	*LAG3*	TSS200	N_Shore	0.542±0.141	0.708±0.123	-0.166	1.42E-02	1.71E-11	-0.324
cg02298570	19	2840764	*ZNF555*	TSS1500	N_Shore	0.307±0.102	0.472±0.151	-0.165	3.89E-03	1.29E-09	-0.293
cg12914657	7	150413680	*GIMAP1*	5'UTR/1st exon	N_Shelf	0.384±0.107	0.55±0.137	-0.165	3.94E-03	5.82E-10	-0.299
cg03427543	2	18060490	*KCNS3*	5'UTR/1st intron	Island	0.072±0.092	0.237±0.249	-0.165	2.33E-02	2.71E-05	-0.205
cg07639783	7	1610747	*PSMG3*	TSS1500	S_Shore	0.427±0.105	0.592±0.234	-0.165	2.32E-02	2.07E-06	-0.231
cg04375036	12	111181819	*PPP1CC*	TSS1500	S_Shore	0.639±0.187	0.804±0.139	-0.165	4.76E-02	4.85E-20	-0.431
cg16352928	12	6881590	*LAG3*	TSS200	N_Shore	0.488±0.112	0.654±0.104	-0.165	3.62E-03	1.68E-09	-0.291
cg11305121	2	121011590	*RALB*	5'UTR/1st intron	S_Shore	0.49±0.125	0.655±0.134	-0.165	8.47E-03	3.05E-06	-0.228
cg12812165	14	51296728	*NIN*	5'UTR	Island	0.16±0.073	0.325±0.179	-0.165	2.72E-03	1.16E-09	-0.294
cg15526708	9	101866433	*TGFBR1*	TSS1500	N_Shore	0.438±0.132	0.603±0.126	-0.165	1.07E-02	4.02E-05	-0.201
cg12210293	7	150413801	*GIMAP1*	5'UTR/1st intron	N_Shelf	0.466±0.143	0.631±0.101	-0.165	1.39E-02	4.88E-20	-0.431
cg04462652	2	133429088	*LYPD1*	5'UTR/1st exon	S_Shore	0.155±0.081	0.32±0.158	-0.165	1.89E-03	9.36E-10	-0.296
cg16479173	11	47290859	*MADD*	TSS1500	N_Shore	0.182±0.112	0.347±0.191	-0.165	1.19E-02	1.16E-07	-0.257
cg20449614	8	22438122	*PDLIM2*	1st exon	S_Shore	0.384±0.129	0.549±0.188	-0.165	1.78E-02	5.55E-13	-0.345
cg14967066	11	314341	*IFITM1*	1st exon	S_Shelf	0.377±0.119	0.542±0.208	-0.165	1.81E-02	2.62E-35	-0.559
cg24605046	6	33245895	*B3GALT4*	1st exon	S_Shore	0.474±0.086	0.638±0.078	-0.164	5.58E-04	1.77E-05	-0.210
cg06784108	13	107187232	*EFNB2*	1st exon	Island	0.402±0.157	0.566±0.208	-0.164	4.03E-02	1.42E-06	-0.235
cg15254238	10	70321874	*TET1*	5'UTR/1st intron	S_Shore	0.564±0.108	0.728±0.104	-0.164	3.16E-03	1.40E-09	-0.293
cg08873266	1	40255052	*BMP8B*	TSS1500	Island	0.224±0.052	0.388±0.177	-0.164	1.55E-03	1.15E-06	-0.237
cg26250609	11	67351273	*GSTP1*	5'UTR/1st exon	Island	0.502±0.137	0.666±0.063	-0.164	1.15E-02	4.97E-22	-0.451
cg23764381	10	116852687	*ATRNL1*	TSS1500	Island	0.125±0.098	0.288±0.222	-0.164	1.52E-02	7.25E-06	-0.219
cg12668155	11	12031113	*DKK3*	TSS1500	S_Shore	0.234±0.076	0.398±0.092	-0.164	2.07E-04	8.22E-12	-0.328
cg05003422	1	85513889	*MCOLN3*	5'UTR/1st intron	Island	0.171±0.148	0.335±0.221	-0.164	3.80E-02	9.69E-06	-0.216
cg08684361	6	30852823	*DDR1*	5'UTR	S_Shore	0.161±0.056	0.324±0.186	-0.164	2.50E-03	3.35E-08	-0.268
cg01527459	10	82116571	*DYDC1*	TSS200	Island	0.533±0.074	0.697±0.18	-0.163	3.18E-03	2.18E-13	-0.351
cg18693051	19	1161800	*SBNO2*	5'UTR/1st intron	S_Shelf	0.679±0.183	0.842±0.075	-0.163	4.07E-02	6.92E-12	-0.329
cg09854317	12	123754235	*CDK2AP1*	5'UTR/1st intron	N_Shore	0.601±0.123	0.765±0.098	-0.163	6.87E-03	3.83E-14	-0.361
cg17518962	7	99766552	*GAL3ST4*	TSS200	N_Shelf	0.31±0.097	0.473±0.166	-0.163	4.91E-03	3.15E-06	-0.227
cg11216632	1	155944655	*ARHGEF2*	5'UTR/1st intron	N_Shelf	0.594±0.122	0.757±0.053	-0.163	6.45E-03	3.92E-18	-0.410
cg00487737	19	11689726	*ACP5*	5'UTR/1st exon	Island	0.343±0.11	0.506±0.153	-0.163	6.26E-03	5.60E-22	-0.450
cg00726615	3	113252640	*SIDT1*	5'UTR/1st intron	S_Shore	0.25±0.108	0.413±0.179	-0.163	9.23E-03	1.91E-11	-0.323
cg05452899	11	2161133	*IGF2*	5'UTR	Island	0.61±0.078	0.773±0.048	-0.163	3.52E-04	8.33E-21	-0.439
cg22776856	16	3072572	*THOC6*	TSS1500	N_Shore	0.506±0.135	0.669±0.153	-0.163	1.56E-02	1.81E-10	-0.307
cg26126806	17	17685031	*RAI1*	5'UTR	Island	0.54±0.112	0.703±0.093	-0.163	3.81E-03	3.23E-05	-0.203
cg26173997	4	76555856	*CDKL2*	TSS200	Island	0.445±0.117	0.608±0.109	-0.163	5.56E-03	3.92E-05	-0.201
cg19266578	20	62711729	*OPRL1*	5'UTR/1st intron	N_Shelf	0.432±0.161	0.595±0.144	-0.163	3.03E-02	1.13E-15	-0.381
cg19266578	20	62711729	*RGS19*	TSS1500	N_Shelf	0.432±0.161	0.595±0.144	-0.163	3.03E-02	2.06E-13	-0.351
cg19428417	16	66959729	*RRAD*	TSS1500	S_Shelf	0.524±0.065	0.686±0.144	-0.163	5.96E-04	3.12E-11	-0.320
cg26542660	4	56813860	*CEP135*	TSS1500	N_Shore	0.391±0.066	0.554±0.196	-0.162	4.58E-03	5.14E-07	-0.244
cg04777348	1	92952897	*GFI1*	TSS1500	S_Shore	0.678±0.086	0.841±0.057	-0.162	6.96E-04	1.54E-18	-0.415
cg00588297	2	74753691	*DQX1*	TSS1500	N_Shelf	0.561±0.075	0.723±0.074	-0.162	1.91E-04	6.49E-17	-0.396
cg22243039	17	42147465	*G6PC3*	TSS1500	N_Shore	0.491±0.068	0.653±0.108	-0.162	1.49E-04	3.75E-06	-0.226
cg03989260	1	119529360	*TBX15*	5'UTR/1st intron	N_Shore	0.47±0.107	0.633±0.127	-0.162	3.90E-03	4.93E-06	-0.223
cg06697536	2	177053496	*HOXD1*	5'UTR/1st exon	Island	0.398±0.14	0.56±0.212	-0.162	3.18E-02	2.19E-06	-0.231
cg07318313	9	130616620	*ENG*	1st exon	Island	0.473±0.094	0.635±0.056	-0.162	1.31E-03	8.25E-24	-0.468
cg22038579	12	8849964	*RIMKLB*	5'UTR/1st intron	Island	0.147±0.086	0.309±0.234	-0.162	1.67E-02	3.96E-05	-0.201
cg18233405	8	98290148	*TSPYL5*	5'UTR/1st exon	Island	0.423±0.066	0.585±0.098	-0.162	8.40E-05	7.55E-11	-0.314
cg24156149	2	197033988	*STK17B*	5'UTR/1st intron	N_Shore	0.479±0.099	0.641±0.106	-0.162	2.03E-03	6.33E-16	-0.384
cg15904523	19	44763979	*ZNF233*	TSS200	Island	0.488±0.166	0.649±0.16	-0.162	3.75E-02	4.25E-16	-0.386
cg15611336	15	75248496	*RPP25*	1st exon	N_Shelf	0.405±0.16	0.567±0.172	-0.162	3.58E-02	5.84E-15	-0.372
cg01555853	2	18059744	*KCNS3*	5'UTR/1st intron	Island	0.156±0.136	0.317±0.217	-0.162	3.19E-02	3.34E-08	-0.268
cg00074818	8	8560427	*CLDN23*	1st exon	Island	0.595±0.132	0.756±0.065	-0.161	1.02E-02	5.70E-07	-0.243
cg22056218	11	63973536	*FERMT3*	TSS1500	N_Shore	0.518±0.058	0.679±0.11	-0.161	6.08E-05	1.59E-15	-0.379
cg08714753	11	35639590	*FJX1*	TSS200	N_Shore	0.294±0.098	0.455±0.189	-0.161	8.77E-03	2.77E-05	-0.205
cg24566261	6	30854164	*DDR1*	5'UTR	S_Shore	0.444±0.099	0.605±0.14	-0.161	3.41E-03	1.40E-10	-0.310

cg07971674	14	23388846	*RBM23*	TSS1500	Island	0.032±0.01	0.193±0.182	-0.161	1.59E-03	9.89E-13	-0.342
cg15962197	1	41704561	*SCMH1*	5'UTR/1st intron	N_Shelf	0.409±0.063	0.57±0.15	-0.161	7.80E-04	1.62E-06	-0.234
cg14052210	6	26521980	*HCG11*	1st exon	Island	0.514±0.079	0.674±0.088	-0.161	3.21E-04	3.63E-28	-0.506
cg18566594	12	4381435	*CCND2*	TSS1500	Island	0.349±0.162	0.51±0.178	-0.161	3.95E-02	3.87E-05	-0.201
cg14221550	8	120220050	*MAL2*	TSS1500	N_Shore	0.269±0.12	0.43±0.235	-0.161	3.06E-02	5.47E-06	-0.222
cg04233664	10	126848743	*CTBP2*	5'UTR/1st intron	Island	0.166±0.147	0.327±0.225	-0.161	4.32E-02	9.99E-07	-0.238
cg17602126	1	40105687	*HEYL*	TSS1500	Island	0.615±0.145	0.775±0.151	-0.160	2.20E-02	1.70E-05	-0.210
cg07903144	6	33358872	*KIFC1*	TSS1500	N_Shore	0.682±0.099	0.843±0.046	-0.160	2.10E-03	4.52E-06	-0.224
cg08159663	16	57022486	*NLRC5*	TSS1500	N_Shore	0.347±0.133	0.508±0.124	-0.160	1.26E-02	4.34E-28	-0.505
cg03662014	16	29086271	*RRN3P2*	1st exon	Island	0.567±0.085	0.727±0.042	-0.160	7.93E-04	9.28E-11	-0.312
cg24082526	19	49993125	*RPL13A*	5'UTR	S_Shelf	0.524±0.053	0.684±0.173	-0.160	1.65E-03	3.46E-15	-0.375
cg03236948	11	63997492	*DNAJC4*	TSS1500	N_Shore	0.317±0.127	0.477±0.162	-0.160	1.47E-02	6.00E-08	-0.263
cg24512005	11	3014291	*NAP1L4*	TSS1500	S_Shore	0.347±0.09	0.507±0.181	-0.160	6.28E-03	1.95E-09	-0.290
cg13804575	9	109626999	*ZNF462*	5'UTR/1st intron	S_Shelf	0.544±0.146	0.704±0.179	-0.160	2.84E-02	1.22E-05	-0.214
cg01633363	17	79140589	*AATK*	TSS1500	S_Shore	0.436±0.143	0.596±0.173	-0.160	2.54E-02	1.37E-08	-0.275
cg01262865	16	75282094	*BCAR1*	5'UTR/1st exon	N_Shelf	0.426±0.057	0.586±0.054	-0.160	1.39E-05	1.71E-11	-0.324
cg10256234	11	66624034	*LRFN4*	TSS1500	Island	0.506±0.021	0.666±0.085	-0.160	2.29E-07	3.71E-16	-0.387
cg21158528	1	211501629	*TRAF5*	5'UTR/1st intron	S_Shore	0.641±0.099	0.801±0.059	-0.160	2.04E-03	3.27E-06	-0.227
cg01578324	6	31704774	*CLIC1*	5'UTR/1st exon	N_Shelf	0.495±0.088	0.655±0.105	-0.160	9.62E-04	6.71E-21	-0.440
cg16587838	6	31704705	*CLIC1*	5'UTR/1st exon	N_Shelf	0.284±0.097	0.443±0.17	-0.160	6.97E-03	3.36E-24	-0.472
cg03679581	9	5304511	*RLN2*	1st exon	S_Shore	0.449±0.132	0.609±0.104	-0.160	1.12E-02	2.90E-10	-0.304
cg21096345	3	32432942	*CMTM7*	TSS1500	N_Shore	0.177±0.09	0.337±0.197	-0.160	9.15E-03	4.49E-15	-0.373
cg02044612	11	67772000	*UNC93B1*	TSS1500	S_Shore	0.654±0.145	0.813±0.074	-0.160	1.68E-02	1.32E-10	-0.310
cg00965469	6	31649469	*LY6G5C*	TSS1500	N_Shore	0.422±0.159	0.581±0.177	-0.160	3.76E-02	5.70E-07	-0.243
cg21626848	17	39969267	*FKBP10*	5'UTR/1st exon	S_Shore	0.557±0.094	0.717±0.072	-0.159	1.34E-03	6.47E-43	-0.607
cg21386373	3	52279602	*PPM1M*	TSS1500	Island	0.181±0.127	0.341±0.181	-0.159	1.84E-02	1.97E-14	-0.365
cg13499300	19	54369556	*MYADM*	5'UTR/1st exon	N_Shelf	0.612±0.083	0.771±0.062	-0.159	5.44E-04	1.04E-07	-0.258
cg10317807	10	97804045	*CCNJ*	5'UTR/1st intron	Island	0.234±0.147	0.393±0.185	-0.159	3.16E-02	5.11E-17	-0.397
cg10379890	6	391208	*IRF4*	TSS1500	Island	0.375±0.156	0.534±0.173	-0.159	3.50E-02	3.37E-07	-0.248
cg14015211	1	223315766	*TLR5*	5'UTR/1st intron	N_Shore	0.756±0.189	0.916±0.053	-0.159	4.90E-02	2.11E-17	-0.402
cg08551725	7	30029776	*SCRN1*	TSS1500	Island	0.417±0.109	0.577±0.182	-0.159	1.18E-02	8.28E-08	-0.260
cg06431702	11	66624841	*LRFN4*	5'UTR/1st intron	Island	0.492±0.123	0.651±0.159	-0.159	1.31E-02	4.25E-20	-0.431
cg02937671	4	2366485	*ZFYVE28*	5'UTR/1st exon	Island	0.62±0.073	0.778±0.117	-0.159	4.03E-04	3.33E-08	-0.268
cg18085627	11	64107672	*CCDC88B*	TSS200	N_Shelf	0.513±0.157	0.672±0.16	-0.159	3.35E-02	2.36E-07	-0.251
cg25015733	2	99342986	*MGAT4A*	5'UTR	N_Shelf	0.522±0.155	0.681±0.154	-0.159	3.05E-02	3.20E-05	-0.203
cg05484548	16	29086428	*RRN3P2*	1st exon	Island	0.331±0.078	0.489±0.057	-0.159	3.61E-04	8.80E-09	-0.279
cg22486834	6	52859107	*GSTA4*	5'UTR/1st intron	N_Shore	0.619±0.087	0.777±0.1	-0.159	9.06E-04	7.61E-25	-0.477
cg23973429	8	102504746	*GRHL2*	5'UTR/1st exon	Island	0.519±0.071	0.678±0.08	-0.159	1.46E-04	2.03E-20	-0.435
cg15801007	6	131383376	*EPB41L2*	5'UTR/1st intron	N_Shore	0.135±0.069	0.294±0.221	-0.159	1.14E-02	1.38E-10	-0.310
cg12085570	19	8674501	*ADAMTS10*	5'UTR/1st intron	Island	0.18±0.069	0.338±0.295	-0.159	4.35E-02	2.89E-13	-0.349
cg22977317	6	2842550	*SERPINB1*	TSS1500	S_Shore	0.603±0.064	0.761±0.093	-0.159	6.68E-05	5.78E-15	-0.372
cg23018117	7	150413687	*GIMAP1*	5'UTR/1st exon	N_Shelf	0.44±0.101	0.599±0.085	-0.159	2.38E-03	3.15E-17	-0.400
cg04896949	1	28918475	*RAB42*	TSS1500	N_Shore	0.378±0.107	0.537±0.158	-0.158	7.45E-03	3.99E-15	-0.374
cg06976589	4	1304048	*MAEA*	5'UTR/1st intron	S_Shore	0.672±0.136	0.831±0.048	-0.158	1.28E-02	4.78E-07	-0.245
cg08806632	19	13214412	*LYL1*	TSS1500	N_Shore	0.586±0.105	0.745±0.091	-0.158	3.08E-03	5.10E-22	-0.451
cg12406559	18	56530302	*ZNF532*	1st exon	N_Shore	0.283±0.097	0.442±0.251	-0.158	2.96E-02	2.02E-12	-0.337
cg18972998	1	55266296	*TTC22*	1st exon	Island	0.71±0.137	0.868±0.121	-0.158	1.53E-02	1.90E-17	-0.402
cg24581462	8	38759627	*PLEKHA2*	5'UTR/1st intron	S_Shore	0.121±0.106	0.28±0.157	-0.158	7.09E-03	8.37E-15	-0.370
cg10122302	1	6051558	*NPHP4*	5'UTR/1st intron	N_Shore	0.079±0.035	0.237±0.208	-0.158	5.57E-03	3.19E-07	-0.249
cg18588934	9	132083215	*C9orf106*	TSS200	Island	0.055±0.08	0.213±0.19	-0.158	6.33E-03	3.92E-10	-0.302
cg06487082	18	56530106	*ZNF532*	1st exon	N_Shore	0.224±0.08	0.382±0.224	-0.158	1.43E-02	1.34E-10	-0.310
cg14228592	8	145639181	*SLC39A4*	TSS1500	Island	0.552±0.106	0.71±0.123	-0.158	4.26E-03	2.26E-12	-0.337
cg13521889	19	12075705	*ZNF763*	TSS200	N_Shore	0.254±0.121	0.412±0.199	-0.158	2.12E-02	3.10E-26	-0.490
cg22862003	21	42797588	*MX1*	5'UTR	N_Shore	0.5±0.12	0.658±0.087	-0.158	7.08E-03	1.98E-14	-0.365
cg04907151	3	135684161	*PPP2R3A*	TSS1500	N_Shore	0.571±0.146	0.728±0.086	-0.158	1.86E-02	2.98E-08	-0.269
cg14524975	12	53594856	*ITGB7*	5'UTR	S_Shelf	0.413±0.132	0.57±0.136	-0.157	1.49E-02	6.41E-10	-0.298
cg07903023	2	239048072	*KLHL30*	5'UTR/1st intron	N_Shore	0.684±0.042	0.842±0.057	-0.157	2.70E-07	4.07E-17	-0.398
cg12832565	1	145715672	*CD160*	TSS200	S_Shore	0.733±0.093	0.89±0.03	-0.157	1.82E-03	5.00E-17	-0.397
cg06753439	6	33245488	*B3GALT4*	1st exon	Island	0.569±0.066	0.726±0.046	-0.157	1.10E-04	2.51E-13	-0.350
cg18668813	6	114177341	*MARCKS*	TSS1500	N_Shelf	0.181±0.135	0.338±0.193	-0.157	2.77E-02	5.71E-08	-0.263
cg19982230	17	27045302	*RAB34*	TSS1500	N_Shore	0.454±0.021	0.612±0.14	-0.157	1.88E-04	1.40E-22	-0.456
cg23378365	5	156696351	*CYFIP2*	5'UTR/1st exon	S_Shelf	0.483±0.126	0.641±0.142	-0.157	1.27E-02	1.01E-11	-0.327
cg24876187	6	31126871	*TCF19*	5'UTR	S_Shore	0.181±0.097	0.338±0.196	-0.157	1.16E-02	9.85E-21	-0.438
cg24876187	6	31126871	*CCHCR1*	TSS1500	S_Shore	0.181±0.097	0.338±0.196	-0.157	1.16E-02	1.11E-07	-0.258
cg14612785	3	71774842	*EIF4E3*	5'UTR	Island	0.118±0.085	0.275±0.258	-0.157	3.01E-02	3.08E-15	-0.375
cg06688014	10	111683554	*XPNPEP1*	TSS1500	S_Shore	0.341±0.08	0.498±0.174	-0.157	4.42E-03	4.28E-07	-0.246
cg14531862	19	44764205	*ZNF233*	5'UTR/1st intron	Island	0.481±0.096	0.638±0.124	-0.157	2.64E-03	1.77E-19	-0.425
cg23612030	2	96811533	*DUSP2*	TSS1500	S_Shore	0.274±0.16	0.431±0.193	-0.157	4.55E-02	2.23E-05	-0.207
cg18087326	1	53067465	*GPX7*	TSS1500	N_Shore	0.714±0.065	0.871±0.031	-0.157	1.46E-04	1.62E-26	-0.492
cg24905196	3	121555186	*EAF2*	5'UTR/1st intron	S_Shore	0.63±0.078	0.787±0.071	-0.157	3.44E-04	1.93E-16	-0.390
cg24905196	3	121555186	*IQCB1*	TSS1500	S_Shore	0.63±0.078	0.787±0.071	-0.157	3.44E-04	4.55E-08	-0.265
cg27002699	19	11689538	*ACP5*	5'UTR/1st exon	Island	0.242±0.135	0.399±0.205	-0.157	3.15E-02	6.04E-13	-0.345
cg11874323	3	111804830	*C3orf52*	TSS1500	N_Shore	0.531±0.111	0.688±0.094	-0.157	4.63E-03	1.90E-11	-0.323
cg09554509	7	27238910	*HOXA13*	1st exon	Island	0.281±0.104	0.438±0.281	-0.157	4.95E-02	1.81E-10	-0.307
cg01716224	22	40391434	*FAM83F*	1st exon	Island	0.502±0.068	0.659±0.074	-0.157	9.58E-05	2.90E-19	-0.422
cg05384724	12	124155451	*TCTN2*	TSS1500	N_Shore	0.494±0.126	0.651±0.179	-0.157	1.96E-02	1.31E-07	-0.256
cg13269555	10	102822002	*KAZALD1*	5'UTR/1st intron	N_Shelf	0.543±0.103	0.7±0.16	-0.157	7.25E-03	1.34E-48	-0.639
cg14885762	17	75446450	*SEPT9*	TSS1500	N_Shore	0.378±0.13	0.535±0.188	-0.157	2.38E-02	5.38E-12	-0.331
cg23057870	8	80679111	*HEY1*	TSS1500	S_Shore	0.563±0.137	0.72±0.173	-0.157	2.44E-02	1.63E-17	-0.403
cg07348922	6	33244990	*B3GALT4*	5'UTR/1st exon	Island	0.457±0.163	0.613±0.069	-0.156	3.05E-02	7.59E-57	-0.678
cg26434048	7	45961569	*IGFBP3*	TSS1500	S_Shore	0.434±0.115	0.591±0.197	-0.156	1.90E-02	1.20E-07	-0.257
cg16536016	8	38757775	*PLEKHA2*	TSS1500	N_Shore	0.419±0.089	0.576±0.141	-0.156	2.70E-03	4.00E-05	-0.201
cg23310850	19	19051337	*HOMER3*	5'UTR/1st intron	Island	0.083±0.053	0.239±0.176	-0.156	2.30E-03	8.44E-06	-0.217
cg13399816	1	68299468	*GNG12*	TSS1500	S_Shore	0.353±0.129	0.509±0.19	-0.156	2.40E-02	4.34E-12	-0.332
cg10059687	1	6526129	*TNFRSF25*	1st exon	Island	0.473±0.093	0.628±0.095	-0.156	1.64E-03	2.97E-15	-0.376
cg03023152	8	102505556	*GRHL2*	5'UTR/1st intron	S_Shore	0.522±0.111	0.678±0.133	-0.156	6.87E-03	1.66E-17	-0.403
cg00249621	8	98290359	*TSPYL5*	TSS200	Island	0.489±0.063	0.645±0.068	-0.156	5.17E-05	7.13E-19	-0.418

cg05204104	2	235403141	*ARL4C*	1st exon/3'UTR	N_Shore	0.462±0.169	0.618±0.157	-0.156	4.55E-02	1.27E-14	-0.368
cg21431832	22	26875652	*HPS4*	TSS200	N_Shelf	0.335±0.128	0.491±0.237	-0.155	4.16E-02	2.53E-06	-0.229
cg05758434	2	207139471	*ZDBF2*	5'UTR/1st exon	Island	0.174±0.076	0.329±0.164	-0.155	3.11E-03	2.36E-10	-0.306
cg22878489	6	33245701	*B3GALT4*	1st exon	S_Shore	0.671±0.087	0.826±0.057	-0.155	1.06E-03	7.28E-09	-0.280
cg02211741	11	46383924	*DGKZ*	5'UTR/1st intron	N_Shelf	0.585±0.081	0.74±0.043	-0.155	6.67E-04	3.94E-11	-0.318
cg24886267	5	167956306	*FBLL1*	5'UTR/1st exon	Island	0.147±0.159	0.302±0.173	-0.155	4.25E-02	5.89E-07	-0.243
cg08678256	16	30196023	*CORO1A*	5'UTR	S_Shore	0.379±0.096	0.534±0.15	-0.155	4.83E-03	2.05E-07	-0.253
cg08545136	17	76775543	*CYTH1*	5'UTR/1st intron	N_Shore	0.406±0.107	0.56±0.148	-0.155	7.46E-03	1.43E-08	-0.275
cg25934064	3	52569026	*NT5DC2*	5'UTR/1st exon	S_Shore	0.573±0.096	0.728±0.096	-0.154	2.07E-03	1.91E-37	-0.574
cg00004421	17	7475079	*EIF4A1*	TSS1500	N_Shore	0.429±0.072	0.583±0.152	-0.154	1.75E-03	9.98E-07	-0.238
cg12628196	7	127672458	*LRRC4*	TSS1500	Island	0.592±0.065	0.746±0.042	-0.154	1.23E-04	5.77E-13	-0.345
cg00039326	1	36615845	*TRAPPC3*	5'UTR/1st intron	S_Shore	0.595±0.145	0.749±0.105	-0.154	2.16E-02	3.32E-05	-0.203
cg22225943	11	2162536	*IGF2*	5'UTR	S_Shore	0.541±0.116	0.695±0.134	-0.154	9.11E-03	5.57E-10	-0.299
cg13937155	17	1548881	*SCARF1*	1st exon	S_Shore	0.591±0.089	0.745±0.051	-0.154	1.31E-03	2.20E-12	-0.337
cg26997028	5	76012827	*F2R*	5'UTR/1st intron	S_Shore	0.56±0.083	0.714±0.055	-0.154	7.58E-04	1.45E-07	-0.256
cg12708994	6	116691460	*DSE*	5'UTR	N_Shore	0.625±0.072	0.779±0.078	-0.154	2.11E-04	9.93E-17	-0.394
cg20954977	2	232260116	*B3GNT7*	TSS1500	Island	0.681±0.126	0.834±0.064	-0.154	1.02E-02	4.06E-12	-0.333
cg09258150	19	10397429	*ICAM4*	TSS1500	N_Shelf	0.335±0.164	0.489±0.132	-0.153	3.91E-02	5.22E-07	-0.244
cg18997901	6	76461321	*MYO6*	5'UTR/1st intron	S_Shelf	0.612±0.143	0.765±0.066	-0.153	1.88E-02	5.66E-18	-0.408
cg23878206	19	50431425	*IL4I1*	5'UTR/1st intron	N_Shore	0.604±0.093	0.757±0.116	-0.153	2.31E-03	2.14E-14	-0.365
cg23878206	19	50431425	*NUP62*	5'UTR/1st intron	N_Shore	0.604±0.093	0.757±0.116	-0.153	2.31E-03	2.77E-14	-0.363
cg22740547	11	13030876	*RASSF10*	TSS200	Island	0.458±0.139	0.611±0.172	-0.153	2.83E-02	7.65E-06	-0.218
cg19670286	5	137610127	*GFRA3*	5'UTR/1st exon	Island	0.448±0.058	0.602±0.058	-0.153	2.91E-05	1.55E-20	-0.436
cg04546041	19	57863210	*ZNF304*	5'UTR/1st intron	Island	0.346±0.097	0.499±0.216	-0.153	2.00E-02	1.21E-09	-0.294
cg21595709	19	15344186	*EPHX3*	TSS1500	Island	0.418±0.096	0.571±0.108	-0.153	2.66E-03	3.00E-12	-0.335
cg14704921	4	53728654	*RASL11B*	5'UTR/1st exon	Island	0.305±0.128	0.458±0.123	-0.153	1.39E-02	3.18E-07	-0.249
cg25189904	1	68299493	*GNG12*	TSS1500	S_Shore	0.352±0.144	0.505±0.206	-0.153	4.26E-02	3.61E-12	-0.334
cg07803420	6	30851517	*DDR1*	5'UTR/1st intron	N_Shore	0.589±0.147	0.742±0.085	-0.152	2.25E-02	9.95E-35	-0.555
cg20017995	9	136216076	*MED22*	TSS1500	S_Shore	0.49±0.127	0.643±0.172	-0.152	2.12E-02	1.22E-17	-0.404
cg17252884	3	15369681	*SH3BP5*	5'UTR	N_Shelf	0.513±0.142	0.665±0.093	-0.152	1.97E-02	4.22E-14	-0.361
cg26398848	2	263559	*SH3YL1*	5'UTR/1st intron	Island	0.498±0.122	0.65±0.09	-0.152	9.50E-03	4.27E-37	-0.571
cg07894352	19	44763993	*ZNF233*	TSS200	Island	0.546±0.149	0.699±0.143	-0.152	3.05E-02	7.86E-30	-0.519
cg20808711	6	32160998	*GPSM3*	5'UTR	N_Shelf	0.674±0.045	0.826±0.047	-0.152	1.65E-06	3.47E-25	-0.480
cg09645475	20	815316	*FAM110A*	5'UTR/1st intron	S_Shore	0.4±0.104	0.552±0.215	-0.152	2.30E-02	4.99E-27	-0.496
cg18080046	6	31704844	*CLIC1*	5'UTR/1st exon	N_Shelf	0.303±0.087	0.454±0.114	-0.152	1.68E-03	2.51E-26	-0.490
cg08144943	3	52280702	*PPM1M*	5'UTR/1st intron	S_Shore	0.459±0.09	0.61±0.115	-0.152	2.08E-03	1.49E-27	-0.501
cg19050555	12	49662405	*TUBA1C*	5'UTR	S_Shelf	0.655±0.107	0.806±0.108	-0.151	5.21E-03	2.42E-26	-0.491
cg03852656	3	186525224	*RFC4*	TSS1500	S_Shore	0.375±0.156	0.526±0.162	-0.151	4.05E-02	2.89E-18	-0.411
cg22695339	17	7791630	*CHD3*	TSS1500	S_Shelf	0.215±0.05	0.366±0.153	-0.151	1.02E-03	3.76E-06	-0.226
cg04590184	17	29297604	*RNF135*	TSS1500	N_Shore	0.448±0.12	0.599±0.156	-0.151	1.50E-02	1.44E-21	-0.446
cg25783099	2	42795486	*MTA3*	5'UTR	N_Shore	0.496±0.131	0.647±0.083	-0.151	1.40E-02	1.05E-20	-0.438
cg08808402	5	115910732	*SEMA6A*	TSS200	N_Shore	0.108±0.106	0.259±0.188	-0.151	1.63E-02	1.80E-05	-0.210
cg16268214	12	49351537	*ARF3*	TSS1500	S_Shore	0.194±0.142	0.344±0.2	-0.151	4.10E-02	8.61E-06	-0.217
cg08916374	1	145471727	*ANKRD34A*	5'UTR	N_Shore	0.44±0.1	0.59±0.133	-0.151	5.10E-03	8.94E-06	-0.217
cg01745690	7	43798100	*BLVRA*	TSS200	Island	0.049±0.077	0.2±0.202	-0.151	1.12E-02	1.23E-11	-0.326
cg17630392	5	32712623	*NPR3*	1st exon	Island	0.506±0.101	0.657±0.118	-0.151	4.27E-03	7.76E-06	-0.218
cg16899892	1	53528971	*PODN*	5'UTR/1st intron	Island	0.577±0.083	0.727±0.035	-0.150	1.09E-03	1.89E-14	-0.365
cg15732851	1	203598761	*ATP2B4*	5'UTR/1st intron	Island	0.706±0.071	0.856±0.039	-0.150	3.40E-04	1.20E-06	-0.236
cg14951292	16	4525986	*NMRAL1*	5'UTR/1st intron	S_Shore	0.456±0.097	0.606±0.133	-0.150	4.59E-03	5.64E-08	-0.264
cg10578938	5	156695410	*CYFIP2*	5'UTR/1st intron	S_Shore	0.561±0.11	0.712±0.129	-0.150	7.86E-03	4.36E-12	-0.332
cg08296037	16	1584118	*TMEM204*	5'UTR	Island	0.583±0.051	0.733±0.041	-0.150	1.18E-05	1.67E-14	-0.366
cg11843238	5	131593191	*PDLIM4*	TSS200	Island	0.426±0.127	0.576±0.205	-0.150	3.36E-02	3.82E-07	-0.247
cg11210813	20	35972385	*SRC*	TSS1500	N_Shore	0.532±0.102	0.682±0.144	-0.150	6.94E-03	7.97E-10	-0.297
cg06173720	7	127670993	*LRRC4*	5'UTR/1st exon	N_Shore	0.603±0.071	0.753±0.054	-0.149	2.64E-04	4.36E-14	-0.360
cg22166883	1	24116900	*LYPLA2*	TSS1500	N_Shore	0.569±0.102	0.719±0.089	-0.149	3.69E-03	3.72E-07	-0.247
cg22322679	6	33244178	*B3GALT4*	TSS1500	N_Shore	0.629±0.069	0.779±0.027	-0.149	3.47E-04	1.36E-16	-0.392
cg14121845	20	25566513	*NINL*	TSS1500	Island	0.299±0.1	0.448±0.206	-0.149	2.04E-02	1.74E-08	-0.273
cg23462242	5	156696185	*CYFIP2*	5'UTR/1st intron	S_Shelf	0.661±0.117	0.81±0.137	-0.149	1.21E-02	6.24E-10	-0.299
cg17497608	7	90896701	*FZD1*	1st exon/3'UTR	S_Shore	0.689±0.153	0.838±0.057	-0.149	2.89E-02	9.12E-07	-0.239
cg20299697	3	138069423	*MRAS*	5'UTR/1st intron	S_Shore	0.67±0.082	0.819±0.093	-0.149	9.21E-04	9.89E-32	-0.534
cg25201101	12	131355465	*RAN*	TSS1500	N_Shore	0.508±0.059	0.657±0.129	-0.149	4.77E-04	6.13E-12	-0.330
cg12897901	11	126225297	*ST3GAL4*	TSS1500	N_Shore	0.061±0.042	0.21±0.217	-0.149	1.12E-02	2.90E-05	-0.204
cg00891541	16	68481467	*SMPD3*	5'UTR/1st intron	Island	0.71±0.146	0.858±0.09	-0.149	2.47E-02	3.85E-11	-0.318
cg18485193	5	16936369	*MYO10*	5'UTR/1st exon	Island	0.401±0.158	0.55±0.173	-0.149	4.90E-02	1.69E-13	-0.353
cg00481884	19	11689867	*ACP5*	TSS1500	S_Shore	0.456±0.094	0.604±0.152	-0.149	6.19E-03	2.96E-25	-0.481
cg07191594	20	44650220	*SLC12A5*	TSS200	N_Shore	0.362±0.099	0.51±0.193	-0.148	1.65E-02	3.56E-13	-0.348
cg15754153	2	43865055	*PLEKHH2*	5'UTR/1st intron	S_Shore	0.106±0.075	0.255±0.161	-0.148	3.81E-03	4.60E-06	-0.224
cg24335895	19	36643771	*COX7A1*	5'UTR/1st exon	S_Shore	0.597±0.079	0.745±0.091	-0.148	7.29E-04	2.42E-06	-0.230
cg16164619	12	57871855	*ARHGAP9*	5'UTR/1st exon	S_Shore	0.286±0.109	0.435±0.164	-0.148	1.35E-02	8.62E-06	-0.217
cg02530437	17	27044745	*RAB34*	5'UTR/1st exon	N_Shelf	0.331±0.116	0.479±0.179	-0.148	2.02E-02	8.80E-13	-0.342
cg07968770	16	21170927	*TMEM159*	5'UTR/1st intron	S_Shore	0.511±0.138	0.659±0.154	-0.148	2.80E-02	2.05E-06	-0.231
cg14166009	19	37825309	*HKR1*	5'UTR	Island	0.532±0.127	0.68±0.131	-0.148	1.72E-02	2.83E-05	-0.205
cg15531369	19	49993157	*RPL13A*	5'UTR	S_Shelf	0.597±0.057	0.745±0.124	-0.148	3.62E-04	4.11E-15	-0.374
cg02498311	11	73020358	*ARHGEF17*	1st exon	Island	0.262±0.155	0.41±0.179	-0.148	4.94E-02	2.02E-06	-0.232
cg19215110	6	30850913	*DDR1*	5'UTR/1st intron	N_Shore	0.584±0.17	0.732±0.092	-0.148	4.70E-02	4.39E-26	-0.488
cg21365364	20	18568287	*DTD1*	TSS1500	N_Shore	0.206±0.099	0.353±0.203	-0.148	2.00E-02	3.97E-08	-0.266
cg17200690	6	33245619	*B3GALT4*	1st exon	S_Shore	0.621±0.085	0.768±0.033	-0.147	1.52E-03	2.84E-06	-0.228
cg06023994	3	170137871	*CLDN11*	TSS1500	Island	0.641±0.082	0.788±0.072	-0.147	9.20E-04	9.22E-11	-0.312
cg09619347	11	64107520	*CCDC88B*	TSS200	N_Shelf	0.468±0.084	0.615±0.132	-0.147	4.18E-03	1.33E-08	-0.275
cg16537028	16	84853380	*CRISPLD2*	TSS1500	Island	0.273±0.119	0.419±0.195	-0.147	2.84E-02	9.66E-07	-0.239
cg25974903	1	65211057	*RAVER2*	1st exon	Island	0.137±0.101	0.283±0.187	-0.147	1.69E-02	1.33E-08	-0.275
cg01430588	17	56769767	*RAD51C*	TSS200	N_Shore	0.547±0.123	0.693±0.154	-0.146	1.93E-02	6.12E-07	-0.243
cg08404227	15	92397279	*SLCO3A1*	1st exon	Island	0.066±0.053	0.212±0.215	-0.146	1.31E-02	3.24E-06	-0.227
cg27046604	15	41135869	*SPINT1*	TSS1500	Island	0.402±0.145	0.548±0.193	-0.146	4.70E-02	2.87E-17	-0.400
cg00798876	7	2670978	*TTYH3*	TSS1500	N_Shore	0.36±0.104	0.506±0.194	-0.146	2.05E-02	3.80E-06	-0.225
cg17402397	22	31477754	*SMTN*	5'UTR/1st intron	S_Shore	0.391±0.142	0.537±0.174	-0.146	3.79E-02	4.95E-18	-0.409
cg21959598	7	55637719	*VOPP1*	5'UTR/1st intron	N_Shore	0.449±0.123	0.594±0.162	-0.146	2.15E-02	4.74E-14	-0.360

cg01890845	11	8102475	*TUB*	TSS1500	Island	0.375±0.136	0.521±0.157	-0.146	2.93E-02	9.65E-08	-0.259
cg18366919	19	15344364	*EPHX3*	TSS1500	Island	0.426±0.109	0.572±0.159	-0.146	1.40E-02	6.22E-09	-0.281
cg02224002	16	88769526	*RNF166*	5'UTR/1st exon	N_Shelf	0.585±0.082	0.731±0.102	-0.146	1.23E-03	4.49E-08	-0.265
cg27321942	19	49993217	*RPL13A*	5'UTR	S_Shelf	0.595±0.07	0.741±0.123	-0.146	9.28E-04	7.00E-11	-0.314
cg04340435	13	114145973	*TMCO3*	5'UTR/1st intron	Island	0.344±0.111	0.49±0.204	-0.145	2.84E-02	2.08E-17	-0.402
cg04340435	13	114145973	*DCUN1D2*	TSS1500	Island	0.344±0.111	0.49±0.204	-0.145	2.84E-02	4.78E-07	-0.245
cg09087503	7	127672473	*LRRC4*	TSS1500	Island	0.499±0.043	0.644±0.049	-0.145	1.29E-06	1.50E-11	-0.324
cg05629015	10	35929139	*FZD8*	1st exon	Island	0.564±0.11	0.709±0.091	-0.145	7.29E-03	1.74E-05	-0.210
cg19820372	11	111411216	*LAYN*	5'UTR/1st intron	Island	0.46±0.121	0.605±0.114	-0.145	1.34E-02	5.43E-06	-0.222
cg07187855	6	30854161	*DDR1*	5'UTR	S_Shore	0.356±0.126	0.501±0.182	-0.145	3.02E-02	5.61E-11	-0.316
cg09273059	10	71077221	*HK1*	TSS1500	N_Shore	0.66±0.085	0.805±0.05	-0.145	1.49E-03	7.24E-07	-0.241
cg24837256	1	19284087	*IFFO2*	TSS1500	S_Shore	0.599±0.08	0.744±0.107	-0.145	1.26E-03	4.41E-07	-0.246
cg27545611	1	68517177	*DIRAS3*	5'UTR/1st exon	S_Shore	0.609±0.132	0.753±0.183	-0.145	3.52E-02	3.33E-08	-0.268
cg21625464	11	314044	*IFITM1*	5'UTR/1st exon	S_Shelf	0.22±0.114	0.364±0.19	-0.145	2.58E-02	5.95E-27	-0.496
cg03292284	1	241804036	*OPN3*	TSS1500	S_Shore	0.63±0.129	0.775±0.119	-0.144	1.86E-02	7.77E-17	-0.395
cg18801945	19	13215041	*LYL1*	TSS1500	N_Shore	0.696±0.08	0.841±0.04	-0.144	1.07E-03	1.28E-23	-0.466
cg04348222	1	32802307	*MARCKSL1*	TSS1500	S_Shore	0.498±0.104	0.642±0.14	-0.144	9.37E-03	1.46E-08	-0.275
cg00929635	20	44035918	*DBNDD2*	5'UTR/1st intron	Island	0.644±0.149	0.788±0.095	-0.144	3.15E-02	1.29E-19	-0.426
cg19760410	1	154300117	*ATP8B2*	5'UTR/1st intron	N_Shore	0.371±0.098	0.515±0.146	-0.144	8.02E-03	3.44E-05	-0.203
cg07483989	9	33751159	*PRSS3*	5'UTR/1st intron	Island	0.353±0.097	0.497±0.191	-0.144	1.82E-02	2.35E-09	-0.289
cg25291978	6	144386422	*PLAGL1*	TSS1500	S_Shore	0.741±0.072	0.885±0.054	-0.144	4.02E-04	1.79E-05	-0.210
cg10914115	6	31704828	*CLIC1*	5'UTR/1st exon	N_Shelf	0.395±0.082	0.539±0.129	-0.144	2.62E-03	3.28E-29	-0.514
cg19714957	19	19843923	*ZNF14*	TSS200	Island	0.336±0.145	0.48±0.147	-0.144	3.64E-02	9.72E-28	-0.502
cg00018181	7	101931734	*SH2B2*	5'UTR/1st intron	S_Shore	0.341±0.148	0.485±0.135	-0.143	3.70E-02	3.23E-05	-0.203
cg24641027	7	128050275	*IMPDH1*	TSS1500	Island	0.082±0.055	0.225±0.179	-0.143	5.32E-03	1.31E-06	-0.236
cg02018408	16	25269986	*ZKSCAN2*	TSS1500	S_Shore	0.242±0.1	0.385±0.148	-0.143	9.46E-03	6.58E-08	-0.262
cg04953061	14	53620393	*DDHD1*	TSS1500	S_Shore	0.28±0.122	0.423±0.157	-0.143	2.20E-02	9.64E-09	-0.278
cg23752923	1	85513892	*MCOLN3*	5'UTR/1st intron	Island	0.144±0.121	0.287±0.191	-0.143	3.19E-02	1.88E-05	-0.209
cg19410791	2	87037000	*CD8A*	TSS1500	Island	0.629±0.116	0.772±0.137	-0.143	1.47E-02	1.99E-07	-0.253
cg04361435	2	106362025	*NCK2*	5'UTR/1st intron	Island	0.021±0.029	0.164±0.229	-0.143	1.78E-02	5.18E-11	-0.316
cg15826897	19	15343373	*EPHX3*	5'UTR/1st intron	N_Shore	0.422±0.119	0.565±0.197	-0.143	3.26E-02	5.98E-07	-0.243
cg09184467	10	14995699	*DCLRE1C*	5'UTR/1st intron	Island	0.223±0.061	0.366±0.198	-0.143	1.09E-02	4.38E-13	-0.347
cg17995652	8	80678925	*HEY1*	TSS1500	S_Shore	0.591±0.108	0.734±0.129	-0.143	9.79E-03	2.57E-15	-0.376
cg02047547	16	19126242	*ITPRIPL2*	1st exon	N_Shore	0.593±0.099	0.736±0.043	-0.143	4.21E-03	4.96E-08	-0.265
cg18650307	4	17809468	*DCAF16*	5'UTR	N_Shelf	0.547±0.074	0.689±0.167	-0.143	5.86E-03	7.72E-06	-0.218
cg20265043	7	23510089	*IGF2BP3*	TSS200	S_Shore	0.173±0.117	0.316±0.23	-0.143	4.69E-02	3.75E-15	-0.374
cg07038400	3	135684163	*PPP2R3A*	TSS1500	N_Shore	0.632±0.142	0.774±0.09	-0.142	2.63E-02	4.70E-10	-0.301
cg23371746	1	119532925	*TBX15*	TSS1500	S_Shelf	0.508±0.152	0.65±0.146	-0.142	4.35E-02	1.23E-05	-0.214
cg09025215	1	223316219	*TLR5*	5'UTR/1st intron	Island	0.506±0.129	0.649±0.214	-0.142	4.86E-02	2.60E-06	-0.229
cg11614622	11	66623681	*LRFN4*	TSS1500	Island	0.645±0.09	0.787±0.083	-0.142	2.33E-03	5.07E-24	-0.470
cg07591622	14	24036914	*AP1G2*	5'UTR/1st exon	Island	0.065±0.018	0.207±0.191	-0.142	6.01E-03	1.41E-11	-0.325
cg03313364	17	40440904	*STAT5A*	5'UTR	Island	0.415±0.079	0.557±0.11	-0.142	1.49E-03	1.23E-13	-0.354
cg03764274	17	79632335	*CCDC137*	TSS1500	N_Shore	0.595±0.071	0.737±0.101	-0.142	6.26E-04	4.58E-10	-0.301
cg08641090	2	72375141	*CYP26B1*	TSS200	N_Shore	0.315±0.088	0.457±0.209	-0.142	2.28E-02	1.03E-07	-0.258
cg17434309	11	2161079	*IGF2*	5'UTR	Island	0.543±0.13	0.684±0.124	-0.142	2.20E-02	4.80E-12	-0.332
cg13806070	1	39957390	*BMP8A*	5'UTR/1st exon	Island	0.439±0.043	0.581±0.111	-0.142	9.62E-05	2.65E-06	-0.229
cg18686270	3	146258875	*PLSCR1*	5'UTR/1st intron	N_Shelf	0.692±0.09	0.834±0.073	-0.142	2.21E-03	1.21E-22	-0.457
cg07763231	17	80187349	*SLC16A3*	5'UTR/1st intron	Island	0.438±0.126	0.58±0.218	-0.142	4.92E-02	1.02E-09	-0.295
cg14374829	21	33244742	*HUNK*	TSS1500	N_Shore	0.188±0.094	0.329±0.184	-0.141	1.63E-02	9.76E-07	-0.238
cg16015113	1	36024366	*NCDN*	5'UTR/1st intron	S_Shore	0.413±0.13	0.554±0.165	-0.141	3.19E-02	1.74E-09	-0.291
cg06948408	1	43736600	*TMEM125*	5'UTR	N_Shore	0.15±0.083	0.291±0.143	-0.141	4.68E-03	1.55E-13	-0.353
cg14722509	5	170815938	*NPM1*	5'UTR/1st intron	S_Shore	0.07±0.013	0.21±0.222	-0.141	1.58E-02	3.03E-10	-0.304
cg08400147	19	42807187	*PAFAH1B3*	TSS1500	N_Shelf	0.118±0.049	0.258±0.157	-0.141	2.27E-03	3.49E-05	-0.202
cg02874908	11	67205113	*PTPRCAP*	5'UTR/1st exon	S_Shore	0.766±0.08	0.906±0.03	-0.140	1.34E-03	2.29E-46	-0.627
cg16242615	19	4059988	*ZBTB7A*	5'UTR/1st intron	Island	0.414±0.124	0.555±0.183	-0.140	3.41E-02	1.15E-05	-0.214
cg11862081	11	66625106	*LRFN4*	5'UTR/1st exon	Island	0.661±0.056	0.801±0.068	-0.140	4.55E-05	3.35E-20	-0.432
cg04060561	6	127663200	*ECHDC1*	5'UTR/1st intron	N_Shore	0.671±0.091	0.811±0.102	-0.140	3.35E-03	7.17E-07	-0.241
cg23679992	12	6421513	*PLEKHG6*	TSS1500	S_Shore	0.624±0.065	0.764±0.074	-0.140	1.95E-04	1.64E-09	-0.291
cg21275690	16	3072970	*THOC6*	TSS1500	N_Shore	0.432±0.048	0.572±0.116	-0.140	2.15E-04	2.00E-09	-0.290
cg18449964	18	72917101	*ZADH2*	5'UTR/1st intron	N_Shelf	0.502±0.107	0.642±0.166	-0.140	1.82E-02	2.34E-07	-0.251
cg26477856	12	56324915	*DGKA*	TSS1500	S_Shelf	0.533±0.083	0.673±0.118	-0.140	2.66E-03	4.04E-23	-0.461
cg10376827	10	88730324	*AGAP11*	TSS200	S_Shore	0.555±0.096	0.695±0.1	-0.140	4.55E-03	4.29E-10	-0.301
cg03721978	6	33245706	*B3GALT4*	1st exon	S_Shore	0.507±0.067	0.647±0.069	-0.140	2.58E-04	1.15E-06	-0.237
cg00878163	17	56565644	*HSF5*	5'UTR/1st exon	Island	0.759±0.068	0.899±0.031	-0.140	4.62E-04	1.40E-05	-0.212
cg22826239	7	23510123	*IGF2BP3*	TSS200	S_Shore	0.106±0.052	0.245±0.236	-0.140	2.73E-02	1.48E-08	-0.274
cg02898094	7	96634914	*DLX6*	TSS1500	Island	0.385±0.068	0.525±0.154	-0.139	3.81E-03	9.82E-12	-0.327
cg21291672	1	29451713	*TMEM200B*	TSS1500	Island	0.601±0.061	0.74±0.095	-0.139	2.18E-04	1.68E-06	-0.233
cg06707978	19	58095155	*ZIK1*	TSS200	Island	0.432±0.123	0.571±0.126	-0.139	1.95E-02	8.44E-16	-0.382
cg08696931	12	123754071	*CDK2AP1*	5'UTR/1st intron	N_Shore	0.574±0.08	0.713±0.053	-0.139	1.14E-03	1.20E-12	-0.341
cg16184495	19	15343350	*EPHX3*	5'UTR/1st intron	N_Shore	0.456±0.115	0.595±0.203	-0.139	3.74E-02	4.85E-08	-0.265
cg18337363	3	52569053	*NT5DC2*	5'UTR/1st exon	S_Shore	0.748±0.119	0.888±0.048	-0.139	1.27E-02	1.39E-45	-0.623
cg00564061	6	117001170	*KPNA5*	TSS1500	N_Shore	0.701±0.144	0.84±0.066	-0.139	2.95E-02	1.08E-07	-0.258
cg03034696	2	47400023	*CALM2*	5'UTR/1st exon	N_Shelf	0.298±0.114	0.436±0.179	-0.139	2.72E-02	5.65E-07	-0.243
cg05568549	6	41907198	*CCND3*	5'UTR	N_Shore	0.669±0.066	0.807±0.032	-0.139	3.85E-04	1.00E-18	-0.417
cg02718531	16	2932507	*FLYWCH2*	TSS1500	Island	0.119±0.091	0.258±0.185	-0.139	1.75E-02	2.01E-08	-0.272
cg20158248	16	16042956	*ABCC1*	TSS1500	N_Shore	0.24±0.122	0.378±0.16	-0.139	2.71E-02	1.07E-10	-0.311
cg21088488	7	44105721	*PGAM2*	TSS1500	S_Shore	0.355±0.061	0.494±0.159	-0.138	3.85E-03	1.15E-05	-0.214
cg12743727	10	44101728	*ZNF485*	TSS200	N_Shore	0.407±0.093	0.545±0.14	-0.138	7.66E-03	5.49E-06	-0.222
cg05503460	15	51387651	*TNFAIP8L3*	TSS1500	S_Shore	0.186±0.097	0.325±0.194	-0.138	2.35E-02	1.72E-06	-0.233
cg04831783	16	88769549	*RNF166*	5'UTR/1st exon	N_Shelf	0.494±0.09	0.632±0.103	-0.138	3.35E-03	2.03E-08	-0.272
cg06209689	1	47696348	*TAL1*	5'UTR	N_Shore	0.302±0.082	0.44±0.137	-0.138	4.41E-03	3.42E-05	-0.203
cg17301902	9	80263219	*GNA14*	5'UTR/1st exon	Island	0.304±0.058	0.442±0.179	-0.138	7.26E-03	6.53E-25	-0.478
cg08114617	12	110271441	*TRPV4*	TSS1500	Island	0.409±0.059	0.547±0.139	-0.138	1.62E-03	1.74E-15	-0.379
cg02690491	19	12075772	*ZNF763*	TSS200	N_Shore	0.236±0.065	0.374±0.139	-0.138	2.09E-03	4.78E-11	-0.317
cg25363789	11	64949878	*CAPN1*	5'UTR/1st intron	S_Shore	0.245±0.1	0.383±0.213	-0.138	3.40E-02	7.13E-23	-0.459
cg00761125	12	96590115	*ELK3*	5'UTR/1st intron	S_Shore	0.658±0.094	0.796±0.147	-0.138	9.29E-03	2.23E-06	-0.231
cg04661436	15	79169207	*MORF4L1*	5'UTR/1st intron	S_Shelf	0.593±0.085	0.731±0.093	-0.138	2.22E-03	3.76E-07	-0.247

cg25823419	3	179169252	*GNB4*	5'UTR/1st exon	Island	0.437±0.117	0.575±0.206	-0.138	4.18E-02	1.84E-08	-0.273
cg20034202	2	232260136	*B3GNT7*	TSS200	Island	0.547±0.093	0.685±0.129	-0.138	6.42E-03	2.79E-06	-0.228
cg05239956	14	24036509	*AP1G2*	5'UTR	Island	0.073±0.033	0.21±0.213	-0.137	1.54E-02	2.97E-15	-0.376
cg03743037	7	134673090	*AGBL3*	5'UTR	S_Shore	0.62±0.12	0.758±0.176	-0.137	3.19E-02	2.28E-06	-0.230
cg19626656	1	1053027	*C1orf159*	TSS1500	S_Shore	0.752±0.116	0.889±0.035	-0.137	1.19E-02	1.21E-06	-0.236
cg02504521	12	46660819	*SLC38A1*	5'UTR/1st intron	N_Shore	0.292±0.098	0.429±0.191	-0.137	2.43E-02	1.10E-14	-0.368
cg12154624	6	29760754	*HCG4*	1st exon	Island	0.078±0.009	0.216±0.166	-0.137	2.80E-03	1.65E-07	-0.254
cg16857428	19	9435206	*ZNF559*	5'UTR/1st intron	S_Shore	0.219±0.074	0.356±0.232	-0.137	3.33E-02	2.52E-19	-0.423
cg07106615	15	43785974	*TP53BP1*	5'UTR/1st intron	S_Shore	0.708±0.099	0.845±0.043	-0.137	5.46E-03	1.26E-07	-0.257
cg08224785	5	156696189	*CYFIP2*	5'UTR/1st intron	S_Shelf	0.708±0.093	0.845±0.042	-0.137	3.67E-03	1.21E-08	-0.276
cg01798499	12	123450932	*ABCB9*	5'UTR/1st exon	Island	0.052±0.12	0.189±0.205	-0.137	4.50E-02	3.43E-05	-0.203
cg02839123	22	40390264	*FAM83F*	TSS1500	N_Shore	0.37±0.065	0.507±0.142	-0.137	2.66E-03	6.38E-09	-0.281
cg04289385	6	36355825	*ETV7*	TSS1500	Island	0.324±0.129	0.46±0.178	-0.136	4.08E-02	1.00E-10	-0.312
cg07741984	3	111805005	*C3orf52*	TSS200	Island	0.264±0.112	0.4±0.17	-0.136	2.50E-02	7.52E-06	-0.219
cg14527110	5	131561292	*P4HA2*	5'UTR	N_Shore	0.463±0.1	0.599±0.122	-0.136	8.65E-03	4.26E-21	-0.442
cg24161793	2	96990762	*ITPRIPL1*	TSS1500	N_Shore	0.323±0.109	0.459±0.203	-0.136	3.68E-02	1.95E-21	-0.445
cg04962480	8	101962118	*YWHAZ*	5'UTR/1st intron	N_Shore	0.193±0.076	0.329±0.181	-0.136	1.26E-02	1.13E-18	-0.416
cg12857875	2	239047191	*KLHL30*	TSS200	N_Shelf	0.559±0.069	0.695±0.078	-0.136	4.41E-04	4.66E-13	-0.346
cg14315198	17	26879866	*UNC119*	TSS1500	S_Shore	0.37±0.1	0.506±0.2	-0.136	3.02E-02	2.37E-20	-0.434
cg20794824	3	170138205	*CLDN11*	TSS1500	S_Shore	0.603±0.083	0.738±0.058	-0.136	1.75E-03	2.24E-12	-0.337
cg02218324	19	46318439	*RSPH6A*	5'UTR/1st exon	N_Shore	0.684±0.075	0.82±0.032	-0.136	1.10E-03	2.65E-05	-0.205
cg02652260	21	18985383	*BTG3*	TSS200	Island	0.019±0.019	0.155±0.203	-0.135	1.17E-02	3.15E-05	-0.204
cg07949597	16	3355079	*ZNF75A*	TSS1500	Island	0.303±0.072	0.438±0.201	-0.135	1.92E-02	1.67E-07	-0.254
cg16800851	6	130339565	*L3MBTL3*	5'UTR/1st exon	N_Shore	0.164±0.107	0.299±0.204	-0.135	3.76E-02	1.91E-09	-0.290
cg10477603	10	126849704	*CTBP2*	TSS1500	Island	0.133±0.043	0.268±0.171	-0.135	4.95E-03	2.58E-06	-0.229
cg08687005	19	56988929	*ZNF667*	TSS200	Island	0.232±0.105	0.367±0.181	-0.135	2.57E-02	2.58E-15	-0.376
cg18359530	5	77945219	*LHFPL2*	TSS1500	Island	0.194±0.071	0.329±0.203	-0.135	1.99E-02	1.22E-05	-0.214
cg13791254	9	100616607	*FOXE1*	1st exon	S_Shore	0.587±0.144	0.722±0.149	-0.135	4.64E-02	7.36E-07	-0.241
cg14135060	5	150456285	*TNIP1*	5'UTR/1st intron	N_Shelf	0.621±0.072	0.756±0.136	-0.135	3.22E-03	6.82E-16	-0.384
cg24367568	19	49837281	*CD37*	TSS1500	N_Shelf	0.385±0.058	0.52±0.126	-0.135	1.06E-03	7.13E-08	-0.262
cg12836958	4	186132412	*SNX25*	5'UTR/1st intron	S_Shore	0.712±0.111	0.847±0.048	-0.135	1.04E-02	1.71E-11	-0.324
cg12800266	6	37225002	*TBC1D22B*	TSS1500	N_Shore	0.419±0.143	0.554±0.151	-0.135	4.64E-02	1.64E-07	-0.254
cg03960072	6	24357985	*DCDC2*	5'UTR	N_Shelf	0.442±0.103	0.577±0.154	-0.135	1.66E-02	4.32E-11	-0.317
cg03960072	6	24357985	*KAAG1*	1st exon	N_Shelf	0.442±0.103	0.577±0.154	-0.135	1.66E-02	3.91E-07	-0.247
cg10059450	11	66405549	*RBM4*	TSS1500	N_Shore	0.27±0.11	0.405±0.214	-0.135	4.55E-02	4.57E-09	-0.284
cg24503712	1	151762261	*TDRKH*	5'UTR/1st intron	N_Shore	0.685±0.085	0.819±0.099	-0.135	2.82E-03	1.58E-09	-0.292
cg23462514	4	1107585	*RNF212*	TSS1500	Island	0.668±0.099	0.802±0.061	-0.135	5.87E-03	3.86E-07	-0.247
cg01347228	17	29297391	*RNF135*	TSS1500	N_Shore	0.67±0.092	0.804±0.125	-0.134	6.75E-03	2.04E-17	-0.402
cg06823034	14	24780734	*LTB4R*	5'UTR/1st exon	Island	0.38±0.11	0.514±0.206	-0.134	4.14E-02	6.68E-10	-0.298
cg08839210	17	27045048	*RAB34*	5'UTR/1st intron	N_Shore	0.393±0.078	0.528±0.206	-0.134	2.43E-02	1.75E-22	-0.455
cg01548965	11	73088618	*RELT*	5'UTR/1st intron	S_Shore	0.68±0.062	0.814±0.032	-0.134	3.06E-04	2.84E-52	-0.657
cg03087507	19	12076234	*ZNF763*	5'UTR/1st intron	Island	0.319±0.065	0.454±0.197	-0.134	1.66E-02	5.57E-30	-0.520
cg06909469	2	96990800	*ITPRIPL1*	TSS1500	N_Shore	0.26±0.075	0.394±0.171	-0.134	1.07E-02	8.22E-15	-0.370
cg19165274	10	4868328	*AKR1E2*	TSS200	Island	0.485±0.071	0.619±0.119	-0.134	1.80E-03	2.18E-18	-0.413
cg08357436	17	56565641	*HSF5*	5'UTR/1st exon	Island	0.765±0.059	0.899±0.031	-0.134	2.06E-04	5.13E-07	-0.244
cg26971042	15	70387217	*TLE3*	TSS200	N_Shore	0.351±0.09	0.485±0.196	-0.134	2.52E-02	5.85E-09	-0.282
cg26683259	5	77943417	*LHFPL2*	5'UTR/1st intron	N_Shore	0.149±0.084	0.283±0.201	-0.134	2.45E-02	4.46E-07	-0.246
cg27379065	19	20011538	*ZNF93*	TSS200	N_Shore	0.121±0.031	0.255±0.145	-0.134	1.34E-03	3.65E-06	-0.226
cg24251890	11	47290825	*MADD*	TSS1500	N_Shore	0.141±0.123	0.275±0.187	-0.134	4.31E-02	7.67E-08	-0.261
cg03736062	4	113208476	*TIFA*	TSS1500	S_Shore	0.763±0.083	0.896±0.02	-0.133	2.56E-03	1.29E-08	-0.276
cg15354065	12	56327217	*DGKA*	5'UTR/1st intron	S_Shore	0.548±0.082	0.681±0.098	-0.133	2.39E-03	1.94E-08	-0.272
cg25324068	17	7462249	*TNFSF13*	1st exon	N_Shelf	0.537±0.067	0.67±0.061	-0.133	3.89E-04	3.57E-26	-0.489
cg12640469	1	53067717	*GPX7*	TSS1500	N_Shore	0.381±0.067	0.514±0.187	-0.133	1.39E-02	3.35E-26	-0.489
cg00316759	15	71407484	*CT62*	5'UTR/1st exon	N_Shore	0.485±0.094	0.618±0.212	-0.133	3.71E-02	1.59E-07	-0.255
cg10782923	12	56329731	*DGKA*	1st exon	S_Shelf	0.649±0.084	0.781±0.07	-0.132	2.21E-03	5.77E-09	-0.282
cg02039839	7	1015730	*COX19*	TSS1500	S_Shore	0.47±0.063	0.603±0.141	-0.132	2.91E-03	2.32E-06	-0.230
cg07783282	12	95942964	*USP44*	5'UTR/1st intron	Island	0.508±0.133	0.64±0.154	-0.132	4.10E-02	6.44E-09	-0.281
cg10177528	1	211499638	*TRAF5*	TSS1500	N_Shore	0.65±0.098	0.782±0.072	-0.132	6.02E-03	3.44E-11	-0.319
cg25331703	15	101459283	*LRRK1*	TSS200	Island	0.425±0.085	0.557±0.145	-0.132	8.19E-03	3.29E-06	-0.227
cg04500986	1	6526531	*TNFRSF25*	TSS1500	S_Shore	0.62±0.099	0.752±0.085	-0.132	6.64E-03	5.92E-30	-0.520
cg11683242	1	32716557	*LCK*	TSS1500	S_Shelf	0.745±0.092	0.877±0.03	-0.132	4.42E-03	5.85E-73	-0.741
cg22096687	11	87908817	*RAB38*	TSS200	S_Shore	0.461±0.093	0.593±0.146	-0.132	1.16E-02	8.76E-22	-0.449
cg10579718	1	1052886	*C1orf159*	TSS1500	S_Shore	0.687±0.077	0.819±0.08	-0.132	1.32E-03	1.86E-10	-0.307
cg00808648	14	105779910	*PACS2*	5'UTR/1st intron	N_Shore	0.633±0.113	0.765±0.084	-0.132	1.33E-02	5.02E-06	-0.223
cg11953272	1	53067965	*GPX7*	TSS200	Island	0.218±0.091	0.35±0.148	-0.132	1.15E-02	6.92E-32	-0.535
cg00730820	1	27694390	*MAP3K6*	TSS1500	S_Shore	0.546±0.048	0.677±0.175	-0.132	7.44E-03	1.92E-06	-0.232
cg26122129	8	30241119	*RBPMS*	TSS1500	N_Shore	0.606±0.141	0.738±0.116	-0.132	3.97E-02	3.72E-08	-0.267
cg22555539	7	43798090	*BLVRA*	TSS200	Island	0.057±0.072	0.189±0.223	-0.132	3.45E-02	2.67E-08	-0.270
cg22174486	11	536817	*LRRC56*	TSS1500	Island	0.378±0.113	0.509±0.143	-0.132	2.24E-02	7.54E-07	-0.241
cg24664689	8	11058690	*XKR6*	1st exon	S_Shore	0.43±0.13	0.562±0.16	-0.131	4.11E-02	1.13E-09	-0.294
cg20886263	3	53196104	*PRKCD*	5'UTR/1st intron	Island	0.113±0.036	0.245±0.195	-0.131	1.23E-02	9.21E-24	-0.468
cg04800681	22	18594235	*TUBA8*	5'UTR/1st intron	S_Shore	0.477±0.092	0.608±0.153	-0.131	1.33E-02	8.42E-11	-0.313
cg20389653	1	16766715	*NECAP2*	TSS1500	N_Shore	0.728±0.106	0.859±0.065	-0.131	9.39E-03	2.41E-05	-0.206
cg00496004	11	130029481	*ST14*	TSS200	N_Shore	0.361±0.132	0.492±0.128	-0.131	3.48E-02	1.42E-18	-0.415
cg24395117	11	70245893	*CTTN*	5'UTR/1st intron	S_Shore	0.596±0.114	0.727±0.041	-0.131	1.35E-02	6.55E-07	-0.242
cg08896939	17	29297380	*RNF135*	TSS1500	N_Shore	0.696±0.07	0.827±0.096	-0.131	1.00E-03	5.77E-19	-0.419
cg22903300	6	29760765	*HCG4*	1st exon	Island	0.046±0.011	0.177±0.197	-0.131	1.20E-02	4.95E-06	-0.223
cg04118190	2	96812528	*DUSP2*	TSS1500	S_Shore	0.57±0.099	0.701±0.115	-0.131	9.50E-03	3.78E-11	-0.318
cg05675373	1	110754257	*KCNC4*	1st exon	Island	0.483±0.077	0.614±0.133	-0.131	4.67E-03	2.45E-05	-0.206
cg17108629	11	87908805	*RAB38*	TSS200	S_Shore	0.429±0.098	0.56±0.17	-0.131	2.19E-02	1.92E-13	-0.352
cg15641364	1	159892206	*TAGLN2*	5'UTR/1st intron	N_Shelf	0.462±0.104	0.593±0.184	-0.131	3.13E-02	1.40E-10	-0.309
cg05798318	11	536758	*LRRC56*	TSS1500	Island	0.453±0.14	0.584±0.146	-0.130	4.82E-02	2.84E-07	-0.250
cg00523161	2	11888753	*LPIN1*	5'UTR/1st exon	S_Shore	0.488±0.122	0.618±0.123	-0.130	2.56E-02	7.40E-20	-0.429
cg11436767	11	46401422	*MDK*	TSS1500	N_Shore	0.526±0.092	0.656±0.093	-0.130	5.16E-03	2.43E-16	-0.389
cg04241075	1	46807263	*NSUN4*	5'UTR/1st exon	S_Shore	0.518±0.061	0.648±0.114	-0.130	9.84E-04	8.45E-06	-0.217
cg22319311	8	98290310	*TSPYL5*	TSS200	Island	0.53±0.07	0.659±0.059	-0.130	6.63E-04	2.34E-13	-0.351
cg21237591	11	2162510	*IGF2*	5'UTR	S_Shore	0.503±0.096	0.633±0.158	-0.130	1.78E-02	3.93E-09	-0.285

cg14630839	22	50919823	*ADM2*	TSS1500	Island	0.042±0.042	0.172±0.195	-0.130	1.43E-02	2.37E-05	-0.207
cg03312716	12	110271581	*TRPV4*	TSS1500	Island	0.473±0.118	0.603±0.125	-0.130	2.36E-02	3.52E-06	-0.226
cg24845234	7	23509610	*IGF2BP3*	1st exon	Island	0.334±0.08	0.463±0.16	-0.129	1.14E-02	7.75E-12	-0.329
cg08014499	11	2162545	*IGF2*	5'UTR	S_Shore	0.582±0.124	0.711±0.158	-0.129	3.83E-02	6.08E-12	-0.330
cg26570683	19	11689860	*ACP5*	TSS1500	S_Shore	0.351±0.068	0.48±0.156	-0.129	7.15E-03	1.23E-20	-0.437
cg02233614	1	207227965	*PFKFB2*	5'UTR/1st intron	S_Shelf	0.659±0.087	0.789±0.071	-0.129	3.50E-03	4.26E-08	-0.266
cg17177816	10	96304193	*HELLS*	TSS1500	N_Shore	0.738±0.137	0.867±0.095	-0.129	3.60E-02	6.70E-10	-0.298
cg02680487	6	30851529	*DDR1*	5'UTR/1st intron	N_Shore	0.49±0.137	0.619±0.138	-0.129	4.49E-02	1.52E-19	-0.425
cg02317321	10	15131036	*ACBD7*	TSS1500	S_Shore	0.195±0.114	0.324±0.181	-0.129	3.93E-02	1.73E-06	-0.233
cg06942110	7	1545385	*INTS1*	TSS1500	S_Shore	0.498±0.072	0.627±0.122	-0.129	2.92E-03	2.97E-07	-0.249
cg04619185	22	45706476	*FAM118A*	5'UTR	Island	0.106±0.079	0.235±0.166	-0.129	1.32E-02	9.20E-06	-0.217
cg09119665	14	74181285	*PNMA1*	TSS200	S_Shore	0.13±0.091	0.259±0.182	-0.129	2.47E-02	7.14E-16	-0.383
cg24006126	1	155290813	*RUSC1*	5'UTR/1st intron	N_Shore	0.454±0.121	0.583±0.121	-0.129	2.61E-02	2.00E-05	-0.208
cg19680850	1	37943106	*ZC3H12A*	5'UTR	S_Shelf	0.111±0.056	0.24±0.12	-0.129	1.04E-03	2.88E-06	-0.228
cg04481779	6	137366125	*IL20RA*	5'UTR/1st exon	Island	0.575±0.128	0.704±0.163	-0.129	4.45E-02	5.28E-15	-0.372
cg18661868	15	91427965	*FES*	5'UTR/1st intron	S_Shelf	0.275±0.092	0.404±0.169	-0.129	2.01E-02	9.53E-40	-0.588
cg05564831	3	52568323	*NT5DC2*	TSS1500	Island	0.477±0.085	0.606±0.162	-0.129	1.45E-02	2.98E-16	-0.388
cg06460568	11	2162211	*IGF2*	5'UTR	Island	0.576±0.024	0.704±0.097	-0.129	3.12E-05	7.53E-07	-0.241
cg20459126	3	142443247	*TRPC1*	TSS200	Island	0.081±0.095	0.21±0.219	-0.128	4.78E-02	1.86E-07	-0.253
cg14269096	18	56532433	*ZNF532*	5'UTR/1st intron	S_Shore	0.597±0.119	0.725±0.088	-0.128	2.01E-02	4.11E-10	-0.302
cg25535587	10	82116456	*DYDC1*	5'UTR/1st intron	Island	0.643±0.088	0.771±0.186	-0.128	2.54E-02	1.97E-14	-0.365
cg25537993	19	58545182	*ZSCAN1*	TSS1500	Island	0.635±0.098	0.763±0.098	-0.128	8.43E-03	3.64E-07	-0.247
cg03127244	6	33245638	*B3GALT4*	1st exon	S_Shore	0.684±0.087	0.812±0.034	-0.128	3.81E-03	4.65E-06	-0.223
cg19688887	19	20150441	*ZNF682*	TSS1500	S_Shore	0.037±0.009	0.165±0.193	-0.128	1.21E-02	3.04E-07	-0.249
cg13660174	9	136238392	*SURF4*	5'UTR	N_Shore	0.596±0.097	0.724±0.081	-0.128	7.22E-03	2.53E-07	-0.251
cg18397882	19	19050971	*HOMER3*	5'UTR/1st exon	N_Shore	0.18±0.093	0.308±0.168	-0.128	2.10E-02	2.57E-08	-0.270
cg01131735	17	39968419	*FKBP10*	TSS1500	Island	0.086±0.088	0.213±0.216	-0.128	4.28E-02	4.08E-06	-0.225
cg07634645	12	14720938	*PLBD1*	TSS200	Island	0.471±0.131	0.599±0.162	-0.128	4.87E-02	6.79E-20	-0.429
cg13951491	1	45793032	*HPDL*	1st exon	Island	0.476±0.056	0.604±0.064	-0.128	1.24E-04	1.28E-07	-0.257
cg06991495	22	38714466	*CSNK1E*	TSS1500	S_Shore	0.499±0.088	0.627±0.088	-0.127	4.42E-03	1.24E-17	-0.404
cg00246451	1	155949012	*ARHGEF2*	5'UTR/1st exon	S_Shore	0.418±0.126	0.545±0.167	-0.127	4.60E-02	1.26E-14	-0.368
cg04413069	12	9800446	*LOC374443*	TSS200	N_Shore	0.11±0.086	0.237±0.18	-0.127	2.27E-02	6.44E-07	-0.242
cg04193820	17	29297414	*RNF135*	TSS1500	N_Shore	0.681±0.066	0.808±0.149	-0.127	5.85E-03	2.72E-15	-0.376
cg15026998	2	160761622	*LY75*	TSS1500	S_Shore	0.362±0.109	0.489±0.181	-0.127	3.84E-02	2.21E-14	-0.364
cg26299148	15	41522672	*EXD1*	5'UTR/1st intron	N_Shore	0.696±0.074	0.823±0.098	-0.127	1.92E-03	1.44E-06	-0.235
cg12090942	14	77279508	*ANGEL1*	TSS1500	S_Shore	0.587±0.066	0.714±0.086	-0.127	7.25E-04	1.27E-05	-0.213
cg06733329	5	176740039	*MXD3*	TSS1500	S_Shore	0.186±0.1	0.312±0.129	-0.127	1.47E-02	1.41E-11	-0.325
cg03131767	12	123446272	*ABCB9*	5'UTR/1st intron	S_Shore	0.638±0.087	0.765±0.123	-0.127	7.42E-03	1.59E-08	-0.274
cg06159340	16	30907560	*CTF1*	TSS1500	S_Shore	0.688±0.085	0.814±0.041	-0.127	3.39E-03	2.70E-31	-0.531
cg26811705	11	118781408	*BCL9L*	5'UTR/1st exon	Island	0.465±0.105	0.591±0.204	-0.126	4.94E-02	2.65E-08	-0.270
cg10193721	14	24780691	*LTB4R*	TSS200	Island	0.37±0.092	0.496±0.181	-0.126	2.76E-02	1.67E-09	-0.291
cg04728789	22	38711154	*CSNK1E*	5'UTR/1st intron	N_Shore	0.516±0.061	0.642±0.102	-0.126	8.21E-04	1.39E-09	-0.293
cg22536775	1	1851439	*TMEM52*	TSS1500	S_Shore	0.479±0.088	0.604±0.132	-0.125	1.02E-02	4.13E-09	-0.284
cg08032476	17	27045176	*RAB34*	1st exon	N_Shore	0.562±0.057	0.687±0.147	-0.125	4.56E-03	6.79E-26	-0.487
cg06303238	20	50418959	*SALL4*	5'UTR/1st exon	Island	0.734±0.12	0.859±0.056	-0.125	2.14E-02	4.79E-20	-0.431
cg04138502	3	123167522	*ADCY5*	TSS200	Island	0.46±0.099	0.585±0.079	-0.125	9.03E-03	8.67E-11	-0.313
cg10101634	3	32432642	*CMTM7*	TSS1500	N_Shore	0.59±0.073	0.715±0.084	-0.125	1.56E-03	7.91E-08	-0.261
cg19301742	6	33421180	*ZBTB9*	TSS1500	N_Shore	0.196±0.054	0.321±0.141	-0.125	3.42E-03	2.13E-05	-0.208
cg08220696	17	61820713	*STRADA*	TSS1500	S_Shore	0.76±0.146	0.885±0.075	-0.125	4.94E-02	9.19E-06	-0.217
cg04303330	5	131992430	*IL13*	5'UTR/1st intron	S_Shore	0.646±0.064	0.771±0.059	-0.125	4.68E-04	2.73E-09	-0.288
cg24427376	1	78354053	*NEXN*	TSS200	N_Shore	0.472±0.089	0.597±0.137	-0.124	1.20E-02	1.24E-15	-0.380
cg11724055	1	23667475	*HNRNPR*	5'UTR/1st intron	N_Shelf	0.495±0.101	0.62±0.166	-0.124	2.85E-02	6.12E-06	-0.221
cg23238834	6	31126879	*TCF19*	5'UTR	S_Shore	0.129±0.058	0.253±0.169	-0.124	1.08E-02	5.55E-16	-0.385
cg23238834	6	31126879	*CCHCR1*	TSS1500	S_Shore	0.129±0.058	0.253±0.169	-0.124	1.08E-02	4.46E-06	-0.224
cg14389924	17	4606729	*PELP1*	5'UTR/1st intron	N_Shore	0.374±0.101	0.499±0.193	-0.124	4.25E-02	4.71E-06	-0.223
cg13980266	9	97022269	*ZNF169*	5'UTR/1st intron	S_Shore	0.831±0.143	0.955±0.024	-0.124	4.36E-02	1.23E-09	-0.294
cg19443075	11	2161403	*IGF2*	5'UTR	Island	0.541±0.074	0.665±0.077	-0.124	1.62E-03	4.67E-13	-0.346
cg19702703	4	7942020	*AFAP1*	TSS1500	S_Shore	0.647±0.122	0.771±0.138	-0.124	3.61E-02	4.86E-16	-0.385
cg08539991	19	36203832	*ZBTB32*	5'UTR	N_Shelf	0.536±0.094	0.66±0.162	-0.124	2.30E-02	9.98E-10	-0.295
cg00352043	22	38863594	*KDELR3*	TSS1500	N_Shore	0.169±0.103	0.292±0.196	-0.123	4.73E-02	9.23E-06	-0.217
cg11509907	2	235404686	*ARL4C*	1st exon	Island	0.316±0.094	0.439±0.162	-0.123	2.36E-02	4.43E-09	-0.284
cg06032349	21	40032331	*ERG*	5'UTR/1st intron	Island	0.457±0.105	0.58±0.111	-0.123	1.64E-02	1.06E-12	-0.341
cg04369964	11	63997765	*DNAJC4*	TSS1500	N_Shore	0.136±0.051	0.259±0.132	-0.123	2.18E-03	5.77E-14	-0.359
cg18347630	20	39765163	*PLCG1*	TSS1500	N_Shore	0.529±0.064	0.652±0.082	-0.123	7.17E-04	1.06E-07	-0.258
cg27506082	2	207139431	*ZDBF2*	5'UTR/1st exon	Island	0.323±0.065	0.446±0.176	-0.123	1.61E-02	3.22E-10	-0.303
cg14293575	22	18635460	*USP18*	5'UTR/1st intron	S_Shelf	0.772±0.113	0.895±0.023	-0.123	1.75E-02	1.00E-08	-0.278
cg23837265	10	88730407	*AGAP11*	TSS200	S_Shore	0.592±0.116	0.715±0.156	-0.123	3.92E-02	1.72E-05	-0.210
cg05544396	16	66959780	*RRAD*	TSS1500	S_Shelf	0.458±0.064	0.581±0.182	-0.123	1.86E-02	6.37E-08	-0.263
cg03802998	11	44972744	*TP53I11*	5'UTR/1st exon	S_Shore	0.497±0.097	0.62±0.132	-0.123	1.64E-02	1.96E-05	-0.209
cg25926515	10	70321889	*TET1*	5'UTR/1st intron	S_Shore	0.598±0.077	0.721±0.147	-0.122	1.08E-02	9.15E-08	-0.259
cg08227353	10	102821670	*KAZALD1*	1st exon	Island	0.601±0.092	0.723±0.174	-0.122	2.86E-02	3.39E-57	-0.680
cg13935127	1	41128874	*RIMS3*	5'UTR/1st intron	N_Shelf	0.731±0.083	0.854±0.041	-0.122	3.63E-03	3.14E-05	-0.204
cg02990598	19	1955419	*CSNK1G2*	5'UTR/1st intron	S_Shore	0.583±0.083	0.705±0.117	-0.122	6.93E-03	9.90E-14	-0.356
cg02835822	11	2161456	*IGF2*	5'UTR	Island	0.491±0.08	0.614±0.173	-0.122	2.16E-02	3.42E-05	-0.203
cg09826895	1	111744309	*DENND2D*	TSS1500	N_Shelf	0.69±0.096	0.812±0.079	-0.122	8.82E-03	1.46E-16	-0.392
cg16361890	5	179220545	*LTC4S*	TSS1500	N_Shelf	0.628±0.077	0.75±0.057	-0.122	2.27E-03	3.20E-08	-0.268
cg22341471	12	123451323	*ABCB9*	TSS1500	S_Shore	0.343±0.112	0.465±0.143	-0.122	3.14E-02	6.53E-12	-0.330
cg19914904	10	102756165	*LZTS2*	TSS1500	N_Shore	0.601±0.085	0.722±0.123	-0.122	8.87E-03	5.61E-07	-0.244
cg10322794	21	47740892	*C21orf58*	5'UTR/1st intron	N_Shore	0.575±0.098	0.696±0.131	-0.122	1.72E-02	6.32E-07	-0.242
cg25314360	6	658460	*HUS1B*	TSS1500	S_Shore	0.685±0.056	0.807±0.058	-0.122	1.66E-04	8.79E-06	-0.217
cg17338403	15	92395836	*SLCO3A1*	TSS1500	N_Shore	0.174±0.049	0.296±0.136	-0.122	2.74E-03	7.10E-15	-0.371
cg02945646	14	24037169	*AP1G2*	TSS1500	S_Shore	0.208±0.071	0.33±0.185	-0.121	2.36E-02	2.09E-13	-0.351
cg27230534	3	195267087	*PPP1R2*	5'UTR/1st intron	N_Shelf	0.679±0.079	0.801±0.025	-0.121	3.04E-03	9.05E-17	-0.394
cg17969560	11	65324768	*LTBP3*	5'UTR/1st intron	N_Shore	0.585±0.126	0.706±0.136	-0.121	4.34E-02	4.48E-23	-0.461
cg14993712	7	138720786	*ZC3HAV1L*	TSS200	Island	0.426±0.13	0.547±0.094	-0.121	3.81E-02	4.18E-17	-0.398
cg17103217	6	33245721	*B3GALT4*	1st exon	S_Shore	0.489±0.057	0.611±0.092	-0.121	5.30E-04	4.43E-08	-0.266
cg11432797	16	29674487	*SPN*	5'UTR/1st intron	N_Shore	0.456±0.119	0.577±0.131	-0.121	3.47E-02	5.54E-07	-0.244

cg14242936	12	52404134	*GRASP*	TSS200	S_Shelf	0.645±0.082	0.766±0.047	-0.121	3.67E-03	6.77E-33	-0.542
cg04472726	8	41386353	*GINS4*	TSS1500	N_Shore	0.164±0.073	0.285±0.191	-0.121	2.87E-02	1.43E-05	-0.212
cg11159295	3	52280139	*PPM1M*	TSS200	Island	0.142±0.087	0.262±0.176	-0.121	2.80E-02	8.32E-23	-0.458
cg20116128	6	32161004	*GPSM3*	5'UTR	N_Shelf	0.689±0.03	0.81±0.029	-0.121	3.20E-07	1.68E-20	-0.436
cg05605377	18	47017768	*RPL17*	5'UTR	S_Shelf	0.676±0.102	0.797±0.111	-0.121	1.61E-02	1.60E-22	-0.456
cg10575089	17	56606542	*SEPT4*	1st exon	N_Shelf	0.519±0.066	0.64±0.093	-0.121	1.26E-03	1.47E-11	-0.325
cg05819371	12	92540484	*BTG1*	TSS1500	S_Shore	0.107±0.087	0.228±0.156	-0.121	1.95E-02	1.63E-06	-0.234
cg25232510	8	134306223	*NDRG1*	5'UTR/1st intron	N_Shelf	0.762±0.124	0.882±0.078	-0.120	3.04E-02	8.49E-24	-0.468
cg02241986	10	5566543	*CALML3*	TSS1500	N_Shore	0.435±0.079	0.555±0.112	-0.120	5.38E-03	1.04E-06	-0.238
cg18593194	19	36205201	*ZBTB32*	5'UTR	N_Shore	0.652±0.088	0.772±0.119	-0.120	1.00E-02	1.74E-06	-0.233
cg13575298	3	170626725	*EIF5A2*	TSS1500	Island	0.64±0.085	0.76±0.148	-0.120	1.59E-02	1.87E-18	-0.414
cg17629322	12	131355424	*RAN*	TSS1500	N_Shore	0.734±0.074	0.854±0.038	-0.120	2.04E-03	2.23E-13	-0.351
cg04207413	10	126431523	*FAM53B*	5'UTR/1st intron	Island	0.162±0.112	0.282±0.147	-0.120	3.49E-02	1.02E-05	-0.215
cg07583420	11	2158555	*IGF2*	5'UTR	N_Shore	0.073±0.036	0.193±0.137	-0.120	2.29E-03	1.28E-05	-0.213
cg14349538	7	128577651	*IRF5*	5'UTR/1st intron	N_Shore	0.424±0.095	0.544±0.149	-0.120	2.24E-02	5.23E-10	-0.300
cg26661436	5	141031071	*FCHSD1*	TSS200	Island	0.21±0.087	0.329±0.158	-0.120	2.10E-02	5.07E-16	-0.385
cg07602073	1	228595680	*TRIM11*	TSS1500	S_Shore	0.731±0.089	0.851±0.027	-0.120	6.48E-03	5.36E-09	-0.282
cg02144874	2	239047336	*KLHL30*	TSS200	N_Shelf	0.666±0.075	0.786±0.069	-0.120	2.25E-03	1.37E-12	-0.340
cg18829411	21	40722023	*HMGN1*	TSS1500	S_Shore	0.573±0.104	0.692±0.151	-0.119	3.08E-02	4.88E-08	-0.265
cg02272599	19	11267029	*SPC24*	TSS1500	S_Shore	0.189±0.048	0.308±0.18	-0.119	1.64E-02	3.69E-05	-0.202
cg20321801	11	2404384	*CD81*	5'UTR/1st intron	N_Shelf	0.48±0.069	0.599±0.085	-0.119	1.64E-03	1.94E-06	-0.232
cg08468370	19	39903678	*PLEKHG2*	TSS1500	Island	0.087±0.075	0.206±0.174	-0.119	2.26E-02	3.92E-08	-0.267
cg21175642	3	48701770	*CELSR3*	TSS1500	S_Shore	0.156±0.053	0.275±0.188	-0.119	2.17E-02	9.33E-13	-0.342
cg19667304	12	68043875	*DYRK2*	5'UTR/1st intron	Island	0.075±0.075	0.194±0.186	-0.119	2.90E-02	1.48E-16	-0.392
cg25942450	5	170736251	*TLX3*	TSS200	Island	0.512±0.094	0.631±0.152	-0.119	2.41E-02	8.89E-06	-0.217
cg23508786	21	34775291	*IFNGR2*	5'UTR/1st exon	N_Shore	0.286±0.115	0.404±0.166	-0.119	4.89E-02	7.51E-07	-0.241
cg11973623	6	29760832	*HCG4*	1st exon	Island	0.131±0.094	0.249±0.19	-0.118	4.50E-02	2.57E-08	-0.270
cg04226536	11	111411071	*LAYN*	5'UTR/1st intron	Island	0.535±0.073	0.653±0.076	-0.118	2.04E-03	8.48E-08	-0.260
cg07827420	17	75371764	*SEPT9*	5'UTR	S_Shore	0.665±0.061	0.783±0.037	-0.118	5.98E-04	6.87E-10	-0.298
cg23673107	19	11908712	*ZNF491*	TSS1500	N_Shore	0.365±0.101	0.483±0.114	-0.118	1.81E-02	6.30E-06	-0.220
cg18568589	19	9609395	*ZNF560*	TSS200	S_Shore	0.572±0.127	0.69±0.115	-0.118	4.32E-02	1.32E-07	-0.256
cg17641710	3	50279038	*GNAI2*	5'UTR/1st intron	S_Shelf	0.683±0.05	0.801±0.038	-0.118	9.60E-05	1.97E-07	-0.253
cg14044426	6	32160798	*GPSM3*	5'UTR	N_Shelf	0.613±0.052	0.73±0.061	-0.118	1.25E-04	5.52E-13	-0.345
cg03098721	1	84464084	*TTLL7*	5'UTR/1st intron	N_Shore	0.543±0.081	0.661±0.203	-0.118	4.62E-02	1.61E-08	-0.274
cg14556683	19	15342982	*EPHX3*	1st exon	N_Shore	0.603±0.035	0.72±0.067	-0.117	5.34E-06	2.71E-09	-0.288
cg10416593	22	50966123	*SCO2*	TSS1500	Island	0.659±0.084	0.776±0.106	-0.117	7.91E-03	8.08E-11	-0.313
cg16534854	5	170815995	*NPM1*	5'UTR/1st intron	S_Shore	0.029±0.019	0.146±0.222	-0.117	3.97E-02	9.84E-07	-0.238
cg19713429	1	19810690	*CAPZB*	1st exon	N_Shore	0.306±0.09	0.423±0.186	-0.117	4.15E-02	1.40E-09	-0.293
cg11507178	11	118781515	*BCL9L*	5'UTR/1st exon	Island	0.17±0.074	0.287±0.153	-0.117	1.53E-02	3.91E-06	-0.225
cg02633117	19	17187589	*MYO9B*	5'UTR/1st intron	S_Shore	0.175±0.065	0.292±0.181	-0.117	2.41E-02	3.75E-09	-0.285
cg02633117	19	17187589	*HAUS8*	TSS1500	S_Shore	0.175±0.065	0.292±0.181	-0.117	2.41E-02	1.30E-05	-0.213
cg10512278	16	68027839	*DPEP2*	5'UTR/1st intron	S_Shore	0.685±0.048	0.802±0.029	-0.117	1.01E-04	4.18E-26	-0.489
cg21703322	11	111411076	*LAYN*	5'UTR/1st intron	Island	0.547±0.063	0.664±0.081	-0.116	9.55E-04	7.49E-08	-0.261
cg02516189	9	139268092	*CARD9*	5'UTR/1st exon	S_Shelf	0.447±0.103	0.564±0.069	-0.116	1.56E-02	2.93E-12	-0.335
cg00747290	2	42721154	*KCNG3*	5'UTR/1st exon	Island	0.342±0.102	0.458±0.139	-0.116	2.87E-02	1.29E-06	-0.236
cg23906687	2	239047029	*KLHL30*	TSS1500	N_Shelf	0.603±0.082	0.719±0.1	-0.116	6.56E-03	3.94E-05	-0.201
cg19628988	5	139040928	*CXXC5*	5'UTR/1st intron	Island	0.668±0.095	0.784±0.129	-0.116	1.99E-02	4.11E-05	-0.201
cg15146125	2	96991753	*ITPRIPL1*	5'UTR/1st intron	S_Shore	0.318±0.046	0.434±0.156	-0.116	8.61E-03	4.33E-10	-0.301
cg01109047	2	120979401	*TMEM185B*	1st exon/3'UTR	N_Shore	0.767±0.058	0.882±0.046	-0.115	4.08E-04	2.12E-08	-0.272
cg27075724	21	18985387	*BTG3*	TSS200	Island	0.039±0.019	0.154±0.19	-0.115	2.12E-02	3.42E-05	-0.203
cg15166332	10	35929604	*FZD8*	1st exon	Island	0.102±0.112	0.217±0.158	-0.115	4.92E-02	8.40E-08	-0.260
cg21800196	3	48673931	*SLC26A6*	TSS1500	N_Shelf	0.663±0.099	0.777±0.046	-0.114	1.35E-02	2.81E-15	-0.376
cg15747595	8	98289880	*TSPYL5*	1st exon	Island	0.589±0.088	0.704±0.093	-0.114	9.46E-03	2.86E-12	-0.335
cg05117208	17	40438312	*STAT5A*	TSS1500	N_Shore	0.74±0.062	0.855±0.056	-0.114	7.51E-04	4.76E-21	-0.441
cg10132208	19	58545542	*ZSCAN1*	5'UTR/1st intron	Island	0.559±0.04	0.673±0.071	-0.114	3.04E-05	3.38E-07	-0.248
cg12411858	22	20863268	*MED15*	5'UTR/1st intron	S_Shore	0.674±0.035	0.788±0.057	-0.114	3.66E-06	4.51E-16	-0.386
cg07856975	6	36356162	*ETV7*	TSS1500	S_Shore	0.724±0.118	0.838±0.101	-0.114	3.58E-02	3.53E-16	-0.387
cg17476421	19	19844057	*ZNF14*	TSS200	S_Shore	0.072±0.035	0.186±0.144	-0.114	4.91E-03	5.05E-12	-0.331
cg03760951	11	2160875	*IGF2*	5'UTR	Island	0.578±0.07	0.692±0.15	-0.114	1.46E-02	8.55E-22	-0.449
cg09747445	15	70387268	*TLE3*	TSS200	N_Shore	0.705±0.059	0.819±0.037	-0.114	5.67E-04	3.86E-06	-0.225
cg18731055	13	49108131	*RCBTB2*	TSS1500	S_Shore	0.659±0.127	0.772±0.055	-0.113	4.05E-02	7.54E-06	-0.219
cg14871225	5	139040820	*CXXC5*	5'UTR/1st intron	Island	0.592±0.078	0.706±0.113	-0.113	7.93E-03	3.26E-07	-0.248
cg05355142	1	1851372	*TMEM52*	TSS1500	Island	0.384±0.069	0.497±0.185	-0.113	3.28E-02	1.50E-05	-0.212
cg18669588	3	52274051	*TWF2*	TSS1500	S_Shore	0.632±0.078	0.745±0.103	-0.113	6.63E-03	3.57E-06	-0.226
cg13766044	17	39968772	*FKBP10*	TSS200	S_Shore	0.103±0.04	0.216±0.106	-0.113	6.14E-04	7.75E-10	-0.297
cg20582800	6	43612764	*RSPH9*	TSS200	Island	0.53±0.064	0.643±0.065	-0.113	1.03E-03	3.75E-05	-0.202
cg10219957	1	1851416	*TMEM52*	TSS1500	S_Shore	0.644±0.094	0.757±0.114	-0.113	1.80E-02	3.82E-11	-0.318
cg15286372	6	53659348	*LRRC1*	TSS1500	Island	0.076±0.036	0.189±0.203	-0.113	3.48E-02	2.30E-07	-0.252
cg12485428	17	1549098	*SCARF1*	TSS200	S_Shore	0.607±0.063	0.72±0.037	-0.112	1.06E-03	6.29E-12	-0.330
cg07351675	7	1578263	*MAFK*	5'UTR/1st intron	S_Shelf	0.834±0.082	0.946±0.026	-0.112	6.01E-03	1.72E-05	-0.210
cg01834776	14	24898905	*KHNYN*	TSS1500	Island	0.219±0.102	0.331±0.149	-0.112	3.83E-02	1.63E-06	-0.234
cg20339650	11	2160882	*IGF2*	5'UTR	Island	0.486±0.096	0.598±0.167	-0.112	4.15E-02	7.57E-19	-0.418
cg21693907	1	203595061	*ATP2B4*	TSS1500	N_Shelf	0.176±0.087	0.288±0.112	-0.112	1.28E-02	1.36E-06	-0.235
cg25410668	1	28241577	*RPA2*	TSS1500	S_Shore	0.691±0.125	0.803±0.075	-0.112	4.19E-02	1.22E-06	-0.236
cg17283169	10	5567524	*CALML3*	1st exon/3'UTR	Island	0.789±0.107	0.9±0.03	-0.111	2.15E-02	1.19E-46	-0.628
cg22678398	3	152884214	*RAP2B*	1st exon/3'UTR	S_Shelf	0.703±0.058	0.815±0.038	-0.111	6.27E-04	1.88E-10	-0.307
cg12306853	6	143833173	*FUCA2*	TSS200	S_Shore	0.653±0.121	0.764±0.12	-0.111	4.88E-02	7.25E-15	-0.371
cg03457485	11	63753251	*OTUB1*	TSS200	N_Shore	0.346±0.102	0.457±0.163	-0.111	4.68E-02	1.04E-07	-0.258
cg26476820	2	85637851	*CAPG*	5'UTR/1st intron	N_Shelf	0.707±0.073	0.818±0.077	-0.111	3.41E-03	2.41E-12	-0.336
cg00591660	16	2478353	*CCNF*	TSS1500	N_Shore	0.654±0.077	0.765±0.062	-0.111	4.24E-03	1.61E-09	-0.292
cg21534299	1	44821474	*ERI3*	TSS1500	S_Shore	0.738±0.115	0.848±0.031	-0.111	2.99E-02	7.98E-08	-0.261
cg09961550	16	21531948	*SLC7A5P2*	TSS200	Island	0.402±0.124	0.512±0.104	-0.110	4.95E-02	3.89E-08	-0.267
cg25923056	19	41306455	*EGLN2*	5'UTR	S_Shore	0.55±0.042	0.66±0.125	-0.110	2.76E-03	9.28E-06	-0.216
cg19429281	19	53496738	*ZNF702P*	1st exon	Island	0.625±0.077	0.735±0.168	-0.110	3.11E-02	2.38E-21	-0.444
cg05280131	2	132248998	*LOC150776*	TSS1500	N_Shore	0.331±0.106	0.441±0.151	-0.110	4.64E-02	4.08E-08	-0.266
cg01745499	6	34206084	*HMGA1*	5'UTR	Island	0.146±0.056	0.256±0.19	-0.110	3.53E-02	1.45E-14	-0.367
cg14843030	1	32716051	*LCK*	TSS1500	S_Shore	0.441±0.079	0.551±0.087	-0.110	6.42E-03	5.75E-08	-0.263

cg18081258	14	21494161	*NDRG2*	5'UTR/1st intron	Island	0.542±0.06	0.652±0.202	-0.110	4.62E-02	2.82E-26	-0.490
cg11278602	6	29760757	*HCG4*	1st exon	Island	0.072±0.015	0.182±0.146	-0.110	6.95E-03	1.59E-06	-0.234
cg05921324	11	116694114	*APOA4*	TSS200	S_Shore	0.563±0.089	0.673±0.109	-0.110	1.51E-02	3.12E-05	-0.204
cg15026089	1	19640712	*PQLC2*	5'UTR	S_Shore	0.698±0.049	0.808±0.041	-0.110	1.41E-04	4.98E-10	-0.300
cg01357925	6	53658617	*LRRC1*	TSS1500	N_Shore	0.017±0.005	0.127±0.164	-0.110	1.14E-02	6.84E-06	-0.220
cg07549715	20	3024204	*GNRH2*	TSS200	N_Shelf	0.623±0.081	0.732±0.065	-0.110	5.91E-03	4.68E-10	-0.301
cg16377367	1	85743497	*BCL10*	TSS1500	S_Shore	0.814±0.105	0.924±0.012	-0.109	2.13E-02	2.92E-09	-0.287
cg13845105	12	112846051	*RPL6*	5'UTR	N_Shore	0.681±0.054	0.79±0.127	-0.109	5.23E-03	8.49E-15	-0.370
cg01753198	16	70560099	*SF3B3*	5'UTR/1st intron	S_Shelf	0.713±0.046	0.822±0.104	-0.109	1.10E-03	4.16E-08	-0.266
cg15114651	19	47289410	*SLC1A5*	5'UTR/1st intron	N_Shore	0.467±0.099	0.576±0.156	-0.109	4.40E-02	1.32E-20	-0.437
cg03591223	14	23388579	*RBM23*	TSS200	Island	0.048±0.005	0.157±0.183	-0.109	2.16E-02	2.20E-33	-0.546
cg26056577	1	6526583	*TNFRSF25*	TSS1500	N_Shelf	0.719±0.082	0.828±0.036	-0.109	6.57E-03	1.52E-09	-0.292
cg22129364	1	53067894	*GPX7*	TSS200	Island	0.268±0.103	0.376±0.153	-0.109	4.74E-02	9.39E-25	-0.477
cg07026490	16	57837114	*KIFC3*	TSS1500	S_Shore	0.589±0.059	0.698±0.066	-0.108	8.40E-04	2.73E-06	-0.229
cg01780943	1	211499648	*TRAF5*	TSS1500	N_Shore	0.35±0.081	0.458±0.116	-0.108	1.31E-02	9.87E-15	-0.369
cg26057752	7	44104935	*PGAM2*	1st exon	Island	0.558±0.108	0.666±0.094	-0.108	3.02E-02	2.67E-16	-0.389
cg04908625	3	123166882	*ADCY5*	1st exon	Island	0.639±0.046	0.747±0.065	-0.108	1.10E-04	4.67E-13	-0.346
cg04064675	11	19139135	*ZDHHC13*	5'UTR/1st intron	Island	0.117±0.074	0.225±0.192	-0.108	4.92E-02	2.80E-09	-0.287
cg18439358	17	41910968	*MPP3*	TSS1500	S_Shore	0.085±0.056	0.193±0.162	-0.108	1.91E-02	1.66E-06	-0.233
cg12992443	20	35093928	*DLGAP4*	5'UTR/1st intron	S_Shelf	0.71±0.068	0.818±0.017	-0.108	2.66E-03	3.19E-14	-0.362
cg20455854	5	139040849	*CXXC5*	5'UTR/1st intron	Island	0.639±0.084	0.747±0.122	-0.108	1.71E-02	1.31E-07	-0.256
cg19738774	11	68778850	*MRGPRF*	5'UTR/1st intron	Island	0.421±0.058	0.529±0.174	-0.108	2.78E-02	4.58E-11	-0.317
cg15709065	3	134090737	*AMOTL2*	5'UTR/1st intron	N_Shore	0.64±0.065	0.747±0.111	-0.108	5.34E-03	5.75E-11	-0.315
cg17462140	11	2162363	*IGF2*	5'UTR	Island	0.463±0.078	0.57±0.14	-0.108	2.01E-02	2.83E-05	-0.205
cg17150663	7	128551672	*KCP*	TSS1500	S_Shore	0.622±0.052	0.729±0.075	-0.107	4.61E-04	1.20E-08	-0.276
cg01385679	20	45033155	*ELMO2*	5'UTR/1st intron	N_Shore	0.655±0.096	0.762±0.079	-0.107	1.81E-02	4.41E-06	-0.224
cg23366166	11	125366506	*FEZ1*	TSS1500	S_Shore	0.574±0.073	0.681±0.151	-0.107	2.28E-02	1.28E-05	-0.213
cg23882186	2	99065421	*INPP4A*	5'UTR/1st intron	S_Shelf	0.804±0.06	0.911±0.025	-0.107	1.27E-03	1.19E-06	-0.237
cg20186396	17	80275498	*CD7*	TSS200	N_Shelf	0.628±0.072	0.735±0.057	-0.107	3.52E-03	2.76E-19	-0.423
cg21658277	22	39745603	*SYNGR1*	TSS1500	N_Shore	0.145±0.077	0.252±0.133	-0.107	1.72E-02	3.87E-14	-0.361
cg18707845	15	32909968	*ARHGAP11A*	5'UTR	S_Shelf	0.728±0.083	0.835±0.066	-0.107	8.01E-03	1.82E-16	-0.391
cg00869668	17	1549012	*SCARF1*	1st exon	S_Shore	0.641±0.057	0.748±0.055	-0.107	6.16E-04	2.22E-11	-0.322
cg10249734	17	80292079	*SECTM1*	TSS200	Island	0.743±0.086	0.849±0.027	-0.107	9.68E-03	2.29E-24	-0.473
cg05836145	7	20827117	*SP8*	TSS1500	S_Shore	0.43±0.06	0.537±0.135	-0.106	1.06E-02	8.76E-07	-0.239
cg02075820	11	17300909	*NUCB2*	5'UTR/1st intron	S_Shelf	0.59±0.1	0.696±0.092	-0.106	2.42E-02	1.36E-14	-0.367
cg07161827	2	71680559	*DYSF*	TSS200	Island	0.143±0.055	0.25±0.19	-0.106	4.07E-02	2.16E-14	-0.365
cg07324245	17	75445905	*SEPT9*	TSS1500	N_Shore	0.549±0.097	0.655±0.16	-0.106	4.95E-02	5.68E-12	-0.331
cg06674062	7	94285887	*SGCE*	TSS1500	Island	0.469±0.053	0.575±0.162	-0.106	2.02E-02	2.60E-25	-0.482
cg06674062	7	94285887	*PEG10*	5'UTR/1st exon	Island	0.469±0.053	0.575±0.162	-0.106	2.02E-02	4.61E-09	-0.284
cg18273817	1	8936178	*ENO1*	5'UTR/1st intron	N_Shore	0.691±0.114	0.797±0.088	-0.106	3.94E-02	4.21E-21	-0.442
cg25320328	1	92953037	*GFI1*	TSS1500	S_Shore	0.548±0.079	0.654±0.159	-0.106	3.28E-02	1.63E-06	-0.234
cg16836311	1	25944712	*MAN1C1*	1st exon	S_Shore	0.771±0.076	0.876±0.147	-0.106	2.42E-02	3.48E-05	-0.202
cg24047810	11	2162341	*IGF2*	5'UTR	Island	0.562±0.065	0.668±0.107	-0.106	5.22E-03	1.91E-06	-0.232
cg07969769	5	49961846	*PARP8*	5'UTR/1st exon	N_Shore	0.224±0.084	0.329±0.086	-0.105	1.10E-02	4.28E-06	-0.224
cg19692584	10	96161889	*TBC1D12*	TSS1500	N_Shore	0.763±0.092	0.868±0.046	-0.105	1.42E-02	1.81E-05	-0.210
cg14160518	20	50418952	*SALL4*	5'UTR/1st exon	Island	0.768±0.103	0.873±0.065	-0.105	2.50E-02	6.44E-20	-0.429
cg05753799	20	3746315	*C20orf27*	5'UTR/1st intron	N_Shore	0.641±0.068	0.746±0.046	-0.105	2.59E-03	5.68E-06	-0.221
cg02971262	9	139925395	*C9orf139*	5'UTR/1st intron	S_Shore	0.39±0.102	0.495±0.13	-0.105	4.12E-02	1.70E-06	-0.233
cg06285333	16	67516546	*ATP6V0D1*	TSS1500	S_Shore	0.724±0.04	0.829±0.022	-0.105	7.62E-05	4.79E-06	-0.223
cg19473623	7	7224869	*C1GALT1*	5'UTR/1st intron	S_Shore	0.733±0.071	0.837±0.068	-0.104	3.72E-03	2.36E-28	-0.507
cg02610070	1	228673723	*RNF187*	TSS1500	N_Shore	0.643±0.079	0.748±0.12	-0.104	1.61E-02	3.75E-07	-0.247
cg04057642	17	45730222	*KPNB1*	5'UTR	S_Shore	0.719±0.103	0.823±0.065	-0.104	2.54E-02	6.12E-10	-0.299
cg00152942	16	57125755	*CPNE2*	TSS1500	N_Shore	0.691±0.05	0.795±0.082	-0.104	7.34E-04	6.66E-08	-0.262
cg13502716	3	52281709	*PPM1M*	5'UTR	S_Shore	0.78±0.102	0.884±0.032	-0.104	2.40E-02	6.87E-17	-0.396
cg15253816	17	66255259	*ARSG*	TSS200	Island	0.273±0.095	0.377±0.146	-0.104	4.37E-02	6.79E-13	-0.344
cg21886367	14	24780825	*LTB4R*	5'UTR/1st exon	Island	0.498±0.078	0.602±0.175	-0.104	4.72E-02	1.11E-16	-0.393
cg16359169	19	12076030	*ZNF763*	5'UTR/1st intron	Island	0.149±0.061	0.252±0.121	-0.103	8.20E-03	1.87E-09	-0.291
cg23616741	7	44105434	*PGAM2*	TSS1500	S_Shore	0.43±0.053	0.533±0.183	-0.103	3.93E-02	4.83E-10	-0.300
cg21395519	3	122746985	*SEMA5B*	TSS1500	Island	0.126±0.058	0.229±0.154	-0.103	2.09E-02	1.04E-05	-0.215
cg02592727	19	55574733	*RDH13*	5'UTR	Island	0.159±0.078	0.262±0.17	-0.103	4.44E-02	4.77E-12	-0.332
cg09138671	1	20125846	*TMCO4*	5'UTR/1st intron	N_Shore	0.255±0.094	0.358±0.144	-0.103	4.26E-02	1.46E-10	-0.309
cg15272684	1	36024473	*NCDN*	5'UTR/1st intron	S_Shore	0.675±0.092	0.778±0.055	-0.103	1.56E-02	4.90E-16	-0.385
cg15272684	1	36024473	*KIAA0319L*	TSS1500	S_Shore	0.675±0.092	0.778±0.055	-0.103	1.56E-02	4.72E-06	-0.223
cg25763292	10	115442231	*CASP7*	5'UTR/1st intron	S_Shelf	0.762±0.059	0.865±0.025	-0.103	1.35E-03	1.11E-06	-0.237
cg27427527	15	71146902	*LARP6*	TSS1500	S_Shore	0.134±0.078	0.236±0.153	-0.103	3.69E-02	1.28E-07	-0.257
cg09349343	6	33245717	*B3GALT4*	1st exon	S_Shore	0.481±0.052	0.584±0.058	-0.102	4.44E-04	4.48E-08	-0.265
cg13892570	1	159893182	*TAGLN2*	5'UTR/1st intron	N_Shore	0.102±0.058	0.204±0.146	-0.102	1.76E-02	1.46E-26	-0.493
cg13660372	7	94285873	*SGCE*	TSS1500	Island	0.516±0.046	0.618±0.129	-0.102	7.07E-03	3.28E-27	-0.498
cg13660372	7	94285873	*PEG10*	5'UTR/1st exon	Island	0.516±0.046	0.618±0.129	-0.102	7.07E-03	3.29E-13	-0.349
cg23336810	21	43936497	*SLC37A1*	5'UTR	S_Shore	0.755±0.114	0.857±0.017	-0.102	3.98E-02	5.90E-28	-0.504
cg03137131	4	2366672	*ZFYVE28*	TSS200	Island	0.533±0.033	0.635±0.122	-0.102	3.34E-03	2.62E-05	-0.206
cg26678012	15	78857577	*CHRNA5*	TSS1500	N_Shore	0.108±0.061	0.21±0.139	-0.102	1.54E-02	1.41E-05	-0.212
cg01519063	8	102505293	*GRHL2*	5'UTR/1st intron	S_Shore	0.691±0.087	0.793±0.111	-0.102	2.26E-02	2.04E-26	-0.491
cg06970461	2	96782027	*ADRA2B*	TSS200	Island	0.442±0.093	0.544±0.143	-0.101	4.29E-02	3.51E-11	-0.319
cg07813265	1	100820528	*CDC14A*	5'UTR	S_Shore	0.743±0.074	0.844±0.063	-0.101	5.93E-03	1.83E-10	-0.307
cg14207210	17	56833195	*PPM1E*	TSS200	Island	0.128±0.058	0.229±0.173	-0.101	3.76E-02	6.93E-06	-0.219
cg20576160	8	17016860	*ZDHHC2*	5'UTR/1st intron	S_Shelf	0.728±0.078	0.828±0.106	-0.101	1.44E-02	4.84E-07	-0.245
cg19278744	4	78079628	*CCNG2*	5'UTR/1st intron	S_Shore	0.197±0.065	0.297±0.172	-0.100	4.13E-02	7.57E-06	-0.219
cg01418527	7	5631189	*FSCN1*	TSS1500	N_Shore	0.7±0.085	0.801±0.159	-0.100	4.83E-02	4.54E-13	-0.347
cg04926767	11	62476194	*BSCL2*	TSS1500	N_Shore	0.677±0.041	0.777±0.042	-0.100	4.98E-05	3.58E-06	-0.226
cg00653615	1	151029327	*CDC42SE1*	5'UTR/1st intron	N_Shelf	0.429±0.076	0.53±0.114	-0.100	1.55E-02	1.08E-09	-0.295

[a]Probe IDs for the Infinium MethylationEPIC BeadChip (Illumina). [b]National Center for Biotechnology Information (NCBI) Database (Genome Build 37/hg19). [c]TSS1500, from 200 bp upstream of the transcription start site (TSS) to 1500 bp upstream of it; TSS200, from the TSS to 200 bp upstream of it; UTR, untranslated region; Gene body, second exon and downstream on the RefSeq database (http://www.ncbi.nlm.nih.gov/refseq/). [d]N-Shelf, 2000 bp region 5' adjacent to N-Shore; N-Shore, 2000 bp region 5' adjacent to CpG island; S-Shore, 2000 bp region 3' adjacent to CpG island; S-Shelf, 2000 bp region 3' adjacent to SShore based on the UCSC Genome Browser (https://genome.ucsc.edu/). [e]Evaluated using the Cancer Genome Atlas database (https://www.cancer.gov/about-nci/organization/ccg/research/structural-genomics/tcga).

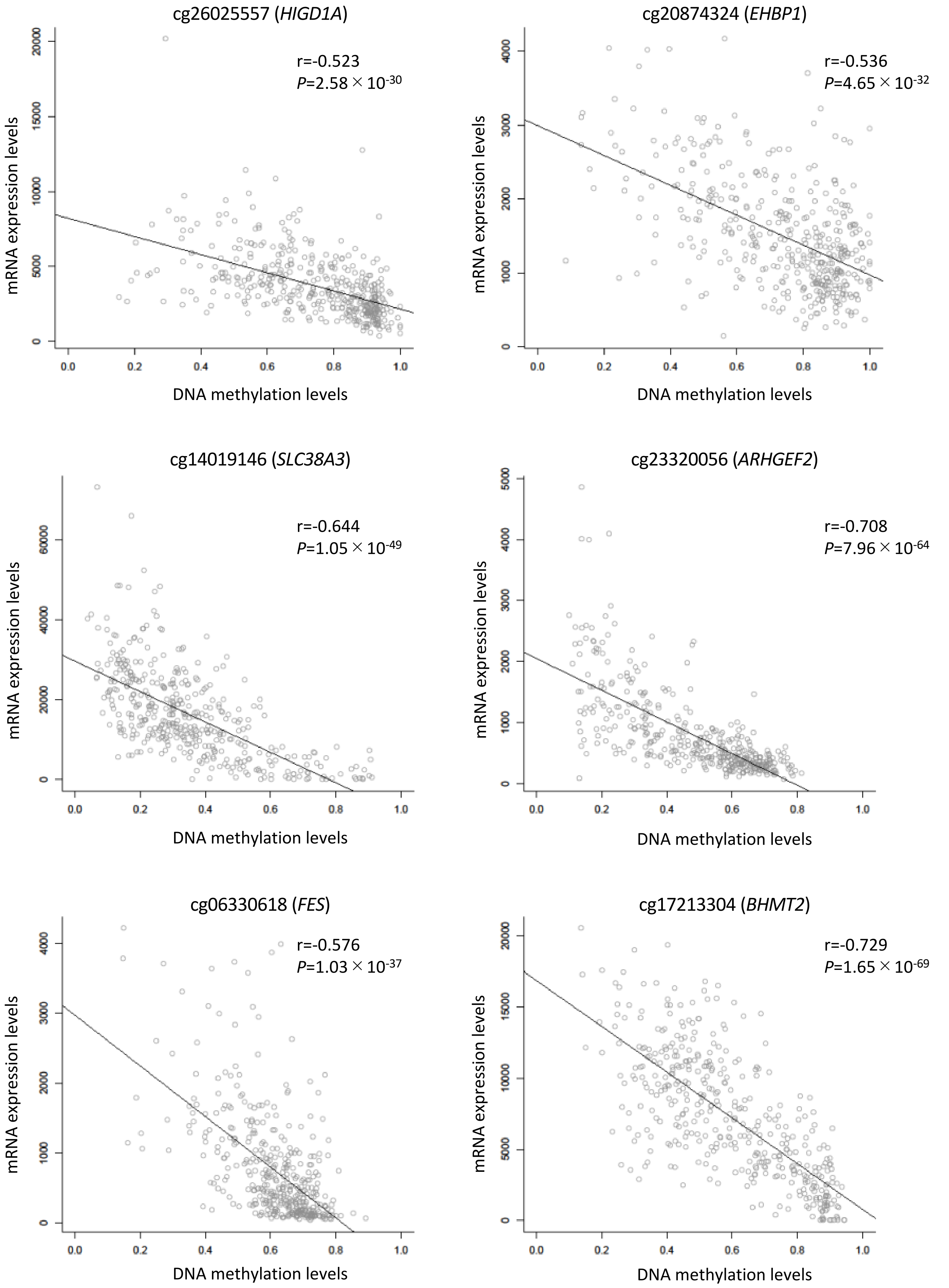

Supplementary Fig. 1 Significant inverse correlation between levels of DNA methylation and mRNA expression at representative CpG sites based on the data for non-cancerous and cancerous liver tissue deposited in the The Cancer Genome Atlas database (https://www.cancer.gov/about-nci/organization/ccg/research/structural-genomics/tcga). Infinium probe IDs and gene symbols are shown at the top of each panel.

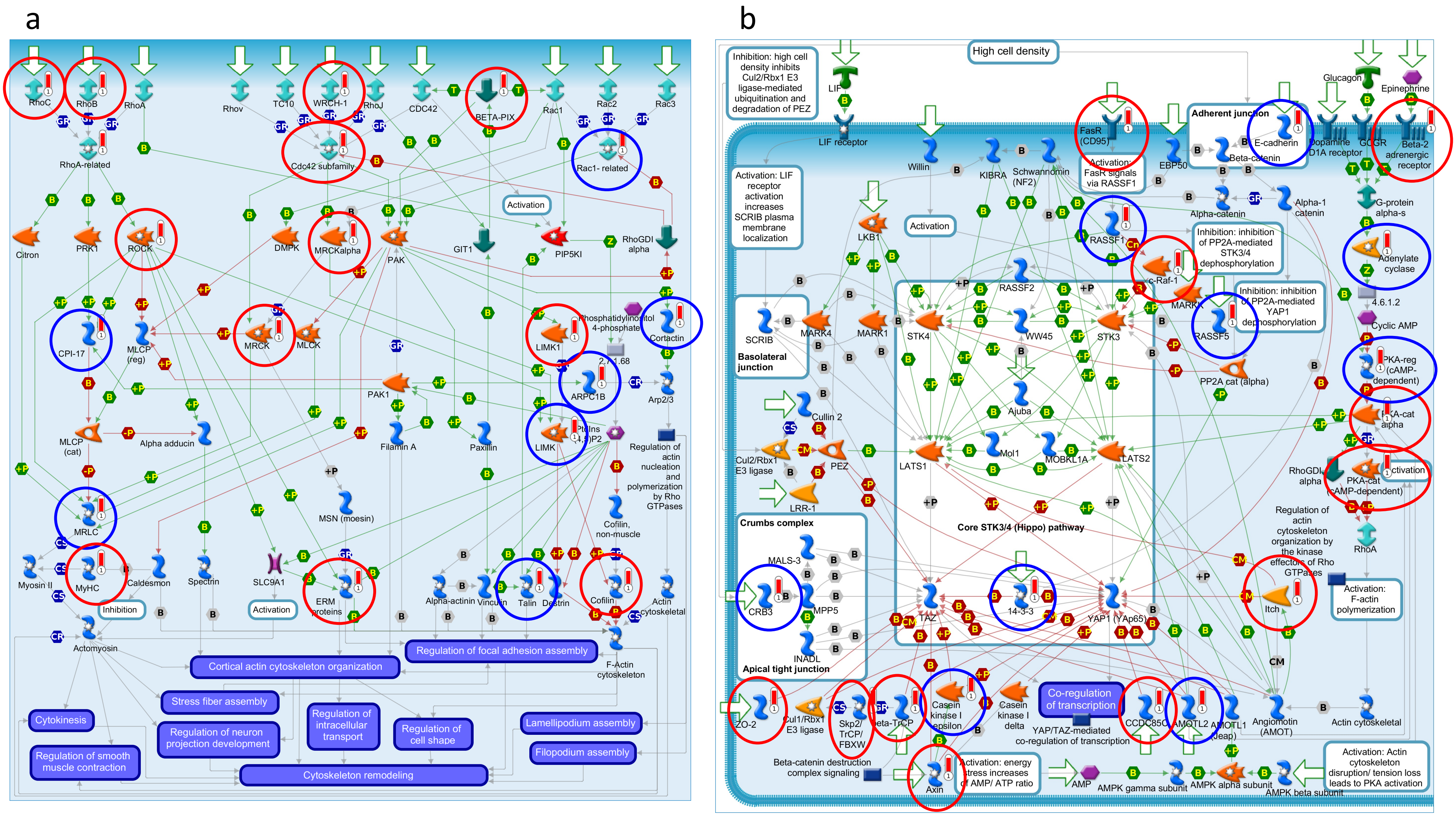

Supplementary Fig. 2 Pathway maps of “Cytoskeleton remodeling_Regulation of actin cytoskeleton organization by the kinase effectors of Rho GTPases” (false discovery rate [FDR]=5.49 × 10^{-6}) (a) and “Development_Positive regulation of STK3/4 (Hippo) pathway and negative regulation of YAP/TAZ function” (FDR=8.64 × 10^{-5}) (b). Network objects whose DNA methylation levels were significantly higher in Cluster I compared to Cluster II (*P*<0.05, $\Delta\beta_{I\text{-}II}$>0.1) and network objects whose DNA methylation levels were significantly lower in Cluster I compared to Cluster II (*P*<0.05, $\Delta\beta_{I\text{-}II}$<-0.1) are indicated with red and blue circles, respectively.

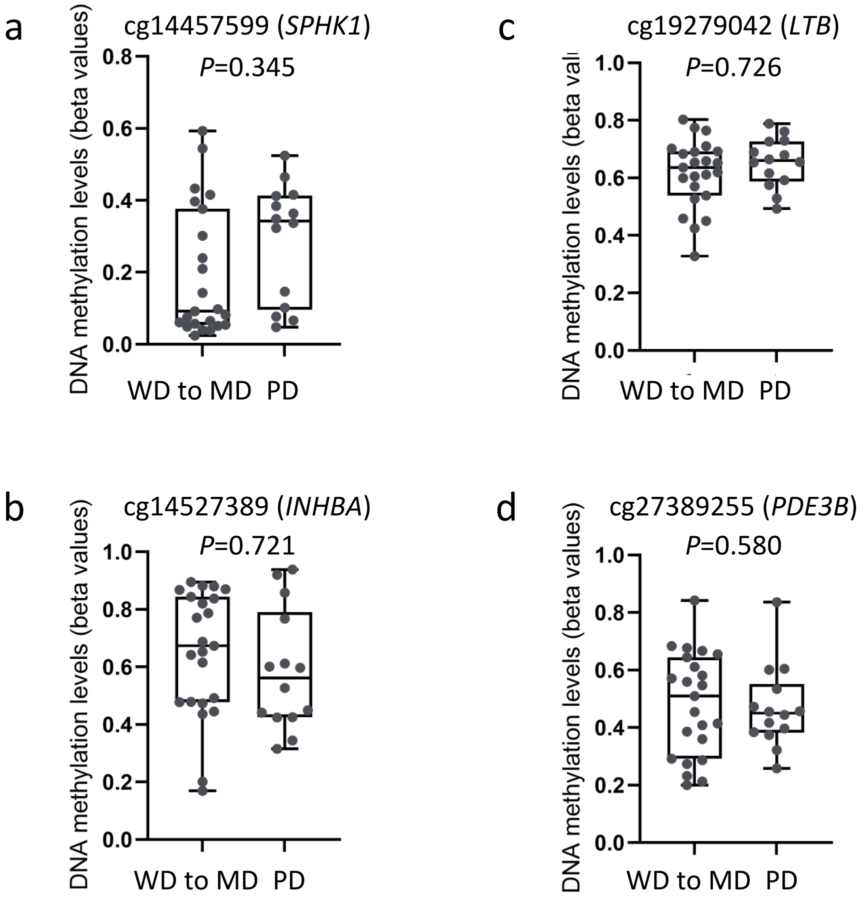

Supplementary Fig. 3 DNA methylation levels based on the Infinium assay for the *SPHK1* (a), *INHBA* (b), *LTB* (c) and *PDE3B* (d) genes in 37 samples of viral hepatitis-related hepatocellular carcinoma (HCC). Infinium probe ID is shown at the top of each panel. DNA methylation levels of these genes in poorly differentiated HCCs (PD) (n=14) are not significantly different from those in well to moderately differentiated HCCs (WD to MD) (n=23).
